# A Deeper Examination of *Thorellius atrox* Scorpion Venom Components with Omic Techonologies

**DOI:** 10.3390/toxins9120399

**Published:** 2017-12-12

**Authors:** Teresa Romero-Gutierrez, Esteban Peguero-Sanchez, Miguel A. Cevallos, Cesar V. F. Batista, Ernesto Ortiz, Lourival D. Possani

**Affiliations:** 1 Departamento de Medicina Molecular y Bioprocesos, Instituto de Biotecnología, Universidad Nacional Autónoma de México, Avenida Universidad 2001, Apartado Postal 510-3, Cuernavaca CP: 62210, Morelos, Mexico; teresaro@ibt.unam.mx; 2 Departamento de Microbiología Molecular, Instituto de Biotecnología, Universidad Nacional Autónoma de México, Avenida Universidad 2001, Apartado Postal 510-3, Cuernavaca CP: 62210, Morelos, Mexico; esteban.peguero@gmail.com; 3 Programa de Genómica Evolutiva, Centro de Ciencias Genómicas, Universidad Nacional Autónoma de México, Apartado Postal 510-3, Cuernavaca CP: 62210, Morelos, Mexico; mac@ccg.unam.mx; 4 Laboratorio Universitario de Proteómica, Instituto de Biotecnología, Universidad Nacional Autónoma de México, Avenida Universidad 2001, Apartado Postal 510-3, Cuernavaca CP: 62210, Morelos, Mexico; fbatista@ibt.unam.mx

**Keywords:** proteome, RNA-seq, *Thorellius*, transcriptome, Vaejovidae, venom, venom gland

## Abstract

This communication reports a further examination of venom gland transcripts and venom composition of the Mexican scorpion *Thorellius atrox* using RNA-seq and tandem mass spectrometry. The RNA-seq, which was performed with the Illumina protocol, yielded more than 20,000 assembled transcripts. Following a database search and annotation strategy, 160 transcripts were identified, potentially coding for venom components. A novel sequence was identified that potentially codes for a peptide with similarity to spider ω-agatoxins, which act on voltage-gated calcium channels, not known before to exist in scorpion venoms. Analogous transcripts were found in other scorpion species. They could represent members of a new scorpion toxin family, here named omegascorpins. The mass fingerprint by LC-MS identified 135 individual venom components, five of which matched with the theoretical masses of putative peptides translated from the transcriptome. The LC-MS/MS de novo sequencing allowed to reconstruct and identify 42 proteins encoded by assembled transcripts, thus validating the transcriptome analysis. Earlier studies conducted with this scorpion venom permitted the identification of only twenty putative venom components. The present work performed with more powerful and modern omic technologies demonstrates the capacity of accomplishing a deeper characterization of scorpion venom components and the identification of novel molecules with potential applications in biomedicine and the study of ion channel physiology.

## 1. Introduction

Scorpions are very successful carnivorous hunters that, except for the frozen poles and a few oceanic islands, inhabit all major terrestrial ecosystems of our planet [1]. Their success relies on the production of very potent neurotoxic venom that paralyzes and kills their preys and repels their competitors or predators. Scorpions are classified into 20 families with 208 genera, covering the 2231 species described to date [2]. Mexico is very rich in wildlife as a result of its wide range of ecosystems. These include over 12% of the described scorpion species, comprising 281 species belonging to 38 genera and 8 families, those of Buthidae (the one of medical importance), Caraboctonidae, Chactidae, Diplocentridae, Euscorpiidae, Superstitioniidae, Typlochactidae and Vaejovidae, being the latter the one with the highest diversity [2]. The family Vaejovidae is broadly distributed from Canada to Guatemala, but Mexico harbors the highest diversity, with 149 species belonging to 21 genera [2]. Within this family, the genus *Thorellius*, comprising some of the largest vaejovids, is endemic to central Mexico. It is distributed over the states of Aguascalientes, Colima, Guanajuato, Guerrero, Jalisco, Michoacán, Nayarit, Sinaloa and the State of Mexico [3].

Scorpion venoms are complex mixtures of different biologically active compounds, including enzymes (such as hyaluronidases, phospholipases, and proteases), toxic and cytolytic peptides, free amino acids, carbohydrates, lipids and other metabolites [4]. Many of the scorpion venom components constitute excellent leads for drug development [5] or are useful tools for physiological research. It is therefore of utmost importance to characterize their rich complexity. Less than 2% of the ca. 200,000 peptides estimated to be present in the venoms of the more than 2000 species, have been identified and/or characterized thus far [6]. One of the reasons for the gap in scorpion venom knowledge resides in the difficulties imposed by the need to collect a relative large number of specimens from natural environments to “milk” the venom in large-enough quantities as to allow the isolation of the less represented components. This procedure can have a negative impact on wild scorpion populations which, besides being important controllers of other arthropods’ populations, sustain other higher predators, therefore playing a relevant role in their ecosystems. The permits for specimens’ collection are becoming more restrictive regarding the particular species and the number of individuals that are authorized for collection. So, alternatives have to be devised to overcome the lack of specimens for classical biochemical characterization. Two technologies have helped partially dealing with this limitation in the recent past: the screening of cDNA libraries constructed from venom gland mRNAs (e.g., [7,8]) and the heterologous expression of the coded peptides for functional characterization (e.g., [9,10]). However, it is only with the advent of high throughput techniques for transcriptomic and proteomic analyses that it became possible to grasp the enormous diversity of scorpion venom components of peptidic nature [7,8]. Only a few individual scorpions are needed for the studies that can be performed with these two new technologies. It will depend on the animal and telson sizes, but for larger species the whole analysis can be performed with just one specimen.

Scorpions of the family Vaejovidae have been shown to contain biomolecules with promising therapeutic potential in their venoms, in particular, antimicrobial peptides [11]. We have previously reported the finding of several mRNAs coding for these peptides after cDNA library screenings in a few species. In particular, for *Vaejovis intrepidus*, 11 distinctive cDNA sequences coding for antimicrobial peptides were reported [12]. A more comprehensive study of this venom is necessary in order to decipher its whole potential as a source of antibiotics and other relevant compounds.

The taxonomy of the family Vaejovidae had been revised, and the particular subspecies that we worked with (*Vaejovis intrepidus atrox)* had been elevated to species, reassigned to the *Thorellius* genus, and therefore renamed as *Thorellius atrox* [13]. It will be referred henceforth as *T. atrox*; this is a scorpion species with a relatively large area of distribution in the states of Colima and Jalisco, but hard to collect due to its low abundance. The need for a more in depth study of the venom components of this species, together with its scarcity, make *T. atrox* a good candidate for high throughput transcriptomic and proteomic analyses. Here we show that, with just a few collected specimens, a detailed analysis of the venom composition can be performed.

## 2. Results and Discussion

### 2.1. RNA Extraction, RNA-Seq and Transcriptome Assembly

From four dissected telsons, 2.1 μg of pure total RNA were obtained. The RNA quality was assessed with the Bioanalyzer. As reported in other scorpion transcriptome analyses [8], the 70 °C-heating step in the RNA purification procedure resulted in the absence of the 28S rRNA peak in the electropherogram, so the RNA Integrity Number (RIN) could not be determined. However, no peaks associated with RNA degradation were observed, reflecting the excellent integrity of the produced total RNA and its suitability for the cDNA library construction. The quality of the Illumina-produced sequences further confirmed the adequacy of the extracted RNA. Paired-end sequencing (2 × 72 bp) was performed at the Massive DNA Sequencing Facility at the Institute of Biotechnology (Cuernavaca, México) with a Genome Analyzer IIx (Illumina, San Diego, CA, USA).

A total of 44,049,844 reads were obtained by the RNA-seq procedure. The Trinity assembly resulted in a total of 129,950 transcripts, with an N50 of 1849 bp. Of those transcripts, 20,851 were successfully annotated by Trinotate. The generated reads, in fastq format, were submitted to European Nucleotide Archive (ENA) and were registered with a study accession number PRJEB23004.

### 2.2. Transcriptome Analysis

As a first approach, the annotated transcripts were classified in accordance to GO categories (Gene Ontology Consortium, http://www.geneontology.org). At the broadest level of ontology, 41% of the transcripts were classified as Biological Process, 33% as Cellular Component, and 26% as Molecular Function (Supplementary Figure S1).

**Figure 1:**
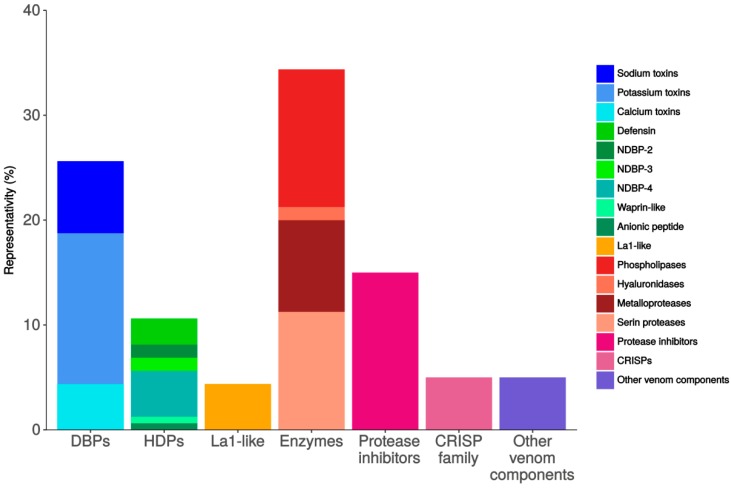
Relative diversity of the annotated transcripts putatively coding for venom components in accordance to protein families and subfamilies. The abundance of the particular transcripts is not considered. The group with the highest representation is that of the enzymes.

By sequence similarity, 160 annotated transcripts were identified as potentially coding for scorpion venom components. Of those, 41 correspond to cysteine-rich sequences (DBPs, including putative toxins acting on sodium, potassium and calcium channels), 17 are classified as Host Defense Peptides (HDPs, including members of the non-disulfide-bound peptide families NDBP-2, NDBP-3, NDBP-4, anionic peptides, waprin-like peptides and defensins), 55 putative enzymes (metalloproteases, phospholipases, hyaluronidases and serine proteases), 7 La1-like peptides, 24 protease inhibitors, 8 cysteine-rich secretory proteins (CRISPs, members of the CAP superfamily) plus 8 other venom components of unknown function (Figure 1 and Supplementary Table S1). 

### 2.3. Transcript Nomenclature

There is no standard nomenclature for naming RNAseq-generated transcripts in the literature, with authors frequently using the unmodified outputs from the assemblers to name the transcripts in their reports. To avoid confusion, we follow here transcript name codes that are both intuitive and easy to standardize. Every transcript reported is named as follows: The first three characters define the species (Tat, from *T. atrox*, in our case). The next three characters define the family of the encoded peptide/protein with respect to its putative function, followed by another three characters related to the subtype. The last two digits indicate the transcript number. Table 1 resumes this nomenclature for all the transcripts reported for *T. atrox.* In case a transcript is found with the same sequence as a previously reported one, the original name is honored to avoid duplications in databases.

### 2.4. DBPs

In scorpion venoms, the disulfide-bound peptides (DBPs) are mainly represented by the ion channel-acting toxins. These are peptides with 28 to 120 amino acids, constrained by 3 to 5 disulfide bonds. These toxins can specifically interact with sodium, potassium or calcium channels, altering the physiology of the cells, tissues and organs that can cause severe intoxications, sometimes ending with the death of the stung animal [14,15]. The DBPs are typically toxic to mammals, insects and crustaceans, and constitute the scorpions’ main weapons for predation and defense [16].

The analysis of the *T. atrox* transcriptome revealed the presence of 41 transcripts whose encoded sequences showed similarity to previously-reported scorpion toxins. They are described below in accordance to their structural family and target channel.

#### 2.4.1. Toxins Acting on Voltage-Gated Sodium Channels

Toxins acting on voltage-gated sodium channels (NaTxs) have been commonly found in scorpion venoms. They are peptides with 58–76 amino acids, stabilized by 3 or 4 disulfide bridges [17] that modify the channel’s opening or closing gating kinetics. They have been classified into two families based on their physiological effect on the channels: α-NaTxs and β-NaTxs [18]. The alpha toxins bind to the voltage-gated sodium channels at their site 3 and inhibit the normal inactivation process of the channels. The beta toxins bind to receptor site 4 and shift the threshold of the channel activation, resulting in the channel opening at more negative potentials [14,19,20]. The NaTxs are the main toxic component of the scorpion venoms and are responsible for most of the intoxication symptoms. It has been previously shown that the venoms of the scorpions belonging to the family Buthidae (which includes the majority of the species dangerous to humans) are more rich and diverse in NaTxs than those belonging to non-Buthidae families [4].

**Figure 2:**
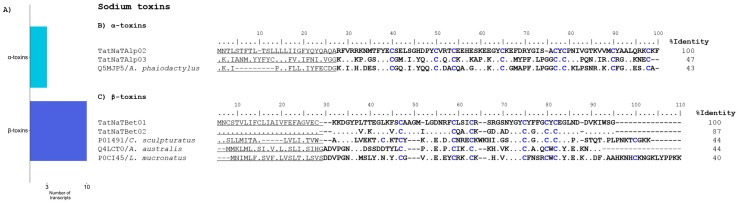
The putative sodium channel-acting toxins derived from the *T. atrox* transcripts. (**A**) Distribution of the found transcripts into alpha and beta NaTx subfamilies. (**B**) Alignment of the translated complete CDS potentially coding for α-NaTxs with their closest matches. (**C**) Alignment of two precursors derived from transcripts potentially coding for β-NaTxs with their closest matches. In all the alignments shown in figures in this report, points indicate sequence identity and dashes indicate gaps. When present, the sequence elements are shown as follows: predicted signal peptides are underlined, mature peptides are in bold type with the cysteine arrays highlighted in blue, and propeptides are in italics. The UniProt/GenBank identifiers precede the name of the scorpion species for the reference sequences. The identity percentages are always calculated for the whole sequences shown, including the signal peptides and propeptides when present.

We identified 13 transcripts potentially coding for NaTxs in the transcriptome of *T. atrox*. Three of them showed sequence similarity to previously-reported α-NaTxs and 10 to β-NaTxs (Figure 2A and Supplementary Table S1) and two of these sequences corresponded to complete α-NaTxs coding sequences (CDS). Their closest match in terms of sequence similarity was the precursor of a toxin from *Anuroctonus phaiodactylus* (now known as *Anuroctonus pococki bajae* [21]) (UniProt Q5MJP5), with 47% and 43% sequence identity, respectively (Figure 2B). This reference toxin, originally named phaiodotoxin, is an insect-specific sodium channel-acting toxin. It defines an independent structural class, and has a peculiar biological activity on the para/tipE sodium insect channel, with both alpha (predominantly) and beta components [22].

Of the 10 transcripts potentially coding for β-NaTxs, 6 were obtained with the complete CDS (Supplementary Table S1). As an example, the two longest encoded sequences were chosen for the alignment in Figure 2C. Their closest matches in terms of sequence similarity were the precursors of CsEI (UniProt P01491) from the scorpion *Centruroides sculpturatus* and of LVP1-alpha (UniProt P0CI48) from *Lychas mucronatus*. The CsEI toxin is lethal to chickens, and mildly toxic to mice and crickets (unpublished data from our lab), while a fragment from the LVP1-alpha (Lipolysis-activating peptide 1-alpha chain) transcript could code for a protein with sequence similarity to neurotoxin BmKBTx from *Mesobuthus martensii* [23].

Two transcripts assigned here to β-NaTxs are interesting since their closest match by the blastp algorithm was toxin KAaH1 (UniProt Q4LCT0) from the scorpion *Androctonus australis*, a known blocker of the Kv1.1 and Kv1.3 voltage-gated potassium channels, and a weak beta toxin [24]. KAaH1 has been proposed as a member of an independent family of scorpion toxins related to the sodium toxins in terms of sequence, but displaying weak beta activity on sodium channels, while in contrast, being potent potassium channel blockers. The putative toxins derived from transcripts TatNaTBet09 and TatNaTBet10 found in this study could be other members of the same group, but their real activity has to be tested experimentally.

The relatively low number of transcripts found coding for NaTxs is in accordance with what has been reported for other non-buthid scorpions [25]. It is relevant to notice that in the previous effort made to describe the transcripts from the venom gland of this species, those coding for NaTxs were even less represented, with no α-NaTxs found and just one β-NaTx reported: ViNaTx1. Intriguingly, the exact sequence of ViNaTx1 was not found in the present study, whereas a very similar homolog with just 2 amino acid changes out of 58 (96.5% identity at the mature protein level), TatNaTBet08, is reported. This variability is to be expected and can be attributed to the normal intraspecific toxin gene diversity.

#### 2.4.2. Toxins Acting on Potassium Channels

**Figure 3:**
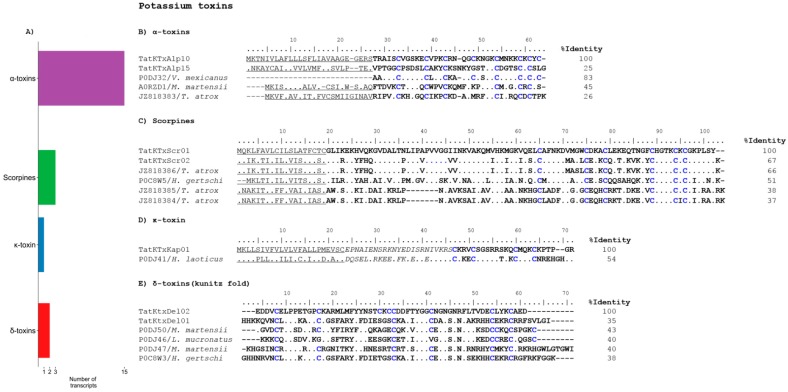
Potassium channel-acting toxins derived from the *T. atrox* transcripts. (**A**) Distribution of the found transcripts with respect to their subfamilies. (**B**) Two of the precursors of α-KTxs derived from transcripts are shown aligned to the sequences of their closest matches by BLAST. (**C**) The precursors of the scorpine-like peptides of the β-KTxs subfamily aligned to previously reported sequences from this species and HgeScplp2 as reference. An exact sequence to the one indicated as ViScplp2 was also found in this work. (**D**) The precursor identified for the κ-KTx aligned to its closest BLAST match. (**E**) The encoded mature sequence of the found δ-KTxs aligned to other known scorpion Kunitz-type peptides.

Toxins acting on potassium channels are structurally constrained peptides stabilized by 3 or 4 disulfide bridges [26] that are essentially blockers of the potassium channels. They have been classified into five subfamilies: α-, β-, and γ-KTxs with a cysteine-stabilized α/β motif (CSα/β), the κ-subfamily with a CSα/α motif and the δ-KTx with a Kunitz-type fold. There are also the scorpine-like peptides, proteins with two domains, one of them with sequence similarity to the β-KTxs [27], this being the reason why they are sometimes considered as a subgroup within the β-KTxs. Twenty-one transcripts potentially coding for KTxs were identified in the venom gland transcriptome of *T. atrox* (Figure 3A).

The α-KTx subfamily is the most diverse one [20], with more than 170 peptides described to date according to Kalium database (http://kaliumdb.org/). These toxins are usually short peptides (20–40 amino acids, ca. 4000 Da) that present the classical CSα/β motif and are mostly blockers of the potassium channels, either via a Lys-aromatic dyad (mainly), or through a patch of basic residues interacting with a negative extracellular loop of the channel [21]. We found 15 transcripts potentially coding for α-KTxs. Figure 3B shows two examples, aligned to other previously reported toxins or their precursors. One of the references is toxin Vm23 (UniPtot P0DJ32), from the scorpion *Vaejovis mexicanus*, which is a blocker of the voltage-gated Kv1.3 channel [28]. The sequence corresponding to the putative mature TatKTxAlp10 toxin and Vm23 share 83% of identity, with only 6 mismatching residues. They are both expected to be stabilized by four disulfide bonds. The other two references are precursors derived from cDNAs. The one coding for BmTX1 (UniProt A0RZD1) is from the scorpion *Mesobuthus martensii* and the other was previously obtained from the cDNA library from the venom gland of the here-reported species, *T atrox* (GenBank JZ8183), known previously as *V. intrepidus*. Transcript TatKTxAlp15 and these last two references encode for α-KTxs stabilized by three disulfide bonds.

The classical β-KTxs are long-chain peptides (50–75 amino acids) with three disulfide bonds. We do not found transcripts coding for the classical β-KTxs in our analysis. As indicated above, the scorpine-like peptides are also considered a subgroup within the β-KTxs. They are composed of two domains: an N-terminal cecropin-like domain displaying a clear antimicrobial activity [29] and a C-terminal domain with sequence and structural similarity to the β-KTxs. Due to their antimicrobial activity, they are also considered as members of the family of the host defense peptides defensins [30]. We had previously identified three sequences putatively coding for scorpine-like peptides in this scorpion species [12]. In this analysis, we also found three possible transcripts for scorpine-like peptides (Figure 3C). The sequence comparison revealed that one of the newly found transcripts codes for exactly the same peptide sequence as the previously-reported ViScplp2, and is 99% identical to the previously reported ViScplp1 sequence, with just one different amino acid. Peptides ViScplp1 and ViScplp2 (GenBank JZ818384 and JZ818385 respectively) are shorter than the reference peptide (HgeScplp2 (UniProt P0C8W5)), as found in a cDNA library from the scorpion *Hoffmanihadrurus gertschi* [31]), with an internal 7 amino acids deletion in the cecropin-like domain. The other two transcripts reported here do not lack those amino acids. One is TatKTxScr03, which is 99% identical to the previously reported ViScplp3 sequence (GenBank JZ818386), with also one different amino acid. The other is TatKTxScr01, which although relatively similar in sequence to the other scorpine-like peptides, had no counterpart in the cDNA library. Considering the expected intra-species variability, we can conclude that this transcriptomic approach covered and excelled the results obtained by the cDNA library screening for the scorpine-like peptides.

The κ-subfamily of potassium channel-acting toxins, with its atypical CSα/α fold [32] was also represented in this analysis. We found one transcript putatively coding for a κ-KTx, as illustrated by the sequence alignment shown in Figure 3D. As reference, its closest blastp match, toxin HelaTx1 (UniProt P0DJ41) from *Heterometrus laoticus* was used. HelaTx1 has been shown to block the Kv1.1 and Kv1.6 channels in a voltage-dependent fashion [33]. The TatKTxKap01 precursor contains the signal peptide, followed by a propeptide and the sequence of the mature κ-KTx with the typical four cysteines.

Finally, the δ-KTx family is integrated by peptides with a Kunitz-type scaffold, with dual activity as serine protease inhibitors and potassium channel blockers (mainly the Kv1.3) [34]. We found two transcripts for δ-KTxs. The mature sequences they encode are shown in Figure 3E, aligned with a selected group of other scorpion δ-KTxs: BmKTT-2 from *Mesobuthus martensii* (UniProt P0DJ50), (UniProt P0DJ46, UniProt P0DJ47, UniProt P0C8W3).

#### 2.4.3. Toxins Acting on Calcium Channels

**Figure 4:**
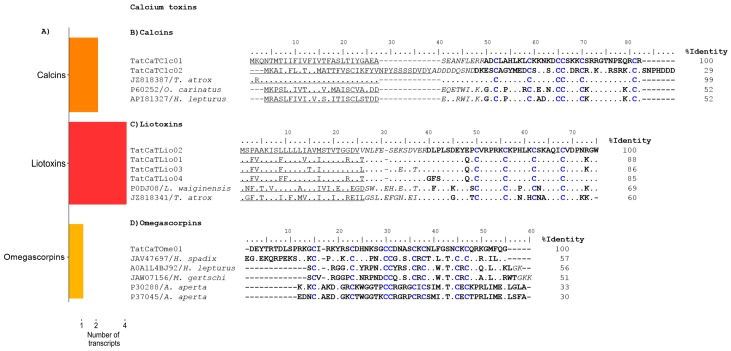
Putative calcium channel-acting toxins derived from the *T. atrox* transcripts. (**A**) Distribution of the found transcripts with respect to their types. (**B**) The precursors of calcins, aligned with the precursors of their closest matches by BLAST. (**C**) The precursors of the liotoxin-like peptides, aligned to the reference sequences. (**D**) The mature putative Ca_v_-acting toxin found in this work, and the other scorpion transcript-derived similar sequences from the databases, aligned to the type IV-ω-agatoxins from *A. aperta* as references.

Just a few scorpion toxins with activity on voltage-dependent calcium channels have been described to date. Examples are kurtoxin, isolated from *Parabuthus transvaalicus*, and kurtoxin-like I and II from *Parabuthus granulatus*. Kurtoxin is more closely related to the α-NaTxs than to other known calcium channel-gating modifiers in terms of sequence, nevertheless affects the T-, L-, N-, and P/Q-type voltage-gated calcium channels in neurons [35,36]. Kurtoxin-like I and II decrease T-type calcium channel activity in mouse spermatogenic cells [37,38]. On the other hand, toxins affecting ligand-activated calcium channels, particularly the calcium release channels/ryanodine receptors (RyRs), are quite commonly found in scorpion venoms. The analysis performed for the transcripts assembled for *T. atrox* revealed the presence of 7 sequences coding for possible calcium channel modifiers.They are show in Figure 4, distributed in letters B (2 sequences), C (4 sequences) and D (1 sequence).

Calcins are short (33–35 amino acids) scorpion peptides that bind with high affinity and specificity to the RyRs [39]. They induce the appearance of a long-lasting subconductance state in the channel that increases its overall open probability [40]. They are fundamentally basic peptides, able to translocate through the cell membrane, and are structurally characterized by an inhibitor cystine knot (ICK) motif. We found 2 transcripts putatively coding for calcins. The translated precursors are shown in Figure 4B, aligned to the precursors of intrepicalcin (GenBank JZ818387) (formerly ViCaTx1) from this same species, opicalcin-1 (UniProt P60252) from *Opistophthalmus carinatus*, and hemicalcin-1 (UniProt API81327) from *Hemiscorpius lepturus*, their closest matches by blastp. The precursor TatCaTClc01 differs from the precursor of ViCaTx1 by just one amino acid in the signal peptide, the mature peptides are identical, and correspond to intrepicalcin. Intrepicalcin is a proven active calcin. It was heterologously expressed and shown to be active on RyRs [41].

LaIT1, the first reported liotoxin-like peptide was isolated from the venom of *Liocheles australasiae* [42]. Later on, a similar peptide, Phi-LITX-Lw1a from *Liocheles waigiensis*, was shown to act on RyRs with a similar mode of action as scorpion calcins, but with significantly greater potency. Liotoxin-like peptides adopt the disulfide-directed hairpin (DDH) motif stabilized by two disulfide bonds [43]. We found 4 transcript sequences related to the liotoxin-like peptides. The translated precursors from these transcripts are shown in Figure 4C, aligned to two reference precursor sequences: VmCaTx1 (GenBank JZ818341) from *Vaejovis mexicanus* and Phi-LITX-Lw1a (UniProt P0DJ08). It is interesting to notice that notwithstanding the different origins of these toxins, with the species belonging to different families (*T. atrox* and *V. mexicanus* to Vaejovidae, while *L. waigiensis* to Hemiscorpiidae) the sequences of the liotoxin-like peptides are highly conserved. It is even more evident for the predicted mature peptides, with identities over 70%.

Scorpion toxins acting on voltage-gated calcium channels have not been commonly reported. Due to their structural similarity to the α-NaTxs, the identification of functional analogs of kurtoxin remains serendipitous, being basically impossible to make any prediction based on the sequence of transcripts. In contrast, other arachnids, e.g., the American funnel web spider *Agelenopsis aperta*, have a battery of toxins acting on voltage-gated calcium channels: the ω-agatoxins. These are presynaptic antagonists of voltage-gated calcium channels and have different specificities against various subtypes of these channels of insects and mammals [44]. We found a transcript from *T. atrox* which was identified by sequence similarity with other sequences putatively coding for ω-agatoxins, here named TatCaTOme01. A more detailed search of the databases resulted in three other scorpion transcripts of the same nature. We had previously reported a transcript from the scorpion *Megacormus gertschi* (UniProt JAW07156) that shared sequence similarity with U8-agatoxin-Ao1a from *Limulus polyphemus* [8], and now found two other unpublished transcripts from *Hadrurus spadix* (UniProt A0A1L4BJ92) and *Hemiscorpius lepturus* (GenBank JAV47697). Figure 4D shows the predicted mature sequences encoded by these scorpion transcripts and of the two *A. aperta* ω-agatoxins (UniProt P30288 and P37045 respectively) that were closer in terms of sequence and the conserved cysteine pattern. They correspond to type IV ω-agatoxins, which have been shown to display high affinity and specificity for the P/Q-type high-voltage-activated calcium channels, highly expressed in the cerebellum and associated with such diseases as Alzheimer’s, migraine and seizures. The activity of these peptides remains to be experimentally determined. To our knowledge, they have not been isolated from the scorpion venoms yet. They could define a completely new family of scorpion toxins, which we propose to name as “omegascorpins”. For the *H. lepturus* and *M. gerstchi* peptides we indicate in Figure 4D, that they end in a canonical signal for amidation (shown in italics), so we postulate that they have amidated C-termini, a feature found in μ-agatoxins and type III, but not in type IV ω-agatoxins [44].

### 2.5. Host Defense Peptides (HDPs)

**Figure 5:**
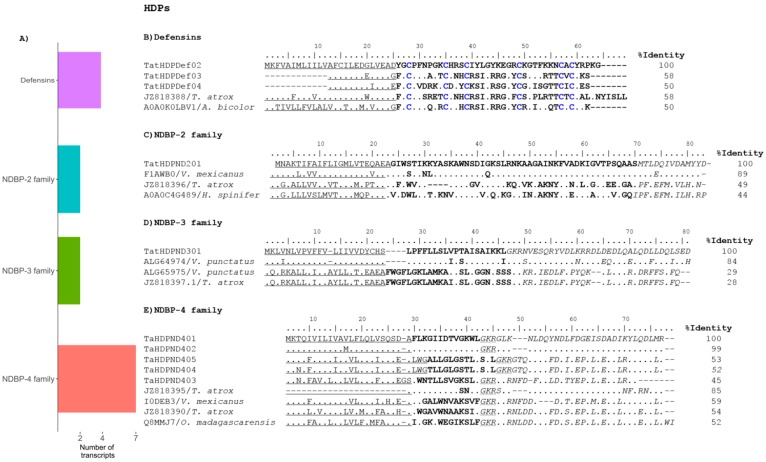
Possible Host Defense Peptides (HDPs) deduced from the transcriptome analysis. (**A**) Distribution of the found transcripts with respect to their types. The NDBPs are further expanded to show their families. (**B**) Precursors of the *T. atrox* β-defensins, aligned to reference precursors from other scorpion defensins. (**C**–**E**) The same sequence analysis for the precursors of the found NDBPs from families 2, 3 and 4, respectively.

Antimicrobial peptides are present in all forms of life and take part in the innate host defense response against any external agents [45]. Some of these peptides, besides their antimicrobial activities, can modulate the host immune system [46,47]. The HDPs can be divided into the cysteine-rich peptides, e.g., the defensins [48] and the non-disulfide-bound peptides (NDBPs). We identified 17 transcripts potentially coding for HDPs from the venom gland of *T. atrox*, which represents more than 10% of all the annotated venom-related transcripts, confirming previous findings in other non-buthid species (Figure 5A).

Defensins have been identified in three eukaryotic kingdoms: Animalia, Plantae and Fungi [49]. They are active against bacteria, fungi and viruses. In particular, the β-defensins are widely distributed. They are small (2–6 kDa) cationic peptides with structures stabilized by three disulfide bonds. They have been commonly found in the scorpion venoms, where they have even been proposed to be functionally and evolutionarily linked to neurotoxins [50]. We identified 4 transcripts with coding sequences related to the β-defensins (Supplementary Table S1). Three of the transcripts include the complete CDS and where selected for the sequence alignment shown in Figure 5B. As references, the precursors for β-defensins ViDef (GenBank JZ818388) (previously reported for this same species) and AbDef-1 (UniProt A0A0K0LBV1) from *Androctonus bicolor* were used.

A major group of HDPs in scorpion venoms is the one constituted by the NDBPs. They are usually small peptides, rich in cationic and hydrophobic residues. This combination results in a random coil structure in ionic aqueous solutions that make a transition to an amphipathic α-helix structure in the cell membrane environment [51]. Their precursor sequences usually contain a carboxy-terminal propeptide after the mature sequence. The NDBPs have attracted much attention due to the large number of valuable activities discovered in these peptides. They are very relevant for their antimicrobial activity, but some also display cytolytic, immunomodulatory, bradykinin-potentiating and anticancer activities, for which they have been proposed as potential leads for drug development [5]. The classification of the NDBPs supported solely on their sequence is not possible, since they are extremely variable. Their systematics is therefore based on their pharmacological activity, the elusive sequence similarity, and the peptide length [52]. Eleven transcripts (Supplementary Table S1) potentially coding for NDBPs are here reported, making the NDBPs the most diverse HDPs of the *T. atrox* venom.

The NDBP-2 family is composed of long chain multifunctional peptides, with 40–60 residues, rich in basic amino acids arginine and lysine. They are considered multifunctional for their antimicrobial, bradykinin-potentiating, insecticidal and anticancer activities. Two transcripts coding for these peptides were found in the transcriptome. One of the sequences was identical to a cDNA for an NDBP-2 previously found in *T. atrox*, ViVlp1 (GenBank JZ818396) [11] and is used as reference in the alignment in Figure 5C. Other two references used are vejovine (UniProt F1AWB0) from *Vaejovis mexicanus* and heterin-1 (UniProt A0A0C4G489) from *Heterometrus spinifer*, two close sequence matches. Vejovine has been shown to be effective against Gram-negative multidrug-resistant bacteria [53] and heterin-1 to both Gram-positive and Gram-negative bacteria [54]. The members of the NDBP-3 family are medium-length antimicrobial peptides (20–30 residues). Two transcripts were also identified for this family of peptides, one identical to the cDNA for the previously reported ViAMP1 (GenBank JZ818397.1). The other is shown in Figure 5D aligned to ViAMP1, VpAMP1.0 (UniProt ALG64974) and VpAMP2.0 (UniProt ALG64975), the last two previously identified in a cDNA library from *Vaejovis punctatus* [11]. For the NDBP-4 family, the short scorpion antimicrobial peptides, a similar situation was observed. Of the 7 identified transcripts, two were identical to previously described *T. atrox* cDNAs corresponding to ViCT2 (GenBank JZ818390) and ViCT7 (GenBank JZ818395) [11]. The precursors derived from 5 remaining transcripts are shown in Figure 5E, aligned with those of ViCT2 and ViCT7, plus VmCT1 (UniProt I0DEB3) and IsCT (UniProt Q8MMJ7) from *V. mexicanus* and *Opistachantus madagascarensis*, respectively. All the NDBP-4 family precursors found present the canonical amidation signal (*GKR*, at the start of the propeptide sequences in Figure 5D), so the mature peptides are expected to be amidated in the venom. It is worth noting that the sequences TatHDPND403 and TatHDPND404 share the same mature peptide and differ only in one residue in their signal peptides. In general, we can assert that this transcriptomic analysis was able to recover four of the NDBP sequences found in the previous cDNA library, while generating eight new precursor sequences potentially coding for seven new NDBPs. The physicochemical properties of these NDBPs are resumed in Table 2. No transcripts coding for peptides from the remaining two families, NDBP-1 and -5 were found.

Two other transcripts, coding for probable HDPs, are worth mentioning here. One is TatHDPAni01, which codes for a highly anionic peptide. Its closest match in terms of sequence similarity was Hta1 (55% identity), from a transcriptome analysis of *Hadogenes troglodytes*. Highly anionic peptides of this kind have been previously found in scorpions [55,56], and in other phyla. They are integral part of the host defense systems of vertebrates, invertebrates and plants [57]. The second is transcript TatHDPWap01, whose putative mature sequence shares 64% identity with the waprin-Enh1-like putative peptide derived from a transcriptome analysis of the spider *Parasteatoda tepidariorum* (GenBank XP_015928629.1). Waprins are ca. 50 amino acids-long peptides that have been identified mainly in snake venoms [58]. They are structural homologs of the whey acid protein (WAP) family, with a conserved four-disulfide-bonds arrangement. Waprins have been shown to be inhibitors of proteases, and antimicrobials with a role in the innate immune system [59]. The transcript found in *T. atrox* contains the complete CDS. This is the first time a sequence related to waprins is reported in scorpions.

### 2.6. Enzymes

The venom of scorpions is known for the presence of enzymes, which play an important role in toxicity and venom spreading in tissues [60]. A total of 55 transcripts (Supplementary Table S1) putatively coding for enzymes were identified for *T. atrox*. This accounts for about a third of all annotated transcripts, which is in remarkable agreement with the numbers found in other scorpion transcriptomic analyses, with the only exception of *Superstitionia donensis* [7]. The most diverse transcripts were those coding for phospholipases (21) and serine proteases (18), followed by metalloproteases (14) and a few (2) hyaluronidases (Supplementary Table S1). Only for four phospholipases (the smaller A2-type transcripts TatEnzPA201, TatEnzPA213 TatEnzPA202, plus the larger B-type transcript TaEnzPLB01) the complete CDS were successfully assembled. For the rest of the enzyme-coding transcripts, only partial CDS were obtained. From the *T. atrox* cDNA library only the partial CDS for one phospholipase A2 was recovered, named Vi20. The exact sequence of Vi20 was not found in our analysis. The closest sequence is TatEnzPA213, which shares 95.7% of identity with Vi20 (154 identical out of 161 overlapping residues for the mature sequence). No other enzymes were found in the *T. atrox* cDNA library. This could be a consequence of either the difficulties associated with the cloning of enzymes’ large cDNAs, or the criteria followed for colony selection for sequencing from the cDNA library. In any case, for the characterization of large transcripts, the RNA-seq methodology by far surpasses the potential of the cDNA library construction followed by standard sequencing.

### 2.7. Protease Inhibitors

Secreted proteases can inflict significant cellular damage if not tightly regulated [61]. Therefore, the scorpion venoms which are rich in proteases (as shown above) are also expected to contain protease inhibitors. That is reflected in our transcriptomic analysis, since 24 sequences were found which could potentially code for protease inhibitors (Supplementary Table S1). Although all were recovered as partial CDS, Pfam domains could be assigned to them, which corresponded to either serpin or Kunitz/Bovine pancreatic trypsin inhibitor domains, both being types of serine protease inhibitors. The majority of the transcripts corresponded to inhibitors of the serpin-type (19) and a few were of the Kunitz-type (5). No protease inhibitors were found while characterizing the sequences from the previous cDNA library from *T. atrox*.

### 2.8. Other Venom Components

Within this group, we describe other annotated transcripts found in the analysis that could code for venom peptides for which a particular function or molecular target has not been experimentally determined or demonstrated yet.

#### 2.8.1. La1-Like Peptides

**Figure 6:**
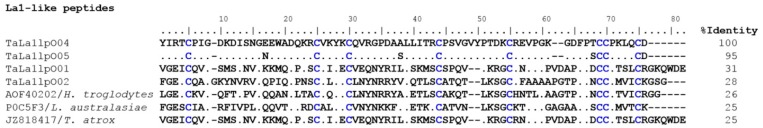
La1-like peptides coded by transcripts from *T. atrox*. Only the mature sequences were used in the alignment.

After the discovery of La1 as the most abundant component in the venom of the scorpion *Liocheles australasiae* [62], this kind of peptides (or transcripts coding for them) have been routinely found in other scorpions. La1 defines a family of peptides structurally characterized by a single domain Von Willebrand factor type C (SVWC); domain with four disulfide bridges. This is probably the most common scorpion venom constituent for which the molecular target or function is unknown. Some information has started to emerge on this regard. For example, spermaurin, a La1-like peptide from the venom of *Scorpio maurus palmatus*, has been shown to improve mammalian sperm motility [63]. Not surprisingly, 7 transcripts coding for La1-like peptides were found in our analysis, of which 6 had complete CDS (Supplementary Table S1). Not surprisingly also, one of the newly found transcripts codes for exactly the same sequence as the previously-reported ViLa1lp1 (GenBank JZ818417) from the same species, the only La1-like peptide recovered from that cDNA library. Two pairs of transcripts were identified by the Trinity assembler as “isoforms” (TatOthLa106 and -07; TatOthLa104 and -05 in Supplementary Table S1), so only one of each was chosen for the alignment shown in Figure 6, which covers the peptides’ mature sequence, includes ViLa1lp1, and uses the original La1 (UniProt P0C5F3) plus HtLa1 (UniProt A0F40202) from *Liocheles australasiae* and *Hadogenes troglodytes* as references. It is interesting to notice that the putative La1-like peptides, although coming from the same species, seem to be highly divergent in terms of sequence.

#### 2.8.2. CRISP Family

The Cysteine Rich Secretory Proteins (CRISP) are members of the CRISP, Antigen-5 and Pathogenesis-related (CAP) superfamily of proteins, which are broadly distributed through many animal kingdoms, including venomous animals. They have been associated with numerous paracrine and endocrine functions [64]. Transcripts coding for these proteins have been found in scorpion transcriptomes with low representation [8] and the peptides have been isolated from the venom of other venomous animals like snakes [65]. We identified 8 transcripts with partial CDS potentially coding for CRISPs in the transcriptome of *T. atrox* (Supplementary Table S1).

#### 2.8.3. Other Undefined Venom Components

Eight transcripts putatively coding for other venom components of unknown function complete the annotated transcripts described here (Supplementary Table S1). They match other scorpion venom or venom gland sequences from the databases, for which no information is available, and are grouped under the Undefined (Und) category.

## 3. Mass Spectrometry Analysis

**Figure 7:**
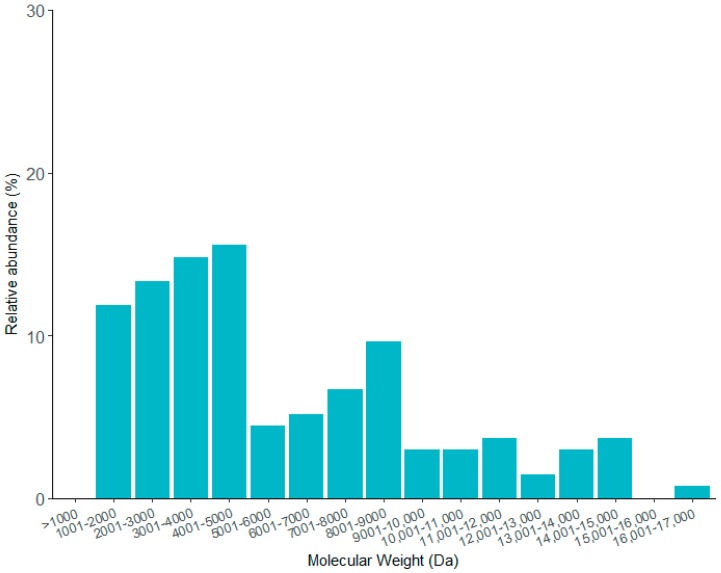
Relative distribution of the MW identified on the venom of *T. atrox* scorpion. Peptides between 1000 and 5000 Da are the most abundant, covering more than 50% of the components identified on the fingerprint.

A total of 135 components were identified (Table 3) using a bottom up LC-MS/MS technique. The MW range of the peptides were from 1077 to 16,920 Da with a median of 4506 Daltons (Da. As shown on Figure 7, the most abundant components are located between 1000 and 5000 Da, in this rank, we can find peptides with putative antimicrobial activity and toxins that affect potassium and calcium ionic channels, among others. It is worth mentioning that to the best of our knowledge this is the first proteome analysis conducted with venom from a Vaejovid scorpion.

Several proteomic studies of scorpion venoms of the Buthidae and non Buthidae families have been reported, for example, the fingerprint of the scorpion *Centruroides tecomanus*, reported by Valdez-Velazquez et al. [66]. In that study, 104 different components were identified, of which the majority fell within two molecular weight ranges, from 3000 to 5000 Da, and from 6000 to 8000 Da. The former usually correspond to the MW reported for toxins that affect potassium channels, whereas the later, correspond to toxins that affect sodium channels and are responsible for the toxicity to mammals. These findings contrast with the fingerprint here reported for the *T. atrox* venom, which has the highest amount of low molecular weight components (1000 to 5000 Da), suggesting a low abundance of sodium toxins. This is in accordance with its known non toxicity to mammals. Using as a database those theoretical MW determined on the mature sequence of each transcript related to venom component, a search for matching masses was performed on the fingerprint resulting in five matches with putative identity of: β-sodium toxins (TatNaTBet03 and TatNaTBet08), α-potassium toxins (TatKTxAlp09, TatKTxAlp10 y TatKTxAlp12), calcins (TatCaTClc01), HDPs (ViCT2) and La1-like peptides (ViLa1lp1) (Table 4).

The LC-MS/MS de novo sequencing allowed to reconstruct and identify 42 proteins (Supplementary Table S2) encoded by assembled transcripts distributed as follows: proteins with sequence identities to cellular components (15 proteins); enzymes (7) that include phospholipases, hyaluronidases and metalloproteinases; HDPs (7) with members of the NDBP-2, -3 and -4 family (Supplementary Figure S2 shows an example of mass spectra and sequence coverage of peptide TatHDPND403); DBPs (3) with putative potassium (scorpine like and k-KTx) and calcium channel toxins (calcin-like); La1-like peptides (2); CRISP-family members (2) and proteins without annotation (6) (See Table 5). 

Using the software Peaks Studio, 221 de novo fragments were identified (Supplementary Figure S2). It is worth noting that these fragments were not identified with the Sequest algorithm. Supplementary Figure S2 reports amino acid sequences found by LC-MS/MS which correspond to segments of peptides/proteins really present in the venom. When comparing these sequences with possible peptides/proteins identified by the transcriptomic analysis it seems that they are not included in Table 5 and Supplementary Table S3. Thus, they are *bona fide*, *de novo* sequences.

## 4. Conclusions

One frequent problem of the increased number of sequences submitted to databases, as a result of the employment of new generation massive sequence technologies, is the use of computer algorithms-derived naming for the sequences. They are usually meaningless and do not help with the annotation process. For reporting the annotated transcripts in this work, we used a simple and straightforward naming scheme. This method, as described, includes identifiers for the species, the peptide family by putative function, the peptide subtype and transcript number. We suggest that adherence to this scheme will facilitate the identification of the sequences by researchers in the future.

The power of the modern omic technologies was demonstrated in the characterization of the venom gland transcriptome and venom proteome of the *T. atrox* species. Despite their broad distribution, *T. atrox* specimens have very low population densities, so they are difficult to collect. Previous attempts to investigate the venom-related mRNA and peptide content in this species were limited by the availability of biological material. We were able to not only validate previous findings, but to generate new and richer valuable sequence information, all from just a few exemplars that were used for both RNA-seq and tandem MS. The annotation of 160 transcripts, coding for possible venom proteins, obtained by RNA-seq, versus only 20 from the cDNA library, supports this conclusion. The value of massive analysis is also reflected by the discovery of two novel sequence types, never before reported in scorpions: the omegascorpins, which share identity with spider ω-agatoxins, and the waprins, previously reported in insects and snakes. A large number of assembled transcripts remains unannotated, which reflects the lack of related annotated sequences in the databases. Thus, there is still an imperative need for the functional characterization of scorpion venom components other than those already studied.

## 5. Material and Methods

### 5.1. Biological Material

Four specimens of the *T. atrox* species were collected in the Coquimatlán locality, in Colima Mexico (19°12’39″ N 103°48’24″ W). They were properly classified (see Acknowledgements) and were kept in captivity at room temperature, with a natural light-dark cycle, provided with egg carton hideouts, fed with crickets on a weekly basis and with permanent access to water.The scorpions were collected with official permit of SEMARNAT (SGPA/DGVS/12063/15 granted to Laura Valdez).

### 5.2. Extraction of Total RNA from Venom Glands, RNA-Seq and Transcriptome Assembly

Five days prior to the RNA extraction procedure, the scorpions were milked by electrostimulation to deprive the glands from any venom and therefore stimulate venom expression. The scorpions were kept unfed until telson disection. The telsons from four specimens (two males and two females) were dissected under RNAse-free conditions and pooled into a single 1.5 mL microtube. Total RNA was purified using the SV Total RNA Isolation System Kit (Promega, Madison, WI, USA). To the dissected telsons the RNA Lysis Buffer was added and the material was manually macerated with a Kontes microtube pellet pestle rod (Daigger Vernon Hills, IL, USA). The sample was diluted with the RNA Dilution Buffer and heated at 70 °C for 3 min. The cellular debris was precipitated by centrifugation and the cleared lysate was mixed with 95% ethanol and centrifuged in one of the spin baskets supplied by the kit. The basket was washed with the RNA Wash Solution, and then treated with the provided DNAse reaction mix for 15 min. After stopping the reaction, the basket was washed twice with the RNA Wash Solution and the total RNA was eluted in Nuclease-Free Water. The RNA was quantified with a Nanodrop 1000 (Thermo Fisher Scientific, Waltham, MA, USA) and its integrity was confirmed using a 2100 Bioanalyzer (Agilent Technologies, Santa Clara, CA, USA).

A cDNA library was constructed from the obtained total RNA, using the Illumina TruSeq Stranded mRNA Sample Preparation Kit, following the protocol supplied by the provider. Automated DNA sequencing was performed at the Massive DNA Sequencing and Bioinformatics Unit in the Institute of Biotechnology (Cuernavaca, Mexico). The 200–400 bp cDNA fragments from the library were sequenced in a Genome Analyzer IIx (Illumina), with the 72-bp paired-end sequencing protocol. After adaptor clipping, the quality of the raw reads was assessed with the FastQC program (http://www.bioinformatics.bbsrc.ac.uk/projects/fastqc/).

The reads were de novo assembled into contigs with the Trinity software (v. 2.0.3, Arlington, TX, USA, 2015), using the standard protocol [67], executing the strand-specific parameter and normalizing the reads. A modification was introduced for the minimum assembled contig length to report (min_contig_length) parameter. The default value of 200 was changed to 100 in order to maximize the recovery of short trasncripts coding for small venom peptides (e.g., antimicrobials). Basic statistics for the assembly, including the total number of Trinity ‘genes’ and ‘transcripts’, contiguity, and contig lengths were obtained with the TrinityStats.pl script. The automatic functional annotation of the transcriptome was performed with Trinotate (https://trinotate.github.io/, Grabherr et al., 2011), which was then manually curated using the Blast suite for sequence similarity searching (https://blast.ncbi.nlm.nih.gov/Blast.cgi, with an e-value cut-off of 1E-4), the Pfam database for protein domain identification (http://pfam.xfam.org), the The Gene Ontology (GO) server for putative function and cellular process assignment (http://www.geneontology.org), and the SignalP (http://www.cdbs.dtu.dk/services/SignalP/) and ProP (http://www.cbs.dtu.dk/services/ProP/) servers for signal peptide and propeptide sequence delimitation, respectively. The theoretical molecular weight of the predicted mature peptides was determined with the ProtParam tool in the ExPASy portal (http://web.expasy.org/protparam).

The subgroup of transcripts potentially coding for venom peptides contains the sequences that either (1) showed similarity to the previously reported EST for this scorpion [12], (2) had one of the ca. 22 distinctive domains associated with scorpion, spider, snake, insect and conus venoms, and/or (3) had sequence similarity with any of the over 6000 sequences identified in the UniProt’s Animal Toxin Annotation Project (http://www.uniprot.org/biocuration_project/Toxins/statistics) as proteins found in venoms.

All alignments were performed with Clustal Omega (http://www.ebi.ac.uk/Tools/msa/clustalo/) with the default parameters).

### 5.3. Mass Spectrometry Analysis

All mass spectrometry determinations were performed into a LC-MS system composed of a nano-flow pump Dionex UltiMate 3000 and an Orbitrap Velos mass spectrometer with a nano-spray ion source both from Thermo-Fisher Scientific (San Jose, CA, USA).

#### 5.3.1. Nanoscale Liquid Chromatography-Mass Spectrometry

Molecular mass fingerprinting analysis of the venom components were obtained by applying 4 μL of 1 μg/μL protein content of whole soluble venom dissolved in 0.1% formic acid solution (solvent A) to Orbitrap Velos mass spectrometer (San Jose, CA, USA). Sample was eluted using a RP C-18 capillary column constructed in house (30 cm length; 0.5 mm ID), which was filled with a C-18 Jupiter silica with 4 μm particle size from Phenomenex (Torrance, CA, USA) by applying a gradient system from 5% to 80% solvent B (0.1% formic acid in acetonitrile) with a flowrate of 300 nL/min for 180 min. Full scan spectra were acquired in positive ion mode using an ionization voltage of 3.1 kV at 60,000 resolution power.

#### 5.3.2. Molecular Mass Fingerprinting

Molecular mass were obtained as [M] by automatic deconvolution (Xcalibur version 2.2, Waltham, MA, USA, 2011) averaging the full scan spectra each 20 min. The screening raw data were filtered using an in house-produced (Microsoft Excel) calculator to eliminate common adducts, amino acid oxidations, dehydrations, deaminations and neutral losses of carbon monoxide. The values used in the calculator were taken from the Mass Spectrometry Adduct Calculator from Fiehn’s laboratory at UCDavis. For peptides with molecular weights less than 3000 Da the monoisotopic masses were used, whereas for peptides with higher molecular weights the average molecular mass was chosen. 

#### 5.3.3. Liquid Chromatography-Tandem Mass Spectrometry (LC-MS/MS)

A bottom-up proteomics approach was used to identify proteins present in the scorpion total venom. Reduction of cysteine residues was performed with addition of 10 mM dithiothreitol (DTT) at 56 °C for 30 min and then alkylated with 50 mM iodoacetamide under light protection for 30 min. After chemical modification of the cysteines and desalting the solution, 50 μg of total proteins were solubilized in 40 mM ammonium bicarbonate and enzymatically digested in (1:25) trypsin solution (Promega, Madison, WI). Digestion was carried out by incubation overnight at 37 °C, pH 8.1. The solution containing the reduced and alkylated tryptic peptides was desalted with ZipTip C-18 under saturation conditions and 5 μg of the tryptic peptides solution was applied into the LC-MS system. Sample was fractionated on a RP C-18 capillary column constructed *in house* (30 cm length; 0.5 mm ID), which was filled with a C-18 Jupiter silica with 4 μm particle size from Phenomenex (Torrance, CA, USA). Gradient elution was performed from 5% to 80% solvent B during 120 min, maintaining a flowrate of 400 nL/min. All spectra were collected in a positive and data dependent acquisition modes set to detect precursor ions from 300 to 1800 *m*/*z* of multi-charged ions from *z* 2^+^ to *z* 5^+^ using an Orbitrap Velos mass spectrometer (Thermo Fisher Scientific, San Jose, CA, USA) with dynamic exclusion set to maximum 120 ions, 30 s for pre-exclusion and 90 min for exclusion. Window length of 0.75 Da was set to include isotopes for MS/MS. The other acquisition parameters were 60,000 for resolution power, 3.0 Da of isolation width, 35 arbitrary units of normalized collision energy, 0.250 of Q-activation and 10 ms of activation time. CID (Collision Induced Activation) and HCD (High-energy Collision Activation) were used alternately and the spectra of both were integrated for data analysis and visualization.

#### 5.3.4. MS Data Analysis

All MS raw files generated were searched against predicted coding sequences (CDSs) from the assembled transcriptome using the Protein Discoverer program. SEQUEST algorithm (Thermo Fisher Scientific, San Jose, CA, USA) was used as engine search according to the following parameters: for MS/MS events precursor and fragment ions tolerance were set to 20 ppm (parts per million) and 0.6 Da, respectively; carbamidomethyl cysteine was set as fixed modification, whereas the oxidation methionine and amidation were set as variable modifications. For decoy data base search False Discovery Rate (FDR) targets were set in 0.01 and 0.05 for strict and relaxed, respectively. Two miss cleavages were allowed per peptide and only identification scores higher than 25, and at least two fragments were considered as positive hits. For de novo protein identification, the software Peaks Studio v8.5 (Bioinformatics Solution Inc., Waterloo, ON, Canada) was used. In this case we have used the same parameters as SEQUEST algoritm (including fixed and variable modifications and fragment ion tolerance). A cutoff of 80 was taken for considering a good de novo sequence (measured as Average Local Confidence (ALC).

## Figures and Tables

**Table 1:** The nomenclature used for the *T. atrox* transcripts.

Species Code	Meaning	Family Code	Meaning	Subtype Code	Meaning	Example
Tat	*T. atrox*	NaT	Na-channel	Alp	Alpha-Na Toxin	TatNaTAlp01
			Toxins	Bet	Beta-Na Toxin	TatNaTBet01
		KTx	K-channel Toxins	Alp	Alpha-K Toxin	TatKTxAlp01
				Bet	Beta-K Toxin	TatKTxBet01
				Kap	Kappa-K Toxin	TatKTxKap01
				Del	Delta-K Toxin	TatKTxDel01
				Scr	Scorpin-like	TatKTxScr01
		CaT	Ca-channel Toxins	Clc	Calcin	TatCaTClc01
				Lio	Liotoxin-like	TatCaTLio01
				Ome	Omegascorpin	TatCaTOme01
		HDP	Host Defense Peptides	Def	Defensin	TatHDPDef01
				ND1–5	NDBPs families 1–5	TatHDPND201
				Ani	Anionic peptide	TatHDPAni01
				Wap	Waprin-like	TatHDPWap01
		Enz	Enzymes	PA2	Phospholipase A2	TatEnzPA201
				PLB	Phospholipase B	TatEnzPLB01
				PLD	Phospholipase D	TatEnzPLD01
				SeP	Serine protease	TatEnzSeP01
				MtP	Metalloprotease	TatEnzMtP01
				Hya	Hyaluronidase	TatEnzHya01
		Pin	Protease	Srp	Serpin-like	TatPInSrp01
			Inhibitors	Kun	Kunitz-type	TatPInKun01
		Oth	Other	La1	La1-like	TatOthLa101
			venom	CRI	CRISP	TatOthCRI01
			components	Und	Undefined	TatOthUnd01

**Table 2:** Physicochemical parameters predicted for the mature NDBPs by the HeliQuest software (http://heliquest.ipmc.cnrs.fr/cgi-bin/ComputParams.py).

ID	NDBp Family	Length of the Mature Peptide	Hydrophobicity	Hydrophobic Moment	Charge
TatHDPND202	NDBP-2	47	0.263	0.065	+6
TatHDPND201	NDBP-2	43	0.212	0.089	+6
TatHDPND301	NDBP-3	19	0.903	0.377	+2
TatHDPND302	NDBP-3	25	0.495	0.327	+4
TatHDPND405	NDBP-4	13	0.819	0.606	+1
TatHDPND406	NDBP-4	13	0.742	0.458	+1
TatHDPND407	NDBP-4	13	0.746	0.456	+1
TatHDPND402	NDBP-4	13	0.778	0.779	0
TatHDPND403	NDBP-4	13	0.752	0.792	+1
TatHDPND401	NDBP-4	13	0.793	0.595	+1

**Table 3:** Mass fingerprint from the fractions of the *T. atrox* soluble venom. The distribution of venom components found with LC-MS were reported in 20 min intervals. Monoisotopic mass was considered for those components with a MW below 3000 Da and for components with MW above 3000 Da, average mass was considered.

RT ^1^ (min)	MW ^2^ (Da)	RT (min)	MW (Da)
**1–20**	1462.7, 2057.24, 2117.68, 2265.06, 2796.27, 3111.96, 9115.86, 10663.93, 11,123.28,	**140–160**	1944.15, 2645.50, 2815.60, 6330.03, 6473.90, 6714.40, 7438.62, 7639.27, 7843.00, 8049.12, 8213.16, 8829.81, 8950.11,9535.2
**20–40**	1076.62, 1205.68, 1212.80, 1673.85, 1817.88, 3427.38, 3499.92, 3586.92, 3878.10, 4197.53, 12,306.36	**160–180**	1337.72, 1497.81, 2193.06, 2248.28, 2347.32, 3338.30, 7040.46, 7956.10, 8201.97, 8727.14
**40–60**	1331.64, 1799.04, 1886.82, 2333.32, 2411.36, 2447.40, 2592.26, 3777.63, 3945.62, 5813.52	**180–200**	1296.10, 2151.20, 4171.38, 4302.42, 4389.42, 4697.56, 4762.08, 6195.66
**60–80**	2377.16, 2850.1, 2944.70, 3606.60, 4485.10, 4595.04, 5279.52, 5654.40	**200–220**	10,039.5, 13,729.41, 14,079.03
**80–100**	3332.90, 3535.47, 3718.60, 3787.85, 4113.96, 4125.80, 4204.00, 4279.05, 4290.36, 5196.42, 5756.56, 7011.33, 7123.96, 7236.99, 8126.40, 8328.51	**220–240**	1828.00, 6554.31, 6750.45, 6946.57, 7269.84, 8272.50, 10,545.20, 12,430.9, 13,591.92, 13,815.51, 14,614.72
**100–120**	3223.80, 3243.80, 3569.92, 3767.15, 4250.67, 8468.54, 8581.60, 8716.70, 9056.88, 9490.25	**240–260**	3821.44, 5409.48, 10,882.9, 16,915.41
**120–140**	1198.64, 1648.86, 3267.39, 4036.16, 4348.40, 4561.84, 4815.2	**260–290**	2038.11, 3347.5, 4505.55, 4791.65, 4949.7, 8355.48, 11,174.46, 11,833.92, 11,847.44, 11,899.27, 13,891.59, 14,257.71, 14,705.56, 14,741.70

^1^ RT (retention time); ^2^ MW (experimental molecular weight in Daltons).

**Table 4:** Molecular masses identified in *T. atrox* transcriptome.

***Sodium toxins***			
**Transcriptome ID**	**Theoretical Mass**	**Experimental Mass**	**RT Range**
**TatNaTBet03**	5196.79	5196.42	80–100
**TatNaTBet08**	6195.85	6195.66	180–200
***Potassium toxins***			
**TatKTxAlp10**	3607.43	3606.60	60–80
**TatKTxAlp12**	4114.86	4113.96	100–120
***Calcium toxins***			
**TatCaTClc01**	3788.48	3787.85	80–100

**Table 5:** Amino acid sequences found by LC-MS/MS using the transcriptome of *T. atrox* as a database for protein identification.

Transcriptome ID	Score	Coverage	Protein Type	Accession Number of the Reference Protein
comp8310_c0_seq1	46.06	19.1%	Allatostatins-like	XP_013775495
comp32030_c1_seq1	28.07	34.8%	Angiotensin-converting enzyme	XP_013773749
comp32030_c2_seq1	32.73	7.7%	Angiotensin-converting enzyme	XP_013773749
comp33161_c0_seq1	535.88	24.5%	Angiotensin-converting enzyme	XP_013773749
comp33725_c0_seq1	65.33	16.8%	Angiotensin-converting enzyme	XP_013773749
comp33936_c0_seq1	64.74	13.1%	Angiotensin-converting enzyme	XP_013773749
TatCaTClc01	88.83	24.2%	Calcium toxin. Calcin	A0A1L4BJ42
comp32319_c0_seq1	18.83	7.56%	Ectonucleoside triphosphate diphosphohydrolase 2-like	XP_013778001
comp881_c0_seq1	452.55	18.7%	Elastase-like protein	CAX51421
TatHDPND201	513.12	46.7%	HDP. NDBP-2 family	F1AWB0
TatHDPND301	22.46	94.7%	HDP. NDBP-3 family	ALG64974
ViVlp1	762.84	28.6%	HDP. NDBP-2 family	AGK88593
ViAMP1	188.70	70.8%	HDP. NDBP-3 family	ALG64975
TaHDPND401	254.77	61.5%	HDP. NDBP-4 family	I0DEB5
TatHDPND403	37.65	100%	HDP. NDBP-4 family	I0DEB5
ViCT2	882.53	76.9%	HDP. NDBP-4 family	I0DEB3
TatEnzHya01	161.40	39.1%	Hyaluronidase	API81375
comp15335_c0_seq1	92.74	10.3%	Hypothetical protein	CAX51393
comp30560_c0_seq1	67.45	9.1%	Hypothetical protein	AEX09195
comp31101_c0_seq1	103.49	29.1%	Hypothetical protein (allergen type)	CAX51409
comp30730_c0_seq1	16.64	6.5%	Hypothetical protein RvY_03950	GAU91754
ViLa1lp1	47.91	63.3%	La1-like	AOF40216
TatOthLa101	469.44	45.5%	La1-like	AOF40202
comp34524_c0_seq1	74.23	11.5%	Metalloproteinase	XP_009865190
TatEnzMtp04	31.25	18.8%	Metalloproteinase	AMO02513
comp32637_c0_seq1	828.12	42%	Nucleotidase	XP_013774694
comp26928_c1_seq1	214.51	23.2%	Other venom components	N/A
comp27809_c1_seq1	1255.98	24.4%	Other venom components	N/A
comp30392_c0_seq1	34.49	32.9%	Other venom components	CAX51433
comp32982_c0_seq3	20.83	13.7%	Other venom components	N/A
comp43100_c0_seq1	70.29	15.3%	Other venom components	N/A
comp31198_c0_seq1	20.19	3.13%	Other venom components	N/A
TatEnzPA201	1616.89	45.5%	Phospholipase A2	API81339
TatEnzPA213	253.38	27%	Phospholipase A2	API81335
TatEnzPA215	877.33	50.2%	Phospholipase A2	API81335
TatEnzPA202	94.96	31.3%	Phospholipase A2	API81335
comp20627_c0_seq1	14.64	0.9%	Protein kinase C-binding protein NELL2-like	XP_022243213
TatOthCRI06	24.43	15.9%	Putative cysteine-rich protein	JAT91149
TatOthCRI07	10.69	23.1%	Putative cysteine-rich protein	API81352
comp30427_c0_seq1	16.87	2.27%	Steryl-sulfatase-like isoform	XP_0193859

## Supplementary Materials

The following are available online at www.mdpi.com/2072-6651/9/12/399/s1, Figure S1: Distribution of the GO terms for the annotated *T. atrox* transcripts, Figure S2: Sequence coverage and MS/MS spectrum of TatHDPND403, Table S1: Distribution of transcripts that putatively code for venom components, Table S2: Sequences identified with the Proteome Discoverer software, Table S3: Sequences identified *de novo* in the proteome of *T. atrox* using Peaks Studio software.
