# Supplementary material for: A Deeper Examination of Thorellius atrox Scorpion Venom Components with Omic Techonologies
Source: Toxins (Basel). 2017 Dec 12;9(12):399. doi: 10.3390/toxins9120399 (PMC5744119; doi:10.3390/toxins9120399)
Supplement: Supplementary file 1 [file toxins-09-00399-s001.zip › supplementary/Figures S1 and S2 updated.pdf]

# Supplementary Materials: A deeper examination of Thorellius atrox scorpion venom components with omic techonologies

**Teresa Romero-Gutierrez, Esteban Peguero-Sanchez, Miguel A. Cevallos, Cesar V. F. Batista, Ernesto Ortiz and Lourival D. Possani**

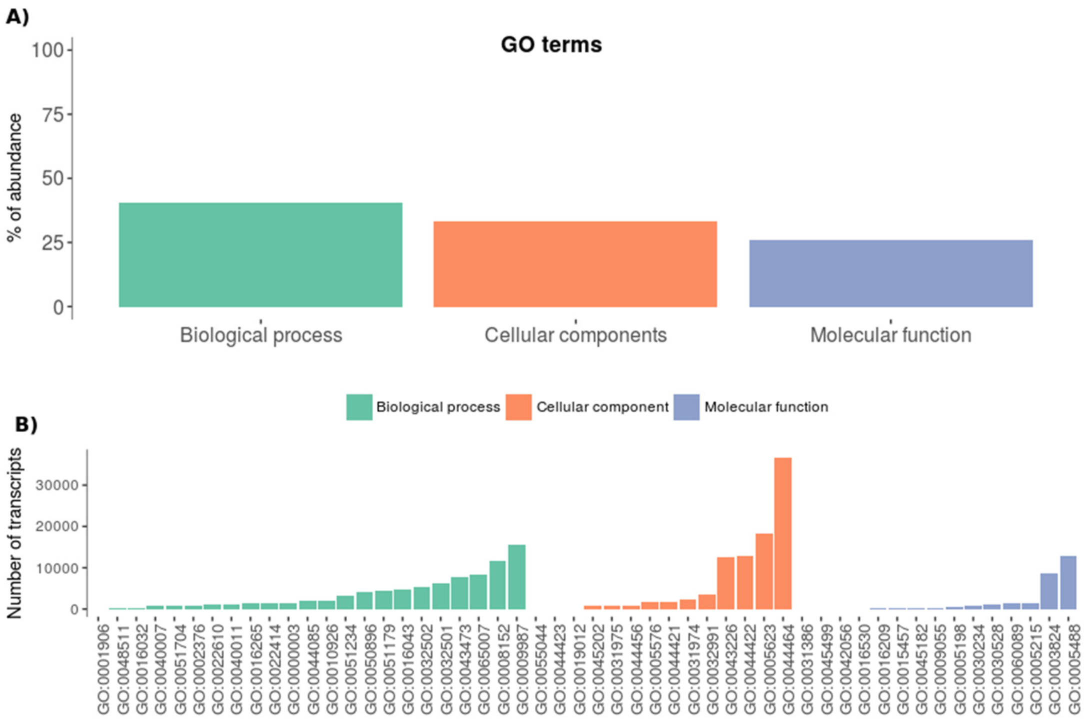


**Figure S1.** Distribution of the GO terms for the annotated *T. atrox* transcripts. (**A**) Distribution in accordance to the main three Gene Ontology (GO) 'root' nodes: Cellular Component, Molecular Function or Biological Process. (**B**) Assignment of the transcripts to the first level of 'child' terms of the GO hierarchy. The IDs of the GO terms are shown in the x-axis.

1 FWNTLLSVGKSLL 13

Sequence: FWNTLLSVGK, Charge: +2, Monoisotopic m/z: 582.8 Da (+0.76 mmu/+1.31 ppm), MH+: 1164.6 Da, RT: 91.93 min, Identified with: Sequest HT (v1.3); XCorr:3.18, Fragment match tolerance used for search: 0.6 Da

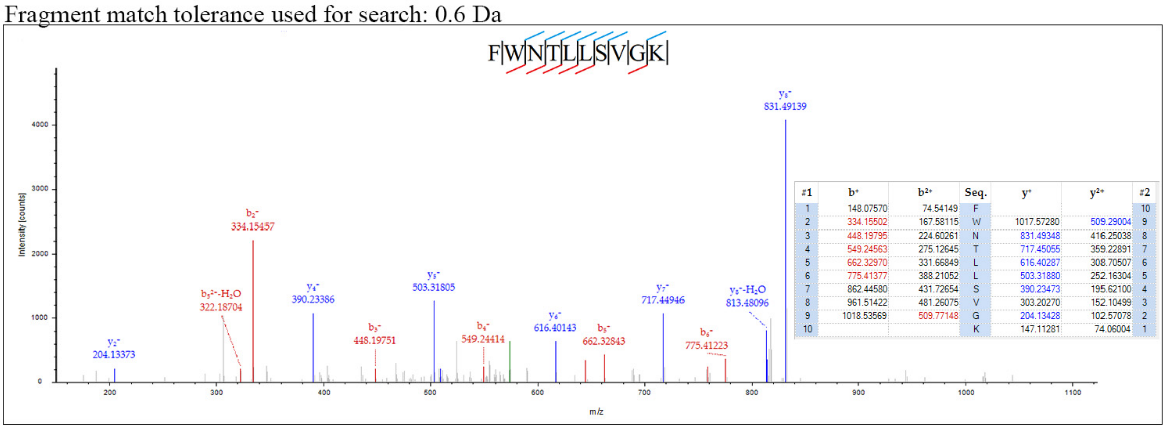


| #1 | $b^+$ | $b^{2+}$ | Seq. | $y^+$ | $y^{2+}$ | #2 |
|---|---|---|---|---|---|---|
| 1 | 148.07570 | 74.54149 | F | | | 10 |
| 2 | 334.15502 | 167.58115 | W | 1017.57280 | 509.29004 | 9 |
| 3 | 448.19795 | 224.60261 | N | 831.49348 | 416.25038 | 8 |
| 4 | 549.24563 | 275.12645 | T | 717.45055 | 359.22891 | 7 |
| 5 | 662.32970 | 331.66849 | L | 616.40287 | 308.70507 | 6 |
| 6 | 775.41377 | 388.21052 | L | 503.31880 | 252.16304 | 5 |
| 7 | 862.44580 | 431.72654 | S | 390.23473 | 195.62100 | 4 |
| 8 | 961.51422 | 481.26075 | V | 303.20270 | 152.10499 | 3 |
| 9 | 1018.53569 | 509.77148 | G | 204.13428 | 102.57078 | 2 |
| 10 | | | K | 147.11281 | 74.06004 | 1 |

**Figure S2.** Sequence coverage and MS/MS spectrum of TatHDPND403
