# Supplementary material for: A Deeper Examination of Thorellius atrox Scorpion Venom Components with Omic Techonologies
Source: Toxins (Basel). 2017 Dec 12;9(12):399. doi: 10.3390/toxins9120399 (PMC5744119; doi:10.3390/toxins9120399)
Supplement: Supplementary file 1 [file toxins-09-00399-s001.zip › supplementary/TableS1.pdf]

Table S1. Distribution of transcripts that putatively code for venom components.

Sodium toxins							
**ID**	**ID/Reference protein**	**e-value**	**PFAM domain**	**MW (Da)**	**Sub-classification**	**Amino acid sequence**	**Nucleotide sequence**
TatNaTAlp01	P63019.1	2 e-28	PF00537	--	alpha toxin	ILLLGIERSES**RDGYPIASNGCKFGCSGLGENNPTCNHVCEKKAGSDYGYCYAW**	ATTCTACTCTTAGGAATAGAAAGAAGCGAGTCGAGAGATGGTTATCCTATAGCTTCAAATGGTTGCAAATTTGGATGCAGTGGATTAGGTGAGAACAATCCAACTTGTAATCACGTTTGTGAAAAGAAAGCCGGAAGTGACTACGGCTATTGCTATGCCTGG
TatNaTAlp02	Q5MJP5.1	6 e-24	N/D	8500.77	alpha toxin	MNTLSTFTLTSLLLLIIGFYQYQAQA**RFVRRKNMTFYECSELSGHDPYCVRTCEEHESKEEGYCKEFDRYGISACYCPNIVGTKVVMCYAALQRKCKF***	ATGAATACACTAAGCACCTTTACCCTCACTTCATTACTTCTTCTTATTATTGGCTTTTATCAATATCAAGCACAGGCAAGATTTGTGAGAAGGAAAAACATGACATTTTACGAATGCAGCGAATTATCTGGCCATGATCCCTATTGTGTTCGTACTTGCGAAGAGCACGAATCAAAAGAGGAGGGATATTGTAAAGAATTTGACCGTTATGGAATTTCTGCTTGTTACTGCCCAAACATTGTAGGAACCAAAGTTGTTATGTGCTACGCAGCTCTTCAAAGAAAATGCAAATTC
TatNaTAlp03	Q5MJP5.1	5 e-34	PF00537	7986.39	alpha toxin	MKTIANMTYYFYCLLLFVGIFNIQVGGK**FVRKPNGSFYECGMLIGYQPYCVQTCKEHKAPEKGYCKEMYPFGLPGGCYCPNIPQNKIRMCRGALKNEC***	ATGACTTATTATTTCTACTGTCTCTTGCTTTTCGTAGGCATTTTTAATATTCAAGTAGGAGGAAAATTTGTGAGAAAACCCAATGGCTCATTTTATGAATGCGGTATGTTAATAGGGTACCAGCCATATTGCGTTCAGACTTGTAAAGAACACAAAGCTCCTGAAAAAGGCTACTGCAAAGAAATGTATCCTTTTGGACTCCCTGGTGGCTGCTACTGTCCAAATATTCCTCAAAATAAAATAAGAATGTGTCGCGGTGCCCTTAAAAATGAGTGT
TatNaTBet01	P0CI45.1	9 e-17	PF00537	6672	beta toxin	MNCSTVLIFCLIAIVFEFAGVEC**KKDGYPLTTEGLKFSCAAGMLGDNRFCLSICRSRGSNYGYCYFFGCYCEGLNDDVKIWSG***	ATGAATTGCTCTACTGTTCTCATTTTCTGCCTAATTGCCATTGTTTTTGAATTTGCTGGTGTGGAGTGCAAAAAGGATGGATATCCTTTGACTACAGAAGGATTGAAGTTCAGTTGTGCTGCAGGAATGTTAGGTGATAACAGATTCTGTCTATCTATCTGTAGAAGTCGTGGTTCAAATTATGGTTATTGTTACTTCTTTGGTTGTTATTGTGAAGGCTTAAATGATGATGTTAAAATTTGGAGTGGCTAA
TatNaTBet02	P01491.3	3 e-16	PF00537	6544.9	beta toxin	MNCSTVLIFCLIAIVFEFAGVEC**KKDGYPVTKEGLKVSCAAGMILGDNRFCQAICKGDGADYGYCYGFGCYCEGLNDDVKIWSG***	ATGAATTGCTCTACTGTTCTCATTTTCTGCCTAATTGCCATTGTTTTTGAATTTGCTGGTGTGGAGTGCAAAAAGGATGGATATCCTGTGACTAAGGAAGGATTGAAGGTCAGTTGTGCTGCAGGAATGATATTAGGCGATAACAGATTCTGTCAAGCCATCTGTAAAGGCGATGGTGCCGATTATGGTTATTGTTACGGGTTTGGTTGTTACTGTGAAGGCTTAAATGATGATGTTAAAATTTGGAGTGGCTAA
TatNaTBet03	D9U299.1	4 e-12	PF00537	5193.2	beta toxin	MHISMAAILPLLCLIVTLVFVAETAYS**GFFPLDENGKVYRCEDLGPNQLCNATCVFHGGTYGYCAVSSCYCENR***	ATGCATATCAGCATGGCAGCCATTTTGCCTCTTCTCTGTCTGATCGTTACTTTAGTCTTTGTTGCTGAAACTGCATACAGCGGATTTTTTCCGCTGGACGAGAATGGGAAAGTGTACCGCTGCGAAGATTTGGGGCCTAATCAGCTGTGCAATGCTACCTGTGTCTTCCATGGTGGAACTTATGGCTACTGTGCCGTCTCCAGCTGTTACTGCGAAAATCGATAA
TatNaTBet04	Q7YT61.1	2 e-27	N/D	--	beta toxin	MNSLLIITACLVLFGTVWA**KEGYLVNKSTGCKYGCFWLGKNENC**	ATGAACTCTTTGTTGATAATCACTGCTTGTTTGGTCCTATTCGGAACAGTGTGGGCAAAAGAAGGTTATCTGGTAAACAAGAGCACGGGCTGCAAATACGGTTGCTTCTGGTTGGGAAAAAACGAAAACTGC
TatNaTBet05	ACJ23108.1	2 e-10	N/D	--	beta toxin	IMRVIVFTATVFALSLLGGYSA**KNAYPRNYEGNCYRCRYPDKDDFCNDICKSLHKANAGECSVSNLFCFCVGIKEENISIKDVGKA**	ATCATGAGGGTTATTGTCTTTACTGCCACGGTATTTGCTCTTTCTCTGTTGGGCGGATACAGTGCCAAAAATGCGTATCCACGCAACTACGAAGGGAACTGCTATCGATGTCGTTATCCCGATAAAGATGATTTTTGCAATGACATCTGTAAAAGTTTGCACAAAGCAAACGCTGGTGAATGCAGCGTATCTAATTTATTCTGTTTCTGCGTTGGCATCAAAGAGGAAAATATTAGCATAAAAGACGTTGGAAAAGCA
TatNaTBet06	P0C1B8.1	4 e-11	PF00537	--	beta toxin	MAVILSIFCFVVALTVIDS**ENTYNGFFPLDENGEVYRCEFLGYDQLCNATCAFHGGIYGYCAISSCYCERGRNSTAAT**	ATGGCAGTCATCTTGTCTATTTTCTGCTTTGTCGTTGCTTTAACCGTTATTGATAGTGAAAATACGTATAATGGATTTTTTCCCTTGGATGAGAACGGGGAAGTATACCGCTGCGAATTCTTGGGATATGATCAGCTATGCAATGCTACTTGTGCCTTCCATGGTGGGATTTATGGATATTGTGCCATCTCCAGTTGTTATTGCGAAAGAGGTAGAAATTCAACGGCAGCTACA

TatNaTBet07	P59865.1	3 e-9	N/D	--	beta toxin	MIFAAYWITLFLFYVLFVPVAT**TPSPTSDDVKRVYIPGYADKTCWKLGSNSFCRDICQETAGHGYCHLFVCYCESIS**	ATGATATTTGCTGCTTATTGGATAACGCTGTTCCTATTTTACGTTCTTTTTGTTCCAGTAGCTACCACTCCATCACCAACGTCTGATGATGTTAAAAGAGTTTACATTCCTGGCTATGCTGACAAAACTTGCTGGAAATTAGGATCGAATTCCTTCTGCAGAGATATTTGCCAGGAAACAGCAGGACATGGATATTGTCATCTTTTCGTTTGCTATTGTGAAAGCATTTCA
TatNaTBet08	P15223.3	6 e-9	PF00537	6191.6	beta toxin	MKCFAFLTVCLAVVLFEFTEVEG**KDGYPVDANGNVFNCVPGKLEVCKSACGERGSDQISCLAFTCWCDGMNDDAETWKHD***	ATGAAGTGTTTTGCCTTTCTCACAGTTTGCCTGGCTGTCGTCCTTTTTGAATTTACTGAGGTGGAAGGAAAGGATGGATATCCAGTGGATGCGAATGGAAATGTTTTCAACTGCGTTCCAGGGAAGTTAGAAGTTTGTAAATCTGCGTGTGGTGAGCGTGGATCTGATCAAATTTCCTGCCTCGCATTCACTTGCTGGTGTGATGGGATGAACGATGATGCCGAAACATGGAAACACGAC
TatNaTBet09	Q4LCT0.1	6 e-9	N/D	5178.1	beta toxin	MQDADNNKILQQKQGIFITGMEG**CDFVGGKGVYLDVGCTELGKNSLCEEKCRQVCNSYGYCYSFECWCE***	ATGCAAGATGCCGACAATAATAAAATATTGCAGCAAAAACAAGGAATTTTCATAACTGGAATGGAAGGCTGTGATTTTGTTGGTGGAAAAGGAGTGTATCTCGATGTTGGCTGTACAGAACTGGGCAAAAATTCGTTGTGTGAAGAGAAGTGCCGACAAGTTTGCAATTCCTATGGTTATTGCTACAGTTTCGAATGTTGGTGTGAA
TatNaTBet10	Q4LCT0.1	7 e-11	N/D	5392.13	beta toxin	MQLRLFSILIVFGMFKTSMEG**CAVSGGIGTTYINDLCSNLGKNDFCKEKCREICYSYGYCYFFQCWCE***	ATGCAGCTGCGATTGTTTTCGATCTTGATTGTATTTGGAATGTTCAAAACCAGTATGGAAGGCTGTGCTGTTTCTGGTGGAATAGGAACCACCTACATCAATGACCTCTGTTCGAATCTCGGCAAAAATGATTTTTGTAAAGAGAAGTGCAGAGAAATTTGTTATTCTTACGGTTATTGCTACTTCTTCCAATGCTGGTGCGAA
						**Potassium toxins**	
**ID**	**ID/Reference protein**	**e-value**	**PFAM domain**	**MW (Da)**	**Sub-classification**	**Amino acid sequence**	**Nucleotide sequence**
TatKTxAlp01	API81322.1	1 e-22	PF14866	4563.34	alpha toxin	MNKALFATVLIVLMVFAVSG**IPAKNYAPTGGCPLSDALCANYCKKNKFGNGGKCDGTKCKCSIK***	ATGAATAAGGCTTTATTTGCAACTGTTCTAATTGTTTTGATGGTGTTCGCCGTTAGTGGTATTCCAGCAAAAAACTATGCACCAACTGGAGGCTGCCCATTGTCAGACGCCCTTTGTGCTAATTACTGCAAGAAAAACAAATTTGGAAATGGTGGGAAATGCGATGGAACTAAATGCAAATGTTCTATTAAG
TatKTxAlp02	P45696.1	1 e-9	PF00451	--	alpha toxin	MRLAIILLLMTTLVLTIG**APPNGAKCSSSVQCTRPCRHGGGTHGKCMNGR**	ATGAGGCTAGCAATTATTTTGCTGTTGATGACAACATTGGTGTTGACAATTGGTGCACCTCCCAATGGTGCAAAATGTTCATCATCTGTCCAGTGCACACGTCCTTGCCGACATGGAGGAGGAACACATGGAAAATGCATGAACGGAAGA
TatKTxAlp03	API81318.1	3 e-13	PF00451	3933.78	alpha toxin	MIGSFILTFDVVTTEG**GKACTRTKECWSYCKKKDGCPKGKCINKICHCYGC***	ATGATCGGCTCTTTTATATTAACGTTTGATGTAGTGACAACTGAAGGAGGAAAGGCCTGCACTAGAACTAAAGAATGTTGGTCCTATTGTAAAAAGAAGGACGGATGTCCTAAAGGAAAATGTATAAACAAAATATGCCATTGCTATGGATGT
TatKTxAlp04	API81318.1	4 e-13	PF00451	4491.24	alpha toxin	MRTVCKKMNTKFAIFLIFTIVILLEGATT**LEKGAKCETSKYCSDYCDRINKCPRGKCISRECKCYVCGG***	ATGAGAACCGTGTGCAAAAAGATGAATACAAAATTTGCTATCTTCCTTATTTTTACCATTGTGATACTGCTTGAAGGTGCAACGACACTCGAAAAAGGAGCCAAATGCGAAACGAGCAAATACTGTTCGGATTACTGTGATAGGATCAACAAATGTCCACGTGGAAAATGCATAAGCAGGGAATGCAAGTGCTATGTTTGTGGAGGC
TatKTxAlp05	API81318.1	3 e-12	PF00451	4606.35	alpha toxin	MVLKFAFILLMFTATILTIGAMA**LEKGAECQTTRYCWDYCGMINNCPRGKCINWECKCYICGG***	ATGGTTCTAAAATTTGCTTTCATTCTCCTTATGTTTACCGCTACGATATTGACAATTGGTGCAATGGCACTCGAAAAAGGAGCCGAATGCCAAACGACAAGATACTGTTGGGATTATTGTGGTATGATCAACAACTGTCCACGTGGAAAATGTATAAACTGGGAATGCAAATGCTACATTTGTGGGGGA
TatKTxAlp06	API81318.1	2 e-21	PF00451	4004.59	alpha toxin	MGTKFVFITLLATTVILAFGALTVEG**GKNCSGSKECYDYCYKTTGCSRAKCINRACKCYGCSG***	ATGGGTACAAAATTCGTCTTCATTACACTCTTGGCCACCACTGTGATATTAGCGTTTGGTGCACTGACAGTTGAAGGAGGGAAGAATTGCAGCGGATCTAAAGAATGTTATGATTATTGTTATAAGACAACCGGATGTTCTCGTGCAAAATGCATCAATAGGGCATGTAAATGTTACGGTTGTAGTGGA
TatKTxAlp07	AHJ59318.1	2 e-16	PF00451	4656	alpha toxin	MSTKFFVLMLMVSTVILT**FDVMTTEGGKTCTGSNQCYPYCRKQTGYNAGKCINGRCKCYGG***	ATGAGCACGAAATTTTTCGTCCTTATGCTTATGGTCAGCACTGTTATACTGACATTTGATGTAATGACAACTGAAGGAGGAAAGACCTGCACTGGAAGTAACCAATGTTATCCTTATTGTAGGAAGCAGACTGGATATAATGCTGGAAAATGTATAAACGGAAGATGCAAATGCTATGGAGGT

TatKTxAlp08	AFB73769.1	7 e-13	PF00451	3533.5	alpha toxin	MSTKFFILMLMVGSVILTFDVMTTEG**GKTCTGSHQCRPYCIEQTGYNNGKCMNGRCKC***	ATGAGCACAAAATTTTTCATCCTTATGCTTATGGTCGGCTCTGTTATACTGACATTTGATGTAATGACAACTGAAGGAGGAAAGACCTGCACTGGAAGTCACCAATGTCGGCCCTATTGTATAGAGCAGACTGGATATAATAATGGAAAATGTATGAACGGAAGATGCAAATGCTAG
TatKTxAlp09	AHJ59318.1	5 e-19	PF00451	4560.25	alpha toxin	KFFILMLVVSSVILTFGVMTTEG**GLPCNGSHQCWDYCRAKTGCFSAKCMNGRCKCYGCSSVNSLQ***	AAATTTTTCATCCTTATGCTTGTGGTCAGCTCTGTTATATTGACCTTTGGTGTAATGACAACTGAAGGAGGACTGCCCTGCAATGGAAGTCACCAATGTTGGGACTATTGTCGAGCTAAGACAGGATGTTTTAGTGCAAAATGTATGAACGGAAGATGCAAATGCTACGGTTGTTCTTCTGTTAATTCTTTACAA
TatKTxAlp10	P0DJ32.1	2 e-16	PF00451	3607.4	alpha toxin	MKTNIVLAFLLLSFLIAVAA*GEGERSTR***AISCVGSKECVPKCRNQGCKNGKCMNKKCKCYC***	ATGAAAACGAATATTGTCCTGGCATTTCTTTTGCTAAGCTTCTTAATAGCCGTTGCAGCTGGTGAAGGTGAGAGAAGCACTCGGGCTATCAGCTGTGTCGGTAGCAAGGAATGTGTTCCAAAGTGTAGAAATCAAGGATGTAAAAATGGAAAATGCATGAATAAGAAGTGCAAATGTTACTGCTAA
TatKTxAlp11	P55928.1	7 e-10	PF00451	--	alpha toxin	ILTFDVMKTEGR**MSCTANRQCWPFCRDHTGYISGKCINRSCKCYRG**	ATACTGACATTTGATGTAATGAAGACTGAAGGAAGAATGTCCTGCACTGCAAATCGCCAATGTTGGCCCTTTTGTAGAGACCATACTGGATATATTAGTGGAAAATGTATAAACAGAAGTTGCAAATGCTACCGAGGTTAA
TatKTxAlp12	API81324.1	5 e-23	N/D	4114.86	alpha toxin	MNKLPCFILISLLFIVITNVAVVDG**ISAGCPLSEKLCTSYCKRGRFGSKGKCVGSIGTDCKCYI***	ATGAACAAATTACCTTGCTTTATTCTGATTTCATTGCTGTTCATCGTTATAACTAACGTTGCAGTCGTGGATGGGATATCAGCTGGCTGCCCGTTGTCTGAAAAACTCTGCACCAGTTATTGCAAAAGAGGACGATTCGGATCAAAGGGGAAGTGTGTTGGAAGCATAGGAACTGACTGCAAATGTTACATT
TatKTxAlp13	ACJ23158.1	7 e-10	N/D	4436.1	alpha toxin	MKLKHLLIFLILCTIASCEDHRYR**RSRGCSRTGACMDYCSIRVHEPQNHRRCPPGEYCCTLVV***	ATGAAGCTTAAACATTTACTGATTTTCTTAATATTATGTACAATTGCTAGCTGTGAAGATCATCGTTACAGACGTTCTAGAGGATGCAGTCGTACTGGAGCCTGTATGGATTACTGTAGTATACGTGTTCATGAGCCACAAAACCACAGAAGATGCCCTCCAGGAGAATACTGTTGTACTCTGGTAGTT
TatKTxAlp14	AOF40181.1	7 e-11	N/D	3644.13	alpha toxin	MKLIILLLLLTLEIATILPTIEG**RKCYDSGWCGGPCRDKGASYGKCIDNQCRCYR***	ATGAAGCTGATTATATTACTGCTTTTGCTTACTCTAGAAATAGCAACGATTCTTCCAACTATCGAAGGCAGAAAGTGCTATGATTCCGGATGGTGCGGAGGACCGTGTCGAGATAAGGGAGCATCTTACGGTAAATGCATAGACAACCAATGTCGGTGCTACAGA
TatKTxAlp15	API81322.1	7 e-31	N/D	3701.21	alpha toxin	MNKAYCAIFLVVLVMFAVSVLPTES**VPTGGCPSDSLCAKYCKSNKYGSTGKCDGTSCKCSLG***	ATGAATAAGGCTTATTGCGCTATATTCCTTGTGGTGCTAGTAATGTTTGCTGTCAGTGTGCTGCCAACTGAGAGCGTACCAACTGGTGGATGCCCAAGTGATTCCCTCTGTGCAAAATACTGCAAAAGTAACAAATATGGTTCTACTGGAAAATGCGATGGCACCAGTTGTAAGTGTTCCCTTGGATGA
TatKTxScr01	P0C8W5.1	4 e-39	PF14866	9051.78	scorpine like	MQKLFAVLCILSLATFCTC**GLIKEKHVQKGVDALTNLIPAPVVGGIINKVAKQMVHKMGKVQELCAFNKDVMGWCDKACLEKEQTNGFCHGTKCKCGKPLSY***	ATGCAGAAACTTTTCGCTGTTTTGTGTATTCTCAGTTTGGCAACTTTCTGCACATGTGGTTTAATCAAAGAGAAACACGTTCAGAAAGGAGTTGATGCTTTGACCAATTTAATACCTGCGCCAGTTGTAGGAGGAATCATAAATAAGGTTGCGAAACAGATGGTGCACAAGATGGGTAAAGTTCAAGAATTATGTGCTTTCAACAAAGATGTGATGGGTTGGTGTGATAAAGCCTGCCTGGAGAAAGAACAGACAAATGGATTTTGTCACGGCACTAAATGCAAGTGCGGGAAACCTTTGAGCTATTAA
ViScplp2	API81325.1	8 e-42	PF14866	8497.85	scorpine like	MNAKITVLFFLVAITIASC**AWISEKKIQDAIDKRLPNGAVKSAIKAVVHKAAKNKHGCLADFDVGGGCEQHCRKTEDKEGVCHGTKCKCGIPRAYRK***	ATGAACGCTAAAATAACTGTCCTATTTTTTCTGGTAGCCATTACAATTGCTTCTTGTGCCTGGATAAGCGAGAAAAAAATCCAAGATGCTATCGATAAGAGGTTGCCAAACGGAGCTGTGAAAAGCGCAATCAAAGCCGTAGTACACAAAGCTGCCAAAAATAAGCATGGCTGTCTAGCTGATTTTGATGTAGGAGGTGGATGCGAACAGCACTGCAGAAAAACTGAAGATAAAGAAGGAGTCTGCCACGGAACCAAGTGCAAATGCGGTATTCCCCGTGCATATAGAAAA
TatKTxScr02	P0C8W5.1	2 e-49	PF14867	9228.06	scorpine like	MQIKFTILILLVISTFCSC**GLIREKYFHQGVDALTPLIPVPVVGGVVNKVAKQMIHKIGKIQSLCAFNKDMAGLCEKKCQETEKVKGYCHGTKCKCGKPLSYK***	ATGCAGATAAAATTTACCATTCTGATTTTACTTGTGATTTCAACATTTTGTTCGTGTGGCTTAATCCGTGAGAAATATTTTCACCAAGGAGTGGACGCTTTAACTCCATTAATACCAGTTCCTGTGGTTGGAGGAGTCGTAAATAAAGTTGCAAAACAGATGATTCACAAAATCGGCAAAATTCAAAGCCTATGTGCTTTCAACAAAGACATGGCTGGTTTATGTGAGAAAAAATGTCAGGAAACAGAAAAGGTGAAAGGATACTGCCACGGTACTAAATGCAAGTGCGGAAAGCCTTTGAGCTACAAA

TatKTxKap01	P0DJ41.1	5 e-24	N/D	3092.71	kappa toxin	MKLLSIVFVLVLVFALLPMEVSC*EPNAIENSRKNYEDISRNIVKR***SCKRVCSGSRRSKQCMQKCKPTPGR**	ATGAAACTGCTATCAATCGTGTTTGTGTTGGTGCTGGTTTTTGCTTTGCTTCCAATGGAAGTTTCCTGTGAACCCAATGCCATAGAGAATAGCAGAAAAAATTATGAAGACATTTCAAGAAATATTGTCAAAAGATCTTGCAAACGAGTATGTTCTGGAAGCAGGCGTTCCAAGCAGTGTATGCAGAAATGTAAACCGACTCCTGGTCGTTGAGAAACAAATTCGGTTCTAAGTTTGTATTCCAAT
TatKTxDel01	P0C8W3.1	1 e-39	PF00014	7457.59	delta toxin kunitz type	MIFAGCLLLILASNALLVTVAG**HHKKQVNCLLPPKAGPCKGSFARYYFDIESGSCKAFIYGGCDANSNNFAKRHHCEKRCRRFSVLGI***	CGCACAAAGAAAAAATTAATTATTACGGAAAAAATGATTTTCGCCGGCTGCCTTTTGTTGATTTTGGCTTCCAATGCCTTGTTAGTGACAGTGGCTGGGCACCACAAGAAACAAGTTAACTGCTTACTTCCACCTAAGGCAGGACCCTGCAAAGGTTCTTTTGCAAGATATTACTTTGATATAGAATCTGGAAGTTGTAAAGCGTTTATCTATGGAGGATGTGATGCAAATTCTAACAACTTCGCCAAAAGACACCACTGTGAAAAGCGTTGCCGAAGATTTTCCGTTCTTGGAATTAGAAAAAGCCAC
TatKTxDel02	ACV66786.1	3 e-16	PF00014	6537.68	delta toxin kunitz type	MASFLLFFLLIALVNS**EDDVCELPPETGPCKARMLMFYYNSTCKCCDDFTYGGCNGNGNRFLTVDECLYKCAED***	AGAATTTCTATATATTTAGAGAATAAGGCTATTAGTTATAAATTCCTCGTCGAAGAGATGGCCTCATTTTTGTTATTCTTTCTGCTTATAGCTTTAGTTAATTCGGAAGACGATGTCTGTGAACTTCCTCCAGAAACAGGACCTTGCAAGGCACGTATGCTAATGTTTTATTACAACTCTACATGCAAATGTTGCGATGACTTCACTTATGGAGGATGCAATGGAAATGGCAATAGATTCTTAACAGTAGATGAGTGCTTATATAAATGTGCAGAAGATTAG

Calcium toxins

ID	ID/Reference protein	e-value	PFAM domain	MW (Da)	Sub-classification	Amino acid sequence	Nucleotide sequence
TatCaTOme01	JAV47697.1	3 e-26	PF02819	6006.83	Agatoxin-like	MKLFVTTLFLCLLFLEISSFG**DEYTRTDLSPRKGCIRKYRSCDHNKSGCCDNASCKCNLFGSNCKCQRKGMFQG***	ATGAAACTCTTCGTTACAACATTGTTTCTGTGTTTATTGTTTCTCGAAATCAGCTCATTTGGGGATGAATACACGCGAACGGACTTATCACCACGAAAAGGTTGTATTCGAAAATACAGAAGCTGTGATCATAACAAGAGTGGATGCTGCGATAATGCCTCTTGTAAATGCAATCTATTTGGGAGCAACTGCAAGTGCCAGAGAAAGGGAATGTTCCAAGGT
TatCaTClc01	A0A1L4BJ42	2 e-26	PF08099	3788.48	Calcin-like	MKQNTMTIIFIVFIVTFASLTIYGAEA*SEANFLERR***ADCLAHLKLCKKNKDCCSKKCSRRGTNPEQRCR***	ATGAAGCAAAATACAATGACCATTATTTTTATTGTTTTCATTGTCACATTTGCTTCATTGACCATCTATGGTGCAGAAGCAAGTGAAGCCAACTTTTTGGAGAGAAGGGCAGATTGCTTAGCACATCTCAAACTCTGTAAAAAGAACAAGGATTGTTGCAGCAAGAAATGTTCCAGAAGAGGAACAAATCCTGAGCAGAGATGCAGA
TatCaTClc02	P60252.1	9 e-12	PF08099	4866.3	Calcin-like	MKAIIFLITFIMATTFVSCIKFYVNPYSSSSDVDY*ADDDDQSND***DKESCAGYMEDCSKNSDCCSDRCRRKGTRSRKRCRSNPHDDD***	ATGAAGGCAATTATTTTTCTGATCACCTTTATCATGGCTACAACATTTGTTTCCTGTATCAAGTTTTACGTAAATCCCTATAGTTCTTCGTCAGACGTGGATTACGCAGATGATGACGATCAATCTAATGATGACAAAGAAAGCTGTGCAGGTTATATGGAGGATTGTAGCAAAAATAGCGACTGTTGCAGTGACAGATGTAGGAGAAAAGGGACGAGGTCAAGAAAACGATGTCGTTCAAATCCTCACGACGACGATTGA
TatCaTLio01	P0DJ08.1	7 e-32	N/D	4149.85	Liotoxin-like	MSFVAKISFLLLLVAVISTVTG*RDVTNLFESEKSDVER***DLPLSDEYQPCVRPRKCKPHLKCSKAQICVDPNKGW***	ATGAGCTTTGTAGCGAAAATATCCTTCCTTCTTTTGCTGGTAGCTGTGATCTCTACAGTCACAGGTAGAGATGTCACCAATTTATTTGAATCTGAGAAAAGTGACGTAGAAAGAGACCTTCCTCTGAGTGACGAATATCAGCCATGCGTTCGCCCCAGAAAATGTAAACCTCACCTGAAATGCAGCAAGGCACAAATCTGCGTTGATCCTAACAAAGGATGG
TatCaTLio02	P0DJ08.1	4 e-32	N/D	4178.85	Liotoxin-like	MSPAAKISLLLLLIAVMSTVT*GGDVVNLFESEKSDVER***DLPLSDEYEPCVRPRKCKPHLKCSKAQICVDPNRGW***	ATGAGCCCTGCAGCGAAAATATCCCTCCTTCTGTTGCTGATAGCTGTGATGTCTACAGTTACAGGTGGAGATGTCGTCAATTTGTTTGAATCTGAGAAAAGTGACGTAGAAAGAGACCTTCCTCTGAGTGACGAATATGAGCCGTGCGTTCGCCCCAGAAAATGTAAACCTCACCTGAAATGCAGCAAGGCACAAATCTGCGTTGATCCTAATAGAGGATGG
TatCaTLio03	P0DJ08.1	1 e-33	N/D	4149.85	Liotoxin-like	MSFVAKISFLLLLITVISTVTG*GDVLNLFESEESDTER***DLPLSDEYQPCVRPRKCKPHLKCSKAQICVDPNKGW***	ATGAGCTTCGTAGCAAAAATATCCTTCCTTCTGTTGCTGATAACTGTAATCTCTACAGTCACAGGTGGGGATGTCCTCAATTTGTTTGAATCTGAGGAAAGTGACACAGAAAGAGACCTTCCTCTGAGTGACGAATATCAGCCATGCGTTCGCCCCAGAAAATGTAAACCTCACCTGAAATGCAGCAAGGCACAAATCTGCGTTGATCCTAACAAAGGATGG

TatCaTLio04	P0DJ08.1	7 e-30	N/D	4143.81	Liotoxin-like	MSFVAKISFFLLLIAVISTVTG*RDVTNLFESEKSDVER***GFSLSDEYQPCVRPRKCKPHLKCSKAQICVDPNRGW***	ATGAGCTTTGTAGCGAAAATATCCTTCTTTCTTTTGCTGATAGCTGTGATCTCTACAGTCACAGGTAGAGATGTCACCAATTTATTTGAATCTGAGAAAAGTGACGTAGAAAGAGGCTTTTCTCTGAGTGACGAATACCAGCCTTGCGTTCGCCCCAGAAAATGTAAACCTCACCTGAAATGCAGCAAGGCACAAATCTGCGTTGATCCTAATAGAGGATGG

Host Defense Peptides (HDPs)

ID	ID/Reference protein	e-value	PFAM domain	MW (Da)	Sub-classification	Amino acid sequence	Nucleotide sequence
TatHDPDef01	KFM64187.1	2 e-12	PF01097	--	Defensin-like	ILCTFETAKAQFGFGCPLFPNDCNKHCQTLKYRGGYCTNFLERTCKCYG	ATCTTGTGCACCTTTGAAACTGCAAAAGCCCAATTTGGATTTGGATGTCCTTTGTTTCCAAACGATTGCAATAAACATTGTCAGACCTTGAAGTATAGAGGGGGATACTGTACAAACTTCTTGGAACGTACCTGCAAATGCTATGGA
TatHDPDef02	AIX87626.1	1 e-21	PF01097	4544.3	Defensin-like	MKFVAIMLIILVAFCILEDGLVEA**DYGCPFNPGKCHRSCIYLGYKEGRCKGTFKKNCACYRPKG***	ATGAAATTCGTAGCCATTATGTTAATCATCCTGGTTGCCTTCTGTATTTTGGAGGATGGACTTGTGGAAGCTGATTATGGATGTCCCTTTAATCCTGGAAAATGCCATAGAAGTTGTATATACCTTGGATACAAAGAAGGACGTTGTAAAGGAACGTTCAAGAAAAACTGTGCTTGCTATAGGCCTAAGGGTTGA
TatHDPDef03	AIX87626.1	3 e-23	PF01097	4099.64	Defensin-like	AFCILEDELVEA**GFGCPFNAGTCHNHCRSIGRRGGYCSGTFRTTCVCYKS***	GCCTTCTGTATTTTGGAGGATGAGCTTGTGGAAGCTGGTTTTGGATGTCCCTTTAATGCTGGAACATGCCATAATCATTGTAGGAGCATTGGACGCAGAGGAGGGTATTGCAGCGGAACCTTCAGGACAACCTGCGTTTGCTATAAGAGT
TatHDPDef04	AIX87626.1	1 e-17	PF01097	--	Defensin-like	AFCILEDGIVEA**EFGCPVDRKKCDRYCKSIGRSGGYCGGISGTTCICYES**	GCCTTCTGTATTTTGGAGGATGGGATTGTGGAAGCTGAATTTGGATGTCCCGTGGATCGTAAAAAATGCGATAGATATTGTAAGAGCATTGGACGCAGTGGAGGGTATTGCGGCGGAATCTCCGGGACAACCTGTATTTGCTATGAGAGT
ViVlp1	AGK88593.1	5 e-33	PF08102	6158.03	NDBP-2	MNGKALLVVFLVTMLVMEPTEA**GIFSWVKKAWNSGVGKSLRKQAVKAAKNYVANKLGGTPEEAGAMPFDEFMDVLHYN***	ATGAACGGAAAAGCACTCCTCGTTGTTTTCTTGGTTACCATGCTTGTAATGGAGCCTACTGAAGCAGGAATTTTCTCATGGGTCAAGAAAGCATGGAACTCAGGTGTAGGCAAATCACTAAGAAAACAGGCTGTCAAAGCAGCGAAAAATTACGTAGCAAATAAACTTGGAGGAACGCCCGAGGAAGCTGGAGCAATGCCGTTCGATGAATTTATGGATGTGCTGCATTATAACTAA
TatHDPND201	F1AWB0.1	7 e-52	PF08102	6510.42	NDBP-2	MNAKTIFAIFLIGMLVTEQAEA**GIWSTIKKYASKAWNSDIGKSLRNKAAGAINKFVADKIGVTPSQAASMTLDQIVDAMYYD***	ATGAATGCAAAAACCATCTTCGCTATTTTCTTGATTGGGATGCTCGTGACTGAACAAGCAGAAGCAGGAATCTGGAGTACAATTAAGAAATATGCTTCAAAAGCATGGAATTCCGACATAGGCAAATCTCTAAGAAACAAGGCAGCTGGCGCTATCAACAAATTTGTGGCAGATAAAATAGGTGTAACACCCTCACAAGCAGCCTCTATGACCCTTGATCAAATAGTAGATGCTATGTATTACGATTAA
TatHDPND301	ALG64974.1	1 e-44	N/D	2070.59	NDBP-3	MKLVNLVPVFFVLIIVVDYCHS**LPFFLLSLVPTAISAIKKL***GKRNVESQRYVDLKRRDLDEDLQALQDLLDQLSED* *	ATGAAGCTGGTTAATTTGGTTCCAGTTTTCTTTGTTCTGATAATAGTTGTTGATTACTGCCATTCTCTTCCTTTTTTCCTTCTGAGTCTCGTACCTACAGCAATCTCAGCAATCAAGAAACTTGGCAAACGAAACGTAGAATCCCAACGTTATGTGGATCTTAAGAGACGTGATCTAGATGAAGATCTGCAAGCTCTGCAGGATTTACTCGACCAGCTGTCTGAGGATTAG
ViAMP1	ALG64975.1	1 e-52	N/D	2509.97	NDBP-3	MQLRKALLVIFVAYLLVTDEAEA**FWGFLGKLAMKAIPSLIGGNKSSS***KRKREIEDLFDPYQKDLDLQRLDRFFSQFQ**	ATGCAGCTCAGAAAAGCACTGTTGGTAATCTTTGTGGCTTATCTCTTAGTGACCGATGAAGCAGAAGCTTTCTGGGGATTCCTGGGTAAACTTGCAATGAAGGCCATCCCATCGTTAATTGGAGGAAACAAAAGTTCATCAAAGAGAAAGCGTGAAATTGAAGATTTATTCGACCCATACCAAAAAGACTTAGATCTGCAACGGTTAGATCGTTTCTTCTCCCAGTTCCAGTAG
ViCT2	I0DEB3.1	1 e-32	N/D	1461.71	NDBP-4	MKTQLVVLIVALVFMQLFAQSHA**FWGAVWNAAKSIL***GKRGLRNLDDLDQFDDSFEPELSEADLKYLEDLLR**	ATGAAAACTCAGCTTGTAGTCCTGATCGTAGCTCTTGTATTCATGCAACTGTTTGCTCAGTCACATGCTTTCTGGGGTGCAGTTTGGAATGCAGCAAAGTCAATATTAGGGAAGAGAGGTCTCCGAAATTTGGATGATTTGGATCAATTTGATGATAGCTTTGAGCCTGAACTGTCAGAAGCTGATCTTAAATATTTGGAAGATCTGCTCCGATAG
ViCT7	I0DEB5.1	7 e-27	N/D	1503.77	NDBP-4	A**FLKGIIDTVSNWL***GKRSLKNLDQYNDLFDGEISDADINFLRNLMR*	GCATTTCTTAAGGGAATCATTGATACTGTGAGCAACTGGCTTGGGAAACGAAGTTTGAAGAATCTAGACCAATACAATGATTTATTCGATGGTGAAATATCCGATGCTGATATCAACTTTCTAAGGAATTTGATGCGATGA

TatHDPND401	I0DEB5.1	8 e-42	N/D	1487.87	NDBP-4	MKTQIVILIVAVLFLQLVSQSDA**FLKGIIDTVGKWL***GKRGLKNLDQYNDLFDGEISDADIKYLQDLMR**	ATGAAGACCCAAATTGTAATTTTGATAGTTGCAGTACTATTCCTTCAATTGGTCTCCCAATCAGATGCATTTCTTAAGGGAATCATTGACACTGTGGGCAAGTGGCTTGGAAAACGAGGCCTTAAGAACCTAGATCAATACAATGATCTTTTTGATGGAGAAATATCTGATGCTGATATCAAGTATTTGCAAGATTTGATGCGCTGA
TatHDPND402	I0DEB5.1	2 e-41	N/D	1487.87	NDBP-4	MKTQIVILIVAMLFLQLVSQSDA**FLKGIIDTVGKWL***GKRGLKNLDQYNDLFDGEISDADIKYLQDLMR**	ATGAAGACCCAAATCGTGATTTTGATAGTGGCAATGTTATTCCTTCAATTGGTTTCCCAATCAGATGCATTTCTTAAGGGAATCATTGACACTGTGGGCAAGTGGCTTGGAAAACGAGGCCTTAAGAACCTAGATCAATACAATGATCTTTTTGATGGAGAAATATCTGATGCTGATATCAAGTATTTGCAAGATTTGATGCGCTGA
TatHDPND403	I0DEB3.1	2 e-24	N/D	1475.83	NDBP-4	MKNQFAVLLVALVLLQLFSQSEGS**FWNTLLSVGKSLL***GKRGLRNFDFDQLDDTYEPELSEADLRYL**	ATGAAGAACCAATTTGCAGTTCTCCTCGTAGCTCTTGTTCTCTTACAACTGTTTTCTCAATCAGAAGGATCCTTCTGGAACACACTTCTAAGTGTTGGGAAGTCCTTACTGGGGAAGAGAGGTCTCAGAAATTTTGATTTTGATCAGCTGGATGACACCTATGAGCCCGAATTGTCAGAAGCTGATCTTAGGTACCTTCAAGACTTGCTACGT
TatHDPND404	I0DEB3.1	2 e-27	N/D	1313.81	NDBP-4	MKNQFVILIIAVVLLQLISQSEALWG**TLLGLGSTLLSKL***GKRGTQNLDQFDDIFEPELSEADLRYLQDLLR**	ATGAAGAACCAATTTGTCATCCTGATCATTGCCGTTGTACTCCTACAATTGATTTCTCAGTCAGAAGCCCTCTGGGGTACACTTTTAGGTTTGGGGAGTACCTTACTGAGCAAATTAGGAAAGAGAGGTACCCAAAACTTGGATCAATTCGATGATATCTTTGAGCCCGAATTGTCAGAAGCTGATCTTAGGTATTTACAAGACCTTCTGCGA
TatHDPND405	I0DEB3.1	6 e-29	N/D	1283.8	NDBP-4	MKTQFVILIIAVVLLQLISQSEALWG**ALLGLGSTLLSKL***GKRGTQNLDQFDDIFEPELSEADLRYLQDLLR**	ATGAAGACCCAATTTGTCATCCTGATCATTGCCGTTGTACTCCTACAATTGATTTCTCAATCAGAAGCCCTCTGGGGTGCACTTTTAGGTTTGGGAAGTACCTTACTGAGCAAATTAGGAAAGAGAGGTACCCAAAACTTGGATCAATTCGATGATATCTTTGAGCCCGAATTGTCAGAAGCTGATCTTAGGTATTTACAAGACCTTCTGCGATAA
TatHDPAni01	AOF40247.1	8 e-10	N/D	5893.99	Anionic peptide	MVSRTLIFLMIVALLATTFTSSYG**FPAQNMDDDDDSFDDLFDDYDDADDDSFDLDLDPADLAMLDMFANLFDSDSS***	ATGGTATCAAGGACTCTCATTTTTCTGATGATAGTGGCTCTGTTAGCAACCACCTTTACTTCATCTTATGGCTTCCCAGCTCAAAATATGGATGATGATGATGACTCGTTTGATGACTTGTTTGATGACTATGACGACGCCGATGATGACTCCTTTGATCTAGACCTGGATCCTGCTGACCTAGCGATGCTGGACATGTTTGCAAATCTATTTGACAGCGACAGTTCTTAA
TatHDPWap01	XP_015928629.1	7 e-47	PF00095	12082.96	Putative waprin-like	MYAPQLFLLVAITVWGSAE**GGSRPFPFGPRRRILPGQRASGCVDCATELKQCVQKCLQQHDCSEMDVKEGFCPAGSPRPEKCPVSAVLSVCENDTDCPGTKKCCENGCSRICANALPVPSAIRQSKRTPKLG***	ATGTACGCTCCACAACTTTTTCTACTCGTAGCTATTACAGTATGGGGGTCGGCTGAGGGTGGCAGTCGCCCTTTTCCTTTCGGTCCAAGACGTAGGATCCTACCTGGACAAAGAGCATCAGGATGCGTGGATTGCGCAACGGAATTAAAGCAGTGTGTCCAGAAATGTCTCCAGCAACATGACTGCTCAGAAATGGACGTTAAAGAAGGCTTCTGCCCAGCTGGATCACCTAGACCAGAGAAATGCCCAGTGAGTGCTGTTCTTTCAGTATGCGAAAACGACACCGACTGCCCAGGTACTAAGAAATGCTGCGAAAATGGATGCTCAAGGATCTGCGCAAATGCACTTCCGGTTCCATCAGCTATCCGACAGTCGAAGAGAACTCCAAAATTAGGT

La1-like peptides

ID	ID/Reference protein	e-value	PFAM domain	MW (Da)	Sub-classification	Amino acid sequence	Nucleotide sequence
ViLa1lp1	AOF40216.1	3 e-43	PF15430	8728.18	Other	MRHLCVVALFGCLSLCTLFSFSKG**VGEICQVGSMSINVGKKMQDPKSCVIYECVEQNYRILLSKMSCSPQVPKRGCRNVPGPVDAPFPDCCPTSLCRGKQWDE***	ATGAGGCATTTGTGCGTAGTTGCTCTTTTTGGTTGTTTATCATTATGCACTCTTTTCTCTTTTTCAAAAGGAGTTGGAGAAATTTGCCAAGTGGGGAGCATGTCAATAAATGTTGGAAAAAAGATGCAAGATCCTAAAAGCTGCGTCATTTATGAATGTGTTGAGCAAAACTATAGAATACTTCTCAGTAAAATGAGTTGTTCTCCGCAAGTACCAAAACGTGGATGCAGAAATGTGCCTGGTCCAGTTGATGCTCCATTCCCTGACTGCTGTCCCACGTCTCTTTGCCGAGGAAAACAATGGGATGAATAA
TatOthLa101	AOF40202.1	3 e-43	PF15430	8260.58	Other	MENKFTCLLLGSLLMFSFCSLSAG**FGETCQAGGKYNVRVGQPIQDPNSCVLYKCLNYNRRYVLQTLSCATQTLKSGCRFAAAAPGTPFPNCCPMVICKGSG***	ATGGAGAACAAATTCACGTGTCTGTTATTGGGCAGTTTGCTAATGTTTTCTTTTTGTTCGTTATCTGCTGGATTTGGAGAAACCTGTCAAGCAGGGGGAAAGTATAATGTTCGGGTTGGTCAGCCGATACAAGATCCAAATTCATGTGTTCTGTATAAATGTCTTAATTATAACCGGAGATATGTGCTTCAAACATTAAGTTGCGCTACTCAGACATTGAAAAGTGGATGTCGTTTTGCTGCTGCAGCCCCAGGAACACCATTTCCTAACTGTTGTCCCATGGTGATTTGCAAAGGAAGTGGTTAA
TatOthLa102	API81329.1	3 e-14	PF15430	--	Other	GEEKQHPNSCALYRCERYLSRIVLYKLICANQTMKSGCRLAAAAGRTPFPNC	GGGGAAGAAAAACAACATCCTAACTCATGTGCATTGTATAGATGCGAAAGATATCTTTCAAGGATTGTTCTTTATAAACTGATTTGCGCAAATCAGACAATGAAAAGTGGATGTCGACTTGCTGCTGCAGCTGGAAGAACACCATTTCCTAACTGT

TatOthLa103	ADY39631.1	3 e-6	PF15430	8693.97	Other	MNTTWIVVFVFLGLVLAATA**SHKDPYIRTCPIGDKDISNGEEWADQKRCVKYKCQVRGPDAALLITRCPSVGVYPTDKCREVPGKGDFPTCCPKLQCD***	TGAGATATGAACACCACTTGGATCGTTGTCTTCGTTTTCCTTGGCTTGGTACTAGCTGCTACAGCCAGCCACAAAGATCCCTACATCAGAACTTGCCCGATTGGTGACAAGGATATTAGCAATGGAGAAGAATGGGCTGATCAAAAACGCTGCGTGAAATATAAATGTCAAGTGCGTGGACCAGATGCTGCTCTGCTTATAACAAGATGCCCATCAGTTGGAGTATATCCAACAGACAAATGCAGAGAGGTTCCCGGAAAAGGTGACTTTCCCACTTGCTGTCCAAAACTGCAGTGCGACTGA
TatOthLa104	ADY39631.1	4 e-5	PF15430	8762.05	Other	MNTTWIVVFVFLGLVLAATA**QHRDPYIRTCPIGDKDISNGNEWADQKRCVKYKCQVRGPDASLLIIRCPSVGVYPTDKCREVPGKGDFPSCCPKLQCD***	ATGAACACCACTTGGATCGTTGTCTTCGTTTTCCTTGGCTTGGTACTAGCTGCTACAGCCCAGCACAGAGATCCTTACATCAGAACTTGCCCGATTGGTGACAAGGATATTAGTAATGGGAATGAATGGGCTGATCAAAAACGCTGCGTGAAATATAAATGTCAAGTGCGTGGACCAGATGCCTCTCTACTTATAATAAGATGCCCATCAGTAGGAGTATATCCAACAGACAAATGTAGAGAGGTTCCCGGTAAAGGTGATTTTCCCTCTTGCTGCCCAAAACTGCAGTGCGACTAA
TatOthLa105	L0GCW8.1	2 e-24	PF15430	9112.04	Other	MKVWNTILCIWLLVVLNVSFVVVEG**YTYVVNQKEGAVVCTDELGVEHQPGDQWFNDENCEMLLCDNATENLVIGALGCGSVYAPDCELKRGSGSYPNCCPRPVCDGNDQ***	ATGAAAGTCTGGAATACAATATTATGTATTTGGTTGTTAGTTGTACTTAATGTTTCATTCGTAGTTGTTGAAGGTTATACGTATGTAGTGAATCAGAAGGAAGGAGCAGTGGTTTGTACCGACGAATTAGGTGTTGAACACCAACCAGGTGATCAGTGGTTCAATGATGAAAATTGCGAGATGTTGCTTTGTGATAATGCTACAGAAAATTTGGTTATTGGAGCATTAGGGTGCGGCAGTGTCTATGCCCCTGATTGTGAATTAAAGCGTGGTTCTGGTTCATATCCTAACTGCTGTCCAAGACCTGTTTGTGATGGGAATGATCAGTAA
TatOthLa106	L0GCW8.1	4 e-22	PF15430	9101.01	Other	MKVWNTILCIWLLVVLNVSFVVVEG**YTYVVNQKEGAVVCTDELGVEHQPGDQWFNDENCEMLLCDNATENLVIGALGCGSVYAPDCELKRGSGSYPNCCLEPVCDGNDQ***	ATGAAAGTCTGGAATACAATATTATGTATTTGGTTGTTAGTTGTACTTAATGTTTCATTCGTAGTTGTTGAAGGTTATACGTATGTAGTGAATCAGAAGGAAGGAGCAGTGGTTTGTACCGACGAATTAGGTGTTGAACACCAACCAGGTGATCAGTGGTTCAATGATGAAAATTGCGAGATGTTGCTTTGTGATAATGCTACAGAAAATTTGGTTATTGGAGCATTAGGGTGCGGCAGTGTCTATGCCCCTGATTGTGAATTAAAGCGTGGTTCTGGTTCATATCCTAACTGCTGTCTAGAACCTGTTTGTGATGGAAATGATCAGTAA

Phospholipases

ID	ID/Reference protein	e-value	PFAM domain	MW (Da)	Sub-classification	Amino acid Sequence	Nucleotide sequence
TatEnzPA201	API81339.1	7 e-96	PF05826	21335.9	A2	MEVAIILALISAALA*ESIEKELYVNFEPLPGQRDGWPMTKAVRMQFMKRSENGR***TLRTFHGCQILQSLTDIAREVSILPKYAIRRISKQELESFQSRCEHVGEEERTIWGTKWCGAGNESSGYEDLGYFYNVDSCCRDHDHCDSIPSGGTKYSLKNEGKFTMMNCECEDAFAKCLDKVVEGTSWYDYLATLGLIKSFKGVYFNLYGNGCFHVKCNSGRSERRALCANPTAEYTGESGFAKLLNG***	ATGGAAGTAGCTATTATTCTAGCACTGATCTCTGCGGCCTTAGCAGAGTCTATCGAGAAAGAGCTATATGTGAACTTCGAGCCATTGCCTGGCCAGCGCGATGGTTGGCCAATGACGAAAGCAGTCCGTATGCAGTTTATGAAAAGATCTGAAAATGGAAGAACACTCAGAACATTCCACGGATGCCAAATACTTCAATCACTAACTGACATCGCAAGAGAAGTTAGCATCTTGCCAAAGTATGCTATTAGAAGGATATCAAAACAAGAATTGGAATCATTCCAAAGTAGGTGCGAACACGTAGGAGAGGAAGAAAGGACTATATGGGGAACTAAGTGGTGCGGAGCAGGGAATGAATCAAGTGGCTATGAAGATTTAGGATATTTTTATAATGTGGATAGCTGTTGCCGCGATCATGATCACTGCGATAGTATTCCTTCAGGTGGAACAAAGTACAGTCTGAAGAACGAAGGGAAATTTACAATGATGAATTGCGAATGCGAAGACGCGTTCGCTAAATGTCTGGATAAAGTTGTAGAAGGGACAAGTTGGTACGATTATCTTGCTACCCTGGGATTGATTAAAAGTTTCAAAGGGGTTTATTTCAATTTATATGGAAACGGATGTTTTCACGTAAAGTGCAACAGTGGACGATCAGAAAGAAGGGCATTATGTGCCAACCCAACTGCGGAGTACACTGGCGAATCCGGATTTGCAAAATTACTTAACGGTTAG

TatEnzPA202	API81335.1	7 e-73	PF05826	22373	A2	MTALFLTAFIALLATGRT*RTVQRELYLNLESSPHQEDSWP*<br>*SVRAIIVNSNERHEEGR***TFSKCRMLNSTKEAAREVSKFP**<br>**QHLIKRVSKEEMDNLERRCSGPLETRGFTDNFAFKG**<br>**TKWCGPGTMAENEDDLGPLEADKCCRAHDHCDSIE**<br>**SGGFKYNLKNNAYRTLLNCECEEAFDRCLQATADRV**<br>**EGTEKEETKMLRNYYFNILHVQCYRLYCRNGGPASE**<br>**NNCTDKFGVWMENYYEENFERKDITFY*****	AGTTTATATATGGACCATAACTGCTATACATGTATGACTGCGTTATTT<br>CTTACCGCATTCATAGCGTTGTTAGCTACAGGCCGCACTCGCACTGTC<br>CAAAGAGAACTCTATTTAAATCTGGAATCGTCACCTCATCAAGAAGA<br>TTCGTGGCCATCTGTAAGAGCAATCATAGTTAATTCCAATGAGAGGC<br>ACGAAGAAGGACGAACATTTTCTAAATGTAGGATGCTGAATTCCACG<br>AAAGAAGCAGCTAGAGAAGTATCTAAATTTCCACAACATTTGATAAA<br>AAGAGTTTCCAAAGAAGAAATGGACAACTTGGAGAGAAGATGCTCA<br>GGGCCATTAGAAACAAGAGGATTTACTGACAATTTCGCATTCAAAGG<br>AACAAAATGGTGTGGCCCTGGTACGATGGCAGAAAATGAAGACGAT<br>CTTGGACCTTTAGAAGCAGATAAGTGCTGTCGCGCTCATGATCACTG<br>TGACAGTATTGAATCAGGAGGATTTAAGTATAACCTGAAGAACAATG<br>CGTACCGTACTTTGTTGAATTGTGAATGTGAAGAAGCTTTCGATCGTT<br>GCTTGCAGGCGACTGCAGATAGGGTAGAAGGTACTGAGAAAGAAGA<br>AACTAAAATGCTTAGGAATTATTACTTTAATATTCTTCATGTTCAGTG<br>TTACCGTTTGTATTGTCGAAATGGGGGACCTGCCAGCGAAAACAACT<br>GTACAGACAAATTTGGTGTGTGGATGGAAAATTATTACGAGGAGAA<br>CTTTGAAAGAAAAGATATTACATTCTAT
TatEnzPA203	Q3YAU5.1	2 e-20	PF05826	--	A2	WTFYSPYFVYMLSRSGTKWCGAGNEATDENDLGLLYN<br>VDSCCRDHDHCDNIASGETKYGLRNNGKYTM	TGGACCTTTTATAGTCCATATTTTGTTTATATGCTTTCTCGCTCAGGA<br>ACAAAGTGGTGCGGAGCTGGAAACGAAGCCACTGATGAGAATGATC<br>TAGGGTTATTGTACAACGTTGACAGCTGTTGCCGTGACCACGATCAC<br>TGTGATAATATTGCGTCGGGTGAAACAAAGTATGGTTTGAGAAACAA<br>TGGGAAATATACAATG
TatEnzPA204	KFM56793.1	4 e-30	PF05826	--	A2	KKCRSLERKRRSLADLAKMVSLTTKRKATDFVPYGNW<br>CGIGGSGKVMDPIDKCCQVHDNCYLKARDKGICKAIVS<br>LYLGKYNWKMENGEIVCSIPEDANACDEASCLCDKEVA<br>ICLAKNIDSYQKDHRYVRSLFKS	AAGAAATGCCGATCGTTAGAGAGGAAACGACGGAGTTTAGCAGATT<br>TAGCAAAAATGGTTAGCTTAACTACCAAAAGGAAAGCAACAGATTTT<br>GTGCCATATGGAAACTGGTGTGGTATTGGCGGTAGTGGTAAAGTCAT<br>GGACCCTATTGATAAATGCTGCCAAGTGCATGATAATTGCTATTTGA<br>AAGCACGCGATAAAGGGATATGCAAAGCTATTGTCAGTCTTTATCTC<br>GGAAAATACAATTGGAAGATGGAAAATGGAGAAATTGTCTGCTCCA<br>TACCAGAAGATGCGAATGCATGTGACGAAGCAAGTTGCCTCTGCGAT<br>AAGGAAGTAGCAATATGTTTAGCAAAAAATATTGATAGCTATCAAA<br>AAGACCATAGATACGTTAGATCATTGTTTAAGAGT
TatEnzPA205	KFM56793.1	5 e-31	PF05826	--	A2	SQFSGSSDMYFKVFFIIVILASVCYQVNECRSLERKRRSL<br>ADLAKMVSLTTKRKATDFVPYGNWCGIGGSGKVMDPI<br>DKCCQVHDNCYLKARDKGICKAIVSLYLGKYNWKMEN<br>GEIVCSIPEDANACDEASCLCDKEVAICLAKNIDSYQKD<br>HRYVRSLFKS	ATCACAATTCAGTGGGTCCAGCGATATGTATTTTAAAGTCTTTTTCAT<br>TATTGTAATATTGGCCTCAGTATGCTACCAAGTTAACGAATGCCGAT<br>CGTTAGAGAGGAAACGACGGAGTTTAGCAGATTTAGCAAAAATGGT<br>TAGCTTAACTACCAAAAGGAAAGCAACAGATTTTGTGCCATATGGAA<br>ACTGGTGTGGTATTGGCGGTAGTGGTAAAGTCATGGACCCTATTGAT<br>AAATGCTGCCAAGTGCATGATAATTGCTATTTGAAAGCACGCGATAA<br>AGGGATATGCAAAGCTATTGTCAGTCTTTATCTCGGAAAATACAATT<br>GGAAGATGGAAAATGGAGAAATTGTCTGCTCCATACCAGAAGATGC<br>GAATGCATGTGACGAAGCAAGTTGCCTCTGCGATAAGGAAGTAGCA<br>ATATGTTTAGCAAAAAATATTGATAGCTATCAAAAAGACCATAGATA<br>CGTTAGATCATTGTTTAAGAGT
TatEnzPA206	JAT91104.1	7 e-85	PF05826	--	A2	MSKQTAMKTLLATILATLSLVDGGIFDAVDDVLPITTTF<br>YREKDGHRMVETIEVNTYVSGKKTVDCYMYGDNYIIN<br>KMVELIPTSITKEVDKEEMSDLVNQCSDLLLNELNSGVF<br>HFVKSPFDSIRKAFKSLLIFPGTKWCGAGNVADDYEDLG<br>RAEDTDKCCRTHDHCDDTIPGFETKYGLKNKDFYTKST<br>CDCDLTFHSCLYEGENLPSDLVGKVFFNILQTQCFKEDY<br>PQVECLEKSGIPLIRESCQEYELDYNGTKKYQFFDAKSY<br>ESKDNASILERILS	ATGTCAAAGCAAACGGCAATGAAGACTTTACTGGCAACAATTCTGGC<br>AACACTTTCGTTAGTAGATGGTGGTATTTTCGATGCAGTAGACGATG<br>TTCTTCCCATAACCACAACCTTTTATCGAGAGAAAGATGGCCATAGA<br>ATGGTTGAAACAATCGAAGTTAATACCTACGTAAGCGGAAAGAAAA<br>CTGTGGATTGTTACATGTATGGAGATAATTACATTATCAACAAGATG<br>GTAGAATTGATTCCAACAAGCATTACCAAAGAAGTGGATAAGGAAG<br>AAATGAGTGATTTGGTAAATCAATGCAGCGACCTGTTATTGAATGAA<br>CTGAACAGTGGCGTATTCCATTTTGTTAAAAGTCCTTTTGACTCCATT<br>AGGAAAGCCTTCAAATCACTGCTGATATTCCCTGGAACTAAGTGGTG<br>TGGAGCAGGTAATGTTGCTGATGATTATGAAGATTTGGGACGTGCTG<br>AAGACACCGACAAGTGTTGTAGAACACATGACCATTGCGATGACACT<br>ATACCTGGATTTGAAACGAAATATGGTCTTAAAAACAAGGATTTTTA<br>TACAAAGAGCACCTGTGATTGTGATCTGACATTTCATTCGTGTTTATA<br>TGAAGGAGAGAACCTGCCATCTGATTTAGTTGGTAAAGTATTTTTCA<br>ACATCCTTCAAACACAATGCTTCAAAGAGGATTATCCACAGGTGGAA<br>TGCTTAGAAAAAAGCGGGATACCATTAATCAGAGAAAGTTGTCAAG<br>AATATGAACTGGATTACAATGGAACAAAGAAGTACCAATTCTTTGAT<br>GCAAAAAGCTACGAATCTAAAGACAACGCCTCAATTCTTGAAAGAA<br>TACTGTCT

TatEnzPA207	API81338.1	2 e-74	PF05826	--	A2	MQFIKRSEEGREFRRFHGCQILQSLNDIAREAYRTPRHSMKRISKEEMRSFESRCERIGVDERTVLGTKWCGAGNEATDENDLGLLYNVDSCCRDHDHCDNIASGETKYGLRNNGKYTMMNCKCEDKFSACLESVIEKTNFLDYLPTLAAIKGFKAIYFDLYGNGCFNLTCSSGRSER	ATGCAGTTTATAAAGAGATCAGAAGAAGGAAGAGAGTTCAGAAGATTCCATGGATGTCAGATACTTCAATCCTTAAATGACATTGCGAGAGAAGCATACAGAACCCCAAGACATTCTATGAAAAGAATATCAAAAGAGGAAATGAGGTCGTTTGAATCAAGATGTGAACGTATTGGAGTTGATGAAAGAACTGTACTTGGAACAAAGTGGTGCGGAGCTGGAAACGAAGCCACTGATGAGAATGATCTAGGGTTATTGTACAACGTTGACAGCTGTTGCCGTGACCACGATCACTGTGATAATATTGCGTCGGGTGAAACAAAGTATGGTTTGAGAAACAATGGGAAATATACAATGATGAATTGCAAATGTGAGGACAAGTTCTCTGCATGCTTGGAGTCAGTAATAGAAAAAACTAATTTTCTTGATTATCTTCCAACTTTGGCTGCAATTAAAGGTTTCAAAGCTATTTATTTCGATCTCTATGGTAACGGATGTTTTAATTTAACATGCAGCAGTGGGCGATCAGAGAGGTGA
TatEnzPA208	API81341.1	2 e-38	PF05826	--	A2	MLSRSGTKWCGAGNEATDENDLGLLYNVDSCCRDHDHCDNIASGETKYGLRNNGKYTMMNCKCEDKFSACLESVIEKTNFLDYLPTLAAIKGFKAIYFDLYGNGCFNLTCSSGRSER	ATGCTTTCTCGCTCAGGAACAAAGTGGTGCGGAGCTGGAAACGAAGCCACTGATGAGAATGATCTAGGGTTATTGTACAACGTTGACAGCTGTTGCCGTGACCACGATCACTGTGATAATATTGCGTCGGGTGAAACAAAGTATGGTTTGAGAAACAATGGGAAATATACAATGATGAATTGCAAATGTGAGGACAAGTTCTCTGCATGCTTGGAGTCAGTAATAGAAAAAACTAATTTTCTTGATTATCTTCCAACTTTGGCTGCAATTAAAGGTTTCAAAGCTATTTATTTCGATCTCTATGGTAACGGATGTTTTAATTTAACATGCAGCAGTGGGCGATCAGAGAGGTGA
TatEnzPA209	P0C8L9.1	1 e-82	PF05826	--	A2	MQFIKRSEEGREFRRFHGCQILQSLNDIAREAYRTPRHSMKRISKEEMRSFESRCERIGVDERTVLGTKWCGAGNEATDENDLGLLYNVDSCCRDHDHCDNIASGETKYGLRNNGKYTMMNCKCEDKFSACLESVIEKTNFLDYLPTLAAIKGFKAIYFDLYGNGCFNLTCSSGRSESNAKCPNPTAAYTGESGAAKLINYLSG	ATGCAGTTTATAAAGAGATCAGAAGAAGGAAGAGAGTTCAGAAGATTCCATGGATGTCAGATACTTCAATCCTTAAATGACATTGCGAGAGAAGCATACAGAACCCCAAGACATTCTATGAAAAGAATATCAAAAGAGGAAATGAGGTCGTTTGAATCAAGATGTGAACGTATTGGAGTTGATGAAAGAACTGTACTTGGAACAAAGTGGTGCGGAGCTGGAAACGAAGCCACTGATGAGAATGATCTAGGGTTATTGTACAACGTTGACAGCTGTTGCCGTGACCACGATCACTGTGATAATATTGCGTCGGGTGAAACAAAGTATGGTTTGAGAAACAATGGGAAATATACAATGATGAATTGCAAATGTGAGGACAAGTTCTCTGCATGCTTGGAGTCAGTAATAGAAAAAACTAATTTTCTTGATTATCTTCCAACTTTGGCTGCAATTAAAGGTTTCAAAGCTATTTATTTCGATCTCTATGGTAACGGATGTTTTAATTTAACATGCAGCAGTGGGCGATCAGAGAGCAACGCCAAATGCCCCAATCCAACAGCAGCATACACTGGTGAATCTGGAGCTGCAAAATTAATTAATTATCTTAGTGGTTAA
TatEnzPA210	JAT91104.1	7 e-94	PF05826	--	A2	MKVAAAVIFALTFVFAKCGIFDIADKLLPITTTFYREKDGHRMVETIEINTYIGGKKLVDCYLYGHVYIIDKMMEVVPPDIVKEVGKKKMSKLVNTCSDLHVTKLRKGIFDIIKSPFDFARKLFKSLLIFPGTKWCGAGDVADDYDDLGVYEDTDKCCRTHDHCNDSIVGFETKYDLKNKDFYTKSSCDCDLPFHECLYQKEAIHSDAVGHLFFNILQTQCFKEEYPIVKCLKKWGIPLIRESCQKYELDYNGTKKHQFFDAKMYKGKNESPFLKQLLSH	CATATCATCACTAGCATATATAGGGTGATTATATTGTTATTTTTATTGCAGCAGAAAATGAAGGTAGCAGCGGCTGTTATTTTCGCGTTAACTTTCGTTTTCGCGAAGTGTGGGATATTTGACATTGCGGACAAACTTCTCCCCATTACCACAACCTTCTATCGAGAGAAAGATGGACACAGAATGGTTGAAACTATCGAAATTAATACTTACATCGGTGGGAAGAAACTGGTTGATTGTTACTTGTATGGACATGTGTACATTATTGACAAAATGATGGAAGTGGTTCCTCCCGATATTGTAAAAGAAGTAGGTAAAAAGAAAATGAGTAAATTGGTCAATACGTGCAGCGATTTACACGTAACTAAACTCCGAAAGGGGATTTTTGACATTATTAAGAGCCCATTCGACTTTGCCAGGAAGCTTTTCAAATCGCTTCTTATATTTCCTGGTACTAAGTGGTGTGGAGCAGGTGACGTAGCTGATGACTACGATGACCTTGGAGTATATGAAGACACTGATAAATGTTGTAGAACCCACGATCATTGCAACGATAGTATTGTTGGATTCGAAACCAAATATGATCTTAAGAATAAGGATTTTTATACAAAGAGCAGTTGTGACTGTGACTTGCCATTCCACGAATGCTTATATCAGAAAGAAGCGATACATTCAGACGCGGTTGGGCATCTATTTTTTAACATTCTGCAAACACAGTGTTTTAAAGAGGAGTATCCGATAGTAAAGTGTTTAAAGAAATGGGGGATACCACTAATAAGGGAATCTTGCCAGAAGTATGAGTTGGATTACAACGGGACGAAGAAACACCAGTTCTTCGATGCGAAGATGTATAAAGGGAAGAATGAATCTCCGTTTCTTAAGCAGTTATTGTCACAT

TatEnzPA211	JAT95961.1	8 e-39	PF05826	--	A2	IRYRLTLYTLWTPLNTTEMTYKGHQMLLLILTAFLSFVQ<br>PLPSAVIELPHENKLTGYYQSEKNPYMLIIGQTGKVIHCH<br>QYEDRNEAKRVLAALHLEDVERVTPQKMDDLLKFCTE<br>EERIEHPKKDVKKVFIYPGTKWCGMGDDAANENELGK<br>EKEADSCCRDHDHCKDSIPAFSIKHNLTNYSPFTKSHCS<br>CDREFHLCLAKAGTEAAKIISGLYFNMLKMECFQTTNCS<br>SSNETCVKTWQWTLSSSYI	TGCCACCATTTCCAACAGTCGTTCTTTAGTAGACTGCATTACATCCTT<br>AAGGGCCATCAGATGTTGCTTCTCATACTAACAGCTTTCTTATCATTT<br>GTACAACCTTTGCCATCCGCTGTTATAGAATTACCGCACGAAAACAA<br>GTTAACTGGATACTACCAAAGTGAAAAAAATCCTTATATGTTGATTA<br>TTGGACAAACAGGAAAAGTAATACACTGCCATCAGTATGAAGACAG<br>GAATGAGGCTAAAAGAGTGCTGGCAGCTTTGCATTTGGAAGATGTGG<br>AAAGAGTAACACCTCAAAAAATGGACGATCTATTAAAGTTTTGTACT<br>GAGGAAGAACGTATAGAGCATCCTAAAAAAGATGTAAAGAAGGTAT<br>TTATTTATCCAGGCACTAAATGGTGTGGAATGGGAGACGATGCAGCT<br>AATGAAAATGAATTGGGTAAAGAGAAAGAAGCTGATTCTTGTTGTCG<br>TGATCACGATCATTGTAAAGATTCAATCCCTGCATTCAGCATTAAAC<br>ATAATCTAACGAACTATTCACCATTCACTAAGAGCCACTGCAGTTGT<br>GACAGAGAATTTCATCTTTGTTTAGCCAAGGCAGGAACTGAGGCAGC<br>TAAAATAATTTCAGGTTTATATTTCAATATGTTAAAAATGGAGTGTTT<br>CCAAACGACAAACTGCAGCAGTTCCAACGAAACCTGTGTCAAAACTT<br>GGCAATGGACTTTATCCAGCTCTTATATC
TatEnzPA212	JAT95961.1	8 e-39	PF05826	--	A2	IRYRLTLYTLWTPLNTTEMTYKGHQMLLLILTAFLSFVQ<br>PLPSAVIELPHENKLTGYYQSEKNPYMLIIGQTGKVIHCH<br>QYEDRNEAKRVLAALHLEDVERVTPQKMDDLLKFCTE<br>EERIEHPKKDVKKVFIYPGTKWCGMGDDAANENELGK<br>EKEADSCCRDHDHCKDSIPAFSIKHNLTNYSPFTKSHCS<br>CDREFHLCLAKAGTEAAKIISGLYFNMLKMECFQTTNCS<br>SSNETCVKTWQWTLSSSYI	ATACGCTACAGACTAACATTATACACATTATGGACTCCCCTGAATAC<br>AACAGAGATGACCTACAAGGGCCATCAGATGTTGCTTCTCATACTAA<br>CAGCTTTCTTATCATTTGTACAACCTTTGCCATCCGCTGTTATAGAAT<br>TACCGCACGAAAACAAGTTAACTGGATACTACCAAAGTGAAAAAAA<br>TCCTTATATGTTGATTATTGGACAAACAGGAAAAGTAATACACTGCC<br>ATCAGTATGAAGACAGGAATGAGGCTAAAAGAGTGCTGGCAGCTTT<br>GCATTTGGAAGATGTGGAAAGAGTAACACCTCAAAAAATGGACGAT<br>CTATTAAAGTTTTGTACTGAGGAAGAACGTATAGAGCATCCTAAAAA<br>AGATGTAAAGAAGGTATTTATTTATCCAGGCACTAAATGGTGTGGAA<br>TGGGAGACGATGCAGCTAATGAAAATGAATTGGGTAAAGAGAAAGA<br>AGCTGATTCTTGTTGTCGTGATCACGATCATTGTAAAGATTCAATCCC<br>TGCATTCAGCATTAAACATAATCTAACGAACTATTCACCATTCACTA<br>AGAGCCACTGCAGTTGTGACAGAGAATTTCATCTTTGTTTAGCCAAG<br>GCAGGAACTGAGGCAGCTAAAATAATTTCAGGTTTATATTTCAATAT<br>GTTAAAAATGGAGTGTTTCCAAACGACAAACTGCAGCAGTTCCAACG<br>AAACCTGTGTCAAAACTTGGCAATGGACTTTATCCAGCTCTTATATC
TatEnzPA213	API81335.1	4 e-76	PF05826	23742.5	A2	MYLAAFITILSLSCSYA**AERELHVNFDPWPVARAAIVN**<br>**FDYNSETRREFSDCRMITSLDEITREGLDLPEHLIKRV**<br>**SKEEMDALEKRCSGSAEMERFGMIYPGTKWCGPGNI**<br>**SSSYSDLGELEADKCCRDHDHCDSIPAGQTKYGLSNT**<br>**GEYTLMNCDCEKAFDSCLGDAADKEYLWNSMRTKT**<br>**LRWTYFSAYSPKCYSVSCSAKRLDMEARCANGIGEW**<br>**KSSYNV**	ATGTATCTCGCTGCATTTATAACAATTCTATCATTGTCCTGCTCTTAT<br>GCTGCCGAAAGAGAACTCCATGTAAATTTTGACCCTTGGCCCGTAGC<br>AAGAGCCGCTATTGTGAATTTTGATTACAACTCTGAAACGAGAAGAG<br>AATTTTCTGACTGCCGCATGATTACTTCGCTGGATGAGATCACTAGA<br>GAAGGTCTGGATTTACCAGAACATTTGATAAAAAGGGTTTCAAAAGA<br>AGAAATGGATGCCTTAGAGAAAAGATGCTCAGGTTCTGCAGAAATG<br>GAACGATTTGGAATGATATATCCTGGAACAAAATGGTGCGGACCTGG<br>AAACATTTCAAGCAGTTATTCTGATCTAGGAGAACTAGAAGCAGACA<br>AATGTTGCCGTGATCACGACCACTGTGACAGCATTCCAGCTGGTCAA<br>ACTAAATATGGTCTTTCAAATACTGGCGAATATACATTAATGAACTG<br>CGACTGTGAAAAAGCCTTTGACTCCTGTTTGGGAGATGCTGCTGATA<br>AAGAATATTTATGGAATAGTATGAGGACTAAAACCTTAAGGTGGAC<br>GTACTTCAGCGCTTATTCACCGAAATGTTACTCTGTTAGTTGCTCCGC<br>AAAGAGATTAGATATGGAGGCAAGATGTGCGAATGGGATTGGTGAA<br>TGGAAGAGTAGTTACAACGTATAG

TatEnzPA214	XP_015925461.1	7 e-51	PF05826	--	A2	MMMTPLNLVLAMLSVVARSVSNEANGRFELTTTFYIEK<br>DGHRMVEMVEGARYKGRSENVACYVYGDRYTIDRMM<br>KFVEKNSMKDVTRAEMRELLNQCSSLNQPEKEEFRRM<br>KNPINYIKNITDKFGIFPGTKWCGSGNIAENDDDLGSSVE<br>TDKCCRAHDKCNDSIPANGTKHGLKNDGSYTKSNCNC<br>DEEFYSCLKRVDDKTSTAVGTTYFDILRTQCFKYEYPIIK<br>CEKTCSILRRNCENYELNYNATKKYQFFDARCFL	ATGATGATGACACCACTGAATCTGGTCTTGGCAATGTTGTCTGTAGT<br>AGCGAGAAGTGTTTCTAACGAAGCTAATGGTAGGTTTGAATTAACCA<br>CAACCTTCTATATAGAAAAGGATGGCCACAGAATGGTAGAAATGGT<br>CGAAGGTGCTAGATACAAGGGCAGAAGTGAAAACGTTGCTTGCTAC<br>GTTTATGGAGACAGATACACTATTGACAGAATGATGAAGTTTGTTGA<br>AAAAAATTCCATGAAGGATGTAACCAGAGCAGAAATGAGAGAATTG<br>CTGAATCAATGTAGTTCATTGAACCAACCGGAAAAAGAAGAATTTCG<br>CAGAATGAAAAATCCCATTAATTATATTAAGAATATTACTGATAAAT<br>TTGGAATCTTTCCTGGTACTAAATGGTGCGGATCTGGGAATATAGCT<br>GAGAATGACGACGATTTGGGCAGTTCAGTAGAAACAGACAAATGCT<br>GTAGGGCTCATGACAAATGTAATGATTCTATACCTGCCAATGGAACA<br>AAACATGGCCTTAAAAATGATGGATCTTATACTAAGAGTAATTGTAA<br>TTGCGATGAAGAGTTTTACTCCTGCTTGAAAAGGGTGGACGATAAAA<br>CGTCTACAGCTGTCGGTACAACATATTTCGATATCTTGAGGACACAG<br>TGCTTCAAATACGAATACCCCATAATTAAATGCGAGAAGACGTGTAG<br>CATACTTAGAAGAAACTGCGAGAACTATGAGCTGAATTACAATGCA<br>ACGAAGAAATACCAATTTTTTGACGCTAGATGCTTTTTATAA
TatEnzPA215	API81335.1	2 e-88	PF05826	--	A2	MYLAVFTALLSLCCSRAAQRELYINFEPLPGQRDSWPIA<br>RAAIVNFEEKSEMGREFSGCRMISSVDELAREGTDLPEH<br>LIKRASKEEMDALQERCSGSAEMERFTMIYPGTKWCGP<br>GNKAKNESDLGSLEADKCCRAHDHCDNIGAGKSKYGL<br>TNTGSFTLLNCDCENTFDRCLSDAAEKEGWFKKQGTKA<br>LRWTYFTAYSPKCYSLSCNKKRSILEARCANPVGKWKE<br>NYKL	TGGATTTTGCAAGCACATCTTGGAACGCATCCATCTTTGAAGGGAAC<br>AGACTTTCCAGTCAATATGTATCTCGCAGTATTTACAGCACTTTTGTC<br>TTTGTGCTGCTCTCGTGCTGCTCAAAGAGAACTCTACATAAATTTTGA<br>ACCACTACCTGGTCAAAGAGATTCTTGGCCCATAGCAAGAGCCGCCA<br>TTGTGAACTTTGAGGAAAAATCCGAAATGGGAAGAGAATTTTCTGGA<br>TGTCGCATGATCAGTTCTGTAGATGAACTGGCTAGAGAAGGAACGGA<br>TTTGCCGGAGCATCTGATTAAAAGAGCTTCCAAAGAAGAAATGGATG<br>CTTTACAGGAAAGATGCTCGGGTTCTGCAGAAATGGAACGCTTCACA<br>ATGATATATCCCGGAACAAAATGGTGTGGACCTGGTAACAAGGCGA<br>AAAACGAATCTGACCTCGGTAGCTTGGAAGCAGATAAATGTTGCCGC<br>GCTCACGACCACTGTGATAATATTGGAGCTGGTAAATCTAAATATGG<br>CCTAACAAACACAGGAAGTTTTACATTATTGAACTGTGATTGCGAAA<br>ATACATTTGACAGATGTTTGAGTGATGCTGCTGAAAAGGAAGGTTGG<br>TTTAAAAAACAGGGCACAAAAGCATTGAGGTGGACGTATTTCACCGC<br>TTATTCACCAAAATGCTATTCACTCAGTTGCAATAAAAAGAGATCTA<br>TTCTGGAGGCTAGATGTGCTAATCCTGTCGGAAAGTGGAAGGAAAAC<br>TATAAACTA
TatEnzPA216	XP_012232705.1	3 e-40	PF05826	--	A2	MVVMIESGRTETPFCFRYGDRRTFQEIVKLIPKDIVKEVS<br>KEEMTELLNKCSEFSFNELENEAFRIQKRQIATYEEFLDL<br>IKRILRFYSISESSIGTFLKSLADYISKNGVISPENPLLFPGT<br>KWCGPGNRGENYNDLGIFSDTDACCRDHDNCNNSIKV<br>GETKCNLTNNGLLTRMSCDCDDALYSCLKNSCNVISEL<br>VGIMFFNVLRNKCFEEEYPQVRCKKPCSTLLFLENCNEY<br>ELDTTKEKKCQFFDPRCYYASQNCLLSTPNGTSPTLLKIL<br>LQLLGIN	ATGGTTGTAATGATTGAAAGTGGCAGAACCGAAACACCTTTTTGCTT<br>TCGTTATGGCGATAGGCGTACATTTCAAGAAATTGTGAAGTTGATTC<br>CAAAGGATATTGTAAAGGAAGTGAGCAAAGAAGAGATGACCGAATT<br>ATTGAATAAATGTAGCGAATTTTCGTTCAACGAATTGGAAAATGAGG<br>CTTTTCGCATCCAGAAAAGACAAATTGCTACCTACGAAGAGTTTTTG<br>GATCTGATTAAACGTATTCTTCGTTTTTATTCGATTTCTGAGAGTTCA<br>ATTGGAACGTTCCTGAAGAGTCTTGCCGACTACATTAGTAAAAATGG<br>TGTTATTAGTCCTGAAAATCCTTTGCTGTTTCCTGGAACTAAATGGTG<br>CGGACCTGGAAACAGAGGTGAAAATTACAACGATTTGGGGATTTTTA<br>GTGACACAGATGCATGCTGTAGGGACCATGATAATTGCAATAACTCC<br>ATAAAAGTAGGAGAAACGAAGTGCAATCTTACAAATAATGGACTTTT<br>AACAAGGATGAGTTGTGATTGCGATGATGCGCTATACTCTTGTTTGA<br>AAAACTCATGTAATGTGATATCTGAGCTTGTTGGAATAATGTTCTTCA<br>ACGTTTTGAGAAACAAGTGCTTCGAAGAAGAATATCCACAAGTTAGG<br>TGTAAGAAACCGTGTAGCACACTTCTGTTCCTGGAAAATTGCAATGA<br>ATATGAATTGGATACTACTAAAGAAAAGAAATGCCAATTCTTTGATC<br>CGAGATGCTACTATGCCTCTCAAAATTGTTTGCTTTCCACTCCCAATG<br>GTACCTCACCAACCTTGTTGAAAATATTGTTGCAACTATTAGGCATA<br>AACTGA

TatEnzPhB01	XP_013790770.1	0	PF04916	61028.7	B-like 2	MECYVIIFCLLPCLVMA**LPPRNVSVSWDSIKGDFSIHDT**<br>**VVKDWVAWASFSNEINSTGWSYLEVHTSEYYKDSYQ**<br>**AYAAGLVEGYVTSDLIKKHWNNLYADYCDGEKAYC**<br>**DRLQQFLKINLDFLNDQISNNRTNAYWHQVALVLEQ**<br>**VKGLEDGYNNKPSFPTTRPSIFGPMFFNLFGDLEDLE**<br>**AVLNKTVKKRVLGSGHCSGLIKLLPGYKDLYVAQDS**<br>**WNTYNSMLRILKKYVMPLKSSTSTGSKRIAGSTMSFS**<br>**SYPATIFSGDDFYIISSRLIALETTIGNSNSSLWKYVVP**<br>**NKIVLEWIRNIVANRLARTGEAWTYLFSLYNSGTYNN**<br>**QWMVVDYNRFFPGQSPRRGLLWVLEQLPGHIERKD**<br>**QTHHLLKETYWPSYNSPFYPDIFNLSGTLDMVKKYG**<br>**DWFTYDKTPRALIFKRDHKKVHDLASMMHLMRYND**<br>**YKNDALSRCNCTPPYSAENAIAARSDLNPPNGTYPFPS**<br>**LGHRSHGAIDMKLTNYSMHMKYQFVAYGGPTYDQQ**<br>**PPFQWSKSDFGSEHHDGHPDLWKFKPVVHKWLSDN**<br>*****	ATGGAGTGCTATGTGATAATTTTCTGTCTTCTGCCATGTCTTGTAATG<br>GCATTACCTCCAAGAAATGTATCAGTTTCATGGGATTCTATTAAAGG<br>AGATTTCTCCATTCATGACACAGTTGTTAAGGACTGGGTAGCTTGGG<br>CTAGTTTTTCTAATGAAATAAATAGTACTGGTTGGTCGTACTTAGAG<br>GTTCACACAAGTGAATACTATAAGGATTCTTATCAAGCTTATGCTGC<br>AGGGCTTGTTGAGGGATATGTAACATCTGATCTGATTAAAAAGCATT<br>GGAACAATTTATATGCTGACTATTGTGATGGAGAAAAAGCATATTGT<br>GATAGGTTGCAACAGTTTTTGAAGATAAACCTGGATTTTCTGAATGA<br>TCAAATTAGCAACAACAGAACAAATGCATACTGGCATCAGGTAGCTT<br>TAGTGTTGGAACAAGTAAAAGGGTTGGAAGATGGTTACAATAATAA<br>ACCTTCCTTTCCTACCACAAGACCAAGCATCTTTGGACCAATGTTTTT<br>TAATTTGTTTGGTGATTTGGAAGATTTAGAAGCTGTTCTTAACAAAAC<br>AGTTAAAAAACGAGTATTAGGAAGTGGTCATTGCTCTGGATTAATCA<br>AACTTTTACCTGGATACAAAGATTTATATGTTGCTCAAGATTCATGG<br>AATACATACAATTCTATGCTTCGTATTCTTAAAAAATATGTTATGCCA<br>CTTAAGTCTTCTACATCTACAGGTAGCAAGAGAATAGCTGGCAGCAC<br>AATGAGCTTTTCCTCTTATCCTGCCACAATATTTTCTGGTGATGATTT<br>TTATATAATATCAAGTAGACTGATTGCTCTTGAGACAACAATTGGTA<br>ATAGTAATTCAAGTCTTTGGAAGTATGTTGTGCCAAACAAAATTGTG<br>TTAGAGTGGATTAGAAATATTGTTGCCAACAGACTAGCAAGGACAG<br>GTGAAGCTTGGACATACTTGTTCAGTCTTTACAATAGTGGAACTTAC<br>AACAACCAATGGATGGTTGTTGATTACAATAGGTTTTTTCCTGGTCA<br>ATCTCCAAGGAGAGGTTTGCTTTGGGTTTTAGAACAATTACCGGGAC<br>ATATTGAACGTAAGGATCAGACACATCACCTTCTAAAAGAAACATAT<br>TGGCCATCATACAACAGTCCCTTTTATCCTGATATTTTCAATCTCAGT<br>GGAACTTTGGACATGGTGAAAAAATATGGAGATTGGTTTACATATGA<br>CAAAACTCCTCGTGCCCTCATTTTTAAAAGGGATCATAAAAAAGTGC<br>ATGATTTGGCATCCATGATGCATCTGATGAGGTATAATGACTACAAA<br>AATGATGCCCTCTCAAGATGCAACTGCACTCCACCATACAGTGCTGA<br>AAATGCAATTGCTGCAAGGAGTGACCTCAATCCACCAAATGGAACTT<br>ATCCATTTCCATCCTTGGGGCATCGTTCTCATGGAGCTATTGACATGA<br>AGCTTACAAATTATTCCATGCATATGAAATACCAGTTTGTGGCATAT<br>GGTGGACCTACTTATGACCAGCAACCACCCTTTCAGTGGAGCAAGTC<br>TGATTTTGGTAGTGAACACCATGATGGTCATCCTGATCTATGGAAAT<br>TCAAGCCTGTAGTTCATAAGTGGCTAAGTGACAAT
TatEnzPhD01	API81381.1	7 e-77	N/D	--	D	QLFHHVNDNAFLFSTGVISEGAKSQLWILLSLPYSTDSDF<br>IIGFKQTLKVYGYQHMQHRVGWDMYGNERLDVIKKTY<br>QKLGISTAVWQGDGINNCLPRSGRRLRKAIHKRDCDAE<br>WTFLKKVYYWTVDKSSTMKEALRMGVDAILTNRPDKF<br>YSALGDDEFSTTHRLATIRDNPWQKVRGSKRSVYC	CAGCTTTTTCACCATGTCAACGACAATGCTTTTCTATTTAGTACCGGC<br>GTTATTTCAGAGGGAGCCAAGTCACAGTTATGGATACTGCTGTCTTT<br>GCCATATAGCACCGACTCCGATTTTATAATAGGGTTTAAGCAGACAC<br>TGAAAGTATATGGTTACCAACATATGCAGCACAGGGTTGGATGGGAT<br>ATGTATGGAAATGAAAGATTAGATGTAATCAAGAAGACGTACCAAA<br>AACTAGGAATTTCCACAGCAGTATGGCAGGGCGATGGGATCAACAA<br>CTGCTTGCCTCGTTCCGGTAGGCGTTTGCGGAAAGCAATACACAAAA<br>GAGATTGCGATGCAGAATGGACATTTCTCAAAAAGGTTTATTACTGG<br>ACGGTAGACAAATCAAGTACCATGAAGGAAGCCCTAAGGATGGGAG<br>TAGATGCAATCCTCACCAATCGCCCCGACAAGTTTTACTCGGCTCTA<br>GGAGATGATGAATTTTCTACAACCCATCGATTGGCAACAATAAGAGA<br>TAATCCATGGCAGAAAGTGCGAGGATCTAAAAGATCAGTATATTGC

TatEnzPhD02	API81381.1	0	N/D	--	D	MSTTMLFYLVPALFQILFSSAEVRPIWNIGHMVNSLSQID<br>QFLNLGANALETDFTFNSDGVAEWTYHGYPCDCFRSCR<br>RYATVEDYLRYVRELTSPDSPKFRSDFVLLQIDLKVSGL<br>SDDATHNAGVDVAVKLIRYLWSGKGAKSQLWILLSFPY<br>STDSDFVIGFMQTLRANGYQNMHQRVGWDMSGNESLD<br>VIKKTYQRLGVSRAVWQSDGITNCLPRSIKRLLEAIHRR<br>DWDAEWEFLDKVYYWTLDKSSSMKQALRIGVDAIITN<br>HPDRFYSVLGDDEFSTTHRLATIRDNPWQKVRGAKRYL<br>SQYTADVNYVFYECNLEEETEKEDIDE	ATGGTAAATTCACTCTCTCAAATTGATCAGTTCCTCAACCTAGGCGC<br>AAATGCTTTGGAGACAGATTTCACATTTAACAGTGACGGAGTCGCTG<br>AATGGACGTACCATGGGTATCCCTGTGACTGTTTTAGAAGCTGCAGG<br>CGATACGCGACAGTTGAAGATTATCTTCGTTATGTTAGAGAACTGAC<br>TTCTCCAGACAGTCCCAAATTCCGTAGTGACTTTGTTCTGTTACAAAT<br>TGACCTGAAAGTCTCAGGATTGTCAGATGATGCGACACACAACGCTG<br>GTGTAGATGTTGCTGTGAAACTTATTAGGTATTTGTGGAGCGGAAAG<br>GGAGCCAAGTCACAATTGTGGATACTGCTGTCTTTTCCATATAGCAC<br>AGATTCCGATTTCGTAATAGGGTTTATGCAGACACTGAGAGCAAATG<br>GTTACCAAAATATGCACCAAAGGGTTGGATGGGATATGTCTGGCAAC<br>GAAAGTTTAGATGTAATCAAGAAGACATATCAAAGATTAGGAGTTTC<br>CAGAGCAGTATGGCAGAGCGATGGGATCACCAACTGTTTGCCACGTT<br>CCATTAAACGTTTGCTAGAAGCAATACACAGAAGAGACTGGGATGC<br>AGAATGGGAATTTCTGGACAAGGTTTATTACTGGACGTTAGATAAAT<br>CGAGTTCCATGAAGCAAGCCCTAAGGATAGGAGTAGATGCAATCAT<br>CACCAACCACCCTGACAGGTTTTACTCGGTTCTGGGAGATGACGAAT<br>TTTCTACAACCCATCGGTTAGCAACTATAAGAGACAATCCATGGCAG<br>AAGGTACGAGGAGCTAAAAGATATCTCAGCCAGTACACTGCTGATGT<br>TAACTATGTATTTTATGAATGTAACCTTGAAGAAGAAACCGAGAAGG<br>AAGATATAGATGAA
TatEnzPhD03	XP_002399661.1	7 e-78	N/D	--	D	KYLSQDLTYFIRYLKIIGVDKGISTFIMIENISLIIILLSSVV<br>QVQGTEEDRRHPVYIIAHMANSIHEVDDYLRRGANAIE<br>ADVTFTSKGEVDSIYHGYPCDCFRVCSRKEKFSKYISYIR<br>NITTPGTKNYKENFTLLFLDLKVKNLIPQLKYAAGIKLT<br>KALVKYLWNGGDNVTVINVLLSVGTTEDSQVIRAAKRT<br>LQRDNLTYLLPRIGFDVGMNDHPLKISNMWKSLGMELN<br>KWQGDGISNCVFPMRSTRRLDWLLRKQRDGESYMSKI<br>YQWTVDFSVHFRRYLGSGVEAFITNYPERLSSLLQESKI<br>RQFYRLATINDNIWERVPIYPSVVRAEVTDTPISSNVYTI<br>GQEVMDSLATFFTDAFSNTINRGVINSEYLSLMNYFADT<br>NNDRSEPEQPDPQDFCRYSLCLFYQLYSNQTASRRLQFV<br>TDCMQRSDSPFMEISTICRSLDKLVHNLFVSNVTSVA	AAGTATCTGTCCCAAGATTTAACATACTTCATCAGATACCTGAAAAT<br>AATTGGAGTGGACAAAGGTATAAGCACTTTCATTATGATTGAAAACA<br>TTTCGTTAATCATCATTCTTCTGTCATCAGTTGTGCAGGTGCAGGGGA<br>CAGAAGAAGATAGAAGGCATCCTGTTTACATCATTGCTCACATGGCG<br>AACAGCATTCATGAAGTTGATGACTACCTACGTAGAGGAGCTAATGC<br>TATCGAAGCAGATGTAACGTTTACTAGTAAAGGAGAGGTAGACTCA<br>ATTTACCACGGATATCCTTGCGACTGCTTTCGTGTATGCTCGAGAAA<br>AGAAAAATTCTCCAAATATATTTCTTACATTCGTAATATTACAACACC<br>AGGTACTAAAAACTACAAGGAAAATTTTACACTATTATTTCTGGACC<br>TAAAAGTAAAAAATTTGATTCCACAATTGAAATACGCTGCTGGAATA<br>AAGCTCACTAAAGCGTTAGTAAAATATCTATGGAATGGTGGCGATAA<br>CGTCACTGTGATAAATGTTCTGTTATCCGTTGGTACCACGGAAGATA<br>GTCAAGTAATAAGAGCTGCAAAACGCACTCTTCAACGAGATAACTTA<br>ACATATCTGCTTCCAAGAATAGGTTTCGATGTTGGGATGAACGATCA<br>CCCACTTAAGATTTCCAACATGTGGAAGAGTTTAGGAATGGAGCTGA<br>ACAAATGGCAAGGTGATGGAATAAGCAATTGTGTCTTTCCAATGAGA<br>TCTACTAGGAGACTCGACTGGCTTCTAAGGAAACAAAGAGATGGAG<br>AAAGTTACATGAGTAAAATATATCAATGGACAGTGGATTTCTCTGTA<br>CATTTCAGAAGATACCTTGGGTCAGGTGTAGAAGCGTTCATCACCAA<br>TTATCCTGAGAGATTAAGCAGTTTACTTCAAGAGTCGAAAATTCGTC<br>AATTCTACAGACTAGCAACAATAAATGATAATATCTGGGAACGCGTG<br>CCAATTTACCCCTCTGTTGTCAGAGCTGAAGTAACCGATACACCAAT<br>CTCTTCTAACGTTTATACAATTGGACAAGAAGTTATGGACTCCTTAGC<br>AACCTTCTTTACCGATGCCTTTAGTAACACCATTAATCGAGGGGTTAT<br>CAACTCAGAATATCTTTCATTGATGAATTACTTCGCGGACACTAACA<br>ATGACAGATCAGAACCTGAGCAACCAGATCCTCAAGACTTTTGTCGG<br>TATTCATTGTGCCTCTTTTACCAACTATATAGTAATCAAACTGCATCA<br>CGCCGCTTGCAGTTTGTCACTGACTGTATGCAAAGAAGCGATTCACC<br>ATTTATGGAAATTTCGACCATATGTAGGTCTCTAGACAAACTAGTAC<br>ATAATTTATTTGTATCTAATGTGACTTCAGTTGCA

TatEnzPhD04	XP_015792031.1	1 e-77	N/D	--	D	KYLSQDLTYFIRYLKIIGVDKGISTFIMIENISLIIILLSSVV<br>QVQGTEEDRRHPVYIIAHMANSIHEVDDYLRRGANAIE<br>ADVTFTSKGEVDSIYHGYPCDCFRVCSRKEKFSKYISYIR<br>NITTPGTKNYKENFTLLFLDLKVKNLIPQLKYAAGIKLT<br>KALVKYLWNGGDNVTVINVLLSVGTTEDSQVIRAAKRT<br>LQRDNLTYLLPRIGFDVGMNDHPLKISNMWKSLGMELN<br>KWQGDGISNCVFPMRSTRRLDWLLRKQRDGESYMSKI<br>YQWTVDFSVHFRRYLGSGVEAFITNYPERLISVLQEKNI<br>RQHYRLATANDNPWETVPNYPTVISDQTANQPISSNVYS<br>IAQEAVESLMMFVGNVFNININRRISNSDIFTTIDYLVNG<br>YRSESELTEPEIFCQYALCTLYEIYSNQTIAYRFKLESKC<br>MQKSGTPGVEISTICKSLHKMVSPWPII	AAGTATCTGTCCCAAGATTTAACATACTTCATCAGATACCTGAAAAT<br>AATTGGAGTGGACAAAGGTATAAGCACTTTCATTATGATTGAAAACA<br>TTTCGTTAATCATCATTCTTCTGTCATCAGTTGTGCAGGTGCAGGGGA<br>CAGAAGAAGATAGAAGGCATCCTGTTTACATCATTGCTCACATGGCG<br>AACAGCATTCATGAAGTTGATGACTACCTACGTAGAGGAGCTAATGC<br>TATCGAAGCAGATGTAACGTTTACTAGTAAAGGAGAGGTAGACTCA<br>ATTTACCACGGATATCCTTGCGACTGCTTTCGTGTATGCTCGAGAAA<br>AGAAAAATTCTCCAAATATATTTCTTACATTCGTAATATTACAACACC<br>AGGTACTAAAAACTACAAGGAAAATTTTACACTATTATTTCTGGACC<br>TAAAAGTAAAAAATTTGATTCCACAATTGAAATACGCTGCTGGAATA<br>AAGCTCACTAAAGCGTTAGTAAAATATCTATGGAATGGTGGCGATAA<br>CGTCACTGTGATAAATGTTCTGTTATCCGTTGGTACCACGGAAGATA<br>GTCAAGTAATAAGAGCTGCAAAACGCACTCTTCAACGAGATAACTTA<br>ACATATCTGCTTCCAAGAATAGGTTTCGATGTTGGGATGAACGATCA<br>CCCACTTAAGATTTCCAACATGTGGAAGAGTTTAGGAATGGAGCTGA<br>ACAAATGGCAAGGTGATGGAATAAGCAATTGTGTCTTTCCAATGAGA<br>TCTACTAGGAGACTCGACTGGCTTCTAAGGAAACAAAGAGATGGAG<br>AAAGTTACATGAGTAAAATATATCAATGGACAGTGGATTTCTCTGTA<br>CATTTCAGAAGATACCTTGGGTCTGGCGTGGAAGCCTTCATTACAAA<br>TTACCCTGAGAGGTTAATCAGCGTACTCCAAGAAAAGAATATTCGTC<br>AGCACTACCGGCTAGCGACAGCAAATGATAATCCCTGGGAAACAGT<br>TCCGAATTATCCAACTGTTATTAGCGACCAAACAGCTAATCAGCCAA<br>TCTCTAGCAATGTTTATAGTATTGCTCAAGAAGCCGTGGAATCTCTG<br>ATGATGTTTGTTGGAAATGTTTTTAATATAAATATCAACAGAAGGAT<br>CAGTAACTCGGATATTTTTACCACAATTGACTACTTAGTTAATGGATA<br>TAGATCAGAGTCTGAATTAACGGAGCCTGAAATCTTCTGTCAGTATG<br>CTCTATGCACTCTATACGAAATATATAGCAATCAGACTATAGCGTAT<br>CGCTTCAAGCTTGAAAGTAAATGCATGCAGAAGAGTGGCACACCAG<br>GTGTGGAAATATCGACGATATGTAAGTCTCTGCATAAAATGGTTTCT<br>CCATGGCCCATTATC

Hyaluronidases

ID	ID/Reference protein	e-value	PFAM domain	MW (Da)	Sub-classification	Amino acid sequence	Nucleotide sequence
TatEnzHya01	API81375.1	2 e-160	PF01630	--	Other	MSIFSTFIISIFAFSIVKANFEVFWNVPSLLCSIKFGVNLTQ<br>TLLKYKILVNNGESFIGDKIALIYENGIGKYPHIDPKKGDI<br>NGGIPQLDKLNEHLKLAENDIQKLIPNPDFNGLGIIDWEA<br>WRPIWEYHWGSLGIYKNRTLEMVKKDHPTWSEQLVQS<br>TAKNIWENSAKQWMLKTLELAKKLRPHGRWCYYLFPD<br>CYNYFGKDQPSQFFCSAMIQNNNDRLSWMWDASTALC<br>PSIYFIENQMKYNGSQRTWFLYGKLAEAVRVARPHTRIY<br>PYINYMVHVSRIPVPEDHFWKMLSLIASLGLDGAIIWGS<br>SSYLSDITSCQDLETYVNNVIGPAVTTVSSNVERCSQMV<br>CNGRGKCTWPNDPFTSWKYLTDINSDFDSKEITCRCQT<br>HKGRYCD	ATGTCCATATTTTCTACATTTATAATTTCTATTTTTGCATTTAGTATTG<br>TCAAGGCCAATTTTGAAGTCTTCTGGAACGTACCTTCTTTGTTATGTT<br>CGATTAAGTTCGGTGTAAATCTGACTCAAACTCTTTTGAAGTATAAA<br>ATATTGGTGAACAACGGGGAAAGTTTTATTGGGGATAAAATAGCCTT<br>AATTTATGAAAATGGAATTGGAAAGTATCCACACATAGATCCTAAGA<br>AAGGTGACATCAATGGAGGAATTCCACAATTGGATAAATTGAATGA<br>GCATTTAAAATTGGCAGAGAATGATATTCAGAAACTAATACCAAATC<br>CCGACTTTAATGGTCTTGGAATTATCGACTGGGAAGCATGGCGTCCA<br>ATCTGGGAGTATCATTGGGGATCGTTGGGCATTTACAAGAATAGGAC<br>TTTGGAAATGGTGAAGAAAGATCATCCAACATGGTCTGAACAGCTCG<br>TCCAAAGTACAGCCAAGAATATATGGGAGAACAGCGCTAAGCAATG<br>GATGCTGAAAACGTTGGAACTTGCAAAGAAGTTACGTCCACATGGA<br>AGATGGTGTTACTATCTTTTTCCCGACTGTTATAATTATTTTGGAAAG<br>GATCAGCCATCTCAATTCTTTTGTAGTGCTATGATACAAAATAATAA<br>CGATAGATTGTCTTGGATGTGGGACGCCAGTACTGCCCTCTGTCCAT<br>CGATATATTTTATTGAAAATCAAATGAAATATAATGGGAGTCAACGT<br>ACGTGGTTTCTTTATGGAAAATTAGCTGAAGCTGTAAGAGTTGCTCG<br>ACCGCACACAAGAATATACCCATATATTAACTATATGGTCCACGTTT<br>CCCGGATTCCAGTTCCAGAGGACCATTTCTGGAAAATGCTGTCTCTT<br>ATAGCATCCCTGGGATTAGATGGAGCAATTATATGGGGATCATCCAG<br>CTATCTCTCAGACATAACCTCTTGCCAAGATCTTGAAACTTACGTTAA<br>TAATGTGATTGGTCCTGCCGTAACTACAGTTTCATCGAATGTCGAAC<br>GGTGTAGCCAAATGGTCTGCAATGGAAGAGGAAAATGTACATGGCC<br>AAATGATCCGTTTACTTCGTGGAAATATTTAACTGACATAAATAGCG<br>ATTTTGATTCCAAAGAAATTACATGCAGATGTCAAACCCATAAAGGA<br>AGATATTGTGATTAA

TatEnzHya02	API81375.1	0	PF01630	--	Other	MNSRILIGIILNYIICVIEASFKIYWNVPSYMCSVRFGINV<br>TETLINSNVMVNNKEEFLGEKLVIFYENRLGKYPYIDPK<br>EGDINGGLPQLGNLEEHLEVAEKDIQEIIPNPNFDGLGIID<br>WEKWRPIWDFNWGKMRIYKTRSMELMKKKHPSWPWK<br>LIENASRKEWEETAMQWMLKTIQLTKKMRPKGRWCFY<br>HLPDCYNYAGKDNPEQFLCSSLVRKHNDRLIWLWNSTT<br>ALCPSIYYDERQMKYNETQDVWFLYGRLCEVMRVSQP<br>NTPIYPYINYKVHTSLENVPKEHFWLMLSHLASLGLDGA<br>VIWGSSNYVKTEEDCKSLELYVKEVIGPAAYTISTNVNY<br>CSETVCNNNGLCTWPHQPYTSWKYLTDPNITVFNPENIT<br>CRCQLYKGRYCDMFESNSTDLL	ATGAATTCGCGCATTTTGATAGGGATAATTTTAAACTATATTATCTGT<br>GTGATCGAGGCATCTTTTAAAATCTACTGGAATGTTCCTTCTTATATG<br>TGCTCAGTGAGATTTGGCATCAATGTTACAGAAACGCTGATAAATAG<br>CAATGTGATGGTGAACAACAAAGAAGAATTTCTTGGTGAGAAGTTG<br>GTGATTTTTTATGAAAATAGATTAGGAAAATATCCATATATTGATCC<br>CAAAGAAGGTGATATTAATGGAGGGTTACCGCAGTTAGGAAATCTA<br>GAAGAACATTTAGAAGTGGCAGAAAAGGATATTCAAGAAATAATTC<br>CCAATCCAAATTTTGATGGACTTGGAATAATTGATTGGGAAAAATGG<br>CGTCCGATTTGGGATTTCAATTGGGGAAAAATGAGAATTTACAAGAC<br>AAGGTCGATGGAGCTAATGAAGAAGAAACATCCTTCTTGGCCATGG<br>AAACTTATTGAGAATGCTTCTAGGAAAGAGTGGGAAGAAACAGCAA<br>TGCAATGGATGTTGAAAACGATTCAACTTACAAAGAAAATGCGTCCT<br>AAAGGAAGATGGTGCTTTTATCATTTGCCTGATTGTTACAATTATGCA<br>GGAAAAGATAATCCAGAACAGTTCCTCTGCAGTTCATTGGTTAGAAA<br>ACACAATGACAGATTGATTTGGCTGTGGAATTCAACAACGGCTCTTT<br>GCCCATCAATATACTATGACGAACGTCAAATGAAATACAACGAAACT<br>CAAGACGTTTGGTTCCTTTATGGCAGACTGTGTGAAGTTATGCGAGT<br>TTCGCAACCAAACACACCTATATACCCTTACATCAATTACAAAGTAC<br>ACACTTCTTTGGAAAATGTACCAAAGGAACATTTCTGGCTAATGTTG<br>TCTCATTTAGCGTCACTTGGCTTAGATGGAGCTGTTATTTGGGGATCT<br>TCCAATTACGTTAAAACAGAAGAGGATTGCAAAAGTCTTGAACTCTA<br>TGTGAAAGAAGTGATTGGGCCTGCTGCATATACCATTTCTACCAATG<br>TCAACTACTGCAGTGAAACAGTGTGCAACAATAATGGACTGTGCACA<br>TGGCCTCATCAGCCTTATACTTCCTGGAAATACCTGACTGACCCAAA<br>CATAACAGTATTCAATCCAGAAAATATCACGTGCAGGTGTCAATTGT<br>ATAAAGGACGCTATTGTGATATGTTTGAATCAAATTCAACTGATTTA<br>CTGTAA

Serin proteases

ID	ID/Reference protein	e-value	PFAM domain	MW (Da)	Sub-classification	Amino acid sequence	Nucleotide sequence
TatEnzSeP01	AFS65328.1	6 e-156	PF00089	--	Trypsin-like	SENLKMCFKMNFSFVTIPLLLFILSRASAMSRHRRQNFIF<br>PSEGENCRTPNEQPGFCIPLSRCEALRRSNDFNLLRNSIC<br>GYDRDVPRVCCPSTNRVVTENPVVVDVSTSNPVPIEVTT<br>RRTVPDGPVIVEVTPRPRTQSTNNNTTRQKPAILPDDCG<br>RSTVSLPKIVNGIPAELAAWPWMAAVYVTRSGLTRGTD<br>CGGALISPRHVMTAAHCVTDRRGNQMSASTFTVRLGEH<br>ILNDDNDGASPVDIPVVRIVRHENFQRRVFRNDIAILTLQ<br>RDVEFNRFIRPICLPYDRFSNDDLAPRGAFVAGWGTTVF<br>DGEYSPILSQIQVPIWRNEDCHRVYQAERVPITREYLCA<br>GVSDGSKDSCQGDSGGPLMLPSEENKFYLVGIVSFGKR<br>CATVGYPGVYTRVNMFLDWIAANLT	TCAGAAAATTTAAAAATGTGTTTCAAAATGAACTTCAGTTTCGTTAC<br>CATTCCACTACTACTGTTTATTTTAAGTCGAGCTTCAGCCATGTCAAG<br>ACATCGAAGGCAAAATTTCATTTTTCCTAGTGAGGGTGAAAATTGTA<br>GAACTCCAAATGAGCAACCAGGATTTTGTATTCCACTTAGCAGGTGT<br>GAAGCATTAAGAAGAAGTAACGATTTTAATCTACTCAGGAACTCAAT<br>TTGTGGATACGACAGAGACGTGCCAAGGGTATGTTGTCCTTCTACAA<br>ATCGCGTTGTTACTGAAAATCCTGTTGTAGTTGATGTGTCAACTTCCA<br>ATCCAGTACCTATTGAGGTAACAACTAGACGAACTGTTCCTGATGGT<br>CCAGTAATTGTTGAAGTAACTCCTAGACCTAGAACTCAATCTACAAA<br>TAATAATACTACAAGACAAAAACCAGCTATTCTTCCGGATGACTGTG<br>GACGTTCAACCGTATCACTTCCAAAAATTGTCAATGGTATACCAGCA<br>GAACTTGCAGCCTGGCCTTGGATGGCAGCTGTATATGTTACAAGAAG<br>TGGACTCACTAGAGGTACTGACTGTGGAGGTGCATTAATCAGCCCTC<br>GTCATGTAATGACAGCTGCGCACTGTGTTACAGATCGCCGTGGAAAT<br>CAGATGAGTGCATCTACATTTACAGTCCGTCTAGGAGAACATATTTT<br>GAATGATGATAATGATGGAGCTTCACCTGTAGACATCCCTGTTGTCA<br>GAATAGTTCGCCATGAGAACTTTCAAAGACGGGTTTTCAGAAACGAT<br>ATTGCAATATTAACGCTACAGAGAGATGTAGAGTTTAACAGGTTCAT<br>ACGTCCAATCTGCCTACCATATGATAGATTCAGTAATGATGACTTAG<br>CTCCAAGAGGTGCATTCGTTGCTGGATGGGGAACCACAGTTTTTGAT<br>GGAGAATACAGTCCAATACTGTCACAGATACAGGTTCCAATATGGAG<br>AAATGAAGATTGTCATCGTGTCTACCAAGCAGAAAGGGTACCAATCA<br>CTCGAGAGTATCTATGTGCAGGTGTAAGTGATGGGAGTAAAGACTCA<br>TGTCAGGGTGACTCTGGAGGTCCTTTGATGTTACCCTCTGAAGAGAA<br>TAAATTTTACTTGGTGGGTATAGTTTCTTTTGGAAAACGTTGTGCAAC<br>AGTTGGATACCCAGGAGTATATACCAGAGTTAATATGTTCTTAGATT<br>GGATAGCAGCAAATCTGACA

TatEnzSeP02	JAT91153.1	3 e-94	PF00089	--	Serin protease	SRIVGGEESSPGSWPWLAALHGGPEEVFFCGGILISPWWVLSAAHCVGNQTNPSGWTMKLGMTRRTSAPFFVHKRGVSLIIKHPDFLSNPFFDDDLVLLMLDKPVNFDEFLRPICLPTLNLSLEGYDSCVVTGWGKAMYDDEADYQVVIHQVKVPIVEFEDCAKWYSLHQVNISEKMICAGYLEGEKDACQGDSGG	TCGAGGATAGTGGGTGGGGAGGAATCATCTCCTGGTAGCTGGCCTTGGTTAGCTGCACTTCATGGTGGTCCAGAGGAAGTCTTCTTTTGTGGAGGAATATTAATTTCACCTTGGTGGGTGCTATCTGCTGCACATTGTGTGGGAAATCAAACAAATCCAAGCGGATGGACCATGAAACTTGGAATGACCAGAAGAACTTCTGCACCCTTCTTCGTTCACAAGAGAGGAGTTTCTCTAATTATCAAACATCCTGATTTTCTCTCAAACCCATTCTTTGATGACGATTTGGTTTTGTTAATGTTGGATAAGCCAGTTAACTTTGACGAGTTCTTGAGGCCCATATGTCTACCAACTTTGAACTTATCTCTCGAAGGCTACGATTCCTGTGTTGTGACTGGTTGGGGAAAAGCTATGTATGATGATGAAGCAGATTATCAAGTTGTCATTCATCAAGTAAAAGTGCCAATTGTTGAATTCGAAGATTGTGCTAAGTGGTATTCCTTACATCAAGTGAATATATCAGAGAAGATGATTTGTGCTGGATATCTTGAAGGTGAAAAAGATGCCTGCCAGGGTGATAGCGGAGGG
TatEnzSeP03	XP_015906323.1	1 e-55	PF00089	--	Trypsin-like	AAHCVYRTATSGLRVRVGEHNIKQTSEPYPHEEYPVRRKIVNPGYHPATYKDDIALLELSHPVIYRKHVIPACLPEKGEDFAGNVASVTGWGRLQYGNRNAPSILQRVEVKVFNLDECREMYKKIGRRETIYSTM	GCAGCTCATTGTGTATATAGAACCGCCACTTCTGGTCTTCGAGTAAGAGTGGGAGAACATAACATAAAGCAAACTAGCGAACCATATCCTCATGAAGAGTATCCGGTGAGGCGAAAGATTGTTAATCCAGGTTATCATCCAGCAACATACAAGGACGATATCGCGTTACTTGAACTCAGCCATCCGGTGATTTACAGGAAGCACGTTATACCTGCTTGTTTGCCAGAAAAAGGAGAGGACTTTGCTGGTAATGTAGCGTCTGTTACTGGATGGGGTCGTTTACAGTATGGTAACAGAAATGCACCTAGCATCTTACAAAGAGTTGAAGTGAAGGTCTTCAACCTCGATGAGTGCAGGGAGATGTACAAGAAGATTGGAAGGCGTGAAACTATATATTCTACAATG
TatEnzSeP04	JAT91142.1	9 e-68	PF00089	--	Serin protease	LTAAHCFGENVGRFPSNYQVRAGGHTRTDGEYYAVEEIRKHPSYTSGRHYYDIAILKVNETFIFGDTVSPACLPSVDESYSNSQVAAFGWGDTSFGGIPSDALQVIIINVLSNFRCDQSYRTLSLSSFPSGITDDLLCAGLSQGGKDTCQGDSGGPLMIKDNSTSKWTVIGVVSFGYQCAVAGYPGVYTRVSSYLPWISENMIN	CTTACAGCGGCTCATTGTTTTGGCGAAAATGTAGGAAGATTCCCTTCCAATTACCAGGTAAGGGCAGGAGGTCATACGAGAACAGATGGAGAGTACTATGCTGTTGAAGAAATCAGGAAACATCCTTCATACACATCAGGACGACATTATTACGATATTGCCATATTGAAAGTGAATGAAACTTTTATTTTTGGTGATACAGTTTCCCCTGCTTGCTTACCTTCAGTGGACGAAAGTTACAGCAATAGTCAAGTAGCTGCGTTTGGATGGGGAGATACCTCCTTCGGTGGAATCCCAAGCGACGCCCTCCAAGTAATAATTATCAATGTTTTGAGTAATTTTCGTTGCGACCAATCTTACAGAACACTGAGCCTGTCTTCTTTTCCAAGCGGGATTACAGATGATTTGCTTTGTGCTGGGTTAAGTCAAGGTGGTAAAGATACTTGTCAGGGAGATTCCGGAGGACCTCTTATGATTAAGGATAATTCAACTTCGAAATGGACAGTAATCGGTGTTGTATCATTTGGATATCAGTGTGCTGTTGCTGGATACCCAGGAGTATATACCAGGGTCTCCAGCTACCTTCCTTGGATTTCTGAAAATATGATAAAC

TatEnzSeP05	XP_015907406.1	0	PF00089	--	Trypsin-like	ASAKTLPAIFAFTIVIAYVILVVKASSLQRISENQDKLKLL<br>KTLDSYNKNILQRQKRETECSANYTVGSKGGNLISPGYP<br>KQYPESLHCQWRLKSSTSSNLILKFTDVSIEEAEDCGYD<br>YLALYDETNNMVEKICGTHSNKVVVLNSTTANIVFHSD<br>ETYTEKGFALEYFLEVTLDCKAILNDSEGTVTSPLYPDN<br>YPGNTDCHTTIMVEPGAKISLKFDVLNLEFDENCDYDY<br>VDIYDGETTDSPLLGHFCSNPEDAVVMSTNNTLMVHFH<br>SDPLINYNGFSASYTSVFPGIQEIGNCVWEKGKNDGTITS<br>PNYPERYPSNSNCRIKIQAAEGYVISIDIDSIKLEVDANCT<br>YDRLEIRDGLNEDSPLLGYFCGQTNEMKHITSAQNGAYI<br>IFVSDSFTEFDGFRIEYRIHRKESEIVSSEDGTVSPSTEIILP<br>SQLFREQPQDFTAIEGEPHVMSCKPEDPNIEVQWTKDGL<br>EIDLFLNIDLHDHGKILWIHSMSEELKGTYTCTAINPHGD<br>SYSVHAILDMKEREWNCDIIFRHTPKNTSILEGDTKLIEC<br>SLSSSKSAQITWTKNGLPLAKSNNYQQIPNGFLLITDATL<br>EMSGIYSCVAEEKSGCVRQHSASIQVSPRISIMNICGRPV<br>MGKPSKRKPQMSHGRILGGKNAKKGAYPWQVIIRNFK<br>HQTACGGTLVNEHWVVTAAHCLYIPKTNHRVEASAFT<br>VKLGEYDVEKKEPEEVLTGMEEYFVHPEFHRATFDNDI<br>AMVKLLDSIQFTDYISPVCLGDHRFIRGVLFEKDLPMGT<br>VTGWGRITESGPTPQFLQELRMPVVDFETCQQSTNYKV<br>TENMFCAGYAQETARDACQGDSGGPFVMEHDDVWYL<br>VGIVSWGEGCGKKGQYGFYTKVDNYHSWIKSIIN	GCAAGTGCCAAAACACTGCCAGCAATTTTCGCTTTTACTATCGTAATT<br>GCCTACGTTATACTGGTAGTTAAAGCTTCGTCGTTGCAAAGGATTTC<br>AGAAAATCAAGATAAGTTAAAATTATTGAAGACCTTGGATAGTTACA<br>ACAAAAATATACTACAACGACAAAAAAGAGAAACAGAATGCTCCGC<br>CAACTATACTGTAGGTTCTAAAGGAGGAAATTTGATCAGTCCTGGGT<br>ATCCAAAACAATATCCTGAAAGTCTGCATTGTCAATGGCGACTGAAA<br>TCCTCAACATCGTCAAATTTAATACTGAAGTTCACTGATGTGAGTAT<br>AGAAGAAGCAGAAGATTGCGGATATGACTATCTGGCTCTCTACGATG<br>AAACAAATAATATGGTTGAGAAAATATGCGGAACCCACAGTAACAA<br>AGTTGTTGTTTTAAACTCAACAACTGCAAATATTGTCTTTCATTCTGA<br>TGAAACTTACACAGAAAAAGGATTTGCTTTGGAATACTTTCTAGAAG<br>TGACATTGGATTGTAAAGCAATTCTGAATGATAGTGAAGGAACTGTA<br>ACAAGCCCACTTTACCCAGATAACTATCCTGGAAACACAGATTGCCA<br>TACAACTATCATGGTTGAACCTGGTGCTAAGATAAGTCTGAAGTTTG<br>ATGTCCTCAACCTAGAGTTTGATGAAAACTGCGATTATGATTATGTA<br>GATATATATGATGGTGAAACAACAGATTCTCCCCTGTTAGGCCATTT<br>CTGTTCAAATCCAGAAGATGCTGTGGTGATGTCAACCAATAATACTC<br>TGATGGTTCATTTTCATTCTGACCCTTTGATAAACTATAATGGTTTCA<br>GTGCATCATATACATCCGTTTTCCCTGGGATACAAGAAATAGGAAAT<br>TGCGTATGGGAAAAAGGAAAGAATGATGGCACAATCACTAGCCCTA<br>ACTATCCAGAAAGATATCCAAGCAACTCTAATTGCAGAATAAAAATC<br>CAAGCAGCTGAAGGTTATGTTATAAGCATTGATATTGATAGTATAAA<br>GCTCGAGGTTGATGCAAACTGCACCTATGACAGACTGGAAATAAGA<br>GATGGGCTAAATGAAGATTCACCATTACTAGGATACTTCTGTGGACA<br>AACAAACGAAATGAAACACATCACATCAGCACAAAATGGTGCCTAC<br>ATTATTTTCGTCAGTGATAGTTTTACAGAATTCGATGGATTTCGTATT<br>GAGTATCGTATACATCGAAAAGAAAGTGAAATTGTATCTTCAGAAGA<br>TGGCACTGTATCTCCCTCTACTGAGATTATTTTGCCATCGCAGTTATT<br>CAGGGAACAACCACAAGATTTTACTGCTATAGAAGGAGAACCTCAC<br>GTTATGTCATGCAAACCAGAAGATCCTAACATCGAAGTGCAATGGAC<br>AAAAGACGGGTTAGAAATAGATTTGTTTCTAAATATCGATTTGCATG<br>ACCATGGAAAAATATTGTGGATACATTCTATGTCAGAAGAATTAAAA<br>GGCACTTACACATGTACAGCAATCAACCCACATGGTGATTCATATTC<br>AGTCCATGCAATACTTGACATGAAAGAACGAGAATGGAACTGTGAT<br>ATAATTTTCAGGCATACTCCAAAGAATACCTCAATCTTAGAAGGAGA<br>TACCAAGCTCATTGAATGCTCATTGTCAAGCTCAAAATCAGCACAGA<br>TAACATGGACGAAAAACGGTCTTCCACTTGCAAAGAGCAACAATTAT<br>CAGCAGATACCAAATGGATTTCTTCTGATAACAGATGCAACTTTGGA<br>AATGAGTGGGATTTATTCTTGCGTGGCTGAAGAAAAAAGTGGATGTG<br>TGAGACAGCACTCTGCATCGATACAAGTTTCCCCTAGGATCAGTATA<br>ATGAATATTTGTGGCCGCCCTGTAATGGGAAAACCAAGCAAAAGAA<br>AACCACAAATGTCTCATGGAAGGATTTTAGGTGGTAAAAATGCCAAG<br>AAAGGAGCATATCCATGGCAGGTGATCATAAGAAACTTCAAACATC<br>AAACGGCTTGTGGAGGCACACTAGTAAATGAGCATTGGGTGGTCAC<br>AGCTGCACATTGTCTTTATATTCCCAAAACTAATCATCGAGTAGAGG<br>CAAGTGCATTCACTGTCAAACTGGGAGAGTATGATGTGGAAAAGAA<br>GGAACCTGAAGAAGTTTTAACTGGGATGGAGGAATATTTTGTTCATC<br>CAGAATTTCATAGAGCTACATTTGACAATGATATTGCTATGGTCAAA<br>CTGCTGGACAGCATTCAGTTTACTGACTATATTTCTCCAGTATGCCTA<br>GGGGACCACAGATTCATACGAGGTGTACTTTTTGAGAAGGATTTACC<br>CATGGGGACTGTAACAGGTTGGGGAAGGATAACAGAGTCTGGACCT<br>ACTCCACAGTTTCTACAGGAATTACGAATGCCTGTGGTGGACTTTGA<br>GACCTGCCAGCAGTCTACAAATTATAAGGTAACTGAAAATATGTTCT<br>GTGCTGGATATGCTCAAGAAACTGCTCGTGATGCTTGCCAAGGTGAC<br>AGTGGTGGACCATTTGTTATGGAGCATGATGATGTTTGGTATCTAGTT<br>GGTATTGTAAGTTGGGGTGAAGGATGTGGAAAAAAGGGACAGTATG<br>GTTTCTATACAAAAGTAGACAATTATCACTCATGGATTAAATCAATT<br>ATTAAC

TatEnzSeP06	JAT91140.1	4 e-120	PF00089	--	Trypsin-like	TVRFERVNYRMIVSLMLLFCGNVALSSWVDDYNNDPRP<br>ICITDFDTNPLNSRIVGGSDAGAGTFPYAVAILMGSPGDS<br>KFRTDNSFCGGTMITESHVLTAAHCLYKNQYLADYLWL<br>NVNDYDTRDSTETRNHVSRVKSITIHPRYVDETFDNDIAI<br>IELSNPVSRNEKDIRMAILPPMGRTLDIGIVCTVWGWGR<br>TSYKGRRPTVMQKVDLPITSREACQEHLSHIITENMICA<br>GGKEGEDACLGDSGGSLLSRDGNVYCLAGIVSFGKGCA<br>MKDVSGVYTNVARYIDWIYENTRTAECKPRILQ	ACTGTTAGGTTTGAAAGAGTGAATTATAGAATGATAGTGTCATTAAT<br>GTTATTGTTTTGCGGCAATGTTGCATTAAGCTCGTGGGTTGACGATTA<br>TAATAACGATCCAAGACCTATCTGTATAACAGATTTCGATACAAATC<br>CCTTGAATAGCCGAATTGTTGGAGGAAGCGACGCTGGTGCTGGAACC<br>TTTCCTTATGCGGTTGCAATACTAATGGGTAGCCCGGGAGATAGTAA<br>ATTTAGAACAGACAATTCGTTCTGTGGAGGAACGATGATCACTGAAT<br>CTCACGTGCTGACAGCTGCTCATTGCTTGTACAAAAATCAATATTTA<br>GCTGATTATTTATGGCTGAATGTGAACGATTATGATACCCGTGATTCT<br>ACCGAAACTAGAAATCATGTGAGCCGAGTTAAAAGTATTACTATACA<br>TCCTAGATATGTGGATGAGACCTTCGATAATGACATCGCAATAATTG<br>AACTAAGTAATCCGGTATCACGCAACGAAAAGGATATAAGGATGGC<br>AATTTTACCTCCTATGGGCAGGACTTTGGATATAGGAATTGTATGTA<br>CAGTATGGGGATGGGGAAGAACTTCTTATAAGGGAAGAAGACCTAC<br>AGTAATGCAAAAAGTAGATCTTCCAATTACGAGTAGGGAGGCGTGC<br>CAAGAACATCTCTCACATATTATTACCGAAAATATGATATGTGCTGG<br>AGGAAAAGAAGGGGAGGACGCTTGCTTGGGTGATTCTGGTGGATCT<br>CTTCTTTCTAGAGATGGAAATGTTTATTGCTTGGCTGGAATTGTGTCC<br>TTTGGCAAAGGATGTGCAATGAAAGATGTATCAGGTGTATATACAAA<br>TGTTGCTAGATATATTGACTGGATTTATGAAAACACAAGGACAGCAG<br>AGTGTAAACCTCGAATACTCCAAT
TatEnzSeP07	JAT91142.1	1 e-83	PF00089	--	Serin protease	RNSWPWMVEIEKKVGPFVQHLCGGCIIGDRYILTAAHC<br>FSQNAERSPSNYTVRTGGHTRTDGVRYTVEEIRRHPSYK<br>AGIHYHDIAILKLKEVIQFYDKVSPACLPSNGDDYVNKR<br>VAVIGWGDTSFGGIPSNVLQEVYINVVNNSLCDQSYRM<br>LSLSSFPRGITQDLLCAGVSKGGKDACQGDSGGPLMIPD<br>FSSLRWEVIGVVSFGYQCAVEGYPGIYTRISSYLPWIAEN<br>MKQ	CGTAATTCCTGGCCATGGATGGTGGAAATTGAGAAGAAGGTTGGCCC<br>ATTTGTTCAGCATCTATGTGGAGGTTGCATTATCGGTGATCGTTATAT<br>TCTTACAGCGGCACACTGTTTTAGCCAAAACGCAGAGAGGTCCCCTT<br>CTAATTATACCGTAAGGACGGGAGGACACACAAGAACAGATGGAGT<br>GAGATATACAGTTGAGGAAATCAGGAGGCATCCTTCGTATAAAGCA<br>GGAATACATTATCATGACATTGCGATATTGAAATTGAAGGAAGTCAT<br>TCAGTTCTACGATAAAGTCTCTCCCGCTTGTTTGCCATCCAATGGTGA<br>TGATTACGTCAATAAAAGAGTGGCTGTCATTGGATGGGGAGATACCT<br>CTTTCGGTGGCATTCCAAGTAATGTTCTTCAAGAAGTTTATATCAACG<br>TGGTCAATAATTCTCTTTGCGATCAATCCTATAGGATGCTAAGTCTGT<br>CGTCATTTCCAAGAGGAATTACTCAGGATTTGCTTTGTGCTGGAGTA<br>AGTAAAGGCGGTAAAGATGCTTGTCAGGGAGATTCTGGAGGTCCCCT<br>GATGATTCCAGATTTCTCTTCATTAAGATGGGAGGTGATTGGGGTGG<br>TGTCATTTGGATATCAATGCGCTGTTGAGGGATACCCAGGAATTTAT<br>ACAAGGATCTCCAGCTACCTTCCTTGGATCGCTGAAAATATGAAGCA

TatEnzSeP08	JAT91148.1	0	PF00089	--	Serin protease	CFQEINFRSLRNRTLPISMKVIFSSMSVSLFPPSTLFTGIPG<br>TDVVSLARLEDWTNSFIKCDRPHHKLDSWWCVWDTDT<br>MRVIFALLVTVCFVSCQSPKEADYERPPKPVKPIGDYDG<br>GDVGGPPPKINDYDEDGKVPLPNPGLKPDVPSDFNDPAP<br>GGGGFDNKPSGDENCVCVPYYQCKEGEIVTDGTGIIDA<br>RKKPPPEEELPLDSQFEPPFCGSFHVCCKTPQQDVVKPY<br>EHKCGIRNPGGIFGKILAPDKKGEANFGEWPWQAAVLK<br>VEGKVNIFQCGGVLVDKKHIATVAHCVCHYKGYNQYP<br>LKVRLGEYDTQKTDEFLAHDDYNVERIFCHPQFRNNSL<br>WNDMALLKLDRDVIFAPHIDSICLPTYEEVFEGQSCVVT<br>GWGKDAYKGGSYSNIMKEVNIPVIDNAKCQEQLRKTRL<br>GRYYRLHDSFICAGGEEGLDSCKGDGGGPLVCYRKDGS<br>YALAGLVSWGIDCGQPGVPGVYVRVQKFLEWVTQQTG<br>LQLNEYWPKVY	TGTTTCCAAGAAATCAATTTTCGCAGTTTGCGAAATCGTACGCTTCCT<br>ATTTCTATGAAGGTTATCTTTTCGTCCATGTCAGTCAGTCTCTTCCCC<br>CCCTCCACTCTCTTCACAGGAATTCCCGGGACTGACGTAGTATCATTG<br>GCTAGATTGGAAGACTGGACAAACAGCTTTATAAAATGTGACCGTCC<br>TCATCATAAACTAGACAGTTGGTGGTGCGTTTGGGATACAGATACAA<br>TGAGAGTCATCTTTGCATTATTAGTGACAGTTTGCTTCGTTTCGTGTC<br>AGTCACCTAAGGAAGCTGATTATGAACGACCACCAAAACCTGTGAA<br>ACCGATTGGCGATTATGATGGTGGTGATGTGGGAGGTCCACCACCTA<br>AGATTAACGATTATGACGAAGATGGAAAAGTACCTCTACCAAATCCC<br>GGACTAAAACCAGATGTACCCAGTGACTTTAACGATCCCGCCCCGGG<br>AGGAGGGGGGTTTGATAATAAACCTAGTGGAGATGAAAATTGCGTC<br>TGTGTTCCTTATTATCAGTGTAAAGAGGGAGAAATTGTTACCGACGG<br>AACTGGTATCATTGATGCCCGTAAAAAACCACCCCCAGAGGAAGAA<br>CTCCCGCTGGATAGTCAGTTTGAACCTCCATTTTGTGGTAGCTTCCAC<br>GTCTGTTGTAAAACCCCACAACAGGACGTTGTAAAGCCATATGAACA<br>TAAGTGCGGTATACGCAATCCCGGTGGAATATTTGGCAAAATTCTAG<br>CTCCAGACAAGAAGGGAGAAGCTAATTTTGGTGAATGGCCTTGGCA<br>GGCAGCTGTATTGAAAGTTGAAGGAAAAGTTAACATATTCCAGTGTG<br>GTGGCGTACTAGTTGACAAGAAACACATAGCAACTGTAGCTCATTGC<br>GTCTGCCATTACAAAGGTTACAACCAGTATCCATTGAAAGTTCGACT<br>TGGTGAATATGACACTCAGAAAACAGACGAATTTCTTGCCCATGATG<br>ATTACAACGTAGAAAGGATATTCTGCCACCCCCAATTCCGAAATAAT<br>AGTTTATGGAATGATATGGCCCTTTTGAAGCTTGACCGTGATGTTATA<br>TTTGCACCCCATATTGACTCTATCTGCTTACCAACTTATGAGGAAGTC<br>TTCGAAGGTCAAAGTTGTGTTGTTACTGGCTGGGGAAAAGACGCTTA<br>TAAAGGAGGCTCATATTCAAATATCATGAAAGAAGTCAACATTCCAG<br>TGATTGACAATGCTAAGTGTCAAGAACAACTGAGAAAAACGAGATT<br>GGGCAGATATTATAGGCTTCACGATAGCTTCATATGCGCAGGAGGTG<br>AAGAGGGCCTTGACTCTTGCAAGGGAGATGGAGGAGGACCCTTGGT<br>ATGCTACAGAAAAGATGGAAGCTATGCCTTAGCTGGTTTAGTATCAT<br>GGGGTATAGATTGTGGACAGCCAGGTGTACCAGGTGTATACGTCAGA<br>GTTCAGAAATTTTTGGAATGGGTTACACAGCAGACAGGATTACAGTT<br>GAATGAATATTGGCCAAAAGTGTAT
TatEnzSeP09	XP_002411811.1	7 e-43	PF00089	--	Trypsin-like	EGKTMSKCFFENQQCGRTVPAREIDEVLYRNDSTDLDR<br>GSERLEINTDGTFVIQPRILIRERFRVLSGEWPWMIAIYLK<br>SNGSFVCNGFIIDNSHLLTAAHCFHEKRRQLQDYYIGTIH<br>NEEILNVEVTELHIHHHYKDNYYKDIALLKMSPRLTDV<br>ASICMPSSVNESNYLLGKEITVLDWVGTEYVMQRNRIA<br>ETQLCNQLYTNLTNSPFPNGIVDELICAIPVEKTDNVCLV<br>HSGGPVMIEENGKWIAIGIATFGIPCTLPTYPAVYTRISPY<br>LQWIRDNE	GAGGGAAAAACCATGTCCAAGTGTTTCTTTGAAAACCAACAATGTGG<br>AAGAACTGTCCCTGCAAGAGAAATTGATGAAGTTCTCTACAGAAACG<br>ACTCTACAGATCTCGATAGAGGATCGGAACGGCTAGAAATCAACACT<br>GATGGAACCTTCGTTATACAACCAAGAATCCTTATTCGTGAAAGGTT<br>TCGAGTTTTGTCTGGTGAATGGCCTTGGATGATAGCAATCTATTTGAA<br>ATCAAATGGAAGTTTCGTCTGCAATGGCTTTATCATCGATAATTCTCA<br>TCTGTTAACAGCAGCTCATTGTTTCCATGAAAAAAGAAGGCAATTGC<br>AAGATTACTACATCGGTACGATACATAACGAGGAAATACTGAATGTA<br>GAAGTGACCGAATTGCACATTCATCATCATTACAAAGATAATTATTA<br>CAAGGATATTGCGTTGCTAAAGATGTCTCCACGTTTAACGGATGTTG<br>CTTCGATTTGCATGCCATCTAGTGTCAACGAAAGCAACTACCTTTTAG<br>GAAAAGAAATAACCGTACTGGATTGGGTCGGAACAGAGTATGTCAT<br>GCAACGAAACCGGATAGCAGAAACACAATTGTGCAATCAGCTTTAT<br>ACAAATTTAACTAATTCGCCGTTTCCTAATGGTATCGTCGATGAACTA<br>ATATGTGCCATTCCTGTAGAAAAAACTGACAATGTCTGTCTGGTTCA<br>TTCGGGTGGACCAGTGATGATTGAAGAAAATGGAAAATGGATTGCT<br>ATAGGAATAGCAACGTTCGGGATACCTTGTACGCTTCCCACCTATCC<br>GGCTGTATATACTCGAATATCGCCTTATCTACAGTGGATCAGAGATA<br>ATGAA

TatEnzSeP10	AFS65328.1	5 e-173	PF00089	--	Trypsin-like	KRGVVINMKYILAIIVVLFLVLIQAARETRKRRQIYFPDD<br>EEEEGCRTPNERRGSCVPLNRCPALRRASIPYLRESICRY<br>NRNTPLVCCPSSRPQEVTPRPVRPTRPTRPTRPTRPTRPP<br>APPPTPRPRNPIVGPNSRKPSILPTECGKSTVPVSRIIGGR<br>KSEVGAWPWMAAVYLTRTGLSRGTDCGGALVSNRHIIT<br>AAHCVVDTRRGTVMSPSSFTVRLGEHTLNDDNDGASPI<br>DFAVSNIVAHEDFERRTFKNDIAILTLRDRVQFNSFIRPIC<br>LPYDSVSQQNLVSRSAFVVGWGTTAFDGNFNPVMSEIQI<br>PIWENDECRRIYQREVPITREYLCAGVSDGSKDSCQGDS<br>GGPLMLPTSDTRFFLVGVVSFGKRCATPGYPGVYTRVT<br>MYLDWLAENLN	AAGCGCGGTGTTGTGATCAATATGAAGTATATTCTTGCCATTATTGTC<br>GTATTATTTCTAGTCCTAATCCAAGCTGCCAGAGAAACAAGAAAACG<br>CAGACAGATATATTTTCCTGATGACGAGGAAGAAGAAGGATGTAGA<br>ACTCCAAACGAAAGGAGAGGATCTTGTGTCCCATTAAATAGATGTCC<br>TGCCTTAAGAAGAGCCAGTATTCCGTATCTAAGAGAATCGATATGTC<br>GGTATAATAGAAATACACCGTTAGTGTGCTGTCCATCATCTAGACCC<br>CAAGAAGTCACCCCACGTCCTGTTAGACCGACAAGGCCTACCAGACC<br>AACTAGGCCAACGAGGCCTACTCGACCCCCAGCACCACCTCCCACAC<br>CACGACCAAGGAATCCCATAGTAGGACCTAATAGTCGAAAGCCCTC<br>AATTTTGCCAACAGAATGTGGAAAAAGCACGGTACCTGTTAGTAGAA<br>TCATCGGAGGAAGGAAGTCAGAAGTAGGTGCTTGGCCTTGGATGGC<br>GGCAGTTTATTTGACACGAACTGGTCTGAGCAGAGGAACCGATTGTG<br>GAGGGGCGCTGGTTTCTAACAGACACATAATTACAGCCGCGCACTGT<br>GTTGTTGACACCAGAAGAGGAACAGTAATGAGCCCTTCCAGCTTTAC<br>AGTTAGATTGGGTGAACACACTTTGAATGATGACAATGATGGCGCCT<br>CACCTATCGACTTTGCCGTAAGCAATATTGTAGCTCATGAAGATTTTG<br>AAAGGAGGACATTCAAAAATGATATCGCTATACTGACTCTGAGGGAT<br>AGGGTTCAGTTCAATTCTTTCATTCGTCCAATTTGTTTGCCTTATGAC<br>AGTGTAAGCCAACAAAATTTAGTTTCAAGATCAGCATTCGTTGTTGG<br>TTGGGGCACTACAGCTTTTGATGGTAATTTTAACCCAGTTATGTCAGA<br>AATCCAAATACCAATATGGGAGAATGATGAGTGTAGACGAATATAC<br>CAACGAGAAGTTCCAATTACCAGAGAATACTTATGCGCGGGAGTCA<br>GTGATGGTTCCAAGGATTCCTGTCAGGGTGATTCAGGAGGACCGTTG<br>ATGTTACCTACAAGTGACACTAGGTTTTTCCTTGTAGGCGTTGTGTCA<br>TTTGGAAAGCGCTGCGCAACTCCAGGATACCCAGGTGTTTATACTCG<br>CGTTACTATGTACTTGGACTGGTTAGCAGAAAATTTGAAT
TatEnzSeP11	XP_011185363.1	3 e-27	PF00089	--	Serin protease	LTMLRLLLLVLYFLSAIASPINDDEDSSRIWNGNIATQGQ<br>FPFVASLQLVYPDGYHSHTCTVSLISTKSCLGAAHCVFR<br>KDETVKGPDEFCVIVGSYVQNDKLPCQELKSLVYHEKF<br>DHKDPIYDIALFELSNEIMLNDDIRTVKLAGPSIEVFGSIV<br>TAIGWGRIVADRRSPHRLRYAFFDVVANSVCKDILNPE<br>MKDDICIYSNEYSICPGDSGGPIVLEGSDIEIGLSSYGGKI<br>CGKIPVAVYTDVRKYLDWIKEHAVGEIEIVEISDDNALY<br>HQQS	TTGACCATGCTAAGGCTTCTGTTGCTAGTTCTATATTTCCTCTCCGCC<br>ATAGCATCACCCATTAACGATGATGAAGATAGTTCAAGAATTTGGAA<br>CGGAAATATTGCTACGCAAGGACAATTCCCTTTTGTGGCGTCTCTGC<br>AGTTAGTTTACCCTGATGGGTACCACAGTCATACCTGTACAGTTTCGC<br>TGATTTCAACAAAATCCTGTCTAGGAGCTGCTCACTGTGTCTTCCGGA<br>AAGACGAAACTGTGAAAGGACCTGATGAATTTTGCGTAATCGTTGGT<br>TCTTACGTGCAAAATGATAAACTTCCTTGTCAAGAACTTAAAAGCCT<br>TGTTTACCATGAAAAGTTTGATCATAAGGATCCAATATATGACATTG<br>CTTTGTTTGAGTTGTCAAATGAAATCATGCTTAATGATGACATAAGA<br>ACAGTAAAGTTAGCTGGACCAAGCATAGAAGTTTTTGGAAGCATAGT<br>AACAGCAATTGGTTGGGGAAGAATAGTAGCAGATAGACGTAGCCCA<br>CATCGATTGAGATATGCTTTTTTCGATGTGGTAGCTAATTCCGTGTGC<br>AAAGACATCCTTAATCCCGAAATGAAAGATGACATTTGTATTTACAG<br>CAATGAGTACAGCATTTGCCCGGGTGATTCTGGAGGACCCATCGTTC<br>TTGAGGGCAGCGACATAGAAATTGGTCTTTCATCTTATGGAGGAAAA<br>ATTTGTGGTAAAATACCAGTAGCTGTATATACAGACGTCCGAAAATA<br>TCTCGATTGGATTAAAGAACATGCTGTTGGAGAGATAGAAATCGTTG<br>AGATATCGGATGATAATGCCTTGTATCACCAACAGTCC

TatEnzSeP12	XP_018529593.1	2 e-30	PF00089	--	Serin protease	MTSSNSAFLLLLLVDYHLTSFSKDITDKFEVAETSRIIGG<br>NQAKPKQFPYAVSIQEYNEDFQTYEHICTFSLITTQSVLG<br>VAHCVVGQNNEILDVDDYIGVLGTVDQWNRTNSVTFD<br>EFIVHRGYNIEYFSNDIAIFVLSESVKLGDSIQTIPLIGQGV<br>SFIGKRGTTFGWGFIYPSATSSTRILRYVDVDIVENEECE<br>QYYGFLDKGQFCFGTEGRSGCDGDSGAPIIVRYRKKEV<br>QMGILSFNTDPCARDPSTGTNISSYNSWIRKNVVGKPVFI<br>N	ATGACTTCTTCAAATTCAGCATTTCTCCTTTTGCTATTGGTTGATTATC<br>ATTTGACTTCATTTTCCAAGGATATTACTGACAAATTTGAAGTTGCGG<br>AAACGTCGAGGATCATTGGTGGAAATCAAGCAAAACCTAAGCAGTT<br>CCCATATGCAGTGTCAATTCAAGAATACAATGAAGATTTCCAAACTT<br>ACGAACACATCTGCACTTTTTCCTTGATTACAACTCAATCAGTTCTAG<br>GGGTAGCGCATTGTGTAGTAGGACAGAATAATGAAATTTTAGATGTA<br>GACGATTACATTGGAGTTTTAGGAACTGTGGATCAGTGGAACAGAAC<br>CAACAGTGTAACTTTCGATGAATTCATTGTTCATCGTGGTTATAACAT<br>CGAATACTTTTCGAATGACATTGCAATATTTGTACTTTCCGAATCGGT<br>AAAACTGGGTGATTCTATTCAAACTATACCTTTGATTGGACAAGGTG<br>TCTCGTTTATTGGAAAGCGAGGTACAACTTTCGGATGGGGTTTCATCT<br>ACCCTTCAGCCACGAGTTCAACTCGTATATTGAGATACGTTGATGTT<br>GATATTGTGGAAAATGAGGAATGTGAACAATATTATGGTTTCCTAGA<br>TAAAGGTCAGTTTTGTTTCGGAACAGAAGGAAGAAGTGGTTGCGATG<br>GAGATAGTGGCGCTCCAATCATAGTAAGATATCGGAAGAAGGAAGT<br>ACAAATGGGTATTTTATCATTTAACACAGATCCTTGTGCAAGAGATC<br>CAAGCACGGGTACAAATATCAGTTCTTATAATTCGTGGATTCGAAAG<br>AATGTTGTGGGAAAGCCAGTTTTCATTAAT

TatEnzSeP13	XP_019869370.1	0	PF00089	--	Nudel-like	TLMREHQKTETPRLDNLAFVTTFPKDYLPYARQKRKSDPMESNPHWKYKRKRWWPFRRCCNLSSATTIISITVTILAFMAALGVSIYFGVLTSMYTSPVFHIYGKIRVTEGDEFDSSLLDANSANFASKAAIYENKLRSTYLRSLFQYVLHEVRIYGFREPGLNIFFRLHLDRRKGTRIQGINNLARTVLLNEIHNGEIFEGLKIDPESVSFSGISCLDDCPTQAPTNTTTPLLVSDWFNTIDVTTKKNEQDEAHFKRLNGTNNNETVVQWVTPSNTKITNVTILQIDANSTLLPWKNASEWDNTTVSAKYNDTGVTVSSPKFVSQFTNREENTLFHQENDKQNRNLSVIEDDLIVPVNETTLSSTTDLWNEFDVVNIKDGKNKIPECGKEGFRCADNKCIPGYRKCDRFSDCSDSSDEANCTCSDYLISLEQPRKICDGFMDCWDYSDETHCPWCSDDMFHCPGAKVCINKTKVCDGIRDCPSGSDEATCMKIAKDVSGANGFTFAKEGYLMIQREGQWKKLCLDNNNLIDKPRWNVEKLGYTVCSSLTYKALESIVLSPRKYQEDSYYELIPATERSGTKFLISDCADGKIARIRCRDLACGLGTPVQLPTKRIVGGDSVPMGARPWLAALYREGQFQCGAIVIADRWLLSAGHCFFHTKHSYWVARMGLLRRGTDMPTPYEIVKRIIQIKVNPQYINKNFINDIAVLKLESAVPFSNYIRPICLPQPEDDVVKWNGKKCSVVGWGKLGENGDEFPDTLQEVQLPVISTEECRKRTLFLSIYQITDNMFCAGYERGGRDACLGDSGGPMMCQKEDGRWVLIGIISNGDGCARPSRPGVYTKVGNFMNWIDSIMNENATDEVKPDSCLGTRCKLGMCLFPESNCDGNADCPDGSDEKGC	ACACTGATGAGGGAACACCAGAAGACAGAAACGCCACGCCTGGATAATTTAGCGTTTGTAACTACATTTCCGAAGGATTACCTACCGTACGCTCGACAGAAAAGAAAATCGGATCCGATGGAATCTAATCCTCATTGGAAATATAAGAGGAAACGATGGTGGCCTTTCAGAAGATGTTGCAATCTCTCCTCAGCGACTACAATTATTTCAATAACTGTTACCATTTTAGCATTTATGGCAGCTTTAGGTGTATCGATATATTTTGGCGTTTTAACCAGTATGTACACCTCTCCAGTCTTTCACATATACGGAAAAATACGGGTAACGGAGGGTGACGAATTTGACTCTTCCTTGTTAGATGCCAACTCTGCTAATTTTGCTAGCAAAGCAGCTATTTACGAAAACAAGCTTAGAAGCACGTATCTTAGAAGTTTATTTCAGTACGTATTACACGAAGTCAGGATTTACGGTTTTAGGGAACCTGGCTTGAATATTTTCTTCAGACTTCATTTAGACAGAAGAAAAGGTACCCGGATACAAGGTATTAATAATTTGGCAAGGACTGTTCTTTTAAACGAGATACATAATGGAGAAATATTTGAGGGCTTGAAAATCGATCCTGAATCGGTGTCCTTCTCCGGTATTAGTTGCCTTGACGACTGTCCAACTCAGGCTCCCACTAACACGACCACACCTTTATTAGTATCTGACTGGTTTAATACTATTGATGTCACTACAAAAAAGAATGAGCAAGATGAAGCGCATTTCAAAAGGCTTAACGGTACTAACAACAATGAAACCGTTGTTCAATGGGTCACTCCATCCAATACCAAGATCACTAACGTTACAATTCTCCAAATTGATGCAAACAGTACGTTATTACCCTGGAAAAATGCTTCAGAATGGGATAATACCACAGTAAGTGCCAAATACAATGATACTGGAGTCACTGTCAGTTCACCGAAATTCGTATCGCAATTCACGAACAGAGAAGAGAACACACTCTTTCATCAGGAGAATGATAAGCAGAACCGGAATTTAAGTGTAATTGAAGATGATTTGATTGTACCTGTTAACGAAACTACACTAAGTTCGACCACCGATTTGTGGAATGAGTTTGATGTCGTCAATATTAAAGATGGCAAAAATAAAATTCCAGAATGCGGCAAAGAAGGATTTCGCTGTGCTGACAACAAATGTATTCCTGGTTATCGTAAATGCGATCGCTTCTCGGATTGTTCAGATAGTTCTGATGAAGCGAACTGCACCTGTTCTGACTATTTGATATCGCTCGAACAGCCGAGGAAAATATGCGATGGTTTTATGGACTGCTGGGATTATAGTGATGAAACCCATTGCCCGTGGTGTTCTGATGATATGTTCCATTGTCCTGGTGCAAAAGTGTGTATCAATAAAACTAAAGTATGCGATGGTATTCGAGACTGCCCTTCCGGTTCTGATGAGGCCACATGCATGAAAATAGCCAAGGACGTAAGCGGAGCTAATGGTTTTACTTTTGCCAAGGAAGGTTACTTAATGATTCAGAGAGAGGGTCAATGGAAAAAGTTATGTCTTGACAACAATAATCTAATCGACAAACCTAGATGGAATGTCGAAAAGCTTGGATACACCGTTTGTTCTTCTCTGACTTATAAGGCTTTAGAATCGATTGTTTTATCACCACGGAAGTATCAAGAAGATTCATATTATGAACTGATACCAGCTACAGAAAGGTCTGGCACTAAGTTTCTTATATCCGATTGTGCAGATGGAAAAATCGCCCGAATAAGATGTCGAGACTTGGCGTGTGGATTGGGAACGCCTGTTCAGTTACCTACCAAAAGAATCGTCGGAGGTGACAGCGTACCGATGGGAGCGAGACCATGGCTTGCTGCTCTTTATAGAGAAGGACAGTTCCAATGCGGAGCTATCGTTATTGCGGACAGATGGCTTTTGTCGGCGGGACATTGTTTTTTTCACACCAAGCATTCTTATTGGGTGGCAAGAATGGGCTTGCTTAGAAGAGGGACAGATATGCCAACGCCTTACGAGATAGTGAAGAGGATTATTCAGATTAAAGTCAATCCTCAATACATAAATAAGAACTTCATCAATGATATAGCCGTTCTTAAATTAGAGAGTGCCGTCCCCTTCAGTAATTACATTCGTCCAATCTGCTTACCACAACCAGAAGATGATGTTGTTAAATGGAATGGTAAAAAATGTTCAGTTGTAGGATGGGGAAAATTAGGAGAAAATGGTGATGAATTTCCCGACACTCTTCAAGAAGTGCAGTTACCCGTCATCTCAACCGAAGAATGCAGGAAAAGAACTTTATTTTTATCTATCTATCAAATAACCGATAACATGTTTTGTGCCGGATACGAAAGAGGGGGTCGGGATGCGTGTTTGGGTGACTCGGGTGGTCCCATGATGTGCCAGAAGGAAGATGGTCGTTGGGTGTTAATTGGCATTATAAGCAACGGTGACGGATGTGCGAGACCTTCTCGTCCGGGGGTATATACTAAAGTTGGAAATTTTATGAACTGGATAGATTCTATAATGAATGAAAACGCTACAGACGAAGTAAAACCTGACAGCTGCCTTGGAACTAGGTGTAAGCTAGGCATGTGTCTTTTCCCAGAATCAAACTGTGATGGAAATGCAGATTGCCCGGATGGTTCCGACGAAAAAGGGTGC

TatEnzSeP14	JAT91142.1	7 e-94	PF00089	--	Chymotrypsin-like	VLTPLTKMNYALLMVSFLHFSKYCVLSQNEQSPSKKLS<br>YDDDVMVEGDGCFTQFRATGVCRAITACKTLSKTERQN<br>PKLCSWDRDVPVICCPVPLADVLKPAAITNQECGIRTAV<br>TIITDGRLKRMALEFANRQNKPTVVGGKDVIRNSWPWM<br>VSIRKNTQIHCGGCIIGDRYILTAAHCFDGNNRSPSDFTV<br>RVGGHTRADGVDYSVEEIKKHLLYKAGIRYHDIAILKTK<br>ESIRFHRTVSPACLPSSEDTYVNRTATVIGWGDTFFGGIP<br>SSVLQEASISVVSNSICDQSYRTLSLSSFPEGITDNLLCAG<br>LSEGGKDACQGDSGGPLMIFDLPSLKWMVTGVVSFGY<br>QCAVKGFPGIYTRVSSYLPWISEIMGS	GTGCTAACACCCTTAACGAAAATGAACTACGCTTTGTTAATGGTATC<br>GTTTCTGCATTTTTCTAAATACTGTGTTCTTTCTCAGAATGAACAATC<br>ACCTTCCAAGAAACTTAGCTATGACGATGATGTTATGGTTGAAGGAG<br>ACGGTTGCTTCACTCAGTTCCGTGCAACGGGAGTATGTCGAGCAATA<br>ACAGCCTGCAAAACTCTATCAAAAACGGAGAGACAAAATCCTAAAT<br>TATGTAGCTGGGATAGAGATGTGCCAGTTATATGTTGCCCCGTACCA<br>TTAGCTGACGTACTAAAGCCAGCTGCTATCACAAACCAAGAATGTGG<br>AATAAGAACAGCTGTAACAATAATAACGGATGGCAGACTAAAGAGA<br>ATGGCCCTCGAATTCGCGAACCGTCAGAACAAACCTACCGTAGTTGG<br>CGGTAAGGACGTAATTCGTAATTCCTGGCCATGGATGGTATCCATCC<br>GCAAAAATACCCAAATCCATTGCGGAGGATGCATCATAGGCGACCG<br>TTATATTCTTACAGCTGCTCACTGTTTTGATGGAAATAACAGATCGCC<br>TTCTGATTTTACCGTAAGGGTTGGTGGCCATACTCGGGCTGATGGAG<br>TAGACTATAGTGTGGAAGAAATTAAGAAGCACCTATTATACAAGGC<br>AGGCATACGTTATCACGATATTGCCATACTGAAAACAAAAGAAAGT<br>ATTCGTTTCCATAGGACAGTTTCTCCAGCTTGCTTGCCTTCGTCAGAG<br>GATACTTACGTAAATAGGACAGCCACTGTTATTGGATGGGGAGATAC<br>GTTTTTCGGGGGTATTCCCAGCAGCGTGCTTCAGGAAGCAAGTATTA<br>GTGTGGTCAGCAATTCTATCTGTGACCAGTCTTACAGAACATTAAGC<br>CTATCTTCTTTTCCTGAAGGAATTACAGATAATCTCCTTTGTGCTGGT<br>TTAAGCGAAGGTGGTAAAGACGCCTGTCAGGGTGATTCTGGGGGTCC<br>TCTTATGATTTTCGACTTACCCTCATTGAAATGGATGGTGACTGGTGT<br>AGTATCATTTGGGTATCAATGTGCTGTTAAAGGATTCCCAGGAATTT<br>ACACCAGGGTCTCCAGCTACTTACCTTGGATTTCTGAAATTATGGGT<br>AGC
TatEnzSeP15	XP_013781651.1	1 e-141	PF00089	--	Trypsin-like	GTHADDGVESVACTRAGQRLSGPSGIITTPGFKDSKPYP<br>PRAVCTWLFQLPPMKYISFEFEEFDVELSNDCKTDGVDI<br>FAMNPPKLLRKFCGSEVPKIVILPTNDGIRLKFRSDDLYE<br>RKGAKIKYEILNTYPDCGKDHIRCKNGKCVSKDKKCNK<br>IDDCGDGTDEECSNILEHPKTCGKPKYSPVIGNGDRIIGG<br>QETAPHSWPWQVSIQNAISFPSGHYCGGALINNLWVVT<br>AGHCLYGRDIGSIRIHLGMHNRFYKDDGEIVRYASKSCV<br>HPDFRRQLLKDDIALIKLNAPVAYNDNIQPVCMPPLTES<br>LREGTYCHVTGWGTTFGTGNFHVLKQTMVPIIDHDKCK<br>KMVKLLPLSPNAICSGFEDGYHSPCFGDSGGPFVCDYNK<br>TWHLTGVVSWGPMMCGIREEPSVYTKVADYKPWLERII<br>ENDEKGIQNRCL	GGAACACATGCTGATGATGGCGTGGAGTCTGTGGCTTGTACTCGTGC<br>TGGGCAGAGACTGAGTGGTCCATCAGGTATAATTACAACTCCTGGTT<br>TCAAAGACTCTAAACCATACCCTCCACGAGCAGTATGCACATGGTTG<br>TTCCAGCTACCACCTATGAAATATATTTCTTTTGAATTCGAGGAGTTC<br>GATGTAGAATTGTCAAATGACTGCAAGACTGATGGTGTTGACATATT<br>TGCAATGAATCCACCTAAATTGCTTCGAAAGTTTTGTGGTTCTGAAGT<br>GCCAAAAATCGTTATTTTGCCTACGAATGATGGAATTCGACTGAAGT<br>TTCGCAGCGATGACTTGTATGAGAGGAAGGGTGCAAAAATCAAATA<br>TGAGATCCTAAATACTTATCCTGATTGTGGGAAAGATCACATTAGAT<br>GCAAGAATGGCAAATGTGTCTCCAAAGATAAAAAATGCAATAAAAT<br>TGACGACTGTGGAGACGGTACTGACGAAGAGTGCTCTAACATCTTGG<br>AGCATCCAAAGACCTGTGGCAAGCCAAAATACAGCCCAGTAATAGG<br>AAATGGCGATAGAATAATCGGAGGACAAGAAACTGCACCTCACAGC<br>TGGCCATGGCAAGTCAGTATTCAAAATGCGATATCTTTTCCATCTGG<br>ACATTACTGCGGAGGTGCTCTTATTAACAATCTGTGGGTAGTTACCG<br>CCGGGCATTGCCTTTACGGAAGGGATATTGGAAGTATCAGAATTCAC<br>CTGGGAATGCACAATCGTTTTTATAAAGACGATGGTGAAATTGTTAG<br>GTATGCATCTAAATCGTGTGTCCATCCAGACTTTAGAAGGCAACTAC<br>TGAAGGATGACATTGCCTTGATAAAGCTGAACGCTCCTGTCGCATAT<br>AATGATAATATTCAACCTGTTTGTATGCCTCCACTGACCGAAAGTTTA<br>AGAGAAGGAACGTACTGTCATGTGACAGGATGGGGAACTACGTTTG<br>GGACTGGAAATTTCCATGTTTTGAAGCAAACTATGGTTCCAATTATT<br>GATCATGATAAATGTAAGAAAATGGTAAAACTTCTTCCACTTTCGCC<br>TAATGCCATTTGCTCCGGATTTGAAGATGGTTATCATTCACCCTGCTT<br>TGGTGATAGCGGTGGTCCATTCGTTTGTGATTATAACAAAACATGGC<br>ATTTAACTGGAGTAGTTTCTTGGGGTCCAATGATGTGTGGAATTAGA<br>GAAGAACCTTCTGTTTATACAAAGGTTGCTGATTATAAGCCATGGTT<br>AGAGAGAATTATTGAAAACGATGAGAAGGGTATCCAAAATCGTTGT<br>TTG

TatEnzSeP16	EZA52937.1	5 e-25	PF00089	--	Serin protease	IGAANVRKLFFALLTTLCLLPSIYVDGVEVLSPRIVGGET<br>AMAGEMPCVLAVEDVRLHGNQSYCTFCLINEQTSIGGA<br>LCVYQAKENIKTVGLFIYLIGGDVDRRNGTILPVKDIKIH<br>EEFNYTSMRNDISLITVVKPITITPNLKPVLLPSPDQEFEGI<br>TAKISGWGSTTFDTWYTTRYLRISEVKVLPDSDCNSFEE<br>FYQPEEHLCAGASESGLAAYDSGVPLIISMNDEQIIIGVG<br>SLADTNTTDTGNNAAYTNIKHYIPWIKENALGEVKLFN<br>ASSTF	ATTGGTGCTGCTAACGTCAGAAAATTATTCTTCGCATTGCTGACGAC<br>CTTATGTCTACTTCCTTCGATATATGTGGATGGAGTGGAGGTACTCTC<br>ACCGAGGATTGTGGGAGGAGAAACAGCAATGGCGGGTGAAATGCCT<br>TGTGTGTTGGCCGTGGAAGATGTCAGACTCCATGGTAACCAATCCTA<br>TTGCACATTCTGTCTGATTAACGAACAAACATCCATTGGAGGTGCAC<br>TCTGCGTATATCAAGCTAAAGAAAATATAAAAACAGTAGGTCTGTTC<br>ATCTACCTTATTGGCGGTGATGTGGATAGGCGTAATGGAACCATTTT<br>GCCGGTAAAAGACATAAAAATACACGAAGAATTTAATTATACAAGT<br>ATGAGGAATGACATTTCTCTAATAACAGTTGTGAAGCCTATTACAAT<br>AACACCAAATTTGAAGCCAGTCCTCCTTCCATCGCCAGACCAAGAGT<br>TTGAAGGAATAACTGCCAAAATATCTGGATGGGGAAGCACTACATTT<br>GACACTTGGTATACAACTAGATATCTTCGAATATCAGAAGTTAAAGT<br>CCTTCCTGATTCAGACTGTAATTCTTTTGAAGAATTTTATCAACCAGA<br>GGAGCATCTTTGCGCTGGTGCAAGCGAATCAGGTCTTGCAGCGTATG<br>ATTCTGGTGTACCTCTGATAATATCTATGAATGACGAGCAAATAATT<br>ATTGGGGTTGGATCTCTAGCTGATACCAATACAACGGATACTGGAAA<br>TAATGCAGCATACACAAATATAAAACATTACATCCCTTGGATTAAGG<br>AAAATGCACTGGGTGAAGTCAAGCTTTTTAACGCTTCCAGCACATTC
TatEnzSeP17	XP_015911504.1	1 e-158	PF00089	--	Serin protease	DPYCGRNAVKTSRIVGGNDASFGQFPWQALIQVGGSRC<br>GGALVGRCHVVTAGHCVARSQHNPSNIRVTLGDFVLHS<br>NIESLPTETIGVSQVKLHPNFRFTPQADRFDVAVLILKRA<br>APYRANIKPICLPRKNARFLGRTAYAAGWGAVQPGSKL<br>RPKILQYVGVPIIENKICETWHKKRGINIRIHDEMMCAG<br>YERGGRDACQGDSGGPLMMKEYNVWYLIGIVSAGYSC<br>AKQHQPGIYHRVSSSSDWISSNLF	GATCCTTATTGTGGCAGAAATGCGGTGAAAACTAGTAGAATAGTTGG<br>TGGAAATGATGCGTCCTTTGGACAATTTCCATGGCAGGCTTTAATTC<br>AAGTAGGAGGAAGCAGGTGTGGTGGCGCTCTTGTCGGACGCTGCCAT<br>GTTGTGACTGCTGGTCACTGTGTGGCACGATCGCAGCACAATCCTTC<br>CAATATCCGCGTTACTTTAGGTGACTTCGTTCTCCACTCAAATATCGA<br>AAGTTTGCCAACGGAAACAATTGGAGTCAGCCAGGTGAAGTTGCATC<br>CGAATTTTCGTTTCACCCCACAAGCTGATCGTTTCGACGTGGCCGTGT<br>TGATTTTGAAGAGAGCAGCACCTTATCGTGCTAATATCAAACCCATT<br>TGTCTACCAAGGAAGAATGCTAGATTTTTGGGAAGGACGGCTTACGC<br>TGCTGGATGGGGAGCTGTTCAGCCAGGTTCAAAGTTACGGCCGAAGA<br>TTCTGCAGTACGTAGGCGTCCCTATTATAGAAAATAAAATATGTGAA<br>ACGTGGCACAAAAAGCGTGGGATCAATATTCGAATACATGATGAAA<br>TGATGTGTGCTGGTTATGAAAGGGGAGGAAGGGATGCCTGTCAGGG<br>TGATTCCGGAGGACCACTGATGATGAAAGAATACAATGTGTGGTATT<br>TGATAGGAATAGTTTCTGCTGGTTATTCTTGCGCCAAACAGCACCAA<br>CCTGGAATTTACCACCGAGTTAGCAGTTCCTCAGACTGGATATCGTC<br>TAATCTCTTT
TatEnzSeP18	AAD00320.1	6 e-60	PF00089	--	Serin protease	LVMKTVHIWFLLSISSAAVSVIHSVKCGVSSKRDDGKFR<br>LIVGGKPVPSGEWPWQVSLQVREGQNSFKHFCGGTLLN<br>ENWILTAAHCFGSYGIPERVVLGEHNLKVKEKTEVHRNI<br>AKTVLHPLYDRETISHDVALVKFLEPLDLEYEHNHLVPI<br>CIPDKDTDVDNLSCVATGWGHLTPRGRRPDILQAVVLPI<br>IDQEECKDRYTNVISYPITSDMICAGASEDGKGTCQGDS<br>GGPLQCRMGQQWYQVGITSWGIDCASGLFPDVYARVS<br>SVHEWIRSTINGQ	TTAGTCATGAAGACAGTACATATTTGGTTCCTGCTTTCCATTTCTTCA<br>GCTGCAGTTTCGGTCATCCATAGTGTAAAATGTGGCGTTTCGTCGAA<br>AAGAGATGATGGCAAGTTTAGGTTGATTGTAGGAGGAAAGCCAGTG<br>CCCTCTGGTGAGTGGCCTTGGCAAGTATCTTTGCAAGTCAGAGAAGG<br>GCAAAATAGTTTCAAACATTTCTGTGGAGGGACTCTTTTGAATGAAA<br>ATTGGATTTTAACAGCAGCCCATTGCTTTGGAAGTTATGGTATTCCCG<br>AAAGAGTTGTTCTTGGAGAACATAATTTAAAGGTGAAGGAGAAAAC<br>GGAAGTTCACAGGAACATTGCAAAGACTGTGCTTCATCCTCTATATG<br>ATCGAGAAACTATATCCCATGATGTAGCTTTAGTAAAGTTTTTGGAA<br>CCTTTAGATTTGGAATATGAGCATAATCACTTGGTCCCAATATGTATT<br>CCTGACAAAGACACAGATGTAGACAACTTGTCCTGCGTAGCCACTGG<br>TTGGGGACACCTTACTCCTAGAGGAAGACGTCCAGATATACTGCAGG<br>CAGTAGTCCTGCCAATTATAGATCAGGAAGAGTGCAAAGATCGATAC<br>ACAAATGTAATTTCTTATCCAATCACATCTGATATGATCTGCGCTGGA<br>GCATCTGAAGATGGAAAAGGCACTTGTCAAGGCGATTCTGGTGGACC<br>ATTACAATGTAGAATGGGGCAGCAGTGGTACCAGGTTGGAATTACAT<br>CTTGGGGCATAGATTGTGCATCGGGATTGTTTCCAGATGTGTACGCT<br>CGTGTCTCTAGTGTTCATGAGTGGATTCGTTCAACTATCAATGGTCAA

Metalloproteinases

ID	ID/Reference protein	e-value	PFAM domain	MW (Da)	Sub-classification	Amino acid sequence	Nucleotide sequence

TatEnzMtp01	AMO02512.1	3 e-118	PF00200	--	ADAM-like	SPNARHGGSYLMYTYSVSGYDPNNKKFSPCSIRSVRAV<br>LLAKASKCFSTPEESFCGNSLVEVGEQCDAGLIGSEDND<br>PCCDTNCTFRKDVICSDKNSPCCRDCRYVAAGEKCRDA<br>QTTACKKEAYCSGDSSDCPPSAPQPNKTLCLDRGQCLM<br>GECIPFCETRDRLSCMCDREEDACKRCCRRSQNDTCDP<br>MEPVEILRDG	TCACCTAATGCACGGCATGGTGGTTCCTATCTGATGTATACATACTCT<br>GTTAGTGGTTATGATCCAAATAATAAGAAATTCTCGCCTTGCAGTAT<br>ACGTTCTGTTCGAGCAGTATTGCTAGCTAAAGCGAGCAAATGTTTTT<br>CCACACCAGAAGAGTCATTCTGCGGCAATTCACTAGTTGAAGTTGGA<br>GAACAGTGTGATGCTGGTTTGATTGGAAGTGAAGATAATGATCCTTG<br>TTGTGATACAAATTGTACCTTTAGAAAGGATGTGATATGCAGTGACA<br>AAAATTCACCTTGTTGTCGTGATTGTCGGTATGTAGCAGCAGGGGAG<br>AAATGTAGAGATGCGCAGACAACAGCATGTAAAAAAGAAGCATACT<br>GCAGTGGGGACTCATCTGATTGCCCGCCATCTGCACCTCAGCCTAAC<br>AAAACACTTTGTTTAGATAGAGGGCAATGCTTGATGGGAGAATGCAT<br>TCCTTTCTGTGAGACAAGAGACCGGCTATCATGCATGTGTGACAGAG<br>AGGAAGATGCATGTAAGCGATGTTGTCGCCGTTCACAAAATGACACC<br>TGTGATCCTATGGAGCCTGTAGAAATTCTAAGAGATGGC
TatEnzMtp02	XP_013793082.1	0	PF13574,<br>PF00200	--	Disintegrin-like	MIQNKFFLSLLVCFVTGGETGRLNQYISYFEPLRYNHET<br>VHQGHLRAKRSPSDSLVYYVNFHAHGRHFNIRLKRDTSA<br>FAPDVVIETTNGVAEVDTSHLYDGHLLGESGSTVYGSIR<br>NGVFEGKIHTPNKVYYYVERSHKYFSSNPQPFHSVIYSSD<br>DVEDPYADKRESHAGGCGITEDVLQWMQEVSNSADPK<br>EMSPTNKTTNPEQSHLERSERSTAAENAPHNIYTREAQR<br>DINQIDQLQAKRACTLYVQTDVFLWKHILTYEKTASSA<br>REEISSLISQHVRAVNYIYENTDFDGYRGIKFVVQRIKIN<br>DTSSCSNQGLDYKRTNPFCSPNIDVSNFLNLNSQFNHDD<br>FCLAYIFTYRDFSGGTLGLAWVASASGASGGICEKYKPY<br>NENINGRQVQTKRSLNTGVITFVNYNSRVPPKVSELTLA<br>HEIGHNFGSPHDYPIDCRPGGSDGNFIMYASATSGDRKN<br>NNKFSQCSIRNITTVLRAVFNGEGKENCFQRSDGAFCGN<br>KIVENTEECDCGYDAKECNDECCYPRDVTLFQMKDPNA<br>QRCKLRPRKVCSPSQGPCCSPSCNYVANGTICRGESECT<br>LISLCNGSSAVCPASKPRPNRTECNKGTQVCWNGECTGS<br>ICQKYNMEECFLTSLNGAKPEEMCEVACQKLGQPATCE<br>STYGIAKMKNISGLKLQPGSPCNEFQGYCDVFQKCRAV<br>DAEGPLARLKNLLLNQQTLRTIKQWITTYWWAVMLML<br>VGLAIFMGLFIKCCAVHTPSSNPRKAPALRISDTLRHPAD<br>TLRRKRHRPPPQPGIPSGPPPPYPGPQPSAPPSRSRSSAGP<br>SRGYGEGRGHYNRRDRGQMHHGERGKLYIEDPSYAPA<br>HPGRGSAMEMRVKSQA	ATGATACAAAACAAGTTCTTTTTGTCACTGCTTGTTTGTTTTGTGACA<br>GGAGGTGAAACCGGTCGACTTAATCAGTATATAAGTTATTTTGAGCC<br>ACTGAGGTACAACCACGAAACTGTACATCAAGGTCATTTACGTGCCA<br>AACGTTCACCTTCAGATTCCTTAGTTTATGTGAATTTCCATGCACATG<br>GAAGGCATTTTAATATTCGACTTAAAAGGGACACATCAGCTTTTGCT<br>CCAGATGTTGTTATAGAAACTACTAATGGTGTAGCAGAAGTTGACAC<br>ATCCCACTTATATGATGGGCATTTGTTAGGGGAATCAGGATCCACTG<br>TATATGGCTCAATAAGGAATGGTGTATTTGAGGGAAAGATTCATACA<br>CCGAACAAAGTTTATTATGTAGAACGCTCTCACAAATATTTTTCATCA<br>AACCCTCAACCATTTCACTCTGTGATATACTCATCAGATGATGTGGA<br>AGATCCATATGCTGACAAACGGGAGTCACATGCAGGTGGATGTGGC<br>ATCACAGAGGATGTTTTACAGTGGATGCAAGAAGTTAGCAATTCAGC<br>AGATCCTAAAGAAATGTCCCCTACAAACAAGACAACAAACCCGGAG<br>CAGTCTCATTTGGAAAGAAGTGAACGTTCAACAGCTGCGGAAAATGC<br>ACCTCATAATATCTATACAAGAGAAGCGCAACGTGATATTAATCAGA<br>TAGACCAATTACAAGCAAAAAGAGCATGTACATTATATGTGCAAAC<br>AGATGTATTTCTATGGAAGCATATTTTGACGTATGAAAAAACTGCTA<br>GTAGTGCACGAGAAGAAATATCTTCATTAATATCTCAACATGTGAGA<br>GCCGTTAACTATATTTATGAAAATACTGACTTTGATGGCTATCGGGG<br>CATAAAGTTTGTGGTGCAAAGAATTAAGATAAATGACACATCATCGT<br>GTTCCAATCAAGGGTTGGATTACAAACGAACAAATCCATTCTGCTCG<br>CCAAATATTGATGTATCAAACTTTTTAAATTTGAATTCTCAATTCAAC<br>CATGATGATTTCTGTTTAGCATACATTTTTACGTACAGAGATTTCTCT<br>GGTGGTACATTAGGACTTGCATGGGTTGCATCAGCATCAGGTGCTTC<br>TGGAGGAATCTGTGAGAAATACAAGCCATACAATGAAAATATTAAT<br>GGCAGACAAGTACAAACCAAAAGAAGCCTGAACACTGGTGTGATTA<br>CCTTTGTCAATTACAACAGCCGAGTACCTCCAAAAGTGTCAGAATTG<br>ACATTGGCTCATGAAATTGGACACAACTTTGGTTCACCTCATGATTAT<br>CCTATTGACTGCAGACCTGGTGGTAGTGACGGAAACTTTATTATGTA<br>TGCAAGTGCTACATCTGGGGATAGGAAGAACAATAACAAATTTTCTC<br>AGTGCAGTATCAGAAACATTACTACTGTCCTCCGTGCTGTGTTCAAT<br>GGCGAAGGAAAGGAAAATTGCTTTCAGCGAAGTGATGGTGCTTTCTG<br>TGGTAATAAAATTGTGGAAAATACTGAAGAATGCGACTGTGGGTATG<br>ATGCAAAGGAATGCAATGATGAATGCTGCTATCCACGAGACGTCACT<br>CTTTTCCAAATGAAAGATCCAAATGCACAACGATGCAAACTACGTCC<br>AAGAAAAGTTTGCAGTCCAAGCCAAGGACCATGTTGCTCACCATCTT<br>GCAATTATGTTGCGAATGGCACCATTTGCAGAGGAGAATCTGAATGC<br>ACTCTCATTTCCTTGTGCAATGGAAGCAGTGCAGTGTGCCCTGCATC<br>AAAACCAAGGCCTAATAGAACAGAATGTAATAAAGGAACACAAGTC<br>TGTTGGAATGGTGAATGTACTGGTTCTATTTGCCAGAAATATAATAT<br>GGAAGAGTGTTTCCTGACTAGTCTGAATGGTGCAAAACCAGAAGAG<br>ATGTGTGAAGTAGCTTGCCAGAAATTAGGTCAACCAGCAACATGCGA<br>AAGTACATATGGAATTGCAAAAATGAAAAATATTAGTGGGTTAAAG<br>CTACAACCAGGATCACCATGCAATGAATTTCAAGGCTACTGTGATGT<br>ATTTCAAAAGTGCAGAGCTGTTGATGCAGAAGGACCATTAGCACGCC<br>TCAAGAACTTACTATTAAACCAGCAAACTTTGAGAACTATTAAGCAA<br>TGGATAACAACATATTGGTGGGCTGTCATGCTGATGCTAGTAGGATT<br>GGCCATCTTCATGGGTTTGTTTATAAAGTGTTGTGCTGTACATACACC<br>TAGCAGTAATCCTAGGAAAGCTCCAGCACTTCGTATCAGCGACACGT<br>TGCGTCATCCTGCTGACACTCTTCGCAGAAAGCGGCACCGTCCACCA<br>CCACAACCAGGCATTCCTAGCGGACCACCTCCACCTTATCCAGGACC<br>TCAGCCTTCAGCACCTCCCTCTAGGAGTAGATCTTCAGCTGGACCAT<br>CACGTGGTTACGGAGAAGGTCGGGGACATTACAACAGAAGGGACAG

							AGGCCAAATGCATCATGGTGAAAGGGGGAAGTTGTATATTGAAGAC<br>CCGTCCTATGCTCCTGCTCATCCTGGTCGGGGCAGTGCAATGGAAAT<br>GAGGGTTAAATCACAGGCCTGA
TatEnzMtp03	XP_021004202.1	0	PF01421,<br>PF00200	--	Disintegrin-like	MHWIYIVIIFFCYGTTLRICALLKRKREADSFPFDDVGDE<br>NWPVAEADRLRRIYPENERLIANIPSRFFEVIYPVQVRQH<br>QKLGISTRDATANKVSLPSTGKHFHQTSLLVKAFNYKFR<br>LELELNMYLLAPNLIQKHFLPEGAQQISTQEIEHCYYHA<br>VIKDYPGALAALRTCNGVSGIIHVSNETFVIHPFYGGDLS<br>RKHPHVIYRYFGEAKEKHMCGNTGMHEWGFKQFRIQP<br>GRIKRDVREVFKFIELALVLDQAMFDNRNATRSEVVND<br>AIQVVNCVDMYFRTVNTRVSVLYVETWAHGDQMEINN<br>DVRQTLLNFMEYASRKLYKVAKDATHLLTGHHFKGAE<br>VGMSVPDSICTAKAVGVSQDTNIHEPHLVASTMTHMLG<br>HNIGMSHDQYDNNDECECQDWWGCIMAQTILGQNRIQ<br>PYHFSKCSFQDYINALRIGHGICLFNKPNQLEDFRSCGNK<br>LVEEGEQCDCGSIDECLEHDQCCDPITCKLRVEAECSKG<br>PCCADCKLKASGQPCREAIDECDIPEYCNGRDGQCPTDL<br>FKRNGALCKNGIGFCFNGRCPSADERCEYIWDFGAVSS<br>DIQCYEQFNTQGSLNGHCGTDGKGGYIKCTEENILCGTV<br>QCQQGRRVPIVQGMNKQYARTIVSIGGAEYECKVASGN<br>LDLDIVDLGMMPDGSKCAENKICVNQTCLRLDLFVEPG<br>SCLSNNVALPCSGHGVCSNINTCFCDEGWTAADCSQRL<br>NGDDPAADHTDEPFGGGNHAYIPVSTTQGSASENGQNL<br>KKTPKSTTYGKKDALSAPSLVVVLVSVVGGVFIFFALLA<br>TCYRRSTLPKQEPPHMKKHIVRKHTLPLTVATKRDEDSS<br>QENVNRIITFGSMPSYREDKQRQQLQLKDTSLSEEGESH	ATGCATTGGATATATATAGTTATCATATTTTTCTGTTATGGAACAACG<br>TTACGGATCTGCGCTTTGTTGAAGAGGAAAAGAGAAGCTGATTCATT<br>CCCTTTTGATGATGTTGGAGATGAGAATTGGCCTGTTGCTGAGGCTG<br>ATCGCTTAAGGCGAATATATCCAGAGAATGAACGTCTCATAGCCAAC<br>ATTCCGAGTCGTTTCTTTGAAGTTATCTATCCTGTGCAGGTACGACAG<br>CATCAAAAGCTGGGTATTTCCACAAGGGATGCCACAGCCAACAAAG<br>TAAGCCTCCCTAGTACTGGCAAGCACTTCCATCAGACATCCCTCCTTG<br>TCAAAGCTTTCAATTACAAATTCCGTCTTGAATTGGAATTGAATATGT<br>ACCTTTTGGCACCAAACCTTATCCAAAAGCATTTTTTGCCTGAAGGA<br>GCACAACAAATATCTACTCAGGAAATAGAACATTGTTACTATCATGC<br>TGTTATCAAAGATTATCCAGGTGCTCTTGCTGCACTTAGGACCTGTAA<br>TGGAGTTAGTGGCATTATTCATGTCAGTAATGAAACTTTTGTAATTCA<br>TCCTTTCTATGGAGGTGATCTTTCTAGGAAGCATCCACATGTAATTTA<br>TCGATATTTTGGTGAAGCAAAAGAAAAACATATGTGTGGAAATACA<br>GGAATGCATGAATGGGGCTTCAAACAATTTAGAATACAACCAGGAA<br>GAATTAAGCGGGATGTACGAGAAGTTTTCAAATTCATAGAACTTGCT<br>TTAGTGTTAGATCAGGCAATGTTTGACAATCGGAATGCAACTAGATC<br>TGAAGTAGTAAATGATGCTATCCAGGTTGTTAATTGTGTGGATATGT<br>ACTTTCGAACTGTTAACACTAGAGTATCTGTTTTGTATGTGGAGACAT<br>GGGCTCATGGTGATCAAATGGAAATTAACAACGATGTACGACAGAC<br>ATTGCTAAACTTTATGGAATATGCTTCCCGAAAATTATATAAAGTTG<br>CAAAGGATGCCACCCATTTATTAACAGGCCATCATTTTAAAGGTGCT<br>GAAGTGGGCATGTCTGTGCCAGATAGTATTTGTACAGCTAAAGCAGT<br>TGGAGTTAGTCAAGATACCAATATCCATGAACCCCATCTAGTAGCAA<br>GTACGATGACTCATATGTTAGGACACAACATTGGAATGAGCCATGAC<br>CAGTATGATAATAATGATGAATGTGAATGTCAAGATTGGTGGGGGTG<br>CATTATGGCACAAACAATTCTGGGTCAAAATAGGATACAACCATATC<br>ACTTTTCCAAGTGTAGCTTTCAGGATTATATTAATGCTCTTCGCATTG<br>GTCATGGTATCTGTCTATTCAACAAGCCAAATCAGCTAGAAGACTTC<br>AGGAGTTGTGGAAATAAGCTAGTTGAAGAAGGTGAACAGTGTGATT<br>GTGGTAGTATTGATGAATGCTTAGAACATGATCAGTGTTGTGATCCT<br>ATCACTTGCAAGCTGCGAGTTGAAGCTGAATGCTCCAAAGGTCCATG<br>CTGTGCTGACTGCAAGCTAAAGGCTAGTGGACAACCCTGTCGAGAGG<br>CAATTGATGAGTGTGATATTCCAGAATATTGTAATGGACGTGATGGG<br>CAGTGTCCAACTGACTTGTTCAAGAGAAATGGTGCCTTGTGTAAAAA<br>TGGAATTGGTTTTTGCTTCAATGGTAGATGTCCAAGTGCTGATGAAC<br>GTTGTGAATACATTTGGGATTTTGGTGCAGTATCCTCAGATATCCAAT<br>GTTATGAACAGTTTAACACACAAGGAAGTCTAAATGGTCATTGTGGC<br>ACTGATGGAAAAGGTGGATACATCAAGTGTACAGAAGAAAATATTC<br>TCTGTGGTACTGTTCAGTGTCAGCAAGGTAGACGTGTGCCTATTGTG<br>CAAGGAATGAATAAACAGTATGCACGTACAATTGTATCTATTGGTGG<br>GGCAGAGTATGAATGCAAAGTTGCTAGTGGAAATCTGGACTTAGATA<br>TAGTTGACCTTGGAATGATGCCTGATGGCTCAAAGTGTGCTGAAAAT<br>AAGATATGTGTGAATCAGACTTGCCTCAGGTTAGATCTGTTTGTGGA<br>GCCAGGTAGCTGTCTGTCCAATAATGTTGCACTTCCTTGCTCTGGCCA<br>TGGTGTATGTTCAAATATTAATACTTGCTTTTGTGATGAGGGCTGGAC<br>AGCAGCAGATTGTAGCCAACGTTTAAATGGTGATGATCCTGCAGCTG<br>ATCATACTGATGAACCTTTTGGAGGAGGAAACCATGCTTATATCCCT<br>GTCTCAACTACGCAAGGATCTGCATCAGAAAATGGACAAAATCTAA<br>AAAAGACACCAAAGTCAACAACTTACGGGAAGAAGGATGCTTTAAG<br>TGCACCATCACTTGTGGTTGTGCTCGTTTCAGTTGTTGGAGGGGTTTT<br>TATATTCTTCGCTCTACTAGCAACATGCTACAGAAGAAGCACACTTC<br>CAAAGCAAGAACCACCTCATATGAAAAAGCACATAGTTAGAAAGCA<br>CACACTGCCATTAACTGTTGCTACTAAACGTGATGAAGATTCATCTC<br>AAGAAAATGTGAATAGAATAATTACCTTTGGTAGCATGCCATCTTAT<br>CGGGAAGACAAACAAAGGCAGCAACTGCAGTTAAAAGACACTTCAC<br>TTTCTGAAGAAGGGGAAAGTCAT
TatEnzMtp04	XP_021001151.1	3 e-86	PF00200	--	Disintegrin-like	LKGCFTEPQDAICGNEVVEKGEECDCGWEEDCEEPCCF<br>PMRSNPPRDEPPCHLRPNVVCSPSQGPCCTHDCRIKVGE<br>ECRGDNGCRSASYRDGQGPHCPSSTNKPNKTVCNDEFV<br>CYMGECTGSICMAYGLESCQCKRGLHDPLTKACELCCK<br>LPRD	TTAAAAGGTTGTTTCACTGAGCCACAAGATGCCATCTGTGGAAATGA<br>AGTAGTGGAGAAAGGTGAAGAATGTGACTGTGGCTGGGAAGAGGAC<br>TGTGAAGAACCATGTTGTTTTCCCATGCGTAGCAATCCCCCTAGAGA<br>TGAACCACCTTGTCACCTTAGACCCAATGTAGTTTGCAGCCCAAGCC<br>AAGGTCCTTGTTGTACACATGACTGTAGGATAAAGGTTGGAGAAGAG

							TGTCGTGGAGATAATGGTTGTAGAAGTGCAAGCTACCGTGATGGTCA<br>AGGACCACATTGCCCATCATCCACCAACAAACCAAATAAAACTGTCT<br>GCAACGATGAATTTGTTTGTTATATGGGAGAATGTACAGGCTCGATA<br>TGTATGGCATATGGACTAGAGTCTTGCCAGTGCAAACGTGGCCTACA<br>TGATCCTTTAACAAAAGCATGTGAACTTTGTTGTAAGCTGCCAAGGG<br>AT
TatEnzMtp05	XP_015912698.1	0	PF00557	--	Other	MADKEENEPTIAQDLVVTKYKMAGEMVNRVLGQLIKK<br>CQTGESVINICELGDKLLLDETGKVFKREKEMKKGIAFP<br>TCLSVNNCICHFSPLKSEPDYILKDGDMVKVDLGAHIDG<br>FIAVVAHTLIVGSEKDCKIAGKKADVIKAAYYAAEAAL<br>RLVKPGGENSEVTEAVQKVAESFKCRPVEGMLSYQLKQ<br>YRIDGEKSIIQNPTEAQRKEHEKCEFELHEVYAVDVLIST<br>GEGKGREMDTRTTVYKKTDEIYQLKMKASRAFFSEVD<br>KRFGNMPFTLRAFDDEKKARMGVVECVNHKLVEPFTV<br>LYEKEGEYVAQFKFTVLLMPTGSHKITTGPIDMDIYESE<br>FKLEDESLKSLLNRSVAPKSAKKKKKKAEKAVASSVET<br>GDKIEDEKLNENNTLVED	ATGGCGGACAAAGAAGAAAATGAACCGACAATTGCTCAAGATCTTG<br>TTGTGACTAAATATAAAATGGCTGGAGAAATGGTTAATAGAGTTCTA<br>GGTCAACTGATAAAAAAATGCCAGACTGGAGAGAGTGTAATCAACA<br>TATGCGAATTGGGTGATAAATTGTTACTGGATGAAACAGGGAAGGTT<br>TTTAAAAGGGAAAAAGAAATGAAAAAAGGTATTGCATTTCCAACAT<br>GTTTATCAGTGAACAACTGTATTTGTCATTTTTCACCATTGAAAAGTG<br>AACCTGATTATATTTTAAAAGATGGGGATATGGTTAAAGTGGATTTG<br>GGAGCACACATTGATGGATTTATTGCAGTGGTTGCTCACACATTGAT<br>TGTTGGCTCAGAAAAGGACTGCAAAATTGCTGGTAAGAAGGCAGAT<br>GTCATTAAAGCTGCTTACTATGCTGCAGAAGCTGCTCTAAGACTAGT<br>AAAGCCAGGTGGAGAGAATTCTGAAGTCACTGAAGCTGTTCAGAAA<br>GTAGCAGAATCATTTAAATGTAGGCCTGTTGAAGGTATGCTATCATA<br>TCAGTTAAAACAATACAGGATAGATGGTGAAAAATCGATAATTCAA<br>AATCCTACAGAAGCTCAAAGGAAGGAACATGAAAAATGTGAATTTG<br>AGCTACATGAAGTTTATGCGGTAGATGTTTTAATTAGTACAGGTGAA<br>GGAAAGGGTCGTGAAATGGATACACGTACCACTGTATACAAGAAAA<br>CAGATGAAATATATCAATTAAAGATGAAAGCATCTAGGGCATTTTTT<br>AGTGAAGTAGACAAGAGATTTGGTAATATGCCTTTCACATTAAGAGC<br>ATTTGATGATGAGAAGAAAGCCAGGATGGGTGTAGTTGAATGTGTCA<br>ATCACAAACTTGTTGAACCTTTTACAGTTTTATATGAAAAAGAAGGA<br>GAATATGTAGCACAGTTCAAATTTACTGTTTTGTTAATGCCTACTGGG<br>TCCCATAAAATTACAACTGGTCCTATTGATATGGATATTTATGAATCA<br>GAATTCAAATTAGAAGATGAATCGTTAAAGTCATTGTTAAATCGTTC<br>TGTGGCACCAAAGTCAGCTAAGAAGAAAAAGAAAAAGGCAGAAAA<br>AGCTGTAGCATCATCGGTAGAAACAGGTGACAAAATTGAAGATGAA<br>AAACTAAATGAAAATAATACCTTGGTTGAAGATTGA

TatEnzMtp06	AMO02513.1	0	PF01421,<br>PF00200	--	Disintegrin-like	MEWLVRSDALLGRLVCFIIVLRTCSTLRSKDVRSEFHNH<br>YVIQPKLHTLDGRERRDVNGAKEVPKDNLVMTFEVLGE<br>EFVLDLKMNTDLLPENYFEKYQLNSRTILNKPMKKFRR<br>HCHYLGQIRGIPTSWAAISMCRGINGMVFDGKENYYVH<br>AVNESNHILLKASDWKANNLTCGYKEHYDSNLRSQML<br>NNIKRNRRSAVIQPPSGSSSDSRYVELILVNDYKEFKELN<br>EDVESVFERSKQIANILNGLYIPLNIYIALVGVITWTEKDP<br>IVMSPDGDATLTNFLHYRRQRLAREHPNDNAQLITAMT<br>FDGGVVGKALKGPICTYEYSGGVNMDHSHIVGLVATTV<br>AHELGHNFGMEHDTDECECPEDKCIMAPASSATSPHHW<br>SSCSREYVHMAFQHGMDYCLRNVPSNIVGPICGNGFLE<br>DGEECDCGLKEFCNNLCCNATTCRLNPNATCATGLCCD<br>VHTCKMKHVATLCRGSVSECDLPEYCDGVTEYCPSDV<br>HVQDGTDCGDGKGYCYDGACQSHSNQCKLLWGSTGK<br>TSHHHCYDQNSNGNVNGNCGYNRINKTYKACAKKDVR<br>CGMLQCTHLNERLEFGMPSAAILSRSFINVQGKILPCRT<br>AVIDLGLTSVDPGLTPNGAKCGDGMACLNQKCVPVSHL<br>RKKTCSQNCNDNGICNNRINCHCNYGYGPPHCDGPGHG<br>GSIDSGPPSDPNAGQSFFIAMLIIFLGIIPFIAITAYVVYHY<br>NHHLKNWWLLKIRKEPKKPPEPKSAPPPKKFNYPHGTV<br>KKLEISAPFLQHGPSDPIVTVPLETAQRHIHNGNLPIKAT<br>TSQETPRPVPLRAAPPVPPGPVQRSHSSVGATSSNRRASR<br>PLSEASFSKRNLPPPPRPPPPKHLKPSVRPQSFSAGSNRVT<br>ANDNATNVNNVELSRKGSNASNQNQVKKTATNNASVS<br>AAASVGKPNRQTKPNSAFKPARNSDPNVGRTNSGQISR<br>VASLTQRFEQQGGVNAKV	ATGGAGTGGTTAGTGCGAAGTGATGCTTTGTTGGGGAGATTAGTTTG<br>CTTTATCATCGTTCTGCGCACTTGTTCAACATTAAGAAGTAAAGATGT<br>CCGCTCCGAGTTCCACAACCATTACGTCATCCAACCGAAACTCCACA<br>CCCTGGACGGAAGGGAGAGGCGAGACGTTAACGGCGCAAAGGAAGT<br>CCCCAAAGATAATTTAGTTATGACATTTGAAGTACTTGGTGAAGAAT<br>TTGTGCTAGATCTAAAAATGAATACAGATCTACTGCCAGAAAACTAC<br>TTTGAAAAATATCAACTTAATTCACGCACAATTTTAAATAAACCAAT<br>GAAAAAGTTCAGGAGACACTGTCATTACTTAGGGCAGATCCGAGGC<br>ATTCCTACTTCTTGGGCGGCTATAAGCATGTGTCGTGGAATCAATGG<br>AATGGTATTTGATGGAAAGGAAAATTATTATGTTCATGCAGTTAATG<br>AAAGCAATCATATTCTACTTAAAGCTTCTGACTGGAAAGCAAACAAT<br>CTCACTTGCGGGTACAAAGAACACTATGATAGCAATCTAAGATCTCA<br>AATGTTAAACAATATAAAGAGGAATCGTCGATCAGCTGTGATACAGC<br>CACCATCAGGATCTTCCAGTGACTCCCGCTATGTAGAGCTAATACTT<br>GTCAATGATTATAAGGAGTTCAAAGAACTTAATGAAGATGTTGAAAG<br>TGTTTTTGAAAGAAGTAAACAAATTGCTAATATTCTCAATGGGCTAT<br>ATATTCCACTCAATATTTATATTGCATTGGTTGGTGTCATTACCTGGA<br>CAGAAAAAGATCCAATTGTAATGTCTCCAGATGGTGATGCAACTCTC<br>ACTAATTTCCTTCACTATCGTCGGCAACGTTTAGCACGTGAGCATCCA<br>AATGATAATGCCCAGCTAATTACAGCTATGACATTTGATGGTGGAGT<br>AGTAGGCAAAGCACTAAAGGGACCAATATGTACATATGAATACTCT<br>GGGGGAGTTAATATGGATCATAGCCACATTGTGGGTCTTGTAGCTAC<br>TACTGTTGCACATGAACTTGGGCATAATTTTGGAATGGAGCATGACA<br>CAGATGAGTGTGAGTGTCCAGAAGATAAATGCATAATGGCCCCAGCT<br>TCCAGTGCTACAAGTCCACATCATTGGTCATCATGCTCAAGAGAATA<br>TGTCCATATGGCATTTCAGCATGGGATGGACTATTGCCTAAGAAATG<br>TTCCTTCAAATATTGTTGGACCAATTTGTGGAAATGGATTTTTAGAAG<br>ATGGGGAAGAGTGTGACTGTGGATTAAAGGAGTTTTGTAATAATCTT<br>TGTTGTAATGCAACAACTTGTCGACTAAATCCTAATGCCACTTGTGCT<br>ACAGGACTGTGTTGTGATGTTCATACATGCAAGATGAAGCATGTTGC<br>TACTCTGTGCCGTGGCTCAGTTTCTGAATGTGACCTTCCTGAATACTG<br>TGATGGAGTAACAGAGTATTGCCCATCTGATGTTCATGTCCAAGATG<br>GAACTGATTGTGGAGATGGAAAGGGTTATTGCTATGATGGAGCATGT<br>CAGTCACATAGTAACCAGTGCAAGCTATTATGGGGATCAACTGGTAA<br>AACCTCTCATCATCACTGCTATGATCAAAATAGTAATGGAAATGTTA<br>ATGGAAATTGTGGTTACAACAGAATTAATAAGACCTATAAAGCCTGT<br>GCTAAAAAGGATGTCCGTTGTGGAATGCTTCAGTGCACACACTTGAA<br>TGAACGACTTGAATTTGGTATGCCTTCAGCTGCTATTTTATCAAGATC<br>ATTCATAAATGTGCAAGGGAAAATTCTTCCATGTCGAACAGCTGTCA<br>TTGATTTAGGACTCACGTCAGTTGATCCAGGCCTAACCCCCAATGGA<br>GCAAAGTGTGGTGATGGCATGGCATGTCTGAACCAAAAGTGTGTGCC<br>AGTTTCTCACTTGAGGAAGAAAACCTGCTCGCAAAACTGCAATGACA<br>ATGGGATATGCAATAACCGTATTAACTGTCATTGTAATTACGGCTAT<br>GGCCCACCGCATTGTGATGGCCCAGGTCATGGTGGCAGTATAGATAG<br>TGGACCACCATCTGATCCAAACGCTGGACAGAGCTTTTTCATTGCAA<br>TGCTTATCATCTTTCTTGGTATCATACCATTCATTGCCATCACTGCAT<br>ATGTTGTGTATCACTACAACCATCATCTCAAGAATTGGTGGCTTCTTA<br>AGATAAGGAAGGAACCAAAGAAGCCACCTGAACCTAAAAGTGCACC<br>TCCACCAAAGAAATTCAATTATCCTCATGGTACAGTAAAAAAGTTGG<br>AAATATCAGCTCCATTCCTACAACATGGCCCTTCTGATCCTATAGTAA<br>CAGTCCCATTGGAAACAGCACAGAGGCATATTCACAATGGCAATTTG<br>CCAATTAAGGCAACAACGTCACAAGAAACTCCTCGTCCTGTGCCTCT<br>CAGAGCTGCTCCACCAGTCCCACCTGGACCAGTGCAGAGATCTCATT<br>CATCAGTTGGTGCAACGTCATCAAATCGAAGAGCCTCTCGACCCCTG<br>AGCGAGGCATCCTTTAGTAAGCGTAATTTACCGCCTCCACCTCGTCC<br>ACCACCACCTAAACATTTAAAACCAAGTGTTCGACCACAGTCTTTCA<br>GTGCAGGTAGTAATAGGGTGACAGCAAATGACAATGCAACAAATGT<br>TAATAATGTAGAACTGAGTCGGAAAGGTAGTAATGCTTCTAATCAGA<br>ATCAAGTTAAAAAGACTGCAACGAATAATGCTTCTGTGAGTGCAGCA<br>GCTTCTGTAGGAAAGCCTAATAGACAGACAAAACCCAACAGTGCCTT<br>CAAACCAGCAAGGAATTCAGATCCTAATGTTGGAAGGACCAATAGT<br>GGACAAATATCGAGGGTAGCATCATTAACACAACGGTTTGAGCAAC<br>AGGGTGGTGTAAATGCTAAAGTATAA

TatEnzMtp07	XP_021004202.1	0	PF01421,<br>PF00200	--	Disintegrin-like	MHWIYIVIIFFCYGTTLRICALLKRKREADSFPFDDVGDE<br>NWPVAEADRLRRIYPENERLIANIPSRFFEVIYPVQVRQH<br>QKLGISTRDATANKVSLPSTGKHFHQTSLLVKAFNYKFR<br>LELELNMYLLAPNLIQKHFLPEGAQQISTQEIEHCYYHA<br>VIKDYPGALAALRTCNGVSGIIHVSNETFVIHPFYGGDLS<br>RKHPHVIYRYFGEAKEKHMCGNTGMHEWGFKQFRIQP<br>GRIKRDVREVFKFIELALVLDQAMFDNRNATRSEVVND<br>AIQVVNCVDMYFRTVNTRVSVLYVETWAHGDQMEINN<br>DVRQTLLNFMEYASRKLYKVAKDATHLLTGHHFKGAE<br>VGMSVPDSICTAKAVGVSQDTNIHEPHLVASTMTHMLG<br>HNIGMSHDQYDNNDECECQDWWGCIMAQTILGQNRIQ<br>PYHFSKCSFQDYINALRIGHGICLFNKPNQLEDFRSCGNK<br>LVEEGEQCDCGSIDECLEHDQCCDPITCKLRVEAECSKG<br>PCCADCKLKASGQPCREAIDECDIPEYCNGRDGQCPTDL<br>FKRNGALCKNGIGFCFNGRCPSADERCEYIWDFGAVSS<br>DIQCYEQFNTQGSLNGHCGTDGKGGYIKCTEENILCGTV<br>QCQQGRRVPIVQGMNKQYARTIVSIGGAEYECKVASGN<br>LDLDIVDLGMMPDGSKCAENKICVNQTCLRLDLFVEPG<br>SCLSNNVALPCSGHGVCSNINTCFCDEGWTAADCSQRL<br>NGDDPAADHTDEPFGGGNHAYIPVSTTQGSASENGQNL<br>KKTPKSTTYGKKDALSAPSLVVVLVSVVGGVFIFFALLA<br>TCYREDKQRQQLQLKDTSLSEEGESH	ATGCATTGGATATATATAGTTATCATATTTTTCTGTTATGGAACAACG<br>TTACGGATCTGCGCTTTGTTGAAGAGGAAAAGAGAAGCTGATTCATT<br>CCCTTTTGATGATGTTGGAGATGAGAATTGGCCTGTTGCTGAGGCTG<br>ATCGCTTAAGGCGAATATATCCAGAGAATGAACGTCTCATAGCCAAC<br>ATTCCGAGTCGTTTCTTTGAAGTTATCTATCCTGTGCAGGTACGACAG<br>CATCAAAAGCTGGGTATTTCCACAAGGGATGCCACAGCCAACAAAG<br>TAAGCCTCCCTAGTACTGGCAAGCACTTCCATCAGACATCCCTCCTTG<br>TCAAAGCTTTCAATTACAAATTCCGTCTTGAATTGGAATTGAATATGT<br>ACCTTTTGGCACCAAACCTTATCCAAAAGCATTTTTTGCCTGAAGGA<br>GCACAACAAATATCTACTCAGGAAATAGAACATTGTTACTATCATGC<br>TGTTATCAAAGATTATCCAGGTGCTCTTGCTGCACTTAGGACCTGTAA<br>TGGAGTTAGTGGCATTATTCATGTCAGTAATGAAACTTTTGTAATTCA<br>TCCTTTCTATGGAGGTGATCTTTCTAGGAAGCATCCACATGTAATTTA<br>TCGATATTTTGGTGAAGCAAAAGAAAAACATATGTGTGGAAATACA<br>GGAATGCATGAATGGGGCTTCAAACAATTTAGAATACAACCAGGAA<br>GAATTAAGCGGGATGTACGAGAAGTTTTCAAATTCATAGAACTTGCT<br>TTAGTGTTAGATCAGGCAATGTTTGACAATCGGAATGCAACTAGATC<br>TGAAGTAGTAAATGATGCTATCCAGGTTGTTAATTGTGTGGATATGT<br>ACTTTCGAACTGTTAACACTAGAGTATCTGTTTTGTATGTGGAGACAT<br>GGGCTCATGGTGATCAAATGGAAATTAACAACGATGTACGACAGAC<br>ATTGCTAAACTTTATGGAATATGCTTCCCGAAAATTATATAAAGTTG<br>CAAAGGATGCCACCCATTTATTAACAGGCCATCATTTTAAAGGTGCT<br>GAAGTGGGCATGTCTGTGCCAGATAGTATTTGTACAGCTAAAGCAGT<br>TGGAGTTAGTCAAGATACCAATATCCATGAACCCCATCTAGTAGCAA<br>GTACGATGACTCATATGTTAGGACACAACATTGGAATGAGCCATGAC<br>CAGTATGATAATAATGATGAATGTGAATGTCAAGATTGGTGGGGGTG<br>CATTATGGCACAAACAATTCTGGGTCAAAATAGGATACAACCATATC<br>ACTTTTCCAAGTGTAGCTTTCAGGATTATATTAATGCTCTTCGCATTG<br>GTCATGGTATCTGTCTATTCAACAAGCCAAATCAGCTAGAAGACTTC<br>AGGAGTTGTGGAAATAAGCTAGTTGAAGAAGGTGAACAGTGTGATT<br>GTGGTAGTATTGATGAATGCTTAGAACATGATCAGTGTTGTGATCCT<br>ATCACTTGCAAGCTGCGAGTTGAAGCTGAATGCTCCAAAGGTCCATG<br>CTGTGCTGACTGCAAGCTAAAGGCTAGTGGACAACCCTGTCGAGAGG<br>CAATTGATGAGTGTGATATTCCAGAATATTGTAATGGACGTGATGGG<br>CAGTGTCCAACTGACTTGTTCAAGAGAAATGGTGCCTTGTGTAAAAA<br>TGGAATTGGTTTTTGCTTCAATGGTAGATGTCCAAGTGCTGATGAAC<br>GTTGTGAATACATTTGGGATTTTGGTGCAGTATCCTCAGATATCCAAT<br>GTTATGAACAGTTTAACACACAAGGAAGTCTAAATGGTCATTGTGGC<br>ACTGATGGAAAAGGTGGATACATCAAGTGTACAGAAGAAAATATTC<br>TCTGTGGTACTGTTCAGTGTCAGCAAGGTAGACGTGTGCCTATTGTG<br>CAAGGAATGAATAAACAGTATGCACGTACAATTGTATCTATTGGTGG<br>GGCAGAGTATGAATGCAAAGTTGCTAGTGGAAATCTGGACTTAGATA<br>TAGTTGACCTTGGAATGATGCCTGATGGCTCAAAGTGTGCTGAAAAT<br>AAGATATGTGTGAATCAGACTTGCCTCAGGTTAGATCTGTTTGTGGA<br>GCCAGGTAGCTGTCTGTCCAATAATGTTGCACTTCCTTGCTCTGGCCA<br>TGGTGTATGTTCAAATATTAATACTTGCTTTTGTGATGAGGGCTGGAC<br>AGCAGCAGATTGTAGCCAACGTTTAAATGGTGATGATCCTGCAGCTG<br>ATCATACTGATGAACCTTTTGGAGGAGGAAACCATGCTTATATCCCT<br>GTCTCAACTACGCAAGGATCTGCATCAGAAAATGGACAAAATCTAA<br>AAAAGACACCAAAGTCAACAACTTACGGGAAGAAGGATGCTTTAAG<br>TGCACCATCACTTGTGGTTGTGCTCGTTTCAGTTGTTGGAGGGGTTTT<br>TATATTCTTCGCTCTACTAGCAACATGCTACAGGGAAGACAAACAAA<br>GGCAGCAACTGCAGTTAAAAGACACTTCACTTTCTGAAGAAGGGGA<br>AAGTCAT

TatEnzMtp08	XP_021000996.1	0	PF13574	--	Disintegrin-like	MIQNKFFLSLLVCFVTGGETGRLNQYISYFEPLRYNHET<br>VHQGHLRAKRSPSDSLVYVNFHAHGRHFNIRLKRDTSA<br>FAPDVVIETTNGVAEVDTSHLYDGHLLGESGSTVYGSIR<br>NGVFEGKIHTPNKVYYVERSHKYFSSNPQPFHSVIYSSD<br>DVEDPYADKRESHAGGCGITEDVLQWMQEVSNSADPK<br>EMSPTNKTNPEQSHLERSERSTAAENAPHNIYTREAQRD<br>INQIDQLQAKRACTLYVQTDVFLWKHILTYEKTASSARE<br>EISSLISQHVRAVNYIYENTDFDGYRGIKFVVQRIKINDT<br>SSCSNQGLDYKRTNPFCSPNIDVSNFLNLNSQFNHDDFC<br>LAYIFTYRDFSGGTLGLAWVASASGASGGICEKYKPYN<br>ENINGRQVQTKRSLNTGVITFVNYNSRVPPKVSELTLAH<br>EIGHNFGSPEVMVLSVVIKLWKILKNATVGMMQRNAM<br>MNAAIHETSLFSK	ATGATACAAAACAAGTTCTTTTTGTCACTGCTTGTTTGTTTTGTGACA<br>GGAGGTGAAACCGGTCGACTTAATCAGTATATAAGTTATTTTGAGCC<br>ACTGAGGTACAACCACGAAACTGTACATCAAGGTCATTTACGTGCCA<br>AACGTTCACCTTCAGATTCCTTAGTTTATGTGAATTTCCATGCACATG<br>GAAGGCATTTTAATATTCGACTTAAAAGGGACACATCAGCTTTTGCT<br>CCAGATGTTGTTATAGAAACTACTAATGGTGTAGCAGAAGTTGACAC<br>ATCCCACTTATATGATGGGCATTTGTTAGGGGAATCAGGATCCACTG<br>TATATGGCTCAATAAGGAATGGTGTATTTGAGGGAAAGATTCATACA<br>CCGAACAAAGTTTATTATGTAGAACGCTCTCACAAATATTTTTCATCA<br>AACCCTCAACCATTTCACTCTGTGATATACTCATCAGATGATGTGGA<br>AGATCCATATGCTGACAAACGGGAGTCACATGCAGGTGGATGTGGC<br>ATCACAGAGGATGTTTTACAGTGGATGCAAGAAGTTAGCAATTCAGC<br>AGATCCTAAAGAAATGTCCCCTACAAACAAGACAAACCCGGAGCAG<br>TCTCATTTGGAAAGAAGTGAACGTTCAACAGCTGCGGAAAATGCACC<br>TCATAATATCTATACAAGAGAAGCGCAACGTGATATTAATCAGATAG<br>ACCAATTACAAGCAAAAAGAGCATGTACATTATATGTGCAAACAGA<br>TGTATTTCTATGGAAGCATATTTTGACGTATGAAAAAACTGCTAGTA<br>GTGCACGAGAAGAAATATCTTCATTAATATCTCAACATGTGAGAGCC<br>GTTAACTATATTTATGAAAATACTGACTTTGATGGCTATCGGGGCAT<br>AAAGTTTGTGGTGCAAAGAATTAAGATAAATGACACATCATCGTGTT<br>CCAATCAAGGGTTGGATTACAAACGAACAAATCCATTCTGCTCGCCA<br>AATATTGATGTATCAAACTTTTTAAATTTGAATTCTCAATTCAACCAT<br>GATGATTTCTGTTTAGCATACATTTTTACGTACAGAGATTTCTCTGGT<br>GGTACATTAGGACTTGCATGGGTTGCATCAGCATCAGGTGCTTCTGG<br>AGGAATCTGTGAGAAATACAAGCCATACAATGAAAATATTAATGGC<br>AGACAAGTACAAACCAAAAGAAGCCTGAACACTGGTGTGATTACCT<br>TTGTCAATTACAACAGCCGAGTACCTCCAAAAGTGTCAGAATTGACA<br>TTGGCTCATGAAATTGGACACAACTTTGGTTCACCTGAAGTGATGGT<br>GCTTTCTGTGGTAATAAAATTGTGGAAAATACTGAAGAATGCGACTG<br>TGGGTATGATGCAAAGGAATGCAATGATGAATGCTGCTATCCACGAG<br>ACGTCACTCTTTTCCAAATGA

TatEnzMtp09	XP_021000340.1	0	PF13582	--	Disintegrin-like	SDSSHIEPYSTRHLFRHGKSPSVFPVAKRPMYLCSILLAT<br>LWICGVYTYQNIKVHERMTPEELRQIFHVDLHNEVPEY<br>DVVNVRSLSKRSTPSGNRKQLHLSAFGKKLHLNLKKNE<br>EFENKLASMKVFEAETNQNGLKYREMEPQEHASDMGV<br>SFQDEQQMAAVLVRHSDSGTVQVEGTIGNNLVIKPVPPS<br>LVAFDDAYVDDEMFLDEDGINSTKPSQRHLPILNATSHII<br>YMRKGYGSEQLSDYMDLESGHTDKQNWTMRSPRVKR<br>KAPSTVFPEVLVVVDYNTLLMHGSDSRALKRYFVSFWN<br>GVDLRYKTLSNPRVKVSLAGMVVAKDKDATPYLEKNR<br>LRPPNYDAVDAAGALSDMGKYLYREDRLPTFDVAVVIT<br>KLDMCRRQFNGGRCSRGTAGFAYVGGACVVNKRLEKV<br>NSVAIIEDSGGFSGIIVAAHEVGHLLGCVHDGSQPPSYLG<br>GPGATSCPWEDGYIMSDLRHTDRGFKWSPCSVQQFKHF<br>LHGETAVCLYNYPHDNQALARILPGTTMSLDDQCRKDR<br>GTTACFKDARVCAQLFCYDTSSGFCVSFRPAAEGSTCG<br>HGQVCR	TCGGATTCAAGTCACATCGAACCATATTCCACCCGCCATCTTTTCCGG<br>CACGGAAAAAGTCCGTCCGTATTTCCGGTGGCAAAACGACCCATGTA<br>CCTGTGTTCCATTCTCTTAGCCACTCTGTGGATATGTGGCGTATATAC<br>GTATCAAAATATAAAGGTTCACGAAAGGATGACGCCCGAAGAATTA<br>AGACAAATATTTCATGTGGATTTACATAACGAAGTGCCGGAATATGA<br>CGTCGTTAACGTTCGATCCTTGTCAAAACGTTCAACGCCCTCTGGCA<br>ACAGGAAACAGCTGCATCTATCAGCGTTCGGGAAAAAATTGCATTTA<br>AATTTAAAAAAGAATGAAGAATTTGAAAACAAGTTGGCATCGATGA<br>AAGTATTTGAAGCAGAAACCAATCAAAATGGACTTAAATATCGAGA<br>AATGGAACCACAAGAGCACGCGTCTGACATGGGTGTTAGTTTCCAAG<br>ATGAGCAACAGATGGCAGCAGTATTAGTAAGACACTCAGACAGCGG<br>AACAGTACAAGTGGAAGGCACAATCGGTAACAACCTTGTAATTAAA<br>CCAGTACCTCCATCGTTGGTTGCATTTGATGATGCTTACGTCGATGAC<br>GAGATGTTCCTTGATGAAGATGGAATTAATAGTACAAAGCCAAGTCA<br>GCGCCATCTACCGATACTAAATGCAACTAGTCATATCATTTATATGA<br>GAAAAGGATATGGTTCGGAACAACTTAGTGATTACATGGATTTGGAA<br>AGTGGTCATACAGACAAGCAAAACTGGACAATGAGGAGTCCAAGAG<br>TCAAAAGAAAAGCTCCTTCAACCGTTTTTCCTGAGGTTTTAGTAGTTG<br>TGGATTATAATACTCTGTTGATGCACGGAAGTGATAGTCGGGCATTA<br>AAAAGATACTTTGTCAGTTTTTGGAACGGGGTAGATTTGCGTTACAA<br>GACACTCTCGAATCCTAGGGTTAAAGTTAGTTTAGCTGGGATGGTAG<br>TGGCAAAGGATAAAGATGCTACGCCTTATCTAGAAAAGAATCGCCTT<br>AGGCCACCAAATTACGATGCTGTGGATGCTGCGGGAGCCTTGTCTGA<br>CATGGGAAAGTACTTATATAGAGAAGACAGGCTACCGACCTTCGATG<br>TTGCTGTTGTCATCACTAAATTAGATATGTGTAGAAGACAGTTCAAT<br>GGGGGACGATGTAGCCGTGGAACTGCGGGTTTCGCTTATGTGGGTGG<br>TGCTTGTGTAGTTAATAAAAGACTAGAAAAAGTAAACAGTGTTGCCA<br>TTATCGAGGACAGTGGTGGATTTAGTGGTATCATTGTAGCAGCTCAT<br>GAGGTTGGCCACCTGTTAGGCTGCGTTCACGATGGCTCCCAACCACC<br>CAGTTACTTAGGAGGACCAGGTGCTACCAGTTGTCCATGGGAAGATG<br>GTTATATCATGAGTGACCTTCGACACACTGATAGGGGGTTCAAATGG<br>TCACCTTGCAGCGTACAGCAGTTTAAACATTTTTTACATGGAGAAAC<br>TGCTGTGTGTCTTTACAACTATCCTCATGACAACCAAGCTCTAGCAA<br>GAATATTACCTGGAACTACGATGTCTTTGGATGATCAATGTAGAAAG<br>GATAGAGGCACCACTGCTTGTTTTAAAGATGCCAGAGTATGTGCGCA<br>GTTATTTTGTTATGATACATCATCAGGCTTCTGTGTGTCGTTCAGGCC<br>AGCAGCAGAAGGTTCTACTTGCGGCCATGGTCAAGTATGCCGA

TatEnzMtp10	KFM63257.1	5 e-25	PF13582	--	Disintegrin-like	RKPSMLSLFLLASFTFVIGKAQDHEIVYPRMIISRSGEKRVLFRAFDRDFRLRLEPVDALTDDLVVHTTDDNGEEVFYKPNVDNLRETILQDPNVGATISIDDNGPLKIKGVINEKLRIEPDVSSHHKGGHHGGHYGKKGKKGKYYGKYMRQYDEFSEGIEQEREDPMEQIPHRVYEVTQRINDPEFFYDEIMPPDLKYNLSNEKTIENRADIIIVEVAVVSDVEHSKMLGSNDDCAVYHVILMNSVNLRFRTMEKTNIRFLLTDIVRNQPGQQPYIDGCMVDGHALSSETLYALCKYVYDNPTKFQADIVHLVTKTPLGDLDKSTNTINPGVIGIAFVGAACNRKYKCGISEDDGSCFSGSSTIAHENGHLLGCSHDGDPCAYDGCPGSTQCSWNDGFIMSYVRNNEDQYAFSSCCDDCVSYFASLRSSYCLSVNNTKRIVNVYDNRLSGTFLKEKWGTTSESYYDIKCKKARVDWSIYRYFPGCCIDAYYCRTVADSRGSFYYWKCNADDGDPCNENKHCENGYCVDN	CGAAAGCCGAGTATGCTGTCCTTGTTCCTATTAGCAAGCTTTACTTTTGTTATTGGTAAAGCCCAAGATCATGAAATCGTTTATCCACGTATGATCATAAGCCGATCCGGTGAAAAACGAGTGCTTTTTCGAGCATTTGACCGAGATTTTCGTTTAAGATTAGAACCAGTCGATGCGTTAACAGACGATTTGGTAGTTCACACGACAGACGATAATGGAGAAGAAGTCTTTTATAAGCCCAATGTTGACAATTTGAGAGAAACCATTCTACAGGATCCAAATGTTGGAGCCACCATCTCCATTGACGATAATGGACCTCTGAAGATTAAAGGAGTCATCAATGAGAAATTGCGTATTGAACCAGATGTGAGCAGCCATCATAAAGGCGGACATCATGGAGGACATTATGGAAAAAAAGGGAAAAAAGGCAAATACTATGGGAAATATATGCGCCAATATGATGAATTTAGTGAAGGAATAGAACAAGAAAGAGAAGATCCTATGGAACAGATTCCGCATAGAGTATATGAGGTCACTCAGAGGATTAACGATCCTGAATTTTTTTATGATGAAATCATGCCTCCTGATCTCAAGTATAACTTATCCAATGAGAAAACTATTGAAAACCGTGCAGACATAATAATCGTGGAAGTTGCTGTGGTTTCAGACGTTGAACATTCAAAAATGCTTGGTTCAAACGACGATTGCGCTGTATATCATGTCATCTTAATGAATTCCGTTAACTTAAGGTTTCGAACCATGGAAAAAACCAACATAAGATTTTTGTTAACTGATATTGTGAGAAACCAACCAGGGCAACAGCCATACATTGATGGTTGTATGGTCGATGGTCATGCTCTATCGAGCGAAACTTTATACGCTTTGTGCAAATATGTGTATGATAACCCAACGAAATTTCAAGCAGATATTGTTCACTTAGTAACTAAGACACCATTGGGAGACCTGGACAAATCAACCAATACAATAAATCCTGGTGTTATAGGAATTGCCTTCGTAGGAGCAGCTTGTAACAGAAAGTATAAGTGTGGTATTTCTGAAGATGATGGTAGCTGCTTTAGTGGAAGTTCAACCATTGCCCATGAGAATGGTCATTTACTTGGTTGTTCCCATGATGGAGATCCATGTGCCTATGATGGATGTCCCGGATCAACACAATGCTCTTGGAATGATGGTTTTATAATGAGTTATGTGAGAAATAACGAAGATCAATATGCGTTCTCATCGTGTTGCGACGATTGCGTATCGTATTTTGCATCGTTACGCTCCTCTTACTGCCTGTCAGTAAACAATACTAAAAGGATAGTTAACGTTTATGATAATCGTCTGTCTGGAACTTTTTTAAAAGAAAAATGGGGAACCACAACATCAGAAAGTTATTACGATATCAAATGCAAGAAAGCAAGAGTTGATTGGTCTATTTATCGATATTTTCCTGGTTGCTGTATAGATGCATATTACTGTAGAACAGTAGCAGATAGTCGTGGTTCTTTCTATTATTGGAAGTGCAATGCCGACGATGGAGACCCTTGCAATGAGAACAAACACTGCGAGAACGGATATTGTGTTGACAAT
TatEnzMtp11	AMO02515.1	1 e-93	PF01421	--	Disintegrin-like	GNTGMHEWGFKQFRIRPAKLKRDVSQVFKYIELALVIDQAMFDNRNATRSEVVNDAVQIVNCVDMYFRTVNTRVSVVYVETWAHGDQIEAGSDVRQTLLNFMEYTSRKLYKVAKDATHLITGRHFRGNEVGMAVPDSICTAKA	GGAAACACAGGAATGCACGAATGGGGTTTTAAGCAATTTCGGATACGTCCAGCTAAGCTCAAGCGTGACGTGAGCCAGGTATTTAAATATATTGAATTGGCACTTGTGATCGATCAAGCTATGTTTGACAACAGAAATGCTACCAGATCTGAAGTTGTAAATGATGCTGTTCAGATAGTCAACTGTGTGGATATGTATTTTCGCACAGTGAACACACGGGTTTCTGTTGTGTATGTTGAAACATGGGCACATGGGGACCAAATTGAAGCAGGCAGTGATGTGCGACAAACCTTGCTCAATTTCATGGAATACACATCACGCAAATTTATAAAGTCGCCAAAGATGCAACACATTTAATAACTGGTAGGCATTTTCGAGGCAATGAAGTTGGGATGGCTGTTCCTGATAGCATTTGCACTGCCAAAGCT
TatEnzMtp12	XP_019771969.1	7 e-8	PF01421	--	Disintegrin-like	SPYVNMASLSILILVLSTFSLYGALNGDNTKVEILAVVEKELSDEFKLDNWRKSYFCRIFNEVQRYYDTIEGVKIKIILSGLKIITESKDQPFIERWTWKRGNRTYLRTICLQKFSRFARGQLWSKDYDVIFLLTRKAIADIYSEIDGIAYKYGTCNFEEKFGVMVPDPKIIAHELGHLLGASNDGSRNPCSSDDGFVMAEYSHLVTENYGRFSSCTTDAIKMHLKSPKSYCLRRNDVQNTVMCENKTSFHDICYPISSVVSRMKGGGKIDKL	TCGCCTTATGTTAATATGGCATCGCTTTCAATCCTAATTTTAGTTTTGAGCACCTTCTCACTTTATGGAGCTTTGAATGGTGATAACACGAAAGTCGAAATTCTTGCCGTTGTGGAGAAGGAACTTTCTGATGAGTTTAAATTAGACAACTGGAGAAAATCATACTTTTGTCGTATATTCAATGAGGTGCAACGATATTACGATACCATAGAGGGAGTCAAAATAAAAATTATTCTCAGCGGATTAAAGATTATTACGGAATCTAAGGATCAACCTTTTATTGAACGCTGGACTTGGAAAAGGGGAAACAGAACTTATCTAAGAACCATATGTCTACAGAAGTTTTCGAGATTCGCGCGAGGGCAATTATGGAGTAAAGATTATGACGTCATATTCCTGTTAACCAGGAAAGCTATAGCAGACATCTATTCTGAAATCGATGGTATAGCTTACAAGTATGGCACCTGCAATTTGAGGAAAAATTTGGAGTAATGGTTCCTGATCCGAAAATAATTGCGCACGAGCTTGGTCATCTGTTAGGTGCTTCCAACGATGGTTCAAGGAATCCATGTTCTAGCGATGATGGCTTTGTAATGGCTGAGTATTCACATCTCGTGACAGAAAACTATGGAAGGTTTTCTAGTTGTACAACTGACGCAATTAAAATGCATCTGAAATCTCCTAAGAGCTACTGTCTTCGAAGAAACGATGTTCAGAATACAGTAATGTGTGAAAACAAAACGTCTTTCCACGATATCTGCTATCCAATAAGCTCAGTGGTTTCCAGAATGAAGGGAGGAGGCAAAATTGATAAGCTA

TatEnzMtp13	XP_013781814.1	1 e-13	PF01421	--	Disintegrin-like	RLYREERGLNMPAFSIQIFVLSMSIFALTEVLYSEEITVEVLAVLEKEIADEYKFESERKANLCSIFKEVQGYYDNFEGVKINIVLNGIKTLTGPYFRPSTDFPHYKYATTMKDERVVQFSRFMQTQTWSNRYDVILLLMKEVFLGKASLFGGLSYLNGSCEHKRKVGIVIPDPRIIAHEIGHLLGAQNDGTNNSCSSEDGYIMQSTATVSVENYHRFSNCSIEEIKRRIRSPEAHCLYTNDIMDSVKCGQMTSCHEHCLPITSVVSSTIDDWKN	CGGTTATACAGAGAAGAAAGAGGATTAAACATGCCTGCATTTTCTATCCAAATTTTTGTTTTGAGTATGAGCATATTTGCATTAACTGAAGTTCTGTATAGTGAAGAAATAACAGTAGAAGTTCTTGCAGTTCTGGAGAAGGAAATAGCCGATGAGTATAAATTTGAGTCCGAGAGAAAAGCAAATCTTTGCAGTATTTTCAAAGAGGTGCAGGGATATTATGATAATTTTGAAGGAGTTAAAATAAACATTGTTCTCAATGGAATCAAGACCCTTACGGGCCCGTATTTCCGTCCATCTACTGATTTTCCGCATTATAAATATGCAACTACAATGAAAGATGAAAGAGTGGTGCAATTTTCTCGCTTCATGCAAACACAAACGTGGAGTAACAGATATGATGTCATATTACTTTTAATGAAAGAAGTTTTTTTAGGCAAGGCCTCGTTATTCGGTGGTCTCTCTTATCTCAATGGTTCCTGCGAACATAAGCGGAAAGTAGGAATAGTGATCCCTGATCCGAGAATAATAGCGCACGAAATTGGCCATTTATTAGGTGCTCAGAACGATGGTACCAATAATTCATGTTCAAGCGAAGACGGATATATAATGCAATCAACAGCTACTGTATCTGTAGAAAATTACCATAGATTTTCCAATTGTTCAATTGAAGAAATTAAGAGACGTATAAGATCGCCTGAAGCTCATTGTCTCTATACCAATGATATAATGGATAGTGTAAAATGTGGACAGATGACATCTTGCCATGAACACTGCCTCCCAATAACCTCTGTGGTTTCCAGCACAATTGATGACTGGAAAAATTGATTAAGAAAACATAACATTGAA
TatEnzMtp14	XP_013781814.1	6 e-14	PF01421	--	Disintegrin-like	EKQVNLMSNCCLIAMLALSIEIFVFSIFPLIEAQNSKKEEAEVEVLAILEKEYSDEFESDSKRKLDICDIFNEKTEDQPFIEHSSWRNNNVTYLNKTVLAKFSSFLQKEVWNNQYDVILLLTKKLMAGKNWSLKGAAYRYGSCRHSLKFGIVFPDPRMIAHEIGHLLGADNDGANNTCSSDDGYLMRAYSDIVSYENHRKFSHCSIEAIKQHLESHWSDCLRDTGVKDTVRCGERTSCNNHCRII	GAAAAGCAAGTTAATTTAATGAGTAACTGCTGCCTTATTGCCATGCTCGCGCTTTCAATTGAGATTTTCGTTTTTAGTATCTTCCCACTTATTGAAGCTCAGAATAGCAAAAAAGAAGAAGCAGAAGTCGAAGTTCTTGCCATTCTAGAGAAGGAATATTCTGATGAGTTTGAATCTGACTCCAAAAGAAAATTAGATATTTGTGATATTTTTAATGAGAAAACGGAGGACCAGCCTTTCATTGAACACTCGTCTTGGCGAAATAACAACGTAACTTACCTAAACAAAACCGTTCTGGCCAAATTTTCAAGTTTCTTGCAAAAAGAAGTATGGAATAATCAGTATGATGTCATACTGCTTTTAACAAAGAAATTGATGGCAGGCAAAAAATTGGAGTCTAAAAGGTGCAGCTTACCGATATGGATCCTGCAGGCATTCGCTAAAATTCGGAATAGTTTTTCCTGATCCAAGGATGATAGCACACGAAATTGGCCATCTGTTAGGTGCTGACAATGATGGCGCAAATAATACATGTTCCAGCGACGATGGCTATTTAATGAGAGCATATTCGGATATTGTATCTTATGAAAACCATAGGAAATTTTCTCATTGTTCAATTGAAGCTATTAAACAGCATCTGGAGTCACATTGGAGCGACTGTCTTCGAGATACCGGCGTTAAAGACACAGTAAGATGTGGAGAGAGAACGTCTTGCAACAACCACTGCCGAATAATA

CAP-Superfamily

ID	ID/Reference protein	e-value	PFAM domain	MW (Da)	Sub-classification	Amino acid sequence	Nucleotide sequence

TatOthCRI01	JAV48224.1	0	PF00188	--	CRISP	MIKKITRFTVSFVPQIWIIVLLILSSSSSHGETICEAKYRNI<br>HPEHSMCKARNNTCKFRQGLEENAKELVDFHNRIRKSI<br>RQIIGNNFSEGKSMKNMQWDHELYLIAQKHVLQCTGLP<br>DCYQCHQTDRFYVEQNFAVKTFNSVKESFNGTIERFKA<br>VIKEWAEELSEYGPEVVNKFHFTGLPTNWTNIFRATTNK<br>VGCASIAYDTQKEGTFTEVYVCNYGPAILTENEVIYEPV<br>YTNCNNGLCKNQFLEKYGNSNFYRSNRPPERGKRSGAQ<br>LQHNNGRLSRNSEEEECPAKYRNITVDHSYCKPDVEGC<br>VFSRKYVRYRKKLLDTHNEIRNSVEAYAGWAYGTATN<br>MRVMEWDDELYDIAKRYVIQCLVEPDCHLCHQAADFP<br>VEQNFAVKVISYNGPQSQERFGAVIKGWAMELKNFAQS<br>DVEHLSKEIIQRDDKNWINVFRASAWKVGCASISFETSP<br>KPDLIVKEVYICNYGPAELSEGEEVYQIGESCSNCDPGFD<br>CDEKYPNLCSKKEKPESNDDWLTQTMAAKTTAETTAS<br>KNSIRTESDIFADEVDGSTVGSVKTSVNITTPTMTTAITTP<br>SAPTTTTITVSPVPTVSLSTENPHATEMPVETSTVSAVTS<br>KQIVLYNNKTKTSKGNIVWNCTLRFFDDEECRREQQCF<br>QAWTMSNDGKQPFMEIDIPENRRTALLFLHQIYIEKPSCF<br>TFTYMKDGLSRLSSLDSTLYGIVVRTESNEHVMVDVNG<br>NVPVWNSLAVDIPWEKVFVQVGVAVKTYDGRGEQHIQ<br>IKDLLIHVGPCTAL	ACACAATGTAGAATAGGATCAGTAAATCCAAAGTTAACAGGAGATA<br>ATCTTATAATGATTAAGAAAATAACTCGATTCACTGTCAGTTTTGTTC<br>CACAAATTTGGATTATCGTTTTGCTCATTCTGAGTTCCAGCTCAAGTC<br>ATGGGGAAACTATTTGTGAAGCCAAATATCGAAATATACATCCGGAA<br>CATTCGATGTGCAAGGCTAGGAATAATACCTGCAAATTTAGGCAAGG<br>ATTGGAGGAAAACGCTAAGGAACTTGTGGACTTTCATAACCGAATCC<br>GAAAGTCGATACGACAGATTATTGGAAACAACTTTTCCGAAGGGAA<br>AAGTATGAAAAATATGCAATGGGATCACGAGCTCTATCTCATAGCTC<br>AAAAACACGTGCTTCAATGCACAGGATTACCCGATTGCTACCAGTGT<br>CATCAAACAGATAGATTTTACGTGGAACAAAATTTTGCGGTCAAAAC<br>CTTCAATTCTGTTAAAGAAAGCTTCAACGGAACCATTGAACGGTTTA<br>AAGCAGTTATAAAAGAATGGGCTGAAGAACTAAGCGAGTATGGTCC<br>AGAGGTAGTTAACAAGTTCCATTTCACTGGGCTTCCTACTAATTGGA<br>CAAATATATTTCGCGCGACAACTAATAAAGTGGGGTGTGCGTCGATA<br>GCTTATGACACGCAGAAAGAGGGCACTTTTACAGAGGTTTACGTATG<br>TAATTACGGTCCTGCAATTCTTACAGAGAACGAAGTTATTTACGAAC<br>CAGTATATACAAATTGCAACAATGGTTTATGTAAAAATCAGTTCTTG<br>GAGAAATATGGAAACAGCAACTTCTATCGTTCCAATAGACCGCCCGA<br>GAGAGGAAAACGTAGCGGTGCACAGTTGCAACATAATAATGGACGT<br>CTATCTCGAAATTCAGAAGAGGAAGAGTGCCCAGCTAAGTATCGAA<br>ATATTACAGTAGATCATTCCTATTGCAAACCAGATGTTGAAGGATGC<br>GTATTTAGCAGAAAATATGTCAGATACAGAAAGAAACTCTTAGATAC<br>TCATAACGAAATTCGTAATTCAGTTGAAGCTTATGCAGGTTGGGCTT<br>ATGGTACTGCAACGAACATGCGAGTAATGGAATGGGATGATGAACT<br>GTATGATATTGCTAAACGCTATGTCATTCAATGTCTTGTGGAACCAG<br>ACTGTCATTTATGCCACCAAGCAGCTGACTTCCCTGTGGAGCAGAAC<br>TTTGCTGTTAAAGTTATAAGCTATAATGGACCCCAATCACAAGAAAG<br>ATTTGGAGCAGTAATTAAGGGATGGGCTATGGAATTAAAGAATTTTG<br>CACAGAGTGATGTGGAGCATCTCTCCAAGGAAATTATACAAAGAGA<br>TGATAAAAACTGGATAAATGTATTTCGAGCGAGTGCATGGAAGGTA<br>GGATGTGCATCAATATCATTTGAAACGTCACCAAAACCAGATCTTAT<br>TGTGAAGGAAGTGTATATATGCAATTATGGCCCAGCTGAATTATCTG<br>AAGGTGAAGAAGTATATCAAATTGGAGAGTCTTGTAGCAACTGCGA<br>CCCAGGGTTTGATTGTGATGAAAAATACCCTAATTTATGTAGCAAAA<br>AAGAGAAACCTGAATCCAACGATGATTGGCTAACTCAAACGATGGC<br>AGCAAAGACAACTGCAGAAACTACAGCTAGCAAAAATTCTATTAGG<br>ACTGAAAGTGATATCTTTGCAGACGAGGTTGATGGATCTACTGTTGG<br>TTCAGTGAAGACAAGTGTAAATATAACCACTCCCACCATGACAACTG<br>CAATTACAACCCCTTCAGCTCCAACCACCACAACAATTACAGTTTCA<br>CCAGTGCCAACTGTATCCCTTAGCACAGAGAACCCACACGCAACTGA<br>AATGCCTGTTGAGACTAGCACAGTATCTGCTGTCACAAGTAAGCAAA<br>TTGTGTTATATAATAATAAAACAAAAACTTCTAAAGGCAACATAGTG<br>TGGAACTGCACCTTGCGGTTTTTCGATGATGAGGAATGTAGACGTGA<br>GCAACAGTGTTTTCAAGCATGGACTATGTCAAATGATGGAAAACAGC<br>CGTTTATGGAAATTGATATTCCAGAAAACCGGAGAACAGCCTTATTG<br>TTTCTTCATCAAATTTATATTGAAAAACCTTCTTGTTTTACTTTTACCT<br>ATATGAAAGATGGGCTCAGCAGATTGTCTTCTCTTGACAGCACTCTTT<br>ACGGTATTGTTGTTAGGACAGAAAGCAACGAACATGTAATGGTGGAT<br>GTGAACGGTAATGTTCCTGTCTGGAATTCTCTAGCTGTGGATATTCCT<br>TGGGAGAAAGTTTTTGTCCAGGTGGGAGTTGCTGTTAAAACATATGA<br>TGGAAGGGGTGAGCAGCACATTCAAATAAAGGATTTACTAATCCATG<br>TTGGACCATGTACTGCATTG

TatOthCRI02	XP_013781883.1	1 e-119	PF00188,<br>PF08562	--	CRISP	MAILGKNLFIKPQLFNSAIVEICLILLVSAYPRLQRPKLYG<br>DAIPLRDLDPTHFKTRRKIVLLHNSYRARVDPPASNMLA<br>MSWHEEAAKDAQRWAEACELLVHDSPTGRWVNNYGS<br>CGQNIFVANMKVSWSFALKAWNVERYDFEYGSNKNIPS<br>VVGHYTQMVWYKSHKVGCGFHYCGENRVKKPFYNYV<br>CNYCPIGNDPATFNEPYGIGKPCSKCPGKCKYKKLCTNG<br>CRYGDSWSNCAELNATWSNWLCSDPKQQRYHSCMGT<br>CRCGSEFIK	ATGGCCATCTTAGGAAAGAATTTATTTATCAAACCACAACTTTTTAAT<br>TCTGCGATTGTAGAAATATGTTTGATTCTACTGGTCTCTGCCTACCCT<br>CGGCTCCAACGTCCTAAACTATACGGCGATGCCATACCGCTTCGTGA<br>TTTAGATCCTACTCATTTCAAAACTCGAAGAAAGATTGTTCTTCTGCA<br>CAATTCTTATCGCGCAAGAGTGGATCCTCCAGCAAGTAATATGTTAG<br>CAATGTCATGGCATGAGGAAGCAGCAAAAGACGCTCAGCGGTGGGC<br>GGAAGCTTGTGAATTACTTGTTCATGACAGCCCAACTGGTCGTTGGG<br>TGAATAATTATGGATCCTGTGGGCAAAATATTTTCGTAGCAAATATG<br>AAAGTGAGTTGGTCTTTCGCTCTAAAAGCTTGGAATGTGGAAAGATA<br>TGATTTCGAATACGGCAGCAATAAAAACATCCCATCTGTGGTGGGAC<br>ACTACACTCAGATGGTATGGTATAAATCTCACAAGGTAGGATGTGGA<br>TTCCATTACTGTGGAGAAAATAGGGTGAAAAAGCCTTTTTACAATTA<br>CGTATGCAATTACTGTCCAATAGGTAATGATCCTGCTACATTTAATG<br>AACCATACGGAATTGGAAAACCTTGTTCAAAATGTCCAGGAAAATGC<br>AAATATAAAAAACTATGCACTAATGGGTGTAGATATGGCGATTCTTG<br>GAGTAACTGTGCAGAACTAAATGCAACTTGGAGCAACTGGTTATGCA<br>GTGACCCTAAACAACAACGCTATCATTCCTGCATGGGTACTTGTCGT<br>TGTGGATCTGAATTCATCAAATGAATTATAAAGCACTTCAAACAACG<br>AGAACGCGAC
TatOthCRI03	XP_015929114.1	3 e-66	PF00188	--	CRISP	MLFLLAFLVPLFLKDWCYGKESPVGITAWKSEFDNMES<br>NYVTLDYAVSNKSRRGATNMGVPIPLSDEEKVEIVSTH<br>NKYRSQVFPPAANMRYMKWNDEVADSAQTWANKCVF<br>KHGRPSVSKFKGAMGQNIYKGSNAPLSFYMSLWYDEV<br>HDFDMRLKQCNGSKCGHYIQIAWAESFLIGCGHTKCEG<br>KRGPFNIFVCHYHKPFEKEDPYIIGRPCSMCNVETGGFC<br>YNNMCVSKEDCERESWPCECNLKCHNCGVLNKSTCSC<br>ECPPGWDFQDCSQQCIDEDSWCGKEGGYQGWVGCAM<br>LEDNGFCRKMCGICEDITKDNEHMTCCEGKRCETGYVL<br>NLVNNNCSCQLLCPGPECFTIPDSGHALQGRYYVLLTV<br>MISMCIVLYSCM	ATGCTGTTTCTTTTAGCATTTCTGGTGCCATTGTTTTTAAAAGACTGG<br>TGTTATGGGAAAGAAAGTCCAGTTGGTATTACTGCGTGGAAAAGTGA<br>ATTTGATAATATGGAATCTAATTATGTGACATTAGATTACGCAGTAT<br>CTAATAAGTCGAGAAGAGGGGCCACTAATATGGGAGTACCTATACC<br>ACTTAGTGATGAAGAGAAGGTAGAAATAGTCAGCACCCACAACAAG<br>TACAGAAGTCAAGTGTTTCCACCAGCTGCCAATATGAGATACATGAA<br>ATGGAATGACGAAGTAGCCGACTCAGCTCAAACCTGGGCGAACAAA<br>TGCGTTTTCAAACATGGCCGTCCCTCAGTGTCGAAGTTTAAAGGAGC<br>GATGGGTCAAAACATATACAAAGGAAGTAATGCGCCTTTGTCATTCT<br>ACATGTCACTCTGGTATGATGAAGTACACGATTTTGATATGAGGCTA<br>AAACAATGTAATGGTAGCAAATGCGGGCATTATATTCAGATTGCATG<br>GGCTGAATCTTTTTTAATAGGCTGTGGTCACACTAAATGCGAAGGAA<br>AACGGGGTCCTTTTAACATTTTTGTATGCCATTACCATAAACCTTTTG<br>AAAAGGAGGATCCATATATCATTGGTAGGCCATGTTCTATGTGCAAT<br>GTAGAAACAGGAGGATTTTGTTACAATAATATGTGTGTCTCTAAAGA<br>GGATTGCGAAAGAGAGAGTTGGCCTTGTGAGTGCAATTTGAAGTGTC<br>ACAACTGTGGTGTGCTTAACAAATCAACGTGTTCCTGCGAATGTCCA<br>CCAGGGTGGGATTTTCAAGATTGTTCGCAACAATGTATAGACGAAGA<br>TAGTTGGTGCGGAAAGGAAGGTGGTTACCAAGGTTGGGTTGGTTGTG<br>CAATGTTGGAAGATAACGGGTTTTGTAGAAAGATGTGTGGAATTTGC<br>GAAGACATCACAAAAGACAATGAACATATGACATGCTGCGAAGGTA<br>AAAGATGCGAAACTGGATATGTCCTAAATCTCGTTAATAATAACTGC<br>AGTTGTCAGCTGTTATGTCCGGGACCAGAATGCTTTACAATACCGGA<br>CAGCGGGCATGCACTGCAAGGAAGATATTATGTGTTACTTACTGTAA<br>TGATATCAATGTGTATAGTTTTGTATAGCTGTATG

TatOthCRI04	JAV45657.1	3 e-16	PF00188	--	CRISP	MQATMKLMLGITSLLFAVPSILCLTCSQSSYGNYDETSL<br>ESTRDETIPIDWWKILKINPGFAERKSIIVQKECPEIYQRF<br>SVNHTFCKEAAEGIITEGVTDEDKNVILDIHNTLRSKLAS<br>GEEARYRQLPSAANMMQMEWDDELAAVAQAHSNLCK<br>FEHDESDQRAVENFRVGQNLLLSTSPNKNWNSSQRWY<br>KEEVCFFFPEYINPFQFQSDFGHFSQVTWATTWKVGCGF<br>TAYQEGSGTRTLYTCNYGPGGNVDGGVQYIVGEPCSQC<br>PDNTECSTQYPGLCKSLTENGPQIPRPAADEYILFCDFSE<br>EDPEECININVEGSKNFSTGHIYSGNYKTVILEEGEHITID<br>FGEAQNDGGFCPFLYSRFGPNNAKESNGAVMEFLFRSP<br>NTVPQPPTKMPPRGPNFSTFGIYMMYNGKLKTTVTLRA<br>EEGAAPQYFDIKYWGIRRGNCKTNF	ATGCAAGCCACCATGAAGTTGATGTTGGGAATAACTTCACTGCTTTT<br>CGCAGTTCCCTCCATTTTGTGCCTTACATGCTCTCAGAGTTCATATGG<br>AAATTACGATGAAACCTCCCTAGAATCCACAAGAGATGAGACGATTC<br>CCATTGATTGGTGGAAAATACTAAAAATCAACCCTGGGTTTGCTGAA<br>AGAAAATCAATTATAGTTCAGAAAGAATGTCCAGAAATCTACCAAC<br>GTTTCAGTGTTAATCACACTTTCTGTAAGGAAGCAGCAGAAGGTATT<br>ATAACAGAAGGAGTGACTGACGAAGACAAGAATGTTATTTTGGACA<br>TACATAATACATTAAGAAGCAAGCTGGCTAGTGGAGAAGAAGCACG<br>TTATCGTCAATTGCCTTCTGCAGCAAACATGATGCAAATGGAATGGG<br>ATGATGAATTAGCAGCTGTAGCTCAAGCCCATTCGAACCTTTGCAAG<br>TTTGAACATGACGAGAGTGATCAGAGAGCTGTAGAAAACTTCCGGGT<br>TGGACAAAATTTGTTGCTATCAACTTCTCCAAACAAGAACTGGAATT<br>CTTCTCAGAGGTGGTATAAAGAAGAAGTGTGCTTTTTCTTTCCTGAAT<br>ATATTAACCCATTTCAATTCCAAAGCGATTTTGGACATTTCTCTCAGG<br>TAACATGGGCAACAACATGGAAGGTAGGATGTGGATTTACAGCTTAC<br>CAAGAGGGTAGTGGAACGAGGACACTCTATACATGCAATTATGGAC<br>CTGGGGGTAATGTTGATGGAGGTGTACAATACATTGTAGGAGAGCCT<br>TGTTCTCAATGTCCTGACAACACAGAGTGTTCGACTCAGTATCCAGG<br>ACTTTGTAAATCTCTAACCGAAAATGGACCACAGATACCAAGACCAG<br>CAGCAGATGAATACATACTGTTCTGCGATTTTAGTGAGGAAGACCCA<br>GAAGAATGTATTAACATTAATGTTGAAGGTTCAAAGAACTTCTCAAC<br>AGGACACATATATTCTGGTAATTACAAAACAGTCATTCTTGAAGAAG<br>GCGAGCATATAACCATTGATTTCGGGGAAGCACAGAATGATGGGGG<br>TTTCTGCCCATTTCTCTACAGTCGTTTTGGTCCTAACAATGCTAAAGA<br>AAGCAATGGAGCTGTAATGGAATTCCTTTTCAGAAGTCCAAACACTG<br>TACCACAGCCTCCAACAAAAATGCCTCCACGTGGTCCTAACTTTTCC<br>ACATTTGGTATATATATGATGTACAATGGCAAGCTTAAGACGACTGT<br>AACACTTAGAGCAGAAGAGGGCGCTGCACCGCAATATTTTGACATTA<br>AGTACTGGGGTATTCGCAGGGGAAATTGCAAAACTAATTTCT
TatOthCRI05	JAV45657.1	4 e-83	PF00188	--	CRISP	MVKLMNLHHYYSIMVFLIFISFLLCGVSCSNKLPEDEDE<br>QRYIFINKKKSCIIGQENDCNSNMFRDIFLHHQNTERKTI<br>GAKLNMNLAASNMLQMEWDQTLADGAWKYVETCFL<br>DYPTYVGIEVCREASIVTTFDGGVNYYRKTYKEDYPHV<br>VDRILNAIANWTNYINDYDKSKLKVLHLYQESGAAEDS<br>WSQVVRATTWKFGCALADGRTENTRLDEFVEVIACFY<br>GNTKLQKGDEIFKPGKPCSSCPLGTHCNEYLCEVEPGQC<br>PVPGNDFALEGQHCHKIDIVEIQEDSEESGEILWECNLQK<br>YGEQCEILPSCSPFWNIDLIGNFKTITVKGNCVSANVFLE<br>EISVVVPSCFRFQYIKERNPTRRFHTMVMGFAFNLNSGE<br>SINIMKNEDANSWADVNLDIPWTNTVIQVGILVRSYSDN<br>IFQQHVTVGNFSVFEGNCS	ATGGTTAAGTTGATGAATCTTCATCATTATTATTCAATTATGGTGTTC<br>CTTATCTTTATAAGTTTCTTATTGTGCGGAGTTTCATGTTCCAATAAA<br>CTGCCAGAGGATGAGGATGAACAAAGATACATATTTATCAACAAAA<br>AAAAGTCATGCATCATTGGTCAAGAAAATGATTGTAATTCAAACATG<br>TTTCGAGACATATTTTTACATCATCAGAACACTGAGCGTAAAACAAT<br>TGGTGCTAAACTAAATATGAATTTAGCTGCATCAAATATGTTACAGA<br>TGGAATGGGATCAGACACTTGCAGACGGTGCATGGAAATATGTTGA<br>AACATGCTTTTTAGACTACCCAACTTACGTCGGGATTGAAGTTTGTA<br>GAGAAGCAAGCATAGTAACTACTTTTGATGGTGGTGTTAATTACTAC<br>CGGAAAACTTACAAAGAAGATTATCCACATGTTGTAGATCGTATCCT<br>TAATGCAATTGCTAATTGGACTAACTACATCAATGATTATGATAAAA<br>GCAAACTGAAAGTACTTCATCTCTATCAGGAAAGTGGCGCTGCTGAG<br>GATTCGTGGTCCCAGGTAGTTCGAGCAACCACATGGAAATTCGGATG<br>TGCACTTGCTGATGGTCGTACTGAAAACACTAGATTAGACGAATTCG<br>TAGAAGTTATAGCTTGCTTTTATGGAAACACAAAGTTGCAAAAAGGT<br>GATGAGATATTTAAACCAGGAAAACCCTGTTCGTCTTGTCCTCTTGG<br>AACACATTGCAACGAATATTTATGCGAAGTAGAGCCAGGCCAGTGTC<br>CTGTACCAGGCAATGATTTTGCACTTGAAGGACAACATTGTCACAAA<br>ATAGACATAGTTGAGATTCAAGAAGATTCAGAAGAGAGTGGAGAAA<br>TTCTTTGGGAATGCAACTTGCAGAAATATGGAGAACAATGTGAAATC<br>TTACCTTCATGCTCACCATTCTGGAACATTGATCTAATTGGGAATTTC<br>AAAACTATTACAGTTAAAGGTAACTGTGTATCAGCCAATGTATTTTT<br>AGAAGAGATCAGCGTCGTAGTTCCATCTTGCTTTAGATTTCAATACA<br>TTAAAGAAAGGAATCCAACGCGACGATTTCATACTATGGTAATGGGA<br>TTCGCCTTTAATTTAAACTCTGGAGAAAGCATTAATATAATGAAGAA<br>TGAAGATGCAAATAGTTGGGCTGATGTCAATTTGGATATCCCATGGA<br>CGAATACTGTAATACAGGTTGGAATACTTGTCCGTTCGTACTCCGAT<br>AACATTTTCCAGCAGCATGTAACTGTAGGGAATTTCAGCGTTTTCGA<br>AGGAAATTGTTCC

TatOthCRI06	JAV48225.1	5 e-145	PF00188	--	CRISP	MASVIITILVLWVTTIQSFEANDECDERYSSITQEHTMCK<br>SINENCHFLRHGGKTYEQQLLRTHNLIRNSIWKYVGKN<br>YPLATNMEIMQWDDELYEIARMHSLQCVEQPDCDLCH<br>QIGYFPVEQNFAVKTYKKSEVANNGPVKRFQTVIKEWA<br>AELKQYDPSIVNDFTVTEELPTNWTNILRANTSFVGCAS<br>MNFYTDETDVFTEVYVCNYGPAKLTTGEEIYKTGGKPC<br>SDCEDDGICDEEFKNLCVPADLELNITIVIPEEEDTEIWLG<br>EGNYNGTSLSEEIEGTTSPNEFTTAESAKTFLYRQTGTPE<br>ETHTAIGEENSSVVFTETAPEETSIVEFTTELSNEPVTTEV<br>EESGERRKRKVHPLISRLLRYSNKRRKRHQEHSLFPMN	ATGGCGTCAGTGATTATTACCATTTTAGTTCTATGGGTGACAACGATT<br>CAAAGCTTTGAAGCCAATGATGAATGTGACGAAAGGTACAGCAGCA<br>TTACTCAGGAACACACCATGTGCAAAAGTATAAATGAAAACTGCCAT<br>TTTCTTAGGCACGGTGGTAAAACATATGAGCAGCAACTACTTCGCAC<br>TCATAACTTGATACGAAATTCAATTTGGAAGTATGTTGGAAAAAACT<br>ACCCTTTGGCAACTAATATGGAAATAATGCAATGGGATGATGAACTG<br>TATGAAATAGCCAGGATGCATAGTCTTCAATGTGTTGAGCAACCTGA<br>CTGTGATCTATGCCACCAGATCGGTTACTTTCCTGTGGAGCAAAATTT<br>CGCCGTTAAAACGTATAAAAAGTCAGAGGTTGCTAACAATGGTCCCG<br>TCAAACGATTCCAAACTGTCATTAAAGAGTGGGCAGCCGAACTGAA<br>ACAGTATGACCCTAGTATCGTTAATGATTTTACAGTTACCGAAGAAC<br>TTCCCACTAATTGGACTAATATCCTGCGTGCTAATACATCGTTCGTTG<br>GATGCGCGTCCATGAATTTCTATACTGATGAAACAGACGTCTTCACA<br>GAGGTATATGTGTGCAATTACGGACCTGCGAAACTTACTACAGGAGA<br>AGAGATATATAAAACAGGTGGTAAACCTTGCAGCGATTGCGAAGAC<br>GATGGAATATGCGATGAGGAGTTTAAAAACCTATGTGTTCCAGCTGA<br>TTTAGAGCTAAATATCACAATAGTTATTCCTGAAGAAGAAGACACGG<br>AAATTTGGCTAGGTGAGGGAAATTACAATGGAACCAGTCTAAGTGA<br>AGAAATCGAGGGAACCACGTCACCTAACGAATTCACTACGGCAGAA<br>TCAGCAAAAACGTTCCTTTATAGACAAACTGGAACTCCTGAAGAAAC<br>ACATACTGCAATAGGCGAGGAAAATTCTTCTGTGGTATTCACAGAAA<br>CAGCTCCAGAGGAAACTTCAATTGTTGAGTTTACAACAGAACTTTCT<br>AACGAACCTGTCACTACAGAAGTCGAAGAAAGTGGAGAGAGGCGTA<br>AAAGGAAAGTTCATCCCTTGATATCAAGATTGCTTAGATATAGTAAC<br>AAACGCAGAAAGAGACATCAAGAACATAGCCTATTTCCTATGAAT
TatOthCRI07	API81352.1	0	PF00188	--	CRISP	MNFLLLSCLVLFSCQAVFAQTCPALYLRFSKDHTYCRHS<br>TCQTEKSGVSEKDKEIIVNMHNEFRSKIAMGKETTPMQ<br>QPPAANMMQISWDEELAAVAQAHANGCKFDHDTAPQR<br>QVENFNVGQNLYITMMSKRIDWRKAALWYTSEVKDFY<br>PQYREPFVFGTYGHFSQVVWAETWKVGCGMAMYYDQ<br>SDNMDKVLYTCNYGPAGNMQGAAMYIKGQPCSQCPK<br>NTKCSDEYEGLCKPITKDGPQNDIAKSSNDFIFYCEFSKN<br>DPSECSKVKVEGTKGFETRKIYSGIYKSVILNGGESITIKL<br>GKAQDAGGICPFIYGSFGPNKDGDAKMSAVSFGFSAPGI<br>IFGDPIKIEQGGSAFWTIGMHMQFDQEMESTIKLEAYPG<br>ATPQYFNVKAFGIGKGKCPKL	ATGAATTTCCTACTGCTGTCCTGTCTTGTCTTGTTTTCTTGCCAAGCTG<br>TTTTTGCTCAAACCTGCCCTGCGTTGTACCTAAGATTCAGTAAGGATC<br>ACACATACTGTCGACATTCAACATGCCAAACTGAGAAAAGTGGTGTT<br>TCTGAAAAGGACAAGGAAATAATTGTAAACATGCATAACGAATTCA<br>GAAGTAAAATCGCTATGGGAAAGGAAACCACACCCATGCAGCAACC<br>CCCTGCAGCTAATATGATGCAAATATCATGGGATGAAGAGCTGGCTG<br>CAGTTGCGCAAGCCCACGCTAATGGATGTAAATTTGACCACGACACT<br>GCACCTCAACGACAAGTAGAAAACTTCAATGTTGGGCAAAATCTGTA<br>TATAACCATGATGTCAAAGAGGATAGATTGGAGGAAAGCTGCTTTGT<br>GGTACACTTCTGAAGTGAAAGATTTCTATCCACAATATAGAGAACCA<br>TTTGTATTTGGCACATATGGGCATTTCAGTCAGGTAGTCTGGGCTGA<br>AACATGGAAAGTGGGTTGTGGAATGGCTATGTATTATGACCAAAGCG<br>ATAATATGGATAAAGTGCTATACACCTGTAATTATGGACCTGCAGGA<br>AATATGCAAGGGGCTGCAATGTATATTAAAGGGCAACCATGCTCTCA<br>GTGTCCAAAGAATACAAAATGCTCAGATGAATATGAAGGACTTTGCA<br>AACCAATAACCAAAGATGGCCCCCAAAATGATATTGCCAAATCTTCA<br>AATGATTTCATCTTTTACTGTGAGTTTAGTAAGAATGACCCAAGTGA<br>GTGCAGTAAAGTCAAAGTTGAAGGAACAAAAGGATTTGAAACAAGG<br>AAAATATACAGTGGCATATACAAGAGTGTTATTCTTAATGGAGGAGA<br>ATCCATTACCATAAAATTAGGAAAAGCTCAAGACGCTGGAGGAATA<br>TGCCCTTTTATTTATGGATCTTTTGGCCCCAACAAAGATGGTGATGCT<br>AAAATGTCTGCGGTATCTTTTGGCTTTTCTGCACCTGGAATCATATTT<br>GGAGATCCTATTAAAATAGAGCAAGGTGGTAGTGCTTTCTGGACAAT<br>AGGAATGCATATGCAGTTTGATCAGGAAATGGAGAGCACTATTAAG<br>CTAGAAGCATATCCTGGAGCAACTCCTCAATATTTCAATGTAAAAGC<br>ATTTGGAATTGGGAAAGGAAAATGCCCAAAACTT

TatOthCRI08	XP_015929114.1	4 e-83	PF00188	--	CRISP	GLVSKVAVERPMNIMVKETVIKLSEEERFYIAQIHNLFRS<br>NATPPAANMRYVQWDETLEKTAQAWASLCVMRHGTP<br>ASPRYHDSYGQNLYYGPSSSTHRAMWLWYNELHDYDH<br>RNAKCALGRECGHYIQMMWWKTKLIGCSLAQCGKSEF<br>FFVCHYYPQFFNNTAPYTVGEPCSLCNIGHGGLCNNSLC<br>VTKTACEKYGLECECHLKCHNCGRFNNSTCSCDCVDG<br>WDFLDCSTQCKDESEHCGKDDGYPSMLNCPMYKGQVK<br>NEHCRKMCQACESVNSSYLRRTCCDGIVCSPGYVIDLE<br>NRPCSCRLLCPGPECYSSFGKIWQVDLFLFEIGICAVLCM<br>IWSR	GGTTTAGTTTCGAAGGTTGCGGTAGAAAGACCAATGAATATAATGGT<br>TAAGGAGACCGTTATTAAACTTAGTGAAGAAGAGAGATTTTATATTG<br>CCCAAATCCATAACTTGTTTCGAAGCAACGCCACACCACCAGCAGCT<br>AATATGAGATACGTGCAGTGGGATGAAACATTAGAGAAAACGGCAC<br>AAGCTTGGGCAAGCTTGTGTGTGATGCGCCATGGCACTCCAGCATCT<br>CCTAGGTACCACGATTCCTATGGGCAGAATTTATATTATGGACCCAG<br>TAGCTCCACTCACAGGGCAATGTGGTTGTGGTATAACGAATTACATG<br>ATTATGATCATCGCAATGCTAAATGTGCTCTAGGAAGAGAATGCGGG<br>CACTATATTCAGATGATGTGGTGGAAGACAAAATTGATTGGTTGTTC<br>ATTGGCCCAATGCGGAAAATCTGAATTCTTTTTCGTATGTCATTACTA<br>TCCTCAGTTCTTTAATAACACAGCACCTTACACAGTTGGGGAACCTT<br>GCTCCCTTTGCAATATTGGTCACGGAGGATTATGTAATAACAGTTTGT<br>GTGTAACGAAGACAGCGTGTGAGAAATATGGTTTAGAATGTGAATG<br>CCATTTGAAATGTCATAATTGTGGTCGATTTAATAATTCAACGTGCA<br>GCTGTGACTGTGTGGATGGATGGGACTTTCTAGACTGTTCAACACAA<br>TGCAAGGATGAGAGTGAACACTGTGGAAAAGATGATGGCTATCCAA<br>GTATGTTAAATTGTCCAATGTACAAAGGTCAAGTCAAGAATGAACAT<br>TGCAGAAAGATGTGCCAAGCATGTGAAAGTGTTAATTCAAGTTATCT<br>AAGACGAACATGCTGTGATGGCATAGTTTGCTCTCCTGGGTATGTTA<br>TTGATTTAGAAAATCGTCCCTGTTCTTGCAGGCTTCTATGTCCCGGAC<br>CAGAATGTTATTCTTCATTTGGGAAGATTTGGCAAGTGGATTTATTTC<br>TATTTGAAATTGGAATATGTGCAGTACTATGTATGATATGGAGCAGA

Protease inhibitor

ID	ID/Reference protein	e-value	PFAM domain	MW (Da)	Sub-classification	Amino acid sequence	Nucleotide sequence
TatPInSrp01	JAT91131.1	4 e-58	PF00079	--	Serpin-like	FQGGEEVVSVQMMETRNTVFYGNFSEYGFEAVKMPYK<br>GEDISMLVLLPFERNGLSGVEQELNSSKLDDVISKMKKG<br>KFNVKIPKFRLKDRKHLRKNLNFLGVKSAFTNKANFTGI<br>SGEGNLEVSKIIYRAVVEVNEKGSEAAGATAEFARFGKS<br>RGRPKPGNFYVNHPFVFFIREDRTGMILFYGRVNKL	TTTCAGGGCGGCGAAGAGGTAGTTTCAGTTCAAATGATGGAAACAA<br>GGAATACAGTTTTTTATGGAAATTTCAGCGAATATGGTTTTGAAGCT<br>GTGAAAATGCCATACAAAGGTGAGGATATCAGTATGTTAGTGCTTCT<br>ACCATTTGAAAGAAACGGCCTAAGTGGGGTAGAACAGGAGTTGAAT<br>TCCAGTAAATTAGACGATGTTATCTCCAAGATGAAAAAGGGAAAGTT<br>TAATGTAAAGATCCCAAAATTTAGACTGAAAGACAGGAAACACCTA<br>AGAAAAAATCTAAACTTCTTAGGGGTGAAAAGTGCATTCACAAATA<br>AGGCAAATTTTACTGGAATTTCTGGAGAAGGAAATTTGGAAGTGTCA<br>AAGATTATTTACAGAGCTGTGGTTGAAGTCAATGAAAAGGGTAGCG<br>AAGCAGCAGGGGCCACTGCCGAATTTGCCAGGTTTGGGAAATCCAG<br>AGGTAGACCAAAGCCGGGAAATTTTTATGTAAACCATCCTTTTGTCT<br>TCTTCATTAGAGAAGACAGGACTGGAATGATTCTTTTCTATGGAAGG<br>GTCAATAAATTGTAA

TatPInSrp02	CAX51426.1	9 e-89	PF00079	--	Serpin-like	MLRTMPSKDQSLLKDTEWDVSVKKKPTMWKTWVRCSI<br>IFFLMLVVLAVVLLVMCGYVYRHDFLGFIQLKPFTEPTS<br>HAFRDERIHAGTVRMVARWLNHANQNENAVWSPLTVI<br>LGLSLLLEGTTGQTAYEIKSTMGWIGIDKSYVRSYLSRVI<br>TRLNNLQHLDVIVANRMFLHDSYDVTSAYKNILNQHYQ<br>VSIDHLDFTNANALQSINKWGLEESRGQIKELIPLDVIDP<br>TTKMVLSTLLTFKGEWKLPFSPNRTKKAEFTSISGKKRQ<br>VDMMYQTGKFMYGEFPAHGFSALELPYSEPNMTMVVL<br>LPRSLSLLKAMLKEQDGTMLMNLSSKMTRTDVEVCIPK<br>FTIEKGTTIENQLSSLGIRRLFDPSFANLELISPNSELYVQ<br>KITHRVKIEVFESGTSGQASSSSILEGRSLTSSFCADHPFIF<br>YVEDKETNSVVFWGEFNGPED	ATGCTAAGAACGATGCCGTCTAAAGATCAGTCGCTGTTAAAAGACAC<br>AGAATGGGATGTAAGTGTCAAGAAAAAGCCAACTATGTGGAAAACG<br>TGGGTGAGATGCAGTATAATATTTTTCTGATGTTAGTCGTGCTGGCC<br>GTTGTATTGTTAGTTATGTGCGGTTACGTATATCGACACGATTTCCTT<br>GGATTCATTCAATTGAAGCCTTTCACAGAACCGACTTCTCACGCCTTT<br>AGAGATGAACGTATTCACGCTGGAACAGTGCGTATGGTGGCTAGATG<br>GCTTAATCACGCGAACCAGAATGAAAACGCAGTGTGGTCACCCCTTA<br>CTGTCATATTAGGCTTAAGTCTATTGCTAGAAGGCACAACTGGACAG<br>ACTGCGTACGAAATCAAGTCAACTATGGGATGGATTGGTATAGACAA<br>AAGCTACGTACGTTCTTACCTTTCACGGGTGATAACACGCTTGAACA<br>ATTTGCAGCACTTGGATGTAATAGTCGCTAACCGGATGTTCCTTCATG<br>ATTCCTACGACGTCACTTCCGCGTATAAAAATATTTTGAATCAACACT<br>ACCAAGTTTCCATCGATCACCTAGATTTTACTAACGCTAATGCACTGC<br>AAAGTATTAATAAATGGGGATTAGAAGAAAGCCGCGGACAGATCAA<br>AGAATTAATTCCATTAGATGTTATTGACCCAACCACTAAAATGGTAC<br>TATCGACGCTTCTTACTTTTAAAGGTGAGTGGAAGCTACCTTTTTCTC<br>CAAATCGTACCAAGAAGGCAGAGTTTACATCAATAAGCGGAAAGAA<br>ACGTCAAGTCGACATGATGTACCAGACGGGAAAATTTATGTACGGTG<br>AATTTCCGGCTCACGGATTTTCTGCGCTGGAATTACCATATTCTGAAC<br>CTAATATGACCATGGTGGTTTTGTTACCTCGGAGTTTGTCTCTTCTGA<br>AAGCGATGCTAAAGGAACAAGATGGGACAATGCTAATGAACCTGAG<br>CTCTAAAATGACGAGAACTGACGTGGAAGTGTGCATACCCAAATTTA<br>CGATAGAAAAAGGGACAACGATAGAAAACCAGCTGTCATCTTTGGG<br>AATCAGGAGATTATTTGATCCATCATTTGCAAATCTAGAATTGATAT<br>CACCTAATTCTGAACTGTATGTGCAGAAAATAACCCACAGAGTAAAA<br>ATAGAAGTATTTGAAAGCGGAACTTCAGGTCAAGCTTCTTCATCGTC<br>CATTTTGGAAGGACGTTCACTGACGTCATCATTCTGCGCGGATCACC<br>CGTTTATATTCTACGTCGAAGACAAGGAAACTAATTCTGTTGTGTTTT<br>GGGGCGAATTCAACGGGCCTGAGGATTAA
TatPInSrp03	JAT91147.1	0	PF00079	--	Serpin-like	MLQAFFALFLLPLAHGLGFTRAIPSSGITALIEGSNNFGFE<br>LYKAIQSDDNLVFSPWSISVGMAMLYLGARNYTAREIES<br>VLYQNDQGVSAREMMVAYGQLDRLMKRKAQLELTKF<br>NAAVIQQGSPVSETYRHRLFHYFNSVLYDLDMKQNGKL<br>VKDWINILAEIKTGGLIKDILTRVPTEDSKLILLSGIHYKG<br>QWVEKFDPEKTTNGKFFNHGNMATTAALMLSTSNYTY<br>YYSYANNLHVLQIPLEGRFAMTFVMPEENATLEEVAQK<br>LNPKLLQKVLEQDASSKMIKLVLPKFKLEVRKDLSEPLK<br>EMGMHTLFSPVNSDLTGIDATGDLFLKDVVHQATVEVN<br>EEGVEAAATTVLGVESRLGAPYISFNRPFMFFVRDLDTR<br>LILFMGHLKDCSEVCGVSNYVQQESQSLFR	ATGCTACAAGCGTTCTTCGCGTTGTTCCTCCTGCCTTTAGCTCATGGC<br>TTAGGCTTTACCAGAGCGATACCATCCTCAGGAATCACAGCTCTAAT<br>AGAAGGCAGCAATAACTTTGGTTTCGAATTGTATAAAGCCATCCAAA<br>GCGATGATAACCTCGTATTCTCACCATGGAGCATCTCAGTAGGTATG<br>GCCATGTTATATCTGGGTGCTAGGAACTATACAGCTCGTGAAATAGA<br>GTCAGTTCTCTATCAAAATGATCAGGGTGTTTCTGCTAGAGAAATGA<br>TGGTGGCATATGGACAGTTAGATCGTCTAATGAAAAGAAAAGCTCA<br>GCTGGAACTCACAAAATTTAATGCTGCAGTTATTCAGCAAGGTTCCC<br>CTGTTTCAGAGACCTACAGACATAGACTCTTCCATTATTTCAATTCCG<br>TTCTTTATGATTTGGATATGAAGCAAAATGGAAAACTGGTGAAAGAT<br>TGGATAAACATCTTAGCAGAAATCAAAACTGGAGGATTGATAAAAG<br>ACATCCTTACGAGAGTCCCTACTGAAGATAGCAAACTAATATTGCTC<br>AGTGGGATCCATTATAAAGGACAATGGGTAGAAAAGTTCGATCCTG<br>AGAAAACCACTAATGGGAAGTTTTTTAACCACGGAAATATGGCCACT<br>ACTGCAGCTTTGATGTTGTCTACTTCTAATTATACGTATTACTACAGC<br>TACGCAAATAATTTGCACGTGCTCCAAATTCCACTTGAGGGTAGATT<br>TGCAATGACCTTCGTTATGCCAGAAGAGAATGCCACTCTCGAAGAAG<br>TAGCACAGAAACTTAATCCCAAATTGCTACAGAAGGTTCTTGAACAA<br>GATGCTTCATCTAAAATGATTAAGCTTGTATTGCCTAAGTTTAAATTG<br>GAAGTAAGAAAAGATCTCTCTGAACCCTTAAAAGAAATGGGGATGC<br>ACACGTTGTTTTCACCTGTAAATTCTGATCTAACTGGGATAGATGCA<br>ACTGGAGACTTGTTTCTCAAAGACGTCGTTCATCAAGCCACAGTTGA<br>AGTAAATGAAGAAGGTGTCGAGGCAGCAGCCACTACAGTACTTGGG<br>GTAGAAAGTAGATTAGGAGCCCCGTATATTTCATTCAACCGTCCATT<br>CATGTTCTTTGTTCGAGACCTTGACACTAGACTCATTTTGTTTATGGG<br>ACATCTTAAGGACTGCAGTGAAGTATGCGGAGTTTCCAACTATGTGC<br>AGCAAGAAAGTCAATCGCTTTTCCGTTAA

TatPInSrp04	KFM76460.1	4 e-120	PF00079	--	Serpin-like	MIWLSLVLAVLVLENAQRYEAKSDILFPTEPQFFLPRFT<br>GDENLKVIEAINDFAFSLYWKLPREGNLLTSPMSLSTAM<br>AMLYYGAQNETKREMSKALGYDGAKLDYDEVHRGFH<br>QVLKSLKNKNAEYDLETANAVLIQKDYPVLASYKSQVE<br>QIYNATIQELDFAQGDIALKMINAWTAWRTRDKIKELL<br>KKPLDSLTRLFLLNAVYFKGTWKTKFDQRITTPDTFYNN<br>GRIPKTVPMMRLKAKLAFGIDRRLKCSILEMPYTGEEVS<br>MVILLPLERDGLPELEKSMSAEVLSNLTSSLRKETVQVTI<br>PRFKLDDEHELKDVLKSMGMPSLFDPYTANLSGISKIEQ<br>LYVTLVVHKTVLDVNEEGTEAAGVTGIAAGVRIGGPYF<br>SAQHPFLIFIRDIRSNMIYFMARVNEL	ATGATTTGGCTGTCATTGGTGTTAGCTGTGTTGGTTTTGGAAAATGCT<br>CAGAGGTATGAAGCAAAATCAGATATCCTGTTCCCAACAGAACCAC<br>AATTTTTCCTACCTCGATTTACTGGTGATGAAAACCTTAAAGTAATTG<br>AGGCTATCAACGATTTTGCGTTCTCTCTATACTGGAAGTTACCAAGA<br>GAAGGAAATCTATTAACATCTCCAATGAGTTTGTCCACTGCAATGGC<br>TATGTTGTATTACGGTGCACAAAACGAAACGAAAAGAGAAATGTCA<br>AAAGCGTTAGGGTATGATGGTGCAAAGTTAGATTATGATGAAGTTCA<br>CCGAGGGTTCCATCAGGTATTGAAAAGCTTAAAGAACAAGAATGCA<br>GAATACGATCTGGAGACTGCAAATGCTGTTCTAATTCAGAAAGATTA<br>TCCAGTGTTAGCCTCTTACAAATCTCAAGTGGAGCAAATATACAACG<br>CTACCATTCAAGAATTAGACTTCGCCCAAGGAGACATAGCTCTTAAA<br>ATGATCAATGCATGGACCGCATGGAGGACTCGCGATAAAATCAAAG<br>AATTGTTAAAGAAGCCACTGGATTCGCTAACAAGGCTATTTCTATTA<br>AATGCTGTTTATTTTAAAGGAACATGGAAAACTAAGTTTGACCAGCG<br>TATCACAACACCAGATACTTTCTACAACAATGGGCGCATACCAAAAA<br>CTGTACCCATGATGAGATTAAAAGCTAAACTGGCTTTTGGAATAGAT<br>CGTCGGTTGAAGTGTTCCATCTTAGAAATGCCATACACGGGTGAAGA<br>GGTCAGTATGGTAATCTTGTTGCCATTGGAAAGAGACGGTCTCCCTG<br>AACTGGAAAAGTCAATGTCTGCAGAAGTGCTATCCAACCTTACATCG<br>TCTCTTAGAAAAGAAACAGTTCAAGTAACGATACCACGTTTTAAACT<br>AGACGATGAACACGAATTGAAAGATGTGCTAAAGAGTATGGGAATG<br>CCATCTTTGTTTGATCCATATACCGCAAACCTATCAGGTATTAGCAAA<br>ATAGAACAGCTGTACGTTACTTTAGTGGTGCACAAAACCGTTTTAGA<br>CGTCAACGAAGAAGGTACAGAAGCGGCAGGTGTTACAGGCATAGCT<br>GCGGGTGTGAGGATAGGAGGCCCATATTTTTCGGCTCAGCATCCATT<br>TTTAATTTTTATTCGCGACATTAGATCAAATATGATTTACTTCATGGC<br>TCGCGTTAATGAGTTGTAG
TatPInSrp05	JAT91131.1	7 e-81	PF00079	--	Serpin-like	MTVDQSCIVYIITILATLIVFSTATSPDNDLTNVTAANND<br>FAFKFLKTIEWKENAFFSPASLFIGLGMLYRGARSNTAK<br>EMRHALSYDLADVDPTNLHRRIRVLLNSLNDNKGQYVL<br>EIANALITQMGYRVRNEYRKALRRYYDTYFKEVDFKSN<br>KEKAIKDINEWVIEKSKGKVPKIVDDLEDDTRLILLNAA<br>YFKGTWKVEFNPTFTSTETFYNYGKDAVTVTMMNTKN<br>RFPYGVFINRGIQALQLPYKGEDIRMVILLPLKLDGLKEL<br>EEILNAEEFNSIIAGMEESLVAVSLPKFKLKDARSLKDNL<br>KDLGMVSAFGNRADFSAISWNNNLLLSDIIHKSVIEVNE<br>EGSEAVSFVAMLMRSKSAADEVDTFIVDGPFLFFIRDNR<br>NGMILFMGRVDKL	ATGACAGTTGACCAAAGTTGCATCGTATATATTATAACAATACTGGC<br>AACACTGATTGTTTTCAGTACCGCAACAAGCCCAGATAATGATCTTA<br>CTAATGTAACAGCTGCCAATAACGACTTTGCCTTCAAATTTCTGAAA<br>ACAATAGAATGGAAAGAAAATGCTTTTTTCTCCCCAGCCAGTCTGTT<br>TATTGGTTTGGGTATGCTCTACCGAGGTGCTCGGTCTAATACAGCTA<br>AAGAAATGCGCCATGCCCTTTCGTATGATCTAGCAGATGTAGATCCA<br>ACAAATCTTCATAGGCGTATAAGAGTGCTACTAAATTCTCTGAATGA<br>TAACAAAGGACAGTATGTACTAGAAATTGCCAATGCCCTCATAACAC<br>AGATGGGCTATCGAGTAAGAAATGAATATAGAAAAGCTCTAAGAAG<br>ATACTACGACACTTACTTCAAAGAAGTGGACTTTAAGTCAAACAAAG<br>AAAAAGCAATCAAAGATATCAATGAGTGGGTTATTGAAAAGAGTAA<br>AGGAAAGGTACCGAAGATCGTTGATGACCTAGAAGATGATACCAGG<br>TTGATTCTTTTGAATGCTGCTTACTTCAAAGGAACTTGGAAGGTTGAA<br>TTCAACCCTACATTCACTTCAACAGAAACGTTTTACAATTATGGTAA<br>GGATGCTGTGACTGTTACTATGATGAATACTAAAAATAGATTTCCTT<br>ATGGTGTTTTCATAAATAGAGGGATTCAAGCTCTTCAGCTACCCTAC<br>AAAGGTGAGGATATAAGGATGGTTATATTGCTTCCTCTAAAACTAGA<br>TGGTTTGAAAGAACTGGAGGAAATTCTGAATGCTGAAGAGTTTAATT<br>CTATAATTGCTGGTATGGAAGAATCACTAGTGGCAGTCTCTCTGCCG<br>AAGTTTAAGCTCAAAGATGCTAGGAGTTTGAAGGATAATTTGAAAGA<br>TTTAGGGATGGTGTCAGCATTTGGAAACAGAGCAGACTTTTCCGCTA<br>TTAGTTGGAATAATAATTTACTGTTATCTGACATCATACATAAATCTG<br>TTATTGAGGTGAATGAGGAAGGAAGTGAAGCTGTCTCATTTGTTGCC<br>ATGTTGATGAGGTCAAAATCAGCTGCTGATGAAGTAGACACTTTTAT<br>TGTTGATGGGCCTTTTTTATTCTTTATTAGGGATAATCGAAATGGAAT<br>GATTTTATTTATGGGCCGTGTTGACAAGTTGTAA

TatPInSrp06	JAT91131.1	3 e-66	PF00079	--	Serpin-like	FKGTWKTRFSRRHTREHLFYNGGEEVVSVQMMETRNT<br>VFYGNFSEYGFEAVKMPYKGEDISMLVLLPFERNGLSG<br>VEQELNSSKLDDVISKMKKGKFNVKIPKFRLKDRKHLR<br>KNLNFLGVKSAFTNKANFTGISGEGNLEVSKIIYRAVVE<br>VNEKGSEAAGATAEFARFGKSRGRPKPGNFYVNHPFVF<br>FIREDRTGMILFYGRVNKL	TTCAAAGGTACATGGAAAACTCGGTTTTCTCGAAGACATACAAGGGA<br>ACATCTCTTTTATAATGGCGGCGAAGAGGTAGTTTCAGTTCAAATGA<br>TGGAAACAAGGAATACAGTTTTTTATGGAAATTTCAGCGAATATGGT<br>TTTGAAGCTGTGAAAATGCCATACAAAGGTGAGGATATCAGTATGTT<br>AGTGCTTCTACCATTTGAAAGAAACGGCCTAAGTGGGGTAGAACAG<br>GAGTTGAATTCCAGTAAATTAGACGATGTTATCTCCAAGATGAAAAA<br>GGGAAAGTTTAATGTAAAGATCCCAAAATTTAGACTGAAAGACAGG<br>AAACACCTAAGAAAAAATCTAAACTTCTTAGGGGTGAAAAGTGCATT<br>CACAAATAAGGCAAATTTTACTGGAATTTCTGGAGAAGGAAATTTGG<br>AAGTGTCAAAGATTATTTACAGAGCTGTGGTTGAAGTCAATGAAAAG<br>GGTAGCGAAGCAGCAGGGGCCACTGCCGAATTTGCCAGGTTTGGGA<br>AATCCAGAGGTAGACCAAAGCCGGGAAATTTTTATGTAAACCATCCT<br>TTTGTCTTCTTCATTAGAGAAGACAGGACTGGAATGATTCTTTTCTAT<br>GGAAGGGTCAATAAATTGTAA
TatPInSrp07	XP_011215073.1	2 e-85	PF00079	--	Serpin-like	MDSAVASSNELSLKLLRICRNSTPEANLFYSPASITICLS<br>MLKTGAKEETLKQIEEILSIGEDQCKDDGFGVLSRYLQE<br>SKGPITLKISNILAVDQQFAIEPEFVNNLKLNYNSEMKEL<br>PFQSDPEKSRKEINRLVEEGTNGKIKDMLVPGSISSDSRL<br>VLANAIYFMGKWQTRFKRYETRKVPFYTINEENSMVD<br>MMFKMESYKYIHSKDLNADIIEVPYLGETVSLIILLPFER<br>FGLPLLEKNISVQKLNHAVGQMQMENYVELGLPKFKLE<br>EKISLEEHLQNMGMTEAFIKGAANFSGISGEGNLCLSSAI<br>HKTFLDINEEGTEAAAATGLEIMLCCLPPPPRFHFTVDHP<br>FMLLIRESTTGLILFLGSVNNL	ATGGATTCTGCAGTTGCATCTTCTAATGAATTAAGCTTGAAACTTCTT<br>CGTATATGTAGAAATAGCACACCAGAAGCGAATCTATTTTATTCGCC<br>AGCCAGTATTACGATATGTTTGTCTATGCTGAAAACTGGTGCAAAAG<br>AAGAAACGCTAAAGCAAATCGAAGAGATTCTAAGTATTGGAGAAGA<br>TCAGTGCAAGGATGATGGATTCGGAGTTCTTTCGAGATATTTGCAAG<br>AATCAAAAGGTCCTATTACTTTGAAGATATCAAACATACTCGCTGTT<br>GATCAGCAGTTTGCGATAGAACCAGAATTCGTTAATAATTTAAAATT<br>AAATTATAACTCTGAGATGAAAGAGCTTCCATTTCAGTCCGATCCGG<br>AAAAGTCCAGGAAAGAAATAAATCGTTTGGTAGAAGAAGGCACGAA<br>TGGAAAAATTAAAGACATGCTTGTACCAGGATCGATCTCTTCAGATT<br>CCAGACTCGTTTTGGCCAATGCTATTTATTTTATGGGGAAATGGCAA<br>ACTAGATTTAAGCGATATGAAACAAGAAAAGTTCCCTTCTATACAAT<br>TAATGAAGAGAACTCTATGGTAGATATGATGTTTAAAATGGAGTCTT<br>ATAAATATATCCATAGTAAGGATTTAAATGCAGACATAATCGAAGTA<br>CCATATCTAGGAGAAACAGTTAGTTTGATCATATTACTTCCATTTGAA<br>AGATTTGGTCTCCCTCTTCTTGAAAAGAATATTTCTGTACAGAAGTTG<br>AACCACGCAGTTGGGCAAATGCAGATGGAAAACTATGTAGAACTTG<br>GACTTCCAAAGTTTAAACTGGAGGAGAAAATCTCTCTCGAAGAACAT<br>CTACAGAATATGGGAATGACAGAAGCATTCATTAAAGGAGCAGCTA<br>ACTTTTCAGGGATTTCTGGTGAAGGAAACCTATGTCTCTCATCCGCTA<br>TCCATAAAACATTCCTTGATATCAACGAAGAGGGCACAGAAGCAGCT<br>GCAGCCACTGGATTAGAAATTATGCTGTGTTGTTTACCTCCACCTCCT<br>CGTTTTCACTTTACCGTGGATCATCCTTTCATGCTGTTGATCCGCGAA<br>TCAACTACTGGACTAATCCTATTTCTTGGAAGTGTTAACAACTTATAG

TatPInSrp08	XP_015915292.1	7 e-120	PF00079	--	Serpin like	MQKIVLILAGIIAVSSSRCIQTNNTIDTTDIERSLPSLQYGS<br>TNFALEMFRVLHSESSIPDNLFFSPVSIWTALVTIFEGAR<br>QQTEKELASVLGLTNISPALLPAALDAFLQSCSSCQEGD<br>KPNVKIANRIYFQDGLEFKLCNQQLEKIIHKIDFESDPEK<br>ARDTINNFVESVTNGKIQELIPPNSVGPLTQMIIANAVYF<br>KGTWKNQFPPSMTRQVRFNVDRDSTVTVSMMNTIGTY<br>FYGVSDQMDCQALEIPYNDDRVSMLILLPKHSFRGLDTL<br>ARTISPERLKALLNSMVSREVLVSIPKFKVEQEYEMSKP<br>LQKMGLRNLFDPRFSDLSGFTGARRLNLDAIHHKSYIKV<br>NEEGTEAAAATAFVISRTGTQDSGITRFYADRPFLYFIRD<br>SYSGSMLFMGTVRSPRYDEN	ATGCAGAAGATAGTTTTAATATTGGCTGGGATCATCGCAGTTTCCTC<br>CTCGAGATGTATACAAACGAACAACACGATTGATACAACAGATATC<br>GAAAGAAGTTTACCATCCTTGCAATATGGAAGCACAAATTTTGCATT<br>GGAGATGTTTCGAGTTCTCCACAGTGAATCGTCCATACCTGATAACT<br>TGTTTTTCAGTCCCGTGAGTATATGGACAGCTCTTGTTACAATTTTTG<br>AAGGAGCCAGACAACAGACAGAAAAAGAACTCGCATCTGTGCTTGG<br>ACTAACCAATATTAGCCCCGCGCTACTTCCAGCGGCACTTGATGCAT<br>TTCTTCAGAGCTGCAGCAGTTGCCAAGAAGGAGATAAACCAAATGTA<br>AAGATAGCCAACAGAATTTATTTTCAAGATGGTCTTGAGTTCAAATT<br>GTGTAACCAACAGTTGGAGAAAATTATTCATAAAATAGACTTCGAAT<br>CTGATCCAGAAAAGGCAAGAGATACTATAAACAACTTTGTGGAGAG<br>TGTAACGAATGGGAAGATACAGGAATTGATACCCCCGAATTCCGTGG<br>GTCCACTTACTCAAATGATAATAGCGAATGCTGTATATTTTAAAGGA<br>ACGTGGAAAAATCAGTTCCCTCCTTCTATGACGCGTCAGGTTCGTTTC<br>AATGTAGATCGCGATTCTACTGTTACAGTGTCGATGATGAACACGAT<br>AGGAACGTACTTCTACGGCGTTAGCGATCAAATGGACTGTCAAGCAT<br>TGGAGATTCCTTACAACGATGACAGAGTAAGCATGCTTATCTTACTA<br>CCTAAACACTCCTTTCGAGGATTGGATACACTTGCTAGGACTATTAG<br>TCCGGAACGACTGAAAGCTCTTCTTAATTCTATGGTATCTCGAGAAG<br>TTCTGGTTTCTATACCGAAATTCAAAGTTGAACAAGAGTACGAAATG<br>TCTAAACCTCTTCAGAAGATGGGATTGCGTAATCTTTTCGATCCTCGT<br>TTCTCTGATTTATCCGGATTTACGGGAGCCAGGAGACTAAATCTAGA<br>TGCCATTCATCATAAATCTTACATTAAAGTTAACGAGGAGGGAACAG<br>AAGCAGCCGCAGCAACAGCGTTTGTAATATCGAGGACAGGGACACA<br>AGATTCGGGGATCACACGTTTCTACGCAGACAGACCATTCTTGTACT<br>TTATACGCGACTCTTATTCTGGAAGTATGCTTTTCATGGGAACTGTAA<br>GATCACCAAGATACGATGAAAACTGA
TatPInSrp09	XP_002407493.1	1 e-90	PF00079	--	Serpin-like	MEIAVSSSNQFNLKLLKIYQDEKKEGNTFYSPASITIALA<br>MLYTGAQEKTRKQMEHILSIEGQHSIHEGFSSVFSLFQD<br>LPSETVLNITNMLAIDQKYNILPEFSNDLKSYYNTQTRLY<br>DFEFQSSKALAEINQLIEVATNGKIKDFFPNESIASDTILV<br>LANAIYFKGLWSISFDEKNTSKKPFYTITNEERMVDMMF<br>RRASFRISDSPNLNARLLEMPYIGNRLRMILILPTEKDGIS<br>FVENNITVDNLNNAIKNLQKANDVRLSLPRFKLEESLPL<br>KDCLIKMGMKDAFTDNADFSGITGNKDTLISAAFHKAFI<br>DVNEEGTEASAVTGMVKIAKCSRPPPPPVLFIVDHPFMFI<br>IQEIGTGLILFTGSIRSL	ATGGAAATTGCTGTTTCTTCCTCCAATCAGTTCAATCTTAAACTTTTG<br>AAAATATATCAAGATGAGAAGAAGGAAGGAAATACATTTTATTCTCC<br>TGCCAGCATTACTATAGCACTTGCAATGCTATATACCGGTGCACAAG<br>AAAAGACAAGAAAACAAATGGAACACATCTTGAGTATTGAAGGCCA<br>GCATTCTATACACGAAGGATTCTCCTCTGTTTTCAGCTTGTTTCAAGA<br>CCTCCCAAGTGAAACTGTTTTGAACATTACTAATATGTTAGCTATTGA<br>CCAAAAATATAATATATTGCCTGAATTTTCTAACGACTTGAAATCGT<br>ATTATAACACACAAACAAGATTGTATGATTTTGAATTTCAGTCATCA<br>AAAGCACTAGCAGAAATCAATCAGTTAATTGAAGTTGCTACTAATGG<br>AAAAATAAAAGACTTCTTTCCTAATGAAAGTATTGCTTCAGATACCA<br>TCTTAGTGTTAGCTAATGCTATTTATTTTAAGGGTCTCTGGAGTATAT<br>CTTTTGATGAAAAAAACACATCTAAAAAGCCTTTCTATACAATAACA<br>AATGAAGAGAGAATGGTTGACATGATGTTCAGAAGGGCTAGTTTTCG<br>TATTTCTGATAGTCCCAATTTAAATGCAAGATTGCTTGAAATGCCTTA<br>CATAGGAAACAGATTACGTATGATCTTGATACTTCCAACTGAAAAAG<br>ATGGTATCTCCTTTGTTGAAAACAATATAACTGTTGATAACTTGAAC<br>AATGCTATTAAAAATTTACAAAAGGCAAATGACGTACGTTTATCACT<br>TCCCAGATTCAAATTGGAAGAAAGTCTTCCATTAAAAGATTGCTTGA<br>TAAAAATGGGAATGAAAGATGCATTCACAGATAATGCTGATTTCTCA<br>GGAATCACTGGCAACAAAGATACTTTAATTTCTGCTGCTTTCCATAA<br>AGCATTTATTGATGTAAATGAGGAAGGCACAGAAGCATCAGCTGTA<br>ACTGGTATGGTAAAGATTGCTAAATGTTCTAGACCTCCCCCACCACC<br>TGTATTATTTATTGTGGATCACCCCTTCATGTTTATTATACAAGAGAT<br>TGGCACAGGGCTTATTCTTTTCACTGGAAGTATTCGATCATTGTAA

TatPInSrp10	JAT91131.1	2 e-105	PF00079	--	Serpin-like	MIKMNFFVLPLLLSFIDSEQLVISGETSVKELAVANNKFA<br>FKLYKTFNSDGNAFWSPISVLTAIGTLYTGARLKTAEEIR<br>HAVLNTVAGLSDEQINKAFHALIRSLSGGSDEGRLDIAN<br>AVIIRNSNPILREYKQNLRYFYSSIVKEMNPEIDSEELIRDI<br>NSWIRKRTYGKITELLESIDYDTVLVLLNAVYFKGKWK<br>KPFLDILTEKEPFYNKGIHATTVSMMIQENHFFYGNFPE<br>YDIHALKMPYLGDKFSMIILLPFQRNGLWRLEENLTPNM<br>FDHIVANLKNQNVIVKLPKFKLEDSRDIKRSLKMLGMK<br>SAFTDKADFGGINKDANLKISKIIHKAVIEVNEAGTEVA<br>GATGVVAIPRMFQEARYFLVEHPFLFFIRNEVTGAIVFFG<br>RLNEL	ATGATAAAAATGAACTTCTTCGTATTACCATTGCTGCTGTCGTTCATT<br>GATAGTGAACAGCTAGTTATATCTGGAGAAACTAGCGTAAAAGAAC<br>TAGCAGTAGCTAATAACAAGTTTGCCTTCAAACTGTACAAAACTTTT<br>AACTCCGACGGAAATGCTTTTTGGTCTCCAATTAGTGTTCTTACTGCC<br>ATTGGAACGCTATATACAGGAGCCAGATTGAAAACAGCGGAAGAAA<br>TTCGTCATGCCGTTTTAAATACCGTTGCAGGATTAAGTGATGAACAG<br>ATAAACAAAGCCTTTCACGCTTTGATTCGTTCCCTTTCAGGAGGATCT<br>GATGAGGGGAGATTAGACATTGCTAATGCTGTAATCATTCGTAATAG<br>TAACCCAATATTGAGAGAATACAAACAGAATTTGAGATACTTTTATA<br>GCTCCATAGTGAAAGAAATGAATCCCGAGATCGACTCTGAGGAGCT<br>GATTCGTGATATCAACAGCTGGATTAGAAAGAGAACTTATGGAAAA<br>ATAACAGAGTTGCTAGAATCTATAGATTATGATACAGTGCTAGTGCT<br>TCTGAACGCAGTTTATTTCAAGGGTAAGTGGAAAAAGCCATTTCTAG<br>ATATCCTCACTGAAAAAGAACCGTTTTATAATAAAGGAATTCACGCT<br>ACTACTGTATCCATGATGATTCAAGAGAATCATTTCTTCTATGGAAAT<br>TTTCCTGAATATGACATTCACGCTTTAAAGATGCCTTACTTAGGTGAT<br>AAATTCAGCATGATTATACTTCTGCCCTTTCAAAGAAATGGTCTTTGG<br>CGATTAGAAGAAAACTTAACTCCTAATATGTTCGATCATATTGTTGC<br>GAATTTAAAAAATCAAAATGTCATAGTTAAATTGCCTAAGTTCAAAC<br>TGGAAGATTCTAGAGACATAAAGCGTAGTTTGAAAATGTTAGGAATG<br>AAAAGCGCTTTTACAGATAAAGCTGATTTTGGTGGTATTAATAAAGA<br>CGCCAATCTGAAAATATCCAAGATCATTCACAAAGCAGTGATAGAA<br>GTAAATGAAGCGGGAACCGAAGTGGCTGGTGCGACTGGTGTAGTTG<br>CTATTCCAAGGATGTTTCAAGAGGCAAGATATTTCCTCGTAGAGCAT<br>CCCTTTTTGTTTTTCATTAGAAATGAAGTAACGGGAGCGATCGTGTTT<br>TTTGGACGCCTCAATGAATTATAA
TatPInSrp11	XP_014715077.1	1 e-83	PF00079	--	Serpin-like	MGIPAMDSAVASSNELSLKLLRICRNSTPEANLFYSPASI<br>TICLSMLKTGAKEETLKQIEEILSIGEDQCKDDGFGVLSR<br>YLQESKGPITLKISNILAVDQQFAIEPEFVNNLKLNYNSE<br>MKELPFQSDPEKSRKEINRLVEEGTNGKIKDMLVPGSISS<br>DSRLVLANAIYFMGKWQTRFKRYETRKVPFYTINEENS<br>MVDMMFKMESYKYIHSKDLNADIIEVPYLGETVSLIILL<br>PFERFGLPLLEKNISVQKLNHAVGQMQMENYVELGLPK<br>FKLEEKISLEEHLQNMGMTEAFIKGAANFSGISGEGNLC<br>LSSAIHKTFLDINEEGTEAAAATALEITLYCLPPPPRFHFT<br>VDHPFMMLIRESTTGLILFLGSVNNL	ATGGGTATACCAGCGATGGATTCTGCAGTTGCATCTTCTAATGAATT<br>AAGCTTGAAACTTCTTCGTATATGTAGAAATAGCACACCAGAAGCGA<br>ATCTATTTTATTCGCCAGCCAGTATTACGATATGTTTGTCTATGCTGA<br>AAACTGGTGCAAAAGAAGAAACGCTAAAGCAAATCGAAGAGATTCT<br>AAGTATTGGAGAAGATCAGTGCAAGGATGATGGATTCGGAGTTCTTT<br>CGAGATATTTGCAAGAATCAAAAGGTCCTATTACTTTGAAGATATCA<br>AACATACTCGCTGTTGATCAGCAGTTTGCGATAGAACCAGAATTCGT<br>TAATAATTTAAAATTAAATTATAACTCTGAGATGAAAGAGCTTCCAT<br>TTCAGTCCGATCCGGAAAAGTCCAGGAAAGAAATAAATCGTTTGGTA<br>GAAGAAGGCACGAATGGAAAAATTAAAGACATGCTTGTACCAGGAT<br>CGATCTCTTCAGATTCCAGACTCGTTTTGGCCAATGCTATTTATTTTA<br>TGGGGAAATGGCAAACTAGATTTAAGCGATATGAAACAAGAAAAGT<br>TCCCTTCTATACAATTAATGAAGAGAACTCTATGGTAGATATGATGT<br>TTAAAATGGAGTCTTATAAATATATCCATAGTAAGGATTTAAATGCA<br>GACATAATCGAAGTACCATATCTAGGAGAAACAGTTAGTTTGATCAT<br>ATTACTTCCATTTGAAAGATTTGGTCTCCCTCTTCTTGAAAAGAATAT<br>TTCTGTACAGAAGTTGAACCACGCAGTTGGGCAAATGCAGATGGAA<br>AACTATGTAGAACTTGGACTTCCAAAGTTTAAACTGGAGGAGAAAAT<br>CTCTCTCGAAGAACATCTACAGAATATGGGAATGACAGAAGCATTCA<br>TTAAAGGAGCAGCTAACTTTTCAGGGATTTCTGGTGAAGGAAACCTA<br>TGTCTCTCATCCGCTATCCATAAAACATTCCTTGATATCAACGAAGA<br>GGGCACAGAAGCAGCTGCAGCCACTGCATTAGAAATTACGCTGTATT<br>GTTTACCTCCACCTCCTCGTTTTCACTTTACCGTGGATCACCCTTTCAT<br>GATGTTGATCCGCGAATCAACTACTGGACTAATCCTATTTCTTGGAA<br>GTGTTAACAACTTATAG

TatPInSrp12	JAT91131.1	4 e-140	PF00079	--	Serpin-like	MKEKMIALMVLFLGGLLTSQASSELKNLGKANNDFSIKI<br>YKSLPSTGNAFFCPASVFTALGMLFKGAGGNTAEEMRE<br>ALSYKTAGLTDETVHQKFKDVMELFGNPSNEYALEIAN<br>AIVTSDGFEISEEYKEALKTFYKAITKKVSFKGAEKAAA<br>LNEINGWVSEKTHGKIAKLLEDLDEDTVMVLLNAVYFK<br>GTWESEFNANLTKDDVFYANGKDPKTVRMMNKRNFFL<br>YNEFDKDNFKALKLPYKGEHLSMVILLPNERDGLKQLE<br>ESLTLQKLTEVMSSLGDRERKVKVSIPKFKLEDSRKLVE<br>NLKQLGMKDAFKGGAADFTGIDKSGRLVVSDVVHKAV<br>IDVNEEGTEAAGATAVIGIVRSSRPLRQLEFKADHPFLFF<br>IHDQRTEMILFMGRVNEL	ATGAAAGAAAAGATGATTGCTTTAATGGTGTTATTTCTCGGTGGTCT<br>CCTTACAAGTCAAGCATCCTCTGAGTTAAAGAACTTAGGAAAAGCGA<br>ATAATGACTTCTCTATAAAGATATACAAATCATTACCATCAACAGGA<br>AATGCCTTCTTTTGTCCAGCTAGCGTCTTTACCGCTCTGGGAATGCTG<br>TTTAAAGGAGCAGGAGGAAATACCGCTGAAGAAATGCGAGAAGCGT<br>TATCCTACAAGACAGCTGGATTAACTGACGAAACGGTACATCAGAA<br>ATTCAAAGATGTCATGGAATTATTTGGAAACCCTTCTAACGAGTACG<br>CACTAGAAATTGCAAATGCTATAGTGACGTCTGATGGATTCGAGATA<br>TCCGAGGAGTATAAGGAAGCATTGAAAACGTTCTACAAGGCAATAA<br>CAAAAAAGGTTAGTTTTAAAGGGGCTGAGAAGGCGGCTGCCCTTAAT<br>GAAATCAATGGCTGGGTTAGTGAGAAAACTCATGGAAAAATTGCCA<br>AACTTTTAGAAGATTTAGATGAGGATACCGTGATGGTACTGCTGAAT<br>GCTGTTTACTTTAAAGGAACATGGGAATCTGAATTCAATGCAAATCT<br>TACAAAAGATGATGTATTTTATGCTAATGGAAAAGATCCCAAAACGG<br>TACGAATGATGAACAAACGAAATTTTTTCTTGTACAATGAATTTGAT<br>AAAGACAATTTCAAAGCACTTAAACTGCCTTATAAAGGGGAACATCT<br>GAGTATGGTAATACTTCTGCCAAACGAAAGAGATGGATTGAAGCAA<br>TTAGAAGAATCTCTGACATTACAGAAGCTTACCGAGGTCATGTCTAG<br>TTTAGGCGACCGTGAACGAAAAGTAAAAGTGTCCATTCCCAAATTTA<br>AACTTGAGGACAGCAGGAAGTTAGTGGAAAACTTGAAGCAGCTGGG<br>TATGAAAGATGCGTTTAAAGGTGGAGCAGCAGATTTTACTGGCATTG<br>ACAAAAGTGGACGATTAGTTGTTTCGGATGTTGTACACAAAGCTGTA<br>ATAGATGTTAACGAAGAAGGAACTGAAGCTGCAGGTGCTACAGCTG<br>TTATTGGCATTGTGCGATCCAGCAGACCTCTTCGTCAACTTGAATTTA<br>AGGCTGATCATCCGTTCTTATTTTTTATTCATGACCAGAGAACAGAA<br>ATGATTCTTTTTATGGGACGAGTTAATGAACTATAA
TatPInSrp13	JAT91131.1	8 e-121	PF00079	--	Serpin-like	MLSKGAGGNTAEEMREVLSYKTAELTDETIHQQFKDLL<br>QLIEKSSNEYVLEIANAIITSDEYAISEEYKGALKTFYSA<br>MSKEVNFKGSEKAAALAEINGWVSEKTHGKIAKLLEDL<br>DEDTVMVLLNAIYFKGMWKSEFNANRTKDDVFYANGK<br>DPKTVRMMNKRNFFLYNEFDKDNFKALKLPYKGEHLS<br>MVILLPNERDGLKQLEESLTLQKLTEVMSSLGDRERKV<br>KVSIPKFKLEDSRKLVENLKQLGMKDAFKGGAADFTGI<br>DKSGRLVVSDVVHKAVIDVNEEGTEAAGATAVIGIVRS<br>SRPLRQLEFKADHPFLFFIHDQRTEMILFMGRVNEL	ATGCTGTCTAAAGGTGCAGGAGGAAATACTGCTGAAGAAATGCGAG<br>AAGTTTTATCCTACAAGACAGCTGAATTAACTGACGAAACAATACAT<br>CAACAATTCAAAGATCTCCTCCAACTGATCGAAAAATCTTCTAACGA<br>ATATGTCCTGGAAATTGCAAATGCCATAATAACATCTGATGAATATG<br>CAATATCCGAAGAGTACAAGGGAGCACTGAAAACTTTTTACAGCGC<br>AATGAGTAAAGAAGTTAATTTTAAAGGAAGTGAAAAAGCTGCTGCC<br>CTTGCAGAAATCAATGGCTGGGTTAGTGAGAAAACTCATGGAAAAA<br>TTGCCAAACTTTTAGAAGATTTAGATGAGGATACCGTGATGGTATTG<br>CTGAATGCTATTTACTTTAAAGGAATGTGGAAATCGGAATTCAATGC<br>AAATCGTACAAAAGATGATGTATTTTATGCTAATGGAAAAGATCCCA<br>AAACGGTACGAATGATGAACAAACGAAATTTTTTCTTGTACAATGAA<br>TTTGATAAAGACAATTTCAAAGCACTTAAACTGCCTTATAAAGGGGA<br>ACATCTGAGTATGGTAATACTTCTGCCAAACGAAAGAGATGGATTGA<br>AGCAATTAGAAGAATCTCTGACATTACAGAAGCTTACCGAGGTCATG<br>TCTAGTTTAGGCGACCGTGAACGAAAAGTAAAAGTGTCCATTCCCAA<br>ATTTAAACTTGAGGACAGCAGGAAGTTAGTGGAAAACTTGAAGCAG<br>CTGGGTATGAAAGATGCGTTTAAAGGTGGAGCAGCAGATTTTACTGG<br>CATTGACAAAAGTGGACGATTAGTTGTTTCGGATGTTGTACACAAAG<br>CTGTAATAGATGTTAACGAAGAAGGAACTGAAGCTGCAGGTGCTAC<br>AGCTGTTATTGGCATTGTGCGATCCAGCAGACCTCTTCGTCAACTTGA<br>ATTTAAGGCTGATCATCCGTTCTTATTTTTTATTCATGACCAGAGAAC<br>AGAAATGATTCTTTTTATGGGACGAGTTAATGAACTATAA

TatPInSrp14	XP_005307140.1	1 e-101	PF00079	--	Serpin-like	MFSCYRLRSSRTNCSCWFNLLLGVVTLNVLATSMATSP<br>KNLLSKAFSEFAVKLYRSVSEQQDNVLVSPISISTVLSMV<br>LVGAKGSTSDQMKKVLSIDSIEETIPGFSDLISHLNSGNIS<br>DKMSIGNRVFLEEGLPIVNDFTFLMKDVFKAEYGKVSFS<br>TQPLAAKEAINSWVSAITNEKIKELITDDIDPLTKLVLVS<br>AIYFKGFWAEQFLKDSTEIAPFHLNSKQSINIEMMYQKK<br>KFGFAYDSELKVKIIDLPYEQLAVSMIIILPDEYEGLSTIE<br>RQLDSDKLNKLLLEVRKHQTKLDVFLPKFKLEESLSLKP<br>ILSAMGMVDMFCSDKADLSGIVEGKDLYVESVIHKACI<br>DVNEEGTEAAAATAVRIMARSIHIIPEFRADHPFLFLIVE<br>NSHNLILFFGALKTPKAVESRDHEEL	ATGTTTTCATGTTATCGATTACGGTCTTCGAGGACAAATTGTAGTTGC<br>TGGTTTAATTTGTTGTTAGGCGTAGTTACTCTCAACGTTTTAGCTACT<br>AGTATGGCTACATCACCCAAAAATCTACTATCCAAGGCATTTTCAGA<br>GTTTGCAGTGAAACTGTACAGATCAGTTTCTGAACAACAAGACAATG<br>TTTTAGTATCCCCTATAAGTATTTCTACTGTGTTGAGCATGGTTTTGG<br>TAGGAGCAAAAGGAAGTACTTCAGACCAGATGAAGAAAGTGCTCAG<br>CATTGATAGCATTGAAGAAACAATACCTGGTTTTTCTGATTTAATCTC<br>TCACTTAAATTCTGGCAACATTTCTGACAAAATGAGCATTGGAAATA<br>GGGTGTTTTTGGAGGAAGGACTGCCAATTGTAAATGATTTTACATTT<br>CTGATGAAAGATGTATTTAAAGCTGAATATGGGAAAGTCAGCTTTTC<br>CACTCAGCCATTGGCTGCTAAAGAAGCTATCAATTCTTGGGTTTCAG<br>CAATCACAAATGAAAAGATAAAAGAATTAATCACTGATGACATCGA<br>TCCATTGACAAAACTTGTGCTTGTCAGTGCAATTTACTTTAAAGGCTT<br>TTGGGCAGAACAGTTTTTGAAGGATTCAACCGAGATTGCTCCATTCC<br>ATTTAAACTCCAAACAATCTATTAATATTGAAATGATGTATCAGAAG<br>AAAAAATTTGGATTTGCCTATGATAGTGAATTGAAAGTTAAGATTAT<br>TGATTTGCCTTATGAACAACTTGCCGTTAGCATGATTATTATATTGCC<br>CGATGAGTATGAAGGACTGAGTACTATTGAGCGGCAGCTTGATTCAG<br>ATAAGCTAAATAAGCTTCTTCTTGAAGTTAGAAAGCATCAGACTAAG<br>TTAGATGTTTTCTTGCCAAAATTTAAATTGGAGGAATCATTGAGCCTA<br>AAGCCCATTCTTTCTGCAATGGGTATGGTGGATATGTTCTGTTCAGAC<br>AAAGCAGACTTATCTGGTATTGTAGAAGGTAAAGATTTGTATGTTGA<br>ATCAGTAATTCACAAAGCATGTATCGATGTAAATGAGGAAGGCACTG<br>AGGCTGCTGCAGCTACTGCCGTTCGTATTATGGCAAGAAGCATTCAT<br>ATTATACCAGAGTTTCGTGCTGATCATCCATTCCTATTTCTTATTGTT<br>GAAAATTCACATAATTTGATTCTTTTCTTTGGTGCTTTGAAAACCCCA<br>AAAGCTGTTGAGAGCAGAGATCATGAAGAATTATAA
TatPInSrp15	XP_013779833.1	6 e-116	PF00079	--	Serpin-like	MQKILLVLICSFAAAVSSCIPENGTTNVTNIQNNLLSFQY<br>GSIDFALDMFRGLYNDTVNLFFSPLSVWVTLVTIYTGSR<br>HQTAKELSSVLGITDTDLTLLPETLSAFIEICSGCKQDNK<br>STVKIANRIYFQEDLELKLCGREVEKMFHKIDFKTNPEG<br>ARDTINDFIKNETNGKILELIPPLSVSPLTQMVIANAVYF<br>KGIWRNKFNKTMTRPLRFYPDRETTLTVDMMNTIGTYI<br>YGVSDQMDCQALEIPYEGGSINMLILLPRHSFRGLDILAS<br>TITPERLENLLNSMASQDVLITLPKFKVEQQYELSKTLRK<br>LGLRNLFDPRFSDLSGFTGIRGLSIDAIHHKSYIKVNEEG<br>TEAAGATGSLLSRDSHPLGVTRFYANRPFVYFIRDTYSD<br>ELLFMGTIRVPQYNKQQ	ATGCAGAAGATATTATTGGTATTAATCTGTAGTTTTGCTGCTGCCGTT<br>TCAAGCTGCATACCAGAAAATGGTACAACAAATGTCACCAATATTCA<br>AAACAATTTACTTTCCTTCCAGTATGGCAGCATAGATTTCGCCCTGGA<br>TATGTTTCGTGGTCTTTATAATGATACCGTAAACTTATTTTTTAGCCC<br>GCTTAGTGTGTGGGTAACTCTCGTTACCATTTATACAGGCTCAAGAC<br>ATCAAACGGCCAAAGAACTTTCCAGTGTTTTAGGGATAACTGATACT<br>GATCTAACATTGCTGCCCGAAACACTAAGTGCCTTTATAGAAATCTG<br>CAGTGGCTGCAAACAGGATAATAAATCAACTGTTAAAATAGCAAAT<br>AGAATTTACTTTCAGGAAGATCTTGAATTAAAACTCTGCGGAAGGGA<br>AGTGGAGAAAATGTTTCATAAAATTGACTTCAAAACTAATCCCGAAG<br>GAGCGAGGGACACTATTAATGATTTCATAAAAAATGAAACAAATGG<br>AAAAATATTGGAATTAATCCCTCCTCTGTCTGTTAGCCCTCTAACTCA<br>AATGGTAATTGCTAATGCAGTTTATTTTAAAGGAATTTGGCGGAATA<br>AATTCAACAAAACAATGACTCGTCCACTTCGTTTCTATCCAGATCGT<br>GAAACCACTTTAACGGTGGACATGATGAATACAATAGGAACGTATAT<br>TTATGGAGTTAGCGACCAAATGGATTGCCAAGCTCTAGAGATTCCTT<br>ACGAAGGTGGAAGCATAAACATGCTGATATTATTACCGAGACATTCC<br>TTCCGAGGATTGGACATCCTCGCAAGTACGATTACTCCAGAACGTCT<br>AGAGAACCTCCTCAACTCTATGGCTTCCCAAGACGTTTTAATTACTCT<br>ACCCAAATTTAAAGTTGAACAACAGTATGAACTATCAAAGACTCTCC<br>GGAAACTTGGATTGCGTAATCTGTTTGATCCTCGTTTTTCCGATCTCT<br>CAGGCTTCACAGGAATTAGAGGCTTAAGCATAGATGCCATACATCAT<br>AAATCTTACATTAAAGTCAATGAAGAAGGAACAGAAGCTGCTGGAG<br>CAACGGGTTCCCTGTTGTCCAGGGATTCTCATCCACTGGGAGTTACA<br>CGATTTTATGCAAATAGACCTTTCGTGTACTTTATTCGTGACACTTAT<br>TCTGACGAACTACTCTTTATGGGCACAATAAGGGTTCCTCAATATAA<br>TAAACAGCAATAA

TatPInSrp16	JAT91131.1	5 e-133	PF00079	--	Serpin-like	MIPLTSAFLLALVVTQQASSEYDDIKNLALANNELAFKL<br>HKTFNLQGNAFTSPASIFTALAMLYQGARGVTAEEMRE<br>VLSFSVAGLTDRNINQHFQELLSLLGRSSGEYKLNIANAI<br>LSQSRSPVLKNYKDVLEKYYHAKLKEVDFARENAKAV<br>EEVNNWVSDKTNNKITNLLDAIPSDTVLLLLNAVYFKG<br>TWKTQFSPRTTREAPFYNDGINEVSVQMMGAKNKFFYG<br>DFNDYGVQAVKLPYNGEDMSMVVLLPYKRDGLQKVE<br>QQLNPNKLDDIISKMELETVIVNIPKFKLEDKKELKLNLT<br>NLGMRSAFVNGANFAGITNEGNLKVSEIVHKAVVEVNE<br>EGSEAAGASAVLIVPYSSGNVVQYFGANHPFIFFIRDDRT<br>GMILFYGRVNNL	ATGATTCCTTTAACATCAGCATTCCTCTTAGCTCTGGTTGTTACCCAA<br>CAAGCCTCCTCAGAATACGATGACATTAAGAATCTAGCATTGGCCAA<br>TAACGAACTAGCTTTCAAACTGCACAAAACATTTAATTTGCAAGGAA<br>ATGCCTTTACCTCACCGGCAAGTATCTTCACTGCTCTGGCTATGCTCT<br>ATCAAGGTGCAAGAGGTGTTACAGCTGAGGAGATGCGTGAAGTATT<br>GTCTTTCAGTGTTGCCGGTTTGACTGACAGAAACATCAACCAACATT<br>TCCAAGAATTACTTTCCCTTCTGGGGAGATCTTCTGGAGAATACAAA<br>CTTAATATTGCCAATGCTATTTTGTCGCAGTCTAGGAGTCCAGTACTG<br>AAGAATTACAAAGATGTCCTGGAGAAGTACTACCATGCCAAGTTGA<br>AAGAAGTTGATTTTGCAAGAGAGAACGCAAAGGCTGTTGAAGAAGT<br>TAATAACTGGGTTAGTGATAAAACAAACAACAAAATCACCAATTTGT<br>TGGATGCAATACCTTCCGATACAGTTTTGTTACTGCTGAATGCTGTTT<br>ATTTCAAAGGTACATGGAAAACTCAGTTCTCTCCACGTACCACTAGA<br>GAGGCACCATTCTACAATGATGGCATCAATGAAGTTTCAGTTCAAAT<br>GATGGGAGCAAAGAATAAGTTCTTTTATGGTGATTTCAATGACTATG<br>GTGTCCAGGCTGTGAAACTGCCATACAATGGTGAAGATATGAGCATG<br>GTAGTACTTCTACCATATAAAAGAGATGGTTTACAAAAAGTAGAACA<br>ACAGCTCAATCCAAATAAATTAGACGACATTATTTCCAAAATGGAGT<br>TGGAAACCGTTATCGTCAACATACCTAAGTTTAAATTGGAAGATAAG<br>AAGGAATTAAAACTTAATCTAACCAATTTAGGAATGAGAAGTGCTTT<br>TGTTAATGGAGCAAATTTCGCCGGTATTACAAATGAAGGAAACCTTA<br>AAGTATCAGAAATTGTTCACAAAGCTGTGGTTGAAGTAAATGAGGA<br>AGGAAGTGAAGCAGCTGGTGCAAGTGCTGTTTTAATAGTACCATATT<br>CCTCAGGGAATGTTGTACAATACTTTGGAGCCAACCATCCTTTTATCT<br>TCTTCATCAGAGACGATAGGACTGGAATGATACTTTTCTATGGAAGA<br>GTCAATAATTTGTAA
TatPInSrp17	JAT91131.1	7 e-139	PF00079	--	Serpin-like	MIGKMIVLMVMFLAGLLTSQASTDLKNLAKANNDFCIK<br>IYKSLPSTGNAFFCPASVFTALGMLFKGAGGNTAEEMRE<br>ALSYKTAGLTDETVHQKFKDVMELFGNPSNEYALEIAN<br>AIVTSDGFEISEEYKEALKTFYKAITKKVSFKGAEKAAA<br>LNEINGWVSEKTHGKIAKLLEDLDEDTVMVLLNAVYFK<br>GTWESEFNANLTKDDVFYANGKDPKTVRMMNKRNFFL<br>YNEFDKDNFKALKLPYKGEHLSMVILLPNERDGLKQLE<br>ESLTLQKLTEVMSSLGDRERKVKVSIPKFKLEDSRKLVE<br>NLKQLGMKDAFKGGAADFTGIDKSGRLFVSDIVHKAIIE<br>INEEGAEAAGVTGVVIVPLSARPPQQLKFNADHPFLFFIQ<br>DDRTNMILFMGRVNDI	ATGATAGGCAAGATGATTGTTTTAATGGTGATGTTTCTTGCTGGTCTT<br>CTAACAAGTCAAGCTTCTACAGATTTAAAGAATTTAGCAAAAGCGAA<br>TAACGACTTCTGTATAAAGATATACAAATCATTACCATCAACAGGAA<br>ATGCCTTCTTTTGTCCAGCTAGCGTCTTTACCGCTCTGGGAATGCTGT<br>TTAAAGGAGCAGGAGGAAATACCGCTGAAGAAATGCGAGAAGCGTT<br>ATCCTACAAGACAGCTGGATTAACTGACGAAACGGTACATCAGAAA<br>TTCAAAGATGTCATGGAATTATTTGGAAACCCTTCTAACGAGTACGC<br>ACTAGAAATTGCAAATGCTATAGTGACGTCTGATGGATTCGAGATAT<br>CCGAGGAGTATAAGGAAGCATTGAAAACGTTCTACAAGGCAATAAC<br>AAAAAAGGTTAGTTTTAAAGGGGCTGAGAAGGCGGCTGCCCTTAAT<br>GAAATCAATGGCTGGGTTAGTGAGAAAACTCATGGAAAAATTGCCA<br>AACTTTTAGAAGATTTAGATGAGGATACCGTGATGGTACTGCTGAAT<br>GCTGTTTACTTTAAAGGAACATGGGAATCTGAATTCAATGCAAATCT<br>TACAAAAGATGATGTATTTTATGCTAATGGAAAAGATCCCAAAACGG<br>TACGAATGATGAACAAACGAAATTTTTTCTTGTACAATGAATTTGAT<br>AAAGACAATTTCAAAGCACTTAAACTGCCTTATAAAGGGGAACATCT<br>GAGTATGGTAATACTTCTGCCAAACGAAAGAGATGGATTGAAGCAA<br>TTAGAAGAATCTCTGACATTACAGAAGCTTACCGAGGTCATGTCTAG<br>TTTAGGCGACCGTGAACGAAAAGTAAAAGTGTCCATTCCCAAATTTA<br>AACTTGAGGACAGCAGGAAGTTAGTGGAAAACTTGAAGCAGCTGGG<br>TATGAAAGATGCGTTTAAAGGTGGAGCAGCAGATTTTACTGGCATTG<br>ACAAAAGTGGACGCCTATTTGTTTCGGACATTGTACACAAAGCTATA<br>ATAGAAATTAATGAGGAGGGAGCAGAAGCAGCAGGTGTGACAGGTG<br>TTGTTATTGTTCCTTTATCCGCAAGACCTCCTCAACAGTTGAAGTTCA<br>ATGCTGACCATCCTTTCTTATTTTTCATTCAGGATGACAGAACAAATA<br>TGATTCTTTTTATGGGTAGAGTTAATGACATATAA

TatPInSrp18	XP_013786963.1	9 e-144	PF00079	--	Serpin like	MYPVVISVFFYISAFLYLQGNAETVNSPFNHLVEANLDF<br>SSDLYNEISKRKGYQDNIMFSPFGLYTVLLMTELGSRGQ<br>TAQELCQLLHLCRVNISYIHDAYGRIVTDLLTDASIRQSL<br>SIANRLYVQKGLEISKFYDYALRYYYNTTIRQVDFQNNS<br>AQVREEINNWVHGQTSGRILHFLSRPPSQATKMMTINAL<br>YFDSDWLWKFDPAMTEPKARFYVTPHFHAVVPMMVG<br>KLNLAIGHCSSLDASILELPFKSNRISMFLVLPDQLEGLGI<br>LQKQLNSTTMKQLISTMKKGEVNVRIPRFYVESQPLITE<br>LLRNLGVHHLFSPSANLKGITTSSDIYIGDMIHWAVINLD<br>ERGSMAVSATATVVERIGTFNGQYFEADHPFLFFIMDKQ<br>TGLVLFMGRIAGP	ATGTATCCTGTGGTCATTTCAGTGTTTTTCTACATATCAGCCTTTTTGT<br>ACCTCCAAGGCAATGCCGAGACAGTGAACTCACCTTTTAACCATTTA<br>GTAGAAGCTAATTTAGACTTCAGTTCTGATTTGTACAATGAAATATC<br>GAAGCGCAAAGGTTATCAAGACAACATCATGTTTTCTCCTTTTGGTTT<br>ATACACGGTACTACTAATGACAGAATTAGGCTCGAGAGGGCAGACC<br>GCACAAGAACTCTGTCAGTTACTCCACTTGTGTCGGGTTAATATTTCG<br>TACATTCACGACGCTTATGGTCGTATAGTGACAGATTTACTCACCGA<br>CGCATCAATTCGTCAATCATTATCAATAGCCAACCGTTTGTACGTTCA<br>GAAGGGGTTAGAAATATCGAAATTTTACGACTACGCACTACGGTATT<br>ACTACAACACAACAATACGACAAGTAGATTTTCAAAATAACAGTGC<br>GCAAGTACGAGAGGAGATAAATAACTGGGTTCACGGACAAACTTCT<br>GGAAGGATTCTACATTTTCTTTCTCGTCCTCCATCGCAGGCTACTAAA<br>ATGATGACTATTAATGCACTTTATTTCGATTCAGATTGGTTGTGGAAG<br>TTTGATCCAGCTATGACTGAACCGAAAGCTCGCTTTTACGTCACCCC<br>ACACTTCCATGCCGTAGTTCCAATGATGGTAGGTAAGCTGAACCTCG<br>CTATTGGACATTGCTCGTCGCTAGATGCAAGTATTTTAGAATTACCTT<br>TCAAATCGAACCGAATCAGTATGTTCCTAGTTTTACCCGATCAACTA<br>GAAGGACTCGGTATTCTACAGAAACAGCTAAACTCAACGACGATGA<br>AACAACTCATATCAACTATGAAGAAAGGTGAAGTGAATGTGCGAAT<br>TCCCAGATTTTATGTCGAATCTCAACCGCTAATCACCGAACTTCTACG<br>GAATCTCGGTGTACATCATCTATTCTCTCCATCTGCTAATCTAAAAGG<br>AATAACAACATCCAGTGACATTTATATCGGCGATATGATCCATTGGG<br>CGGTGATTAATTTAGACGAAAGAGGCAGCATGGCCGTATCTGCAACA<br>GCAACCGTAGTTGAACGGATAGGAACTTTCAATGGACAATATTTCGA<br>AGCTGATCATCCTTTCTTATTCTTTATAATGGACAAACAAACCGGATT<br>AGTGCTGTTTATGGGAAGGATAGCTGGTCCTTAA
TatPInSrp19	JAT91123.1	4 e-45	PF00079	--	Serpin-like	MVISLILTMLLILDVTRQGSSEFDNIKKIALANNEFTFKLS<br>GAFDSQENVIISPANIFIALAMLYQGARGITAEELRELLSF<br>NATGLSDEDMNQQFLELLTFLGNSSDDYNLYIANAVVS<br>QYQYPVLKEYKDVLKYYDATLKESNISEDHTKAVEEVN<br>DWVKNKTMGRIQYLSKSILNDTALVLLNTAYFKGTWK	ATGGTGATATCATTGATCCTAACAATGTTATTGATTCTGGATGTTACC<br>AGACAAGGATCCTCCGAATTTGATAACATTAAGAAAATTGCACTAGC<br>TAATAACGAATTTACTTTCAAATTAAGTGGGGCATTTGATTCTCAGG<br>AAAATGTCATAATTTCACCAGCGAATATTTTCATTGCTCTGGCAATGC<br>TGTATCAAGGTGCAAGAGGTATTACTGCCGAGGAACTGCGTGAATTG<br>TTGTCTTTCAATGCTACTGGATTGAGTGACGAAGACATGAATCAGCA<br>ATTTCTAGAATTGCTCACTTTCTTGGGAAACAGCTCTGATGATTACAA<br>TCTGTATATTGCTAACGCTGTTGTATCACAATATCAGTATCCGGTACT<br>AAAGGAATACAAAGATGTACTGAAGTACTACGATGCTACATTGAAA<br>GAATCTAATATTTCCGAAGACCATACAAAGGCAGTCGAAGAGGTAA<br>ACGATTGGGTCAAGAATAAAACAATGGGTAGGATCCAGTACTTGTCA<br>AAATCAATACTTAATGATACAGCTTTAGTATTGTTGAATACAGCTTAT<br>TTCAAAGGTACATGGAAA
TatPInKun01	XP_008472165.1	2 e-18	PF00014	--	Kunitz-type like	SFKISVLPKPYCRMLFSISVVSFVLFVLHPNGWTKEMDQ<br>CSLQPEGGNCRGIFQKFYYNETTGSCFPFFYGGCGGNEN<br>RFETEEDCMKTCKAEEIEIRIFRILPKQRKGIEKVTEKPQ	TCGTTTAAAATTTCCGTTCTCCCTAAGCCTTACTGCAGAATGCTATTC<br>TCAATTTCTGTTGTTTCCTTTGTTCTATTTGTTTTGCACCCTAACGGAT<br>GGACTAAAGAGATGGACCAATGTTCTCTTCAACCAGAGGGAGGCAA<br>TTGCAGAGGGATTTTCCAGAAATTTTATTACAACGAAACGACAGGTA<br>GCTGCTTCCCCTTTTTCTACGGTGGATGTGGTGGAAACGAAAATCGC<br>TTTGAAACAGAAGAAGATTGCATGAAAACCTGCAAAGCAGAAGAAA<br>TTGAAATTAGGATTTTCCGAATTCTTCCAAAGCAAAGAAAAGGAATT<br>GAAAAGGTTACAGAGAAACCTCAG
TatPInKun02	XP_015905520.1	1 e-109	PF00014	--	Kunitz-type like	VTQWSEWSPCTVTCGKGMKIRTRLYFDSSSLDTCNVELI<br>QKMLCMADRTDCSIDPAEAKEICMQPKETGACRGYFPR<br>WHYDLSRRECVQFIYGGCRGNRNNFERYSDCSQMCSMI<br>FKGPPSTIATLAASPIQVPNITEAPPVNCMVTPWSPWTAC<br>SRSCGNGRKERRRMIKVAPLNGGKPCPRRLTQRRKCKD<br>LPACSVDCMVTPWGEWSTCSTTCGQSSTQQRTREIKRP<br>AKHGGIPCGPRVERRFCTIPLCTY	GTTACACAGTGGTCTGAGTGGTCACCATGCACAGTTACTTGTGGTAA<br>GGGGATGAAAATCCGTACCCGCCTGTACTTTGATTCATCCTCTCTAG<br>ATACATGTAATGTGGAACTAATACAGAAAATGCTTTGTATGGCTGAT<br>AGAACCGATTGCAGTATCGACCCAGCCGAAGCCAAAGAAATCTGTA<br>TGCAGCCTAAGGAAACAGGAGCTTGCAGAGGATATTTTCCAAGGTG<br>GCACTACGATTTATCCAGAAGAGAATGCGTTCAATTTATATACGGAG<br>GATGCCGAGGGAACAGAAATAATTTTGAACGCTATTCAGACTGTAGT<br>CAGATGTGCTCGATGATATTTAAAGGTCCACCTAGCACTATAGCTAC<br>CTTAGCTGCATCCCCTATACAAGTCCCCAACATTACAGAAGCCCCAC<br>CTGTTAATTGTATGGTTACACCTTGGTCACCATGGACAGCATGTTCTC<br>GTTCCTGTGGTAATGGCCGTAAAGAAAGAAGAAGAATGATCAAAGT<br>TGCACCTCTTAATGGCGGGAAACCATGTCCCCGTAGGCTAACCCAGA<br>GAAGGAAGTGTAAAGATTTACCTGCCTGCTCTGTTGATTGTATGGTT<br>ACCCCATGGGGAGAATGGTCAACATGTTCAACAACGTGCGGTCAAA<br>GTAGTACTCAACAAAGAACAAGGGAAATTAAACGTCCAGCCAAGCA<br>TGGTGGTATACCTTGTGGACCAAGAGTGGAACGTAGGTTCTGTACAA<br>TTCCTCTTTGCACTTAT

TatPInKun03	ADV40356.1	9 e-16	PF00014	--	Kunitz-type like	CETGQDRKTVRDKRIFNNMASSLLLFLILTNLVIAVVSSKDVCELPPERGPCKARKTMFYYNSRCKCCKVFTYGGCRGNGNKFKTVDKCLDECAQD	TGCGAAACGGGACAAGACAGAAAGACAGTTCGCGATAAACGTATCTTCAACAACATGGCCTCATCGCTTCTGCTATTTTTAATCTTGACGAATCTAGTAATAGCGGTAGTTTCTTCTAAAGATGTCTGCGAACTTCCTCCGGAAAGAGGACCTTGCAAGGCACGTAAGACAATGTTTTATTACAACTCTAGATGCAAATGTTGCAAAGTCTTCACTTATGGAGGATGCAGAGGAAATGGCAATAAATTCAAAACGGTTGATAAGTGCTTAGATGAATGTGCACAAGATTAG
TatPInKun04	KFM77843.1	2 e-58	PF00014	--	Kunitz-type like	TSLLPIDRSFEDSLRFLVILRGMPSLNGGCGVTAWSDWSPCNATCEMTMGMRLRTRLFLNPAINISSCNIHTIETEPCRSERTDCNGAPKEICFEPKEEGPCRSYFVRWYYNVQEGRCLQFVYGGCRGNRNNFKSFSECHRICNNVLREPPYNMPIPLSPDDPTLVIDCRLTQWAPWSECSNTCGVAWKDRRRTVEINAQNGGKPCDSKLYQRKRCKSQPPCEPIY	ACATCGTTGCTGCCCATCGACCGTTCGTTTGAAGATTCTTTAAGGTTTTTGGTGATTTTGAGAGGAATGCCTTCACTGAACGGTGGATGTGGAGTTACAGCATGGTCAGATTGGTCACCATGCAACGCAACATGCGAAATGACAATGGGAATGAGATTGAGGACACGTTTATTCTTGAACCCCGCAATAAATATTAGCAGCTGCAACATCCATACTATTGAAACTGAACCATGTCGTTCTGAAAGAACAGATTGCAATGGAGCTCCTAAAGAAATATGTTTCGAACCAAAAGAAGAAGGTCCTTGCCGTAGTTACTTTGTGCGCTGGTACTATAACGTTCAAGAAGGAAGATGTCTACAGTTTGTTTATGGTGGATGCCGTGGAAATAGAAATAACTTCAAAAGTTTTTCCGAATGTCATAGGATATGTAACAATGTTCTGAGAGAGCCTCCATACAACATGCCAATCCCTTTGTCTCCTGATGATCCAACTCTAGTTATTGACTGTAGATTAACGCAGTGGGCTCCATGGTCAGAATGTTCTAATACCTGTGGCGTTGCGTGGAAGGACAGAAGACGCACAGTTGAAATAAATGCACAGAATGGTGGAAAACCATGCGATAGTAAATTATACCAAAGAAAAAGATGTAAAAGTCAACCACCTTGTGAACCAATCTAT
TatPInKun05	XP_013169137.1	3 e-22	PF00014	--	Kunitz-type like	IKVQKRSSVIFIKRSEGLLVIMFGSWLACSITIMIMLFNNVVYSWDDCDLIPSSEDCGKRTQMYYYDMRSDTCKEFTDFGCPGNKNKFKDKERCIKTCGNDCSLFPDSGVCFEDHRLYYYNPETNKCKIFSYGGCGGNRNKFVTEAECLKTCERTWMNDNKE	ATCAAAGTACAAAAACGAAGTTCTGTCATTTTCATTAAACGGAGTGAGGGATTGTTAGTCATAATGTTTGGATCATGGCTCGCATGTTCGATTACAATAATGATAATGTTATTCAACAATGTTGTTTATTCCTGGGATGATTGTGACCTGATACCAAGCTCAGAAGACTGCGGAAAACGCACACAGATGTACTACTATGATATGCGATCCGACACCTGTAAAGAGTTTACCGACTTTGGATGTCCAGGGAATAAAAATAAATTTAAAGATAAAGAAAGGTGTATCAAGACTTGTGGAAATGACTGCAGCTTATTTCCCGATTCTGGAGTATGCTTTGAAGATCATCGATTATATTACTACAATCCAGAAACCAACAAGTGTAAGATATTCTCATATGGTGGATGTGGAGGAAACAGAAACAAGTTCGTCACTGAAGCTGAATGCCTTAAAACATGCGAAAGAACTTGGATGAACGACAACAAAGAA

Venom components

ID	ID/Reference protein	e-value	PFAM domain	MW (Da)	Sub-classification	Amino acid sequence	Nucleotide sequence
TatVeCOth01	ABR21061.1	3 e-29	PF15430	12495.38	Venom protein-7	MAWKLLFLFVLPCIVFGTDEPAR**FIAYKNDVVSPLTAGKCKIGEDKMIEQGETWYREDFCEKVYCLRTGNLGHMEVRGCTPIAPTNPNCTVVYHKGLYPDCCSGDIVCNQPPPEPKSDVEMAELFRSLLQNKKK**	ATGGCGTGGAAATTACTGTTCCTGTTTGTTTTACCTTGTATCGTTTTTGGAACCGATGAACCTGCGAGATTCATTGCATATAAAAATGATGTAGTTTCTCCACTGACAGCAGGAAAATGCAAAATTGGTGAAGATAAAATGATTGAACAGGGTGAGACTTGGTACAGAGAGGACTTCTGCGAAAAAGTATACTGTCTCCGTACTGGAAATCTGGGCCATATGGAAGTAAGAGGATGTACTCCTATTGCGCCCACTAACCCTAATTGCACTGTCGTCTACCATAAGGGGCTATATCCTGACTGCTGTAGCGGTGATATCGTTTGTAATCAACCTCCTCCTGAGCCAAAATCAGATGTGGAAATGGCCGAGCTGTTTAGATCCCTACTACAAAATAAGAAGAAATGA
TatVeCOth02	AOF40196.1	2 e-8	PF15430	--	Venom peptide HtfTx2	MENNKGRKCSYASELKENDEIWYNPNFCEQLTCQIEGDKAYIVIIDCGVPAVPHRSCTVSVGKGNYPDCCPKINCNEIMNTAE	ATGGAGAATAACAAAGGAAGAAAATGTTCCTACGCATCAGAATTGAAAGAGAATGATGAGATATGGTATAATCCTAATTTCTGCGAGCAACTGACTTGTCAAATAGAGGGGGATAAAGCCTACATTGTCATAATTGATTGCGGAGTACCAGCAGTGCCACATCGAAGCTGCACAGTATCTGTAGGAAAAGGAAATTATCCAGATTGTTGCCCAAAAATAAACTGTAACGAAATTATGAATACAGCAGAATAA
TatVeCOth03	API81329.1	4 e-32	PF15430	8721.03	Toxin protein	MKSTAVVFFSLYAATYLIFSQA**RQEICMAGEELTLYLGEEKQDPNACALYKCERYFSRIVLHTLTCAPQELSSGCRNVDGPIDAPFPDCCPMVVCNAPVFG**	ATGAAATCGACCGCTGTGGTATTTTTCAGCCTGTATGCCGCTACCTACCTCATCTTTTCCCAAGCTCGGCAAGAAATTTGCATGGCTGGGGAGGAGTTGACCCTATATCTCGGCGAAGAAAAACAAGATCCTAACGCATGCGCATTGTATAAATGCGAAAGATATTTTTCGAGGATCGTTCTTCATACACTGACCTGCGCACCTCAGGAACTGAGTAGTGGATGCCGAAATGTTGATGGACCAATTGACGCTCCTTTTCCAGATTGCTGTCCAATGGTAGTATGCAACGCCCCTGTGTTTGGA

TatVeCOth04	AOF40196.1	3 e-9	PF15430	9766.03	Venom peptide HtfTx2	MMLLCCLITAFALTFSASG**YVFNSTVSLIDGKCSYASEL KENDEIWYNPNFCEQLTCQIEGDKAYIVIIDCGVPAV PHRSCTVSVGKGNYPDCCPKINCNEIMNTAE**	ATGATGCTCTTGTGCTGTCTAATAACCGCATTTGCCTTGACGTTTTCA GCGAGTGGCTACGTTTTTAATAGTACAGTTTCATTAATAGATGGAAA ATGTTCCTACGCATCAGAATTGAAAGAGAATGATGAGATATGGTATA ATCCTAATTTCTGCGAGCAACTGACTTGTCAAATAGAGGGGGATAAA GCCTACATTGTCATAATTGATTGCGGAGTACCAGCAGTGCCACATCG AAGCTGCACAGTATCTGTAGGAAAAGGAAATTATCCAGATTGTTGCC CAAAAATAAACTGTAACGAAATTATGAATACAGCAGAA
TatVeCOth05	C5J895.1	1 e-28	PF15430	--	Venom toxin OcyC11	MKATCFLLISLVVFYTANA**AMIPKGFCRTRTGELLRD GQEWKDPHQCAIYKCSIYDGESELNGMICAVFQVPP NCREVPGRGRLYPECCPTVVC**	ATGAAAGCAACGTGTTTCCTTTTAATATCCTTGGTGGTCTTTTATACT GCAAATGCAGCTATGATCCCGAAAGGATTCTGCAGAACAAGAACAG GAGAACTTTTACGAGATGGTCAAGAATGGAAGGACCCCCATCAATGT GCTATATATAAATGCAGTATTTACGATGGTGAATCTGAACTTAATGG CATGATATGCGCAGTATTTCAGGTCCCGCCTAATTGCAGAGAAGTTC CAGGTAGAGGAAGGCTTTATCCAGAATGCTGCCCCACCGTAGTGTGC
TatVeCOth06	CAX51433.1	1 e-19	N/D	6768.55	Venom component	MWKIVFVCFAVLVSTICKG**DESMEEGRTINLLFSEDGR RTLGCWFTYAFSYNPTADIPTKTEGQKKLCECMQKA LSGSN**	ATGTGGAAGATTGTCTTTGTTTGTTTCGCAGTTTTAGTATCTACTATTT GCAAAGGAGATGAGAGTATGGAAGAAGGAAGAACAATTAATCTCTT GTTTTCGGAAGATGGTAGAAGGACATTAGGGTGCTGGTTCACTTACG CCTTTTCTTATAATCCCACTGCTGATATTCCTACAAAGACAGAGGGCC AGAAAAAGCTGTGTGAGTGTATGCAAAAGGCACTATCTGGAAGTAA TTGA
TatVeCOth07	JAV47658.1	2 e-46	N/D	--	Venom protein	LLMMYALYLLLLFGFMHVAIIKTKESFPYHAAKVGRDC DFKIFPPLKCNEQCWREGYNWGTCVGLLEGLCWHQKC CCFTKPIQELEIDDDPYVDMTIQVEDPNVKTNPDERICQ	TTGCTGATGATGTATGCTCTTTATTTGCTGCTTTTATTTGGATTCATGC ATGTGGCGATCATAAAAACAAAGGAATCATTTCCATATCATGCAGCA AAAGTTGGAAGGGACTGTGACTTTAAAATCTTCCCTCCCCTGAAATG CAATGAACAATGTTGGCGCGAAGGGTACAATTGGGGTACTTGTGTTG GTTTATTAGAAGGTCTATGTTGGCATCAAAAGTGTTGTTGCTTCACTA AGCCAATACAGGAATTAGAAATCGATGACGATCCATACGTGGACAT GACAATACAAGTTGAGGATCCCAATGTAAAGACAAATCCAGACGAA AGAATTTGCCAA
TatVeCOth08	JAV47706.1	6 e-23	N/D	--	Venom protein	MYALRIAVLSLLVYGVICELKMLTASRRYCAKTCDKKS ARHCAEDIMPGFLGLYVKCVQSIRPEANTWDEINDVYC SQVSQEEFLHQVMCFNLELNTRFKDGNHGDVCLKCLDE AGC	ATGTACGCTTTACGAATAGCTGTCCTGTCTCTACTGGTATATGGCGTT ATTTGCGAGCTAAAAATGCTGACTGCTTCACGTAGATATTGTGCAAA AACCTGTGACAAGAAAAGTGCTCGACATTGTGCAGAAGACATTATGC CAGGGTTTTTAGGTTTATACGTGAAATGTGTTCAGTCCATACGACCA GAAGCAAACACATGGGACGAAATCAATGATGTTTATTGTAGCCAGGT CTCCCAAGAAGAATTTTTGCATCAGGTAATGTGTTTCAATCTTGAGCT GAATACAAGATTCAAAGATGGCAACCATGGTGACGTGTGCTTGAAAT GCTTGGATGAAGCTGGTTGT
