# Supplementary material for: A Deeper Examination of Thorellius atrox Scorpion Venom Components with Omic Techonologies
Source: Toxins (Basel). 2017 Dec 12;9(12):399. doi: 10.3390/toxins9120399 (PMC5744119; doi:10.3390/toxins9120399)
Supplement: Supplementary file 1 [file toxins-09-00399-s001.zip › supplementary/TableS2.pdf]

Table S2. Sequences identified with the Proteome Discoverer software. Transcriptome ID corresponds to the identifier of the transcript. Fragments with a good Xcorr value (above 1) are highlighted in green in the corresponding protein/peptide.

Transcriptome ID	Transcript-derived mature aa sequence	Peptide fragments	Xcorr
comp8310_c0_seq1	DQDTSTADRDKRAPQMYSFGLGKKSYD<br>LPLEDSDRDKRAPQLYNFGLGKKSYDLP<br>LDDSTAPDDYMIGEPGVKKDQRFSFGLG<br>KRQDHRFAFGLGKRDPSGRYSFGLGKRE<br>PNRFAFGLGKREPSRFAFGLGKRQITEDF<br>DDYMKRRFSFGLGKRSDNRFSFGLGKRP<br>ENRFSFGLGKRPDNRFSFGLGRRKRFSDD<br>SDSYSEEDYNLSQ	KSYDLPLDDSTAPDDYMIGEPGVK	4.34
comp32030_c1_seq1	RWDLFNGTINVDDMNSKWWEYRLKYQ<br>GNCPSVKRTEKDLDPASLYHVPADISYA<br>RYFTALILQFQFHKALCDAAGHSGPLHK<br>CDIYGSSEAGERLKAMMSLGISKPWPEA<br>LKILTNGEVDDLDVAPMLEYFQPLQEWL<br>EKENKDEVIGWNSDDATICP	DLDPASLYHVPADISYAR<br><br>AMMSLGISKPWPEALK	4.67<br><br>4.1
comp32030_c2_seq1	IYQVLEEKIFAMQAGVVCFLLLSSVSISYS<br>AKFCNGGSCEEEQGIRLLEHYNAGEEDI<br>CSRHTLSIWDYYVNITDENQERESKLQEE<br>YDKFITDIVKEASQCGWSSFSDPMIRRQF<br>KFLVEKSSKQLSPEESKQEETLKYQMQG<br>LFGTAKVCPYDSNGTEKDNCTMTLDDGI<br>QQILESSTNFDELLYYWKAW	LQEEYDKFITDIVK	4.91
comp33161_c0_seq1	MCRFREASKQTSTSSSRMTTFLTFLVLVE<br>LFLNYEIHLVSSLISDEGKAEEFLDDVDQ<br>QHREWCLDNTKELDYRQIWSFDWQHFS<br>NPELRRAFKLLSRTEPASMNEPNQDDKS<br>KYEELEETLISGILGGSVCPYDQMCSKKL<br>KYSGINREVDPSIDYQKRLYYWKEWRD<br>ATGKSLKSTYSDFVDIANKINNGYESYG<br>DYWLREYDVSADEFLQELETLQSQLLPL<br>YQQLHAYVRRRLIDVYGEDKIKYNGPIP<br>EHLIGLIPQKWNKIMKIINPFENDFDVSIK<br>MKQKGMTVSEMVDLAEEFYKSIGTPELD<br>AAFWRDSVFEKSPQMESCFNEIREGCAG<br>NGARIRMCTNATEYYLHLLLQLLGDAH<br>QEKATSNLHYPFQGAANPGFLVAIGRTA<br>DLSAWTESYLRSVGLFDESYGDDRDISV<br>LLRIALENIPIIFSSLSMEKWRLDVFDKITP<br>FSSMNSKYWEKRVKKEGLCPPVRRTEN<br>DFDPGAHIHISTHSPVIEYFVGGILQFQFH<br>KALSEAAGCSDPLHKCSIYQSSAAGRIFN<br>EVMELGKSKPWPEVLSLLTSGRTNSLDT<br>GALLEYFRPLYEWLQQQNENEYIGWVA<br>DDSLQC	TADLSAWTESYLR<br>SIGTPELDAAFWR<br>YNGPIPEHLIGLIPQK<br>ATSNLHYPFQGAANPGFLVAIGR<br>IINPFENDFDVSIK<br>SVGLFDESYGDDRDISVLLR<br>QIWSFDWQHFSNPELR<br>SKPWPEVLSLLTSGR<br>IALENIPIIFSSLSmEK<br>YnGPIPEHLIGLIPQK	3.87<br>3.46<br>3.16<br>5.48<br>4.26<br>3.84<br>3.99<br>3.13<br>4.8<br>3.9

comp33725_c0_seq1	FLDHLVSWNVIILHQVINLLYMTIPLEFSV<br>HFGYPSIRRMRSILFHSVVLIFSFFIRHNLS<br>TLITDEKEAWELLLAEDEKMYNNCLKGE<br>YSLSDTTIDTVAWQNFTDPLVKRAFELN<br>AQKHYYVGSTLRISNTSDGQRRGEIWNE<br>KRNILSNTKVCTYETKDMDGNCTMERIF<br>DILRNYDKSENNDERKYYWVAWHNAIG<br>KMRGIYDEDLQILKRLNLFPDYNDWWL<br>YFYEEPTSHIREEMERVMQEILPLHKELH<br>AYIRRRLINQYGSGVITSDGPIPSQLLGLW<br>EYNGWRITFVPYPEKALNITEKLKEKNIT<br>PVGMLKLAEDFYHSLGLPYIDAEFWDNI<br>DLSTKELCYYNFRYTCKPIISGASGCMPIT<br>AGSLHKLIGAAIDIHWLKLRQTEMWPNV<br>LIDVPNPAFRMAFRHALELPTWTVDYLS<br>QIGLIEHIPMDSDEVTEFDINQLMEVAIRF<br>FTLLPSLLQTAKWKWDSLDFVLSNDNYN<br>RRWWEYSLKYQGVCPPVRRTEENFDPA<br>SSMHLANGLQLVRDFIGVIYGFQFYEAA<br>CKEAGHTDLLHRCNLYGARTFGDKIREV<br>YTLGKSINGSQGIAIMTNGESDKMDTKPL<br>LEYFRPLYEWLKNENEGETVGWKSDDP<br>MICP	LIGAAIDIHWLK<br>TEENFDPASSMHLANGLQLVR<br>FFTLLPSLLQTAK	3.23<br>4.17<br>3.39
comp33936_c0_seq1	NTGSRRLQKSHAYSGVRYILLESFITTDY<br>TKQLLNLQRVSKPSSEGGRFVMQAEVIW<br>SFFLCSFSFSVYCNAISHEEEEGIQLLERY<br>NSRRLDVCRRRSLVIWNSYVDSSQRNPT<br>MASKLSEESNNLTTEFVKKASQYDWTSF<br>SDPLIRRQFKFLVEKLSKQLSPEESEKEET<br>LRRQMGRTFRVATVCPYGINATEKQNCE<br>MTLDNGIESIIGSSTNFDELLYYWKAWR<br>DETGGKMRDLYAKFIPLANKEAVINGFP<br>DHGAQWRSVYEVDNLSNRVDELFNQVL<br>PLYKQLHAYVRNKLIGIYGEDHICTDGPI<br>PAHLLGHMMGESWANLENLTRPYPNKP<br>LVDITPVMQERNMTMLDIVKISEDFFESL<br>GLPPMTNEFWNHSIFERIPGRKMSCHPISF<br>DLCNGEDFRILMCGRVDMNSLKIVHHE<br>MGHIHYYMQYVHQPHLFRKGANEGFHE<br>AIGDTIALSVFTPTHWKALGFIQNETDDE<br>EQDINALFSTAMDKLPLLPHAYVVDRWR<br>WDLFNGTINVSNMNSEWWEYRLKYQG<br>NCPSIRRTENDLDPASLYHVPVDSSYVRY<br>FVALILQFQFHKALCNAAGYTGPLHKCD<br>IYGSHEAGERLKAMMSLGISKPWPEALK<br>VLTNGEVDDLDVSPLLEYFQPLQEWLEN<br>ENKDEVIGWSSDDATICP	AMMSLGISKPWPEALK<br>ASQYDWTSFSDPLIR<br>VDELFnQVLPLYK<br>KGANEGFHEAIGDTIALSVFTPTHWK<br>GANEGFHEAIGDTIALSVFTPTHWK<br>SVYEVDNLSNRVDELFNQVLPLYK	4.1<br>4.08<br>4.36<br>5.67<br>7.32<br>5.37
TatCaTClc01	ADCLAHLKLCKKNKDCCSKKCSRRGTN<br>PEQRCR	ADcLAHLK	2.19
comp32319_c0_seq1	MRSMIDVKILAGIFLVLGISDVITVSLAYK<br>LTGWKIPFQYGIILDAGSSKTQVTLYKWE<br>AHKDKGTGIVEQVDTCKVKGGLHNRNS<br>TIHAGEELLPCLMKISASIPEGQQENTPLY<br>LGATAGMRLLRLGNPLLAAGIILEVKYSL<br>LRNTNFLIRDVRILNGRDEGIFAWVTGNY	LGNPLLAAGIILEVK	4.36

	LLDTLHDGVNDHDPTATYGALDMGGAS TQISFELPPRKIAKIDNATTVNMTLFGQN YLVFGESYLCFGINEAMRRHRARLTIGK DPEGEITDPCGFKDDIVEYSVKDLFGHQC TRSLTPTLNKDGVYKFRGTGDSKLCSIEV EKLTDQEECKKVFKKPCFAEPKEKLSNV KYMAVSTFYFTASALNITNTSLQNYKNA IDYYCGLSKDEAHRRLKPEESQYVRDYC LEAHYVHHILTKEYGFDENTWQNIMFV MKVRGSDLGWSLGYMINATNTIP	EYGFDENTWQNIMFVMKVR	1.4
comp881_c0_seq1	YPASLLEDEDYEENSRVFRGRFAKQGEL PWMIQLQVSKGNGKAGMCGGSIISKRHV LTAAHCVCSNATTKAYANVNDITGRIGH INRADATPVKFKRLVVHPGYDADYNADI ALIEFKTPLTNYDNNIQNICLPKKGKSYP NRQPVLQMGWGRFDNGSVGTSPTLKITN VGTILDRTTCIREMRSYAEPGQLCISNAG GEKICGGDSGGPLVLVNGVNKMAIGIVS FDYFDWCVEDTEGPAIYTDASYYAEWIK TNTNDNGICWKD	TPLTNYDNNIQNIcLPK<br>RLVVHPGYDADYNADIALIEFK<br>QGELPWMIQLQVSK<br>LVVHPGYDADYNADIALIEFK	5.71<br>7.22<br>4.69<br>6.64
TatHDPND201	GIWSTIKKYASKAWNSDIGKSLRNKAAG AINKFVADKIGVTPSQAASMTLDQIVDA MYYD	AWNSDIGK<br>GIWSTIK<br>GIWSTIKK	3.03<br>2.65<br>2.02
TatHDPND301	LPFFLLSLVPTAISAIKKL	LPFFLLSLVPTAISAIKK	5.94
ViVlp1	GIFSWVKKAWNSGVGKSLRKQAVKAAK NYVANKLGGTPEEAGAMPFDEFMDVLH YN	KAWNSGVGK<br>GIFSWVK<br>AWNSGVGK	3.27<br>2.5<br>2.1
ViAMP1	FWGFLGKLAMKAIPSLIGGNK	AIPSLIGGNK<br>FWGFLGK	3.01<br>2.79
TatHDPND401	FLKGIIDTVGKWL	GIIDTVGK	2.31
TatHDPND403	FWNTLLSVGKSLL	FWNTLLSVGK	
ViCT2	FWGAVWNAAKSIL	FWGAVWNAAK	2.06
TatEnzHya01	NFEVFWNVPSLLCSIKFGVNLTQTLLKY KILVNNGESFIGDKIALIYENGIGKYPHID PKKGDINGGIPQLDKLNEHLKLAENDIQK LIPNPDFNGLGIIDWEAWRPIWEYHWGS LGIYKNRTLEMVKKDHPTWSEQLVQSTA KNIWENSAKQWMLKTLELAKKLRPHGR WCYYLFPDCYNYFGKDQPSQFFCSAMIQ NNNDRLSWMWDASTALCPSIYFIENQM KYNGSQRTWFLYGKLAEAVRVARPHTRI YPYINYMVHVSRIPVPEDHFWKMLSLIAS LGLDGAIIWGSSSYLSDITSCQDLETYVN NVIGPAVTTVSSNVERCSQMVCNGRGKC TWPNDPFTSWKYLTDINSDFDSKEITCRC QTHKGRYCD	WcYYLFPDcYNYFGK<br>ILVNNGESFIGDKIALIYENGIGKYPHIDPK<br>IYPYINYMVHVSR<br>cTWPNDPFTSWK<br>YLTDINSDFDSK	4.84<br>4.26<br>4.22<br>3.38<br>2.29
comp15335_c0_seq1	LQTVSSTEMKSIILAVVVVAVASQQLGE DPLCRAPESVVNRYIECMRSNYKPAFDY ALTCSRELGAGALADFIKFSCGKLRATKE QETKYGNCLTRAIDPSNALSEEDLSKVVE SCRQQALASQ	ELGAGALADFIK	3.27
comp30560_c0_seq1	NMNVINVTFMTFVVVFTSPYLHIHVFAE NLSVDHWEFVCSSSEETILGLLDCSMPEE TKDSLKRINGLMECTKMPQIEIIQSLCKID SLPEAKVNLIDSCLEKHFADVDESQPNPV	MPQIEIIQSLcK	4.02

	VKCIEQKPEFHSMRAEK		
comp31101_c0_seq1	EQELRINLYRRMLTCITIGSFILITSVTSIQ<br>CCHHTTTLNNTDVENTNTTSDAFQDSCIL<br>ADPNDYVDDLISKLKNKTIDTIPLPDDPG<br>STSLKEGQLWGLSSLKRVGNATIYCNET<br>LTSVEVLISFEELRGRYNWERKVLLKTFS<br>GQVFVNADTADILFRVSQEHANSTGIVLE<br>ELSIKNLSGIHTKVTGLGVVTWAISVITDP<br>VANLFKKTLSTAVEGPLKDAIGLALRDL<br>DVPLY	TIDTIPLPDDPGSTSLK<br>TFSGQVFVNADTADILFR<br>TLSTAVEGPLKDAIGLALR	3.44<br>6.03<br>5.01
comp30730_c0_seq1	VLTSSTMSRQEFVIVFLALYCISSFSVIKV<br>SATSSIYAPLVKVSVYYETLCPDSRDYIT<br>KQVWPTYQKVSDIMELELIPFGIATETPL<br>NSTHYIYTCHHGKDECYGNAVHTCAIHL<br>LRNMSLALNLINCMEMKPDHMQGRKCA<br>TDLEINYLSIEACANARLGNELQHEMAT<br>KTKNLNPPMDFVPWNVINGNSDSGMQK<br>RATDNLLSVVCTLYKGEKPKPCSSA	ATDNLLSVVcTLYK	4.28
TatKTxScr02	GLIREKYFHQGVDALTPLIPVPVVGGVV<br>NKVAKQMIHKIGKIQSLCAFNKDMAGLC<br>EKKCQETEKVKGYCHGTKCKCGKPLSY<br>K	YFHQGVDALTPLIPVPVVGGVVNK<br>EKYFHQGVDALTPLIPVPVVGGVVNK<br>YFHQGVDALTPLIPVPVVGGVVNKVAK<br>IQSLcAFNK<br>KcQETEKVK	7.67<br>6.73<br>4.51<br>1.96<br>1.48
TatKTxScr01	GLIKEKHVQKGVDALTNLIPAPVVGGIIN<br>KVAKQMVHKMGKVQELCAFNKDVMG<br>WCDKACLEKEQTNGFCHGTKCKCGKPL<br>SY	GVDALTNLIPAPVVGGIINK<br>VQELcAFNKDVMGWcDK	5.76<br>3.94
ViLa1lp1	VGEICQVGSMSINVGKKMQDPKSCVIYE<br>CVEQNYRILLSKMSCSPQVPKRGCRNVP<br>GPVDAPFPDCCPTSLCRGKQWDE	NVPGPVDAPFPDccPTSLcR	3.87
TatOthLa101	FGETCQAGGKYNVRVGQPIQDPNSCVLY<br>KCLNYNRRYVLQTLSCATQTLKSGCRFA<br>AAAPGTPFPNCCPMVICKGSG	FAAAAPGTPFPNccPMVIcK<br>YVLQTLScATQTLK	4.67<br>5.89
comp34524_c0_seq1	MTTEEGKVVDVLDTEPFAEQKSGCGNGI<br>TSSREKNLVIIAFVFGLLVLCLLCAIIMTV<br>VIGLLFPKREFCLTDHCVKKAGSILRIMD<br>TTVDPCVDFFRFACGGWMDEYDVDENS<br>ETAILGKMQLKIYRKLDKIIERVKSSLPTL<br>NLTDPKQIAEAGLKAIDSYDACNQLSTA<br>YDPENIIPFMASFGGWPMVDDKWKEDP<br>NMKIETKISSLISEFGVAPLFIITINPLEDDP<br>TRNVLVIAPPSLSTSFEVYLNASAIIARKM<br>KKVAEEDIDEDVKNVIKLRDEINKTLTAS<br>IEDDSSELTVADLKLHLKNMDINWEKVF<br>ASLLKSGKLAADEDTVKDLPVIVRNKAT<br>IDAVLGVVEYNKDLRALSNYFAIEVLLN<br>HPLLVVNVTDGSSNNQAKALKNNNILTN<br>MKENCLATVSEYLNFAMDHVFVYNEPIS<br>TGKAGEFIKYIREAFKQLIRKYDWIDDIT<br>RRALLKKLEKMNNFIDHPPWIIDKEKLN<br>AYYQDYTYTKGNPVATYFSMVAFGVHK<br>SLETYNQISNRNKWPEIVSFSVSSVNAFYI<br>PHQNNFVLPRSILHPPIYDENNPNYLSFGS<br>IGAVIGHEITHGFDSEGRNYDEIGKVSSSL<br>WTPTSTKEYNKLSQCFVTQYSNYSFGGN	NKATIDAVLGVVEYNKDLR<br>ATIDAVLGVVEYNKDLR<br>NKWPEIVSFSVSSVNAFYIPHQNNFVLPR<br>SILHPPIYDENNPNYLSFGSIGAVIGHEITHG<br>FDSEGR	5.96<br>4.9<br>6.26<br>5.72

	VTVNGKTTLAENIADNGGLRQALRAYRL WLKRNERELPLPGLAKYTPEQMFFISYG QSWCISAGKKFLKKQVKTDEHTPNTYRV IGTLSNMEEFAKEFQCKKATPMNPVNKC VLW		
TatEnzMtp04	LKGCFTEPQDAICGNEVVEKGEECDCGW EEDCEEPCCFPMRSNPPRDEPPCHLRPNV VCSPSQGPCCTHDCRIKVGEECRGDNGC RSASYRDGQGPHCPSSTNKPNKTVCNDE FVCYMGECTGSICMAYGLESCQCKRGLH DPLTKACELCCKLPRD	LKGcFTEPQDAIcGNEVVEK	1.61
comp32637_c0_seq1	FNLTILHTNDVHSRFEQFNTFGSRCTESS AEKGECFGGVARQYTKLKELREKYPNSL FLSAGDYYQGTFMYTLHKWKIVADFMN RLGHDVMAIGNHELDDGVAGLVPLIEKA EFPIICCNLDVSREPSMKGKVSPFVIKEVD GRKVGIIGYTTPDTMFLSRAEKLVFTDEI ECLKDAVKSLKGSGVDIIIALGHSGFPKD VEIAEAVEGVDIVVGGHTDTFLYSGDPPS VEEPQGEYPTVVSHADGTKTLVVQDYTF GKYIGFLKVKFDDKGNVKSWEGNPILLD NSVEQDPEILGALQPYVDAVSSIAKETVG NTKVLLRGDRTVCRMEECNLGNMLADA LVDYFTDSPKQYGWTSAAISIWNSGGIRS SIDETAAEGNITVEDIMNVAPFSNTFSLAE LRGEDLYTLMEESVSEYDASAIDPPGKLL QVSGLKVGYKMDQPPFHRVSELQVRCA KCRVPKYEKVDKKVVYRIVVPAYILGGG DGITVFKEKAVTVFNTGVLDSDVIQTYLS RHSSITTGVEGRIYFITPPAQSNSTSKRYIR SFRFNK	LVFTDEIEcLK<br>AEFPIIccNLDVSR<br>KVGIIGYTTPDTMFLSR<br>VGIIGYTTPDTmFLSR<br>AEKLVFTDEIEcLKDAVK<br>SLKGSGVDIIIALGHSGFPK<br>VGIIGYTTPDTMFLSR<br>GSGVDIIIALGHSGFPK<br>LVFTDEIEcLKDAVK<br>EKYPNSLFLSAGDYYQGTFmYTLHK<br>LGHDVmAIGNHELDDGVAGLVPLIEK<br>QYGWTSAAISIWNSGGIR<br>IVVPAYILGGGDGITVFKEK<br>TLVVQDYTFGKYIGFLK<br>IVVPAYILGGGDGITVFK<br>LGHDVMAIGNHELDDGVAGLVPLIEK<br>AVTVFNTGVLDSDVIQTYLSR<br>mEEcNLGNmLADALVDYFTDSPK	3.08<br>4.33<br>4.61<br>4.56<br>4.89<br>5.2<br>4.94<br>4.79<br>4.63<br>7.05<br>7.12<br>5.6<br>4.78<br>4.29<br>5.39<br>7.05<br>5.81<br>4.34
comp26928_c1_seq1	LLMMYALYLLLLFGFMHVAIIKTKESFP YHAAKVGRDCDFKIFPPLKCNEQCWREG YNWGTCVGLLEGLCWHQKCCCFTKPIQ ELEIDDDPYVDMTIQVEDPNVKTNPDERI CQ	EGYNWGTcVGLLEGLcWHQK	4.98
comp27809_c1_seq1	EQTMAYRCLHIVLLFLAVLGTVRTQLID QCSAVKLTVDKDMYEVNERIVSTCEGCA PIILRMRSAEGEVKVRDRNFNVGMVKIK QAYEATSDLEILCYSDRIGELKEMTLVKC ASNNDLLCPFQGHNKNG	IVSTcEGcAPIILR<br>QAYEATSDLEILcYSDR	4.05<br>6.08
comp30392_c0_seq1	DESMEEGRTINLLFSEDGRRTLGCWFTY AFSYNPTADIPTKTEGQKKLCECMQKAL SGSN	TLGcWFTYAFSYNPTADIPTKTEGQK	6.77
comp32982_c0_seq3	ELKMLTASRRYCAKTCDKKSARHCAEDI MPGFLGLYVKCVQSIRPEANTWDEINDV YCSQVSQEEFLHQVMCFNLELNTRFKDG NHGDVCLKCLDEAGC	HcAEDIMPGFLGLYVK	4.95
comp43100_c0_seq1	MLNIHFHKLSVNINQCSAFIRCTLLFVSH QRSVHIFAVMMLTYLSVVMCITMLSLSA RSQRCVDGCPEVPNGVIETALVGTGKSC EDVTDATVRCGCTSTCEMKETFCTGCTK ACHCEDIDLFFDKNLLSCVSVNDCTH	cVDGcPEVPNGVIETALVGTGK	5.25
comp31198_c0_seq1	MESKQYFVISVYIMGFAELIISTNVYCPD DQLCDCTPESIKCICLQRENIFFQKGETFM GEKIIVKGCGEVKIATSFTKNLVVESFLIS	NLVVESFLISDVLK	5.3

	DVLKLHIAQYAFKGSSIRKLSIGNISDFTL<br>EPFSLSHIKNVSNLKINNVFLEHLPNFVT<br>WNTDAQNVAIMNSHVGAILAGAFKMEN<br>MESFLLENNTVDDLSGSIEISNIKNFSFIR<br>NVVKNSGFYSISLYMADKVEFAYCHFLQ<br>INSSMIYTHDIELFSFHDNHVESCEKKAF<br>WHVQANTKVTFVNNQIISANNDSLIPNFS<br>PKAIDLRIIESFNNTFVCDCNLEWLLNNS<br>NPSYNFIKERSICYDPPYLANLKLSQLKLS<br>INNVGTCISVEKVSVSSAMPKITKLKYILE<br>TSKTSSNTEISTSPRSETLISKKDSALSPKT<br>EISTNQKTVASISPKAEAIHETDLSINPKSE<br>TLPSRKIVTPISPKTMTPASHSAHNEKRL<br>KQNLNSTSADMSTNIKSERPKSSKIMIPT<br>NSKADTSKESSKIDISRIPKSAVSTIQKSDI<br>SINPKMETPKQSKAEISAKFKRNAETSTE<br>TESSQTNTAVTQKTDPKADVSVTPETDS<br>KVQNSESSTTDKSITTEPKSSESSKENTPT<br>SLKEATPGNPKTDLLTTPKSEISASTKPKT<br>TINSKAETATNSKTETTTSPKTETTTSSKT<br>ATTTSPKSQTVTSSKMETTNPKTETA		
TatEnzPA201	TLRTFHGCQILQSLTDIAREVSILPKYAIR<br>RISKQELESFQSRCEHVGEEERTIWGTKW<br>CGAGNESSGYEDLGYFYNVDSCCRDHD<br>HCDSIPSGGTKYSLKNEGKFTMMNCECE<br>DAFAKCLDKVVEGTSWYDYLATLGLIKS<br>FKGVYFNLYGNGCFHVKCNSGRSERRAL<br>CANPTAEYTGESGFAKLLNG	ALcANPTAEYTGESGFAK<br>TIWGTK<br>VVEGTSWYDYLATLGLIK<br>cLDKVVEGTSWYDYLATLGLIK	5.74<br>1.68<br>6.67<br>5.66
TatEnzPA213	AERELHVNFDPWPVARAAIVNFDYNSET<br>RREFSDCRMITSLDEITREGLDLPEHLIKR<br>VSKEEMDALEKRCSGSAEMERFGMIYPG<br>TKWCGPGNISSSYSDLGELEADKCCRDH<br>DHCDSIPAGQTKYGLSNTGEYTLMNCDC<br>EKAFDSCLGDAADKEYLWNSMRTKTLR<br>WTYFSAYSPKCYSVSCSAKRLDMEARC<br>ANGIGEWKSSYNV	WTYFSAYSPK<br>AFDScLGDAADKEYLWNSMR	2.95<br>5.85
TatEnzPA215	MYLAVFTALLSLCCSRAAQRELYINFEPL<br>PGQRDSWPIARAAIVNFEEKSEMGREFSG<br>CRMISSVDELAREGTDLPEHLIKRASKEE<br>MDALQERCSGSAEMERFTMIYPGTKWC<br>GPGNKAKNESDLGSLEADKCCRAHDHC<br>DNIGAGKSKYGLTNTGSFTLLNCDCENT<br>FDRCLSDAAEKEGWFKKQGTKALRWTY<br>FTAYSPKCYSLSCNKKRSILEARCANPVG<br>KWKENYKL	YGLTNTGSFTLLNcDcENTFDR<br>WTYFTAYSPK<br>FTMIYPGTK<br>WKENYKL<br>cLSDAAEKEGWFK<br>SKYGLTNTGSFTLLNcDcENTFDR	7.49<br>3.08<br>3.22<br>2.31<br>3.18<br>7.98
TatEnzPA202	TFSKCRMLNSTKEAAREVSKFPQHLIKR<br>VSKEEMDNLERRCSGPLETRGFTDNFAF<br>KGTKWCGPGTMAENEDDLGPLEADKCC<br>RAHDHCDSIESGGFKYNLKNNAYRTLLN<br>CECEEAFDRCLQATADRVEGTEKEETKM<br>LRNYYFNILHVQCYRLYCRNGGPASENN<br>CTDKFGVWMENYYEENFERKDITFY	NYYFNILHVQcYR	5.16
comp20627_c0_seq1	IQIDKAYQVELIEGLNLFNASYRGVALVD<br>GYHHWSPAVLLRGELRRLQLPIEVCRKA<br>SEILSKSNEFTLMATLRQEQRNAGTILSFS<br>EGNNRFLELQSSGRKHEIRLHYNHNNMV	KASEILSK	2.82

	HVETFPYHLADNSWHQLSMTVSGNNVA LYVDCNRIYKRVINDIDRNFTSKNLSLWL GQRNYQHFFFKGILQDVRIVGKPHGYVL QCPHLETDCPTCGQFKQLQISVRGLENH VRDLIERLAHAEQRIAAVEECECKKHCLF NGTEHQDGSTWQNGCEICFCKLGNVKC KPIHCPHLECKLPVMKEDECCPQCPNQC LLDGIEYDHGDEFVARQCVICTCHDGSV QCRKIDPAEACPALPCPEEEQFTVPEECC RYCPGTDFCARGHNCHINATCLNLQTRH ACQCNMGYQGNGVHCEDINECKTEGGH HGHHCRLNTTCVNIPGSYICECLDGYRR MDRFSCIEHDECRSNDHNCDENAICANT AGSYTCHCKDGYRGDGFICDPICNETCL NGGQCVAPGLCGCHRGYTGPSCEIDIDE CSLGLHMCHPNAQCVNMPGWYYCECR RGYQSHYKNRLGIFCEDIDECSLESHTCH YSATCINEDGSFRCECGNSTSCSYNCIYY GEEKNNGETWTSPTDPCSHCSCYSGVVL CEQSSCDCSNSNVDLTCCPQCDETSICKH QETARLFRNGDKWTYQCQTCECLYGEID CWEIECPPVSCDNPVHHPGDCCLRCQEE PCLLDVKSNKNNSLNIGCTYHGHMYRSG ETIPIVQDPCASCKCQVPHCEHSVSNTGY SSHRMDSSAARTVPFVWTILLSLLLTCPY NTIVRIWRMFLIIINKLYPQKKMLRWKTQ LHYLYICFLPLLQCPLWRMGSYFNNCFP CQRTSQLKIASKVNLAAGSLNTQKIPI		
TatOthCRI06	MASVIITILVLWVTTIQSFEANDECDERY SSITQEHTMCKSINENCHFLRHGGKTYEQ QLLRTHNLIRNSIWKYVGKNYPLATNME IMQWDDELYEIARMHSLQCVEQPDCDLC HQIGYFPVEQNFAVKTYKKSEVANNGPV KRFQTVIKEWAAELKQYDPSIVNDFTVT EELPTNWTNILRANTSFVGCASMNFYTD ETDVFTEVYVCNYGPAKLTTGEEIYKTG GKPCSDCEDDGICDEEFKNLCVPADLEL NITIVIPEEEDTEIWLGEGNYNGTSLSEEIE GTTSPNEFTTAESAKTFLYRQTGTPEETH TAIGEENSSVVFTETAPEETSIVEFTTELS NEPVTTEVEESGERRKRKVHPLISRLLRY SNKRRKRHQEHSLFPMN	MHSLQcVEQPDcDLcHQIGYFPVEQNFAVK	4.59
TatOthCRI07	MNFLLLSCLVLFSCQAVFAQTCPALYLR FSKDHTYCRHSTCQTEKSGVSEKDKEIIV NMHNEFRSKIAMGKETTPMQQPPAANM MQISWDEELAAVAQAHANGCKFDHDTA PQRQVENFNVGQNLYITMMSKRIDWRK AALWYTSEVKDFYPQYREPFVFGTYGHF SQVVWAETWKVGCGMAMYYDQSDNM DKVLYTCNYGPAGNMQGAAMYIKGQPC SQCPKNTKCSDEYEGLCKPITKDGPQNDI AKSSNDFIFYCEFSKNDPSECSKVKVEGT KGFETRKIYSGIYKSVILNGGESITIKLGK AQDAGGICPFIYGSFGPNKDGDAKMSAV SFGFSAPGIIFGDPIKIEQGGSAFWTIGMH MQFDQEMESTIKLEAYPGATPQYFNVKA	mSAVSFGFSAPGIIFGDPIK	5.75

	FGIGKGKCPKL		
comp30427_c0_seq1	QPQPNFIIFIADDLGIGDVGCFGNNTIKTP NIDNLASQGAILTHHLAAAAVCTPSRAA LLTGRYPVRAGMEAGNRNQVLLFVASS GGLPSNETTFAKILQGKGYSTGLIGKWH LGNDCNEKGDMCHHPLHHGFDYYYGIP LTNLKDFGTDGDSVITAYNPKLYTYFTT AVVLLISLSLGLLSHGYIKSFSFVFFITSCA MFVFYVFVVNFGRLNSIILRNTEVVEQPV RLNGLTQRLIQEGITFMKENSGKPFLLVM SFIHVHTALFSAQEFSGKSRHGKYGDNV EELDFAVGEIMTAAESFGHKENTFVYFTS DNGGHLEERGIEGDVQGGYNGIYKGGK GMGGMEGGIRVPTVVSWPGKIPAKLEV AVPTSQMDLLPTVLEAAGIPLPNDRIIDG KSIMLLLLGKRNISPHQYLFHYCGRSIHA VRYIPNNGNSIWKVHYATPRWKPNTTEC DFICHCFDDFVIHHNPPLLYNILDDPSESH PIDVRSNPKWLKLITTINKAVKEHSRSIEH VPQQFTFSNAVWKPWLQPCCNFPWCHC VDNRYPS	WLKLITTInKAVK	2.85
