# Supplementary material for: A Deeper Examination of Thorellius atrox Scorpion Venom Components with Omic Techonologies
Source: Toxins (Basel). 2017 Dec 12;9(12):399. doi: 10.3390/toxins9120399 (PMC5744119; doi:10.3390/toxins9120399)
Supplement: Supplementary file 1 [file toxins-09-00399-s001.zip › supplementary/TableS3.pdf]

Table S3. Sequences identified *de novo* in the proteome of *T. atrox* using Peaks Studio software.

#	Peptide	ALC (%)	Length	m/z	z	Mass (Da)
**1**	LKEEYDKFLTDLVK	97	14	871.0	2	1739.9
**2**	AFDSC(+57.02)LGDAASKEDFLNSMK	97	20	1103.5	2	2205.0
**3**	MPQLELLQSLC(+57.02)K	96	12	730.4	2	1458.8
**4**	LSEDM(+15.99)FESLGLPR	96	13	755.4	2	1508.7
**5**	TPLTNYDNNLQNLC(+57.02)LPK	96	17	1009.5	2	2017.0
**6**	LSEDM(+15.99)FESLGLPR	96	13	755.4	2	1508.7
**7**	NYYM(+15.99)NLLHVQC(+57.02)YR	96	13	895.4	2	1788.8
**8**	KQSDAAEKEGWFK	96	13	762.3	2	1522.7
**9**	AFDSC(+57.02)LGDAADKEYLWNSMR	96	20	1175.0	2	2348.0
**10**	HGC(+57.02)LADFDVGGGC(+57.02)EKHC(+57.02)R	95	18	692.3	3	2073.9
**11**	KGELPWMLQLQVSK	95	14	828.9	2	1655.9
**12**	NYYM(+15.99)NLLHVQC(+57.02)YR	95	13	895.4	2	1788.8
**13**	MPQLELLKSLC(+57.02)K	95	12	730.4	2	1458.8
**14**	WQLLGLGSTLLSK	94	13	708.4	2	1414.8
**15**	HGC(+57.02)LADFDVGGGC(+57.02)EKHC(+57.02)R	94	18	1037.9	2	2073.9
**16**	LQEEYDKFLTDLVK	94	14	871.0	2	1739.9
**17**	KLKDALDKR	94	9	543.8	2	1085.7
**18**	WLGTLLGLGSTLLSK	94	15	780.0	2	1557.9
**19**	HGC(+57.02)LADFDVGGGC(+57.02)EQHC(+57.02)R	94	18	1037.9	2	2073.8
**20**	WC(+57.02)LAHLK	93	7	464.2	2	926.5
**21**	LYPYLNYMVHWR	93	12	827.9	2	1653.8
**22**	HSWSFDWQHFSNMHAR	93	16	1037.0	2	2071.9
**23**	LVFTDELEC(+57.02)LKDAVK	92	15	890.5	2	1778.9
**24**	KLWSFDWQHFSNPELR	92	16	1045.5	2	2089.0
**25**	LWGTLLGLGSTLLGSK	92	16	808.5	2	1614.9
**26**	TNTLAELQVTC(+57.02)FMYR	92	15	923.9	2	1845.9
**27**	EAFPLLC(+57.02)C(+57.02)NLDVSR	92	14	847.4	2	1692.8
**28**	HGC(+57.02)LADFDVGGGC(+57.02)EQHC(+57.02)R	92	18	692.3	3	2073.8
**29**	LLGAALDLHWLK	92	12	675.4	2	1348.8
**30**	WGTLLGLGSTLLSK	91	14	723.4	2	1444.8
**31**	EPVGALLGLGSTLLSK	91	16	778.0	2	1553.9
**32**	LLGAALDLHWLK	91	12	675.4	2	1348.8
**33**	KVVVESFLLSDVLK	90	14	788.5	2	1574.9
**34**	QQSDAAEKEGWFK	90	13	762.3	2	1522.7
**35**	TLVVQDYTFGKYLGFLK	90	17	996.5	2	1991.1
**36**	LYPYLNYMVHVDK	90	13	827.9	2	1653.8
**37**	KLQDALDQR	90	9	543.8	2	1085.6
**38**	LVSTC(+57.02)EGC(+57.02)APLLLR	90	14	794.9	2	1587.8
**39**	WYNWGTC(+57.02)VGLLEGLC(+57.02)WHQK	90	19	1204.1	2	2406.1
**40**	TPLTNYDNNLKNLC(+57.02)LPK	90	17	1009.5	2	2017.0

**41**	WTYM(+15.99)M(+15.99)DTVGK	90	10	632.3	2	1262.5
**42**	LWGALLGLGSTLLSK	90	15	765.0	2	1527.9
**43**	DYYFNLLHVQGYMK	90	14	895.9	2	1789.8
**44**	LWGTLLGLGSTLLSK	90	15	780.0	2	1557.9
**45**	AVWGALLGLGSTLLSK	90	16	793.5	2	1584.9
**46**	AEGTPELDAAFWR	89	13	731.9	2	1461.7
**47**	LVFTDELEC(+57.02)LK	89	11	683.9	2	1365.7
**48**	RDALTNLLKEEVNLLNK	89	17	992.1	2	1982.1
**49**	NLNPFENDFDVSLK	89	14	826.4	2	1650.8
**50**	YVLQTLSC(+57.02)ATQTLK	89	14	813.4	2	1624.8
**51**	WNLM(+15.99)PPWLTK	89	10	651.4	2	1300.7
**52**	LVM(+15.99)TDELEC(+57.02)LK	89	11	683.9	2	1365.7
**53**	AEFPLLC(+57.02)C(+57.02)NLDVSR	89	14	847.4	2	1692.8
**54**	HC(+57.02)AEDLMPGFLGLYVK	89	16	925.5	2	1848.9
**55**	ELQGALADFLK	89	11	602.8	2	1203.6
**56**	WGAVSVNNR	89	9	501.8	2	1001.5
**57**	QVAFLPFGYLLDQWR	89	15	927.0	2	1852.0
**58**	VVEGTSWYDYLATLGLLK	89	18	1014.5	2	2027.1
**59**	RDALTNLLKC(+57.02)NVNLLNK	89	17	1000.0	2	1998.1
**60**	LVSTC(+57.02)EGC(+57.02)APLLLR	89	14	794.9	2	1587.8
**61**	KRPLLAAGLLLEVK	89	14	761.0	2	1520.0
**62**	KTGTLLGLWDGLSK	88	14	744.9	2	1487.8
**63**	WGGALLGLGSTLLSK	88	15	736.9	2	1471.8
**64**	YPKFVTDLLLWK	88	12	761.9	2	1521.9
**65**	LLSVLGLAALSPEK	88	14	705.9	2	1409.8
**66**	HSWSFDWQHFSNPELR	88	16	1037.0	2	2071.9
**67**	KDKLLGLLMLFQPQK	88	15	886.5	2	1771.0
**68**	RAFLPFGYLLDQWR	88	14	891.5	2	1780.9
**69**	VAFLPFGYLLDQWR	88	14	863.0	2	1723.9
**70**	EGYNWGTC(+57.02)VGLLEGLC(+57.02)WHQK	88	20	1204.1	2	2406.1
**71**	EPVGTLLGLGSTLLSK	88	16	793.0	2	1583.9
**72**	NVSTC(+57.02)EGC(+57.02)APLLLR	88	14	795.4	2	1588.8
**73**	FNEVFWNVPSLLC(+57.02)SLK	88	16	977.0	2	1952.0
**74**	WGTLLGLGSTLLSK	88	14	723.4	2	1444.8
**75**	TLVVQDYTFGKYLGFLK	88	17	996.5	2	1991.1
**76**	TFSGQVFVNADTADLLFR	88	18	1001.0	2	2000.0
**77**	LPTGALLGLGSTLLSK	88	16	771.0	2	1539.9
**78**	LPDGALLGLGSTLLSK	87	16	778.0	2	1553.9
**79**	EEVQLLGLGSTLLSK	87	15	794.0	2	1585.9
**80**	LGFLPFGYLLDQWR	87	14	863.0	2	1723.9
**81**	RYFNLYGDGC(+57.02)FHVK	87	14	888.4	2	1774.8
**82**	LVFTDELEC(+57.02)LKDAVK	87	15	890.5	2	1778.9
**83**	EQSDAAEQEGWFK	87	13	762.8	2	1523.7

**84**	M(+15.99)DNKTLSC(+57.02)ATQTLK	87	14	813.9	2	1625.8
**85**	DYYFNLLHVQGYMK	87	14	895.9	2	1789.8
**86**	TADLSAWTELYDK	87	13	756.9	2	1511.7
**87**	NWGALLGLGSTLLSK	87	15	765.5	2	1528.9
**88**	QSTLAELQVTC(+57.02)FMYR	87	15	923.9	2	1845.9
**89**	AVYM(+15.99)HLYGDGC(+57.02)FHVK	87	15	906.9	2	1811.8
**90**	TRSDAAEKEGWFK	87	13	762.8	2	1523.7
**91**	WGAVSVNAAK	87	10	501.8	2	1001.5
**92**	YRLQTLSC(+57.02)ATQTLK	87	14	841.9	2	1681.9
**93**	WDNNDLLC(+57.02)PFQGHNK	87	15	929.4	2	1856.8
**94**	DWGTLLGLGSTLLSK	87	15	781.0	2	1559.9
**95**	ENGTLLGLGSTLLSK	87	15	751.9	2	1501.8
**96**	SM(+15.99)WPVDLSVGK	87	11	617.8	2	1233.6
**97**	WLKLLGLGSTLLMK(-.98)	86	14	786.5	2	1571.0
**98**	NDDLASVEDLSGK	86	13	681.8	2	1361.6
**99**	AQEGTSWYDYNATLGLLK	86	18	1015.5	2	2029.0
**100**	KVFLPFGYLLDQWR	86	14	891.5	2	1781.0
**101**	AEYEATSDLELLC(+57.02)YSDR	86	17	1018.0	2	2033.9
**102**	NWGTLLGLGSTLLSK	86	15	780.5	2	1558.9
**103**	LGNPLLAAGLLLEVK	86	15	761.0	2	1519.9
**104**	LWQLLGLGSTLLSDK(-.98)	86	15	822.0	2	1641.9
**105**	LTPGTLLGLGSTLLSK	86	16	786.0	2	1569.9
**106**	NVSTC(+57.02)EGC(+57.02)APLLLR	86	14	795.4	2	1588.8
**107**	EDTLLGLGSTDTPK	86	14	723.9	2	1445.7
**108**	WELPWMLQLQVSK	86	13	829.4	2	1656.9
**109**	RMNLLSVVTLC(+57.02)YK	86	13	798.9	2	1595.9
**110**	MAPDLLLALGHSGFPK	86	16	833.9	2	1665.9
**111**	FFYLLGLGSTLLMK(-.98)	86	14	801.5	2	1600.9
**112**	DWGTLLGNGSTLLSK	86	15	781.5	2	1560.8
**113**	VVEGTSWYDYLATLGLLGKSFK	85	22	816.4	3	2446.3
**114**	HC(+57.02)AEDLMPGFLGLYVK	85	16	925.5	2	1848.9
**115**	EAVDLLLALGHSFGPK	85	16	833.9	2	1665.9
**116**	DFNQTLSC(+57.02)ATQTLK	85	14	813.9	2	1625.8
**117**	TPLGTLLGLGSTLLSK	85	16	786.0	2	1569.9
**118**	KWLLGGLGYLAGPK	85	14	736.9	2	1471.9
**119**	WGALLGLGSTLLSK	85	14	708.4	2	1414.8
**120**	RYFNLYGDGC(+57.02)FHVK	85	14	888.4	2	1774.8
**121**	LTPQLLGLGSTLLSK	85	15	771.0	2	1539.9
**122**	LLDTVSNFEGGAVWNAAK	85	18	946.5	2	1890.9
**123**	VAEGGTLLGLGSTLLSK	85	17	808.5	2	1614.9
**124**	AQEGTSWYDYLATLGLLK	85	18	1015.0	2	2028.0
**125**	AFDSC(+57.02)LGDAADKEYLWNDMK	85	20	1175.0	2	2348.0
**126**	EYWNTLLSVGK	85	11	655.4	2	1308.7

**127**	TALDAVLGVVEYNSRLR	85	17	938.5	2	1875.0
**128**	DGDLSAWTESEFR	85	13	756.9	2	1511.7
**129**	YHGTLLGLGSTLLSGK	85	16	809.0	2	1615.9
**130**	KVAGTLLGLGSTLLSK	85	16	779.5	2	1557.0
**131**	VEDM(+15.99)DM(+15.99)LPLLAC(+57.02)VWK	85	15	926.5	2	1850.9
**132**	ENGTLLGLGSTLLSK	85	15	751.9	2	1501.8
**133**	WLQLLGLGSTLLSK	85	14	765.0	2	1527.9
**134**	QLWSFDWQHFSNPELR	85	16	1045.5	2	2089.0
**135**	RLWLGSSLDNHR	85	12	727.4	2	1452.8
**136**	HSSLFMFFK	84	9	572.3	2	1142.6
**137**	LPDGTLLGLGSTLLSK	84	16	793.0	2	1583.9
**138**	FDEVFWNVPSLLMNDK	84	16	977.5	2	1952.9
**139**	QAYEATSDLELLC(+57.02)YSDR	84	17	1017.5	2	2032.9
**140**	KQEGTTATYDYLATLGLLK	84	19	1043.6	2	2085.1
**141**	NGFLPFGYLLDKWR	84	14	863.5	2	1724.9
**142**	KQLTNYDNNLQNLC(+57.02)LPK	84	17	1038.5	2	2075.0
**143**	LVVHPGYDADYNADLALLEM(+15.99)K	84	21	1182.1	2	2362.2
**144**	WNGANLGLGSTLLSK	84	15	766.0	2	1529.8
**145**	M(+15.99)M(+15.99)ELLALDANPNADLALLEM(+15.99)K	84	21	788.7	3	2363.1
**146**	EAGTPELDAAFWR	84	13	731.9	2	1461.7
**147**	AYALQTLSC(+57.02)ATQTLK	84	15	834.9	2	1667.9
**148**	LPFFLLSLVPTALSALWK(-.98)	84	18	672.4	3	2014.2
**149**	QGELPWMLQLKWK	84	13	828.9	2	1655.9
**150**	FADSC(+57.02)LGDAASKEDFLNSMK	84	20	1103.5	2	2205.0
**151**	SQPWPEVLSLESAVK	84	15	835.5	2	1668.9
**152**	FGWNTLLSVGK	84	11	611.3	2	1220.7
**153**	YPLSLVPFLLPSK(-.98)	84	13	737.0	2	1471.9
**154**	WDQLLGNYLAGGPK	84	14	766.5	2	1530.8
**155**	TLSTAVEGPLKDASVATHK	84	19	963.1	2	1924.0
**156**	KGSFTVFVNADTADLLFR	83	18	1001.0	2	2000.0
**157**	NWGTLLGLGSTLLGSK	83	16	809.0	2	1615.9
**158**	KNLPDYSSPQFFDVK	83	15	892.9	2	1783.9
**159**	VVEGTSWYDYLATLGLLK	83	18	1014.5	2	2027.1
**160**	LVVPAYLLGGGDGLTVFK	83	18	910.0	2	1818.0
**161**	EPNPFENDFDVSLK	83	14	825.9	2	1649.8
**162**	FATLQTLSC(+57.02)ATQTLK	83	15	841.9	2	1681.9
**163**	DLDPASLYHVPEGLSYAR	83	18	1002.0	2	2002.0
**164**	EDTLNGLGSTDTPK	83	14	724.4	2	1446.7
**165**	LLSVLGLAALSEPK	83	14	705.9	2	1409.8
**166**	WELPWMLKLKWK	83	12	829.4	2	1656.9
**167**	QYGWTSAALSLWNSRAR	83	17	984.0	2	1966.0
**168**	WNGTLLGLGSTLLSK	83	15	780.5	2	1558.9
**169**	TPLTNYDNDLQNLC(+57.02)LPK	83	17	1010.0	2	2018.0

**170**	ASPGTLLGLGSTLLSK	83	16	757.9	2	1513.9
**171**	HVLSPC(+57.02)PQK	83	9	533.3	2	1064.5
**172**	QKEGTTATYDYNATLGLLK	83	19	1044.1	2	2086.1
**173**	YPLSLVPTALSALK	83	14	737.0	2	1471.9
**174**	NAGAVSVNAAK	82	11	501.3	2	1000.5
**175**	EPALPWM(+15.99)LQLQVSK	82	14	828.4	2	1654.9
**176**	LLDTVGK	82	7	745.4	1	744.4
**177**	YVELLNQVLPLYK	82	13	796.4	2	1590.9
**178**	FWNTLLWGK	82	9	582.8	2	1163.6
**179**	EM(+15.99)AYYNFPDC(+57.02)YNYFGK	82	16	1049.4	2	2096.8
**180**	LLSVGK	82	6	616.4	1	615.4
**181**	RLDTVGK	82	7	788.5	1	787.5
**182**	RYFDLYGDGC(+57.02)FHVK	82	14	888.9	2	1775.8
**183**	KMPGTLLGLGSTLLSSR(-.98)	82	17	865.5	2	1729.0
**184**	TVYEATSDLELLHAESTR	82	18	1018.0	2	2034.0
**185**	LSVGK	82	5	503.3	1	502.3
**186**	WC(+57.02)YYLFPDC(+57.02)YNYFGK	82	15	1048.4	2	2094.9
**187**	AVYFHLYGDGC(+57.02)FHVK	82	15	906.9	2	1811.8
**188**	KAWSNGVGK	82	9	473.8	2	945.5
**189**	TLWGTK	82	6	705.4	1	704.4
**190**	YVLQTLSC(+57.02)ATSASPK	82	15	813.4	2	1624.8
**191**	EM(+15.99)AYYLFPDC(+57.02)YNYFGK	82	16	1048.9	2	2095.9
**192**	LLDTVSNM(+15.99)WGAVWNAAK	82	17	946.5	2	1890.9
**193**	AWNSDLGK	82	8	890.4	1	889.4
**194**	EC(+57.02)WLNLATQLQGQTAGMHDLDK	82	22	843.8	3	2528.2
**195**	FTPYVPGTK	81	9	505.3	2	1008.5
**196**	AQYEATSDLELLC(+57.02)YSDR	81	17	1017.5	2	2032.9
**197**	RLLGYTTPDTMFLSR	81	15	886.0	2	1769.9
**198**	QNDM(+15.99)LNQVLPLYK	81	13	796.4	2	1590.8
**199**	TPWTSWYDYLGATLLLGK	81	18	1043.0	2	2084.1
**200**	FWNTLLSRK	81	9	582.8	2	1163.6
**201**	KVSVLGLAALSPEK	81	14	706.4	2	1410.8
**202**	LWGTLGLLGGMLLSSR(-.98)	81	16	837.0	2	1671.9
**203**	AFDSC(+57.02)LGDAADKEYLWNSMR	81	20	783.7	3	2348.0
**204**	M(+15.99)HQRDALTPLLPVPVVGRVNK	81	21	1178.7	2	2355.3
**205**	TLELFNAGVLPLYK	81	14	789.4	2	1576.9
**206**	FGNEVFWNVPSLLC(+57.02)SLK	81	17	1005.5	2	2009.0
**207**	RGYFNLYGDGC(+57.02)FHVK	80	15	916.9	2	1831.8
**208**	RVVPAYLLGGGDGLTVFK	80	18	931.5	2	1861.0
**209**	FPPWLTK	80	7	888.5	1	887.5
**210**	TLSTAVEGPLKDALGLALR	80	19	963.1	2	1924.1
**211**	NSGTPELDAAFWR	80	13	732.4	2	1462.7
**212**	WGLFDESYGDDRDLSVLLR	80	19	1128.6	2	2255.1

**213**	ALC(+57.02)ANPTAEYTGEYQAK	80	17	943.9	2	1885.9
**214**	FM(+15.99)LLSLVPLLNESK	80	14	810.5	2	1618.9
**215**	SSVNM(+15.99)SWVK	80	9	527.3	2	1052.5
**216**	EGSYM(+15.99)TAYSPK	80	11	625.3	2	1248.5
**217**	VAEAFNTGVLDSDVLQTHEPR	80	21	1149.6	2	2297.1
**218**	AYWPTLLSVGK	80	11	617.8	2	1233.7
**219**	C(+57.02)TWPNDPFTSVSK	80	13	769.8	2	1537.7
**220**	M(+15.99)WGVFWNVPSLLMNDK	80	16	977.0	2	1951.9
**221**	LALENLPLLFSTLAMM(+15.99)K	80	17	961.0	2	1920.0
